# Optimal exercise modalities for enhancing motor function recovery after stroke: a Bayesian systematic review with pairwise and network meta-analyses

**DOI:** 10.1016/j.eclinm.2026.103815

**Published:** 2026-03-05

**Authors:** Liqun Jiang, Huimin Ding, Hyun Seo, Buongo Chun

**Affiliations:** a Graduate School of Physical Education, Myongji University, Yongin, 17058, Republic of Korea; b Department of Sport and Leisure Studies, Graduate School, Korea University, Sejong, 02841, Republic of Korea

**Keywords:** Stroke, Exercise intervention, Network meta-analysis, Motor function recovery, Gait speed, Walking endurance, Balance performance

## Abstract

**Background:**

This study aimed to compare the effectiveness of different exercise interventions in improving motor function recovery after stroke and to identify optimal rehabilitation strategies.

**Methods:**

A comprehensive search of five databases was conducted for randomized controlled trials (RCTs) up to March 13, 2025. Primary outcomes included the 6-min walk test (6MWT), 10-m walk test (10MWT), Berg Balance Scale (BBS), and FMA (total, UE, LE). Pairwise and Bayesian network meta-analyses were performed to compare the relative effects of different exercise modalities. Risk of bias was assessed using the RoB 2 tool, and evidence certainty was rated using CINeMA. This systematic review was prospectively registered in PROSPERO (CRD420251091242).

**Findings:**

A total of 317 RCTs involving 14,464 stroke patients were included. Network meta-analysis showed that, compared with routine care, electrical stimulation plus exercise (ESX) was associated with the largest improvement in walking endurance (MD = 53.4 m, 95% CI 14.27–92.58, p < 0.05, I^2^ = 0%), while aerobic exercise (AE), functional training (FT), gait training (GT), and mind-body exercise (MBE) showed smaller effects. Lower limb training (LLT) demonstrated the greatest improvement in gait speed (MD = 0.18 m/s, 95% CI 0.14–0.23, p < 0.05, I^2^ = 0%), with TCMEX, task-oriented training (TOT), and MBE also showing benefits. For balance (BBS), core stability training (CST) ranked highest (SMD = 0.77, 95% CI 0.30–1.24, p < 0.05, I^2^ = 93.24%), followed by MBE and FT. FT showed the largest pooled effect for total FMA (SMD = 2.15, 95% CI 0.48–3.81, p < 0.05, I^2^ = 93.86%), whereas MBE showed larger pooled effects for FMA-LE (SMD = 1.94, 95% CI 0.04–3.85, p < 0.05, I^2^ = 97.54%) and FMA-UE (SMD = 1.85, 95% CI 1.08–2.63, p < 0.05, I^2^ = 95.57%). Substantial heterogeneity was observed in several pooled comparisons. Meta-regression analyses identified multiple outcome-specific clinical and contextual factors (e.g., intervention duration, stroke phase, stroke severity, routine rehabilitation, age, sex distribution, and socioeconomic setting) that partially explained between-study variability, although no single factor accounted for heterogeneity across all outcomes. Importantly, global network diagnostics indicated acceptable overall model fit and consistency, supporting cautious interpretation of the comparative estimates at the network level.

**Interpretation:**

Several structured exercise interventions were associated with improved motor outcomes after stroke. High heterogeneity was observed in some comparisons; however, meta-regression analyses suggested several potential sources of variability, and global network assessments indicated generally acceptable heterogeneity. These findings should therefore be interpreted cautiously, particularly for outcomes with substantial between-study variation.

**Funding:**

This study received no specific funding.

**Research in context**

**Evidence before this study**

We searched PubMed, Embase, Web of Science, Scopus, and Cochrane Library to March 13, 2025, for RCTs on exercise after stroke. Prior reviews showed that structured exercise improves walking, balance, and motor function, but most were limited to pairwise comparisons, leaving uncertainty about the relative efficacy of modalities and the optimal dose. The dose-response relationship had not been systematically assessed.

**Added value of this study**

This review synthesized 317 RCTs (14,464 patients) evaluating 22 interventions. Using pairwise and network meta-analyses, we ranked exercise modalities across six validated outcomes, with the 6MWT as the primary endpoint. Additional model-based analyses were conducted to explore patterns of intervention effects.

**Implications of all the available evidence**

Structured exercise interventions improve post-stroke motor outcomes and provide a robust evidence base to support individualized, evidence-informed rehabilitation strategies. These findings help inform clinical decision-making by clarifying the relative effectiveness of commonly used exercise modalities, while emphasizing the need to tailor interventions to patient characteristics and rehabilitation context.

## Introduction

Stroke is a leading cause of death and disability worldwide, with prevalence rising most rapidly in low- and middle-income countries.^1^^,^^2^ Survivors frequently experience persistent motor dysfunction, gait instability, and balance impairments that diminish quality of life and impose a substantial healthcare burden.^3^, ^4^, ^5^ Identifying effective strategies to restore function therefore remains a critical challenge in rehabilitation medicine.^6^

Exercise interventions are central to stroke rehabilitation, providing safe, flexible, and cost-effective strategies^7^, ^8^, ^9^, ^10^, ^11^ that enhance cardiopulmonary endurance,^12^^,^^13^ muscular strength,^14^ postural stability,^15^^,^^16^ and neuromotor control.^17^^,^^18^ Their efficacy is typically quantified through internationally recognized functional outcomes encompassing walking capacity,^19^ gait speed,^20^ balance,^21^ and motor recovery.^22^

Despite their widespread application, most existing studies have been limited to pairwise comparisons of single interventions vs. control,^18^^,^^23^^,^^24^ lacking comprehensive head-to-head evaluations across multiple exercise modalities. In addition, the dose–response relationship between exercise and rehabilitation outcomes remains poorly defined; unlike pharmacological treatments, exercise lacks clear thresholds, and both insufficient and excessive volumes may blunt benefits.^13^^,^^25^, ^26^, ^27^, ^28^ Defining this non-linear relationship is crucial to optimize exercise prescriptions, maximize recovery, and minimize ineffective or burdensome training.

To address these gaps, we conducted a comprehensive systematic review with pairwise and network meta-analyses to compare the effects of different exercise modalities on motor function recovery after stroke. The primary objective of this study was to evaluate the comparative effectiveness of commonly used exercise interventions across key functional outcomes, including walking endurance, gait speed, balance, and motor recovery. In addition, we aimed to synthesize the available evidence to inform clinical decision-making by clarifying the relative benefits of different exercise modalities in stroke rehabilitation.

## Methods

### Study design and registration

This review followed PRISMA 2020 guidelines and was registered in PROSPERO (CRD420251091242).^29^

### Literature search

A systematic search of PubMed, Embase, Web of Science, Scopus, and Cochrane Library was conducted to March 13, 2025, for randomized trials of exercise in stroke. Detailed strategies are provided in Supplementary Tables S2.1–S2.5.

### Eligibility criteria

#### Inclusion criteria

Population: Adults (≥18 years) with stroke confirmed by CT or MRI were eligible, irrespective of severity, phase (acute, subacute, chronic), or rehabilitation setting (inpatient or outpatient).

Intervention: Structured exercise programs consistent with the FITT framework, delivered at ≥50 METs-min/week with at least one weekly session. Interventions could be performed in person, via telerehabilitation, or as supervised home-based protocols. Single-session trials were excluded.

Comparator: Routine care, no intervention, or alternative exercise modalities.

Outcomes: At least one validated functional outcome was required: 6MWT, 10MWT, BBS, or FMA (total, UE, LE). Outcomes were extracted at the end of the intervention period.

Study design: Only randomized controlled trials (RCTs) were included.

### Exclusion criteria

Studies were excluded if the intervention category could not be determined after expert review, if exercise dose was unreported, or if stroke-specific data were unavailable. Non-English publications, abstracts, protocols, reviews, inaccessible full texts, and studies lacking sufficient outcome data for effect estimates were also excluded.

### Data extraction

Two reviewers independently extracted data using a standardized template; disagreements were resolved by discussion or adjudication by a third reviewer. Extracted information included study characteristics, and intervention details. Comparator interventions were coded using the same framework. Primary outcomes were the 6MWT, 10MWT, BBS, and FMA (total, UE, LE). Continuous outcomes were summarized as mean differences (MD) or standardized mean differences (SMD) with standard deviations (SD). When SDs were not reported, they were derived from available data in accordance with the Cochrane Handbook.^30^, ^31^, ^32^, ^33^ Data conversions are described in Supplementary 4. Potential effect modifiers were also extracted, and adverse events were recorded when available.

### Exposure definition

Exercise exposures were defined as structured, protocolized training interventions delivered as part of stroke rehabilitation. Interventions were grouped into predefined categories according to their primary training focus, including aerobic, resistance- and balance-oriented training, task-specific and neuromotor approaches, technology-assisted modalities, and adjunctive combined interventions. Control conditions were defined as routine care, consisting of standard medical or physiotherapy services without additional structured exercise, or no-exercise controls where applicable. Detailed definitions of all intervention categories are provided in Supplementary Table S1.

### Data coding and management

Exercise interventions were grouped into predefined categories, including aerobic exercise (AE), high-intensity interval training (HIIT), resistance training (RT), balance training (BT), core stability training (CST), task-oriented training (TOT), constraint-induced movement therapy (CIMT), mind-body exercise (MBE), technology-assisted exercise (TAE), and adjunctive methods such as electrical stimulation plus exercise (ESX).^13^^,^^27^^,^^34^ Full definitions of all 22 exercise modalities and control conditions are provided in Supplementary Table S1. Weekly exercise exposure was summarized in METs-min/week based on reported frequency, duration, and intensity, using standard references,^35^, ^36^, ^37^ and was considered supplementary information to characterize intervention volume.

### Risk of bias assessment

Risk of bias was assessed at the study level using the Cochrane Risk of Bias 2.0 (RoB 2) tool, evaluating five domains (randomization process, deviations from intended interventions, missing outcome data, measurement of outcomes, and selection of reported results). Each trial was classified as low risk, some concerns, or high risk of bias; discrepancies were adjudicated by a third reviewer.^38^

### Certainty of evidence

Certainty of network estimates was assessed using CINeMA, considering six domains (bias, reporting, indirectness, imprecision, heterogeneity, incoherence) and graded per GRADE as high, moderate, low, or very low.^39^

### Statistical analysis

#### Pairwise meta-analysis

Pairwise comparisons were performed with the netmeta package in R, using a frequentist NMA model. Direct evidence was summarized in league table heatmaps, showing mean differences (MDs) with 95% CIs, with effect size and significance visualized by color gradients.^40^

### Network meta-analysis

Both Bayesian and frequentist network meta-analyses were conducted to enhance methodological transparency and verify robustness across statistical frameworks by providing complementary perspectives.^41^, ^42^, ^43^ SMDs were converted in Stata, while MDs were modeled directly, and multi-arm trials were split into pairwise comparisons. Bayesian analysis employed a random-effects model via the gemtc package and JAGS to estimate posterior distributions, SUCRA values, and heterogeneity (I^2^).^44^^,^^45^ In parallel, the netmeta framework generated league table heatmaps,^40^ offering a visual synthesis of treatment comparisons. This dual-framework approach combined probabilistic ranking and heterogeneity assessment from Bayesian inference with transparent visualization from frequentist estimation, ensuring consistent and credible pooled results. Consistency was examined using node-splitting,^46^ To explore potential clinical and contextual sources of between-study heterogeneity, robustness was further assessed through meta-regression analyses incorporating key study- and participant-level characteristics (including sample size, age, intervention duration, and risk of bias), alongside a series of sensitivity analyses.^47^^,^^48^ Sensitivity analyses tested an alternative merged taxonomy (NE→RC, TOT/BT→FT, HIIT→AE, RAT/VRG→TAE) and excluded upper-extremity interventions (e.g., ULT, CIMT) to evaluate the impact of clinical classification and intervention scope on pooled estimates and heterogeneity patterns. Publication bias was evaluated using funnel plots, Egger's test, and trim-and-fill correction.^49^

### Dose–response network meta-analysis

Dose–response relationships were evaluated as a supplementary analysis rather than a primary analytical objective. A Bayesian model-based network meta-analysis framework was applied to explore potential associations between exercise volume, summarized as weekly METs-min, and functional outcomes while accounting for intervention type.^50^, ^51^, ^52^, ^53^ In line with WHO guidance,^54^ 600 METs-min/week was used as a reference value to aid interpretation. These analyses were intended to provide supportive, exploratory insights.^55^, ^56^, ^57^

### Ethics approval

Not applicable.

### Consent to participate

Not applicable.

### Consent for publication

Not applicable.

### Code availability

Not applicable.

### Role of the funding source

This study received no specific funding. The funding source had no role in study design, data collection, analysis, interpretation, manuscript preparation, or the decision to submit for publication.

## Results

### Study selection and characteristics

**Fig. 1:**
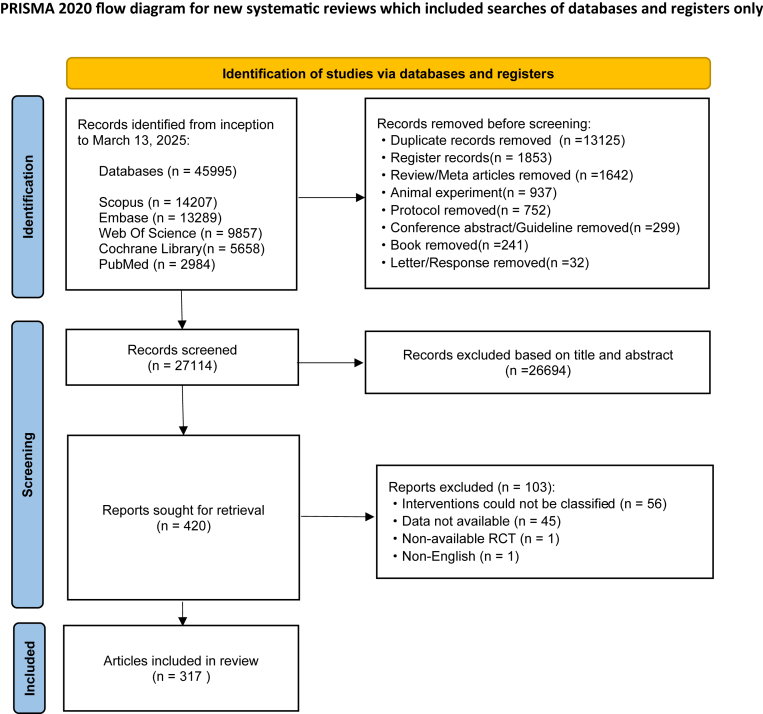
PRISMA flow diagram of study selection. Flowchart illustrating the identification, screening, eligibility assessment, and inclusion of randomized controlled trials, resulting in 317 studies retained for the final analysis. ∗Consider, if feasible to do so, reporting the number of records identified from each database or register searched (rather than the total number across all databases/registers). ∗∗If automation tools were used, indicate how many records were excluded by a human and how many were excluded by automation tools.^58^ For more information, visit: http://www.prisma-statement.org.

We included 317 RCTs involving 14,464 stroke patients (Fig. 1). The 6MWT was the primary outcome, with 10MWT, BBS, and FMA (total, UE, LE) as secondary endpoints. Most participants were aged 40–70 years, with balanced sex distribution; 55.6% had mild-moderate stroke, and 63.0% were in the subacute phase. Injury sites, intervention settings, and diagnostic methods were heterogeneous but well characterized. 22 exercise modalities, standardized by METs-min/week, were compared against routine care. Screening adhered to predefined criteria, with exclusions detailed in Supplementary Table S3. Detailed study characteristics are summarized in Supplementary Table S5.

### Risk of bias assessment

All 317 included RCTs were evaluated at the study level using the RoB 2 tool (Supplementary Table S6). At the study level, the overall risk-of-bias judgment was predominantly classified as “some concerns,” with a smaller proportion of studies rated as low risk and a limited number judged to be at high risk. Overall, most trials were judged to be at low risk of bias for the randomization process and completeness of outcome data. However, a substantial proportion of studies showed some concerns or high risk related to deviations from intended interventions and outcome measurement, primarily due to the lack of blinding of participants, personnel, or outcome assessors. High risk of bias at the study level was mainly driven by serious deviations from intended interventions or high risk in outcome measurement domains. Concerns regarding selective reporting and unclear trial preregistration were also observed in a subset of studies. The overall risk-of-bias profile was considered acceptable for evidence synthesis and was taken into account when interpreting pooled estimates.

### Certainty of evidence (CINeMA assessment)

Evidence certainty (CINeMA) was generally “moderate” to “high,” especially for CST, MBE, and RAT in 6MWT and BBS. Some comparisons were downgraded to “low” or “very low” due to inconsistency, small samples, or imprecision, such as FMA-UE contrasts based on single studies (Supplementary 7, Tables S7.1–S7.6).

### Pairwise and network meta-analysis results

#### 6MWT

**Fig. 2:**
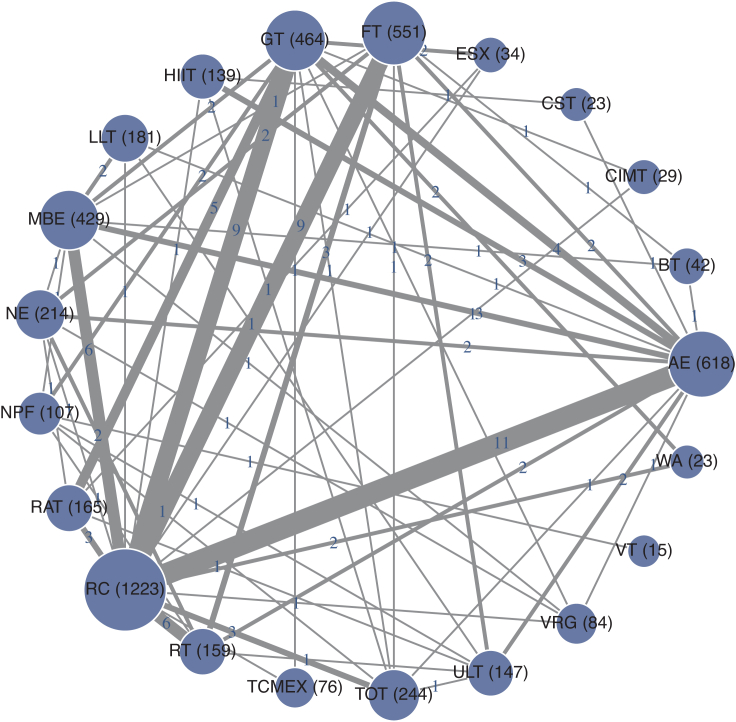
6MWT network plot of exercise interventions. Network geometry of 22 exercise modalities for stroke rehabilitation. Each node represents an intervention, with node size proportional to the number of participants. Edge thickness reflects the number of direct comparisons between interventions.

**Fig. 3:**
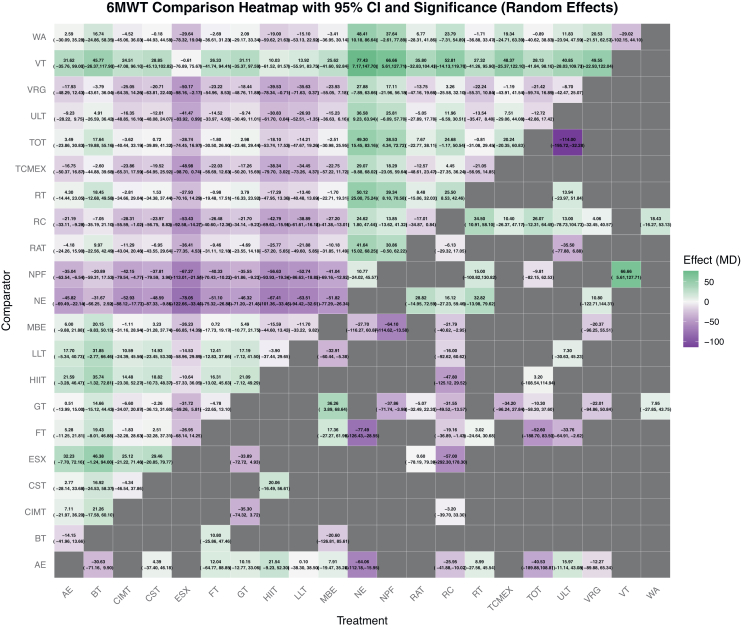
6MWT pairwise comparison heatmap. Heatmap displaying mean differences (MDs) with 95% confidence intervals between interventions. Color coding indicates the direction and magnitude of benefit: green denotes greater benefit, purple denotes lesser benefit, and gray indicates unavailable comparisons.

The network diagram for the 6MWT is shown in Fig. 2. Pairwise meta-analysis showed that AE (MD = 25.95 m, 95% CI: 10.02–41.88 m, p < 0.05, I^2^ = 96.72%), FT (MD = 19.16 m, 95% CI: 1.43–36.89 m, p < 0.05, I^2^ = 48.98%), Gait training (GT) (MD = 31.55 m, 95% CI: 13.57–49.52 m, p < 0.05, I^2^ = 96.74%), and MBE (MD = 21.79 m, 95% CI: 2.95–40.62 m, p < 0.05, I^2^ = 96.68%) significantly improved performance compared with RC. Network meta-analysis further indicated that ESX provided the largest improvement (MD = 53.4 m, 95% CI: 14.27–92.58 m, p < 0.05, I^2^ = 0%), with the highest SUCRA ranking (Supplementary 13, Figure S13.1), followed by HIIT (MD = 42.79 m, 95% CI: 15.95–69.63 m, p < 0.05, I^2^ = 0%) and LLT (MD = 38.89 m, 95% CI: 16.18–61.61 m, p < 0.05, I^2^ = 98.78%) (Fig. 3).

#### 10MWT

For the 10MWT, the network structure is shown in Supplementary 11 (Figure S11.1). Pairwise meta-analysis indicated that MBE (MD = 0.11 m/s, 95% CI: 0.08–0.15 m/s, p < 0.05, I^2^ = 70.29%), GT (MD = 0.10 m/s, 95% CI: 0.05–0.15 m/s, p < 0.05, I^2^ = 0%), FT (MD = 0.08 m/s, 95% CI: 0.04–0.12 m/s, p < 0.05, I^2^ = 26.81%), and CIMT (MD = 0.04 m/s, 95% CI: 0.01–0.07 m/s, p < 0.05, I^2^= NA %) significantly improved walking speed compared with routine care. Network meta-analysis further identified LLT as the most effective intervention (MD = 0.18 m/s, 95% CI: 0.14–0.23 m/s, p < 0.05, I^2^ = 0%), with the highest SUCRA ranking (Supplementary 13, Figure S13.2), followed by TCMEX (MD = 0.15 m/s, 95% CI: 0.12–0.19 m/s, p < 0.05, I^2^ = 76.35%) and TOT (MD = 0.14 m/s, 95% CI: 0.11–0.17 m/s, p < 0.05, I^2^ = 0%) (Supplementary 12, Figure S12.1). These findings suggest that interventions targeting lower limb strength and functional integration may yield superior improvements in gait speed.

#### BBS

For the BBS, the network evidence plot is presented in Supplementary 11, Figure S11.2. Pairwise meta-analysis showed significant improvements with CST (SMD = 1.30, 95% CI: 0.62–1.99, p < 0.05, I^2^ = 94.80%), FT (SMD = 0.87, 95% CI: 0.24–0.50, p < 0.05, I^2^ = 91.98%), and MBE (SMD = 2.53, 95% CI: 1.86–3.20, p < 0.05, I^2^ = 96.95%). Network meta-analysis further identified CST as the most effective intervention (SMD = 0.77, 95% CI: 0.30–1.24, p < 0.05, I^2^ = 93.24%), ranking highest in SUCRA values (Supplementary 13, Figure S13.3), followed by MBE (SMD = 1.37, 95% CI: 1.01–1.73, p < 0.05, I^2^ = 96.42%) and FT (SMD = 1.21, 95% CI: 0.77–1.66, p < 0.05, I^2^ = 88.23%) (Supplementary 12, Figure S12.2).

#### FMA series

For the total FMA score, the network plot is shown in Supplementary 11 (Figure S11.3). Pairwise meta-analysis indicated that FT significantly improved overall motor function (SMD = 2.15, 95% CI: 0.48–3.81, p < 0.05, I^2^ = 93.95%). Consistently, network meta-analysis also identified FT as the most effective intervention (SMD = 2.15, 95% CI: 0.48–3.81, p < 0.05, I^2^ = 93.86%), with the highest SUCRA ranking, followed by CST and MBE (Supplementary 12, Figure S12.3; Supplementary 13, Figure S13.4).

For the FMA-LE, the network plot is presented in Supplementary 11 (Figure S11.4). Pairwise analysis showed that MBE had a robust effect (SMD = 4.16, 95% CI: 2.83–5.49, p < 0.05, I^2^= NA %), and network meta-analysis confirmed MBE as the most effective (SMD = 1.94, 95% CI: 0.04–3.85, p < 0.05, I^2^ = 90.76%), followed by AE and water-based exercise (WA) (Supplementary 12, Figure S12.4; Supplementary 13, Figure S13.5).

For the FMA-UE, network structure is shown in Figure S11.5. MBE also showed significant benefits in both pairwise (SMD = 2.29, 95% CI: 0.92–3.67, p < 0.05, I^2^ = 98.90%) and network analysis (SMD = 1.85, 95% CI: 1.08–2.63, p < 0.05, I^2^ = 97.54%), followed by CIMT (SMD = 1.53) and CST (SMD = 1.88) (Supplementary 12, Figure S12.5; Supplementary 13, Figure S13.6).

#### Consistency and heterogeneity assessments

Network models showed good overall fit. Consistency models outperformed inconsistency models for all outcomes except 10MWT (ΔDIC <0.2; Supplementary 8). Node-splitting analyses confirmed general agreement between direct and indirect evidence, with only a limited number of contrasts showing statistically significant inconsistency (Supplementary 9).

Across all pooled comparisons relative to the reference condition (routine care, RC), between-study heterogeneity varied across outcomes and contrasts. While global network-level assessments indicated acceptable heterogeneity and good overall model fit, moderate to high heterogeneity was observed for certain BBS and RC-related contrasts, suggesting that variability in intervention implementation, patient characteristics, and study context contributed to between-study differences. Outcome-specific I^2^ statistics with corresponding 95% confidence intervals are reported in Supplementary Table S10 (Tables S10.1–S10.6). Accordingly, although the network meta-analysis demonstrated acceptable global model fit and consistency, the presence of moderate to high heterogeneity in several pairwise and network comparisons indicates that the magnitude and ranking of intervention effects should be interpreted with caution.

#### Dose–response meta-analysis

Dose–response analyses showed preliminary, outcome-specific patterns (Supplementary 19). For walking endurance (6MWT), the relationship suggested a nonlinear trend, with larger effects at moderate volumes around 1000 METs-min/week, while uncertainty increased at higher doses. Gait speed (10MWT) generally increased across the observed dose range, whereas balance performance (BBS) improved beyond ∼360 METs-min/week before plateauing (Supplementary 19).

Motor recovery outcomes showed greater variability. Total FMA tended to show larger effects at higher volumes, FMA-LE suggested a possible inverted U-shaped pattern with peak estimates around 1000–1200 METs-min/week, and FMA-UE showed improvements mainly at lower doses, with little additional gain beyond 500–1000 METs-min/week (Supplementary 19).

Model selection favored Emax models for 6MWT and 10MWT and restricted cubic spline models for BBS and FMA outcomes (Supplementary 20). Data at higher dose ranges were sparse and estimates imprecise. Intervention-specific results showed larger estimated effects for ESX on 6MWT and for TAE and VRG on 10MWT, while FT and TOT showed more consistent estimates for BBS; in the FMA series, TOT and CST were more stable at moderate volumes, whereas MBE and ULT were more variable (Supplementary 21–22). Overall, these findings should be interpreted as exploratory and preliminary.

#### Meta-regression and sensitivity analyses

Meta-regression identified several moderators (Supplementary 16, Table S16; Supplementary 17). For the 6MWT, intervention duration, the presence vs. absence of concomitant routine rehabilitation, and stroke severity were significant factors. For the 10MWT, duration and stroke phase (subacute > chronic) influenced effects. BBS outcomes were moderated by GDP, sex ratio, and sample size, while total FMA was affected by age, sex ratio, and stroke phase. No significant covariates were found for FMA-LE or FMA-UE. These outcome-specific effects showed no consistent pattern, supporting the transitivity assumption. Importantly, these analyses suggest that clinical and contextual variables explained heterogeneity only partially, and substantial residual between-study variability remained across several outcomes, indicating that pooled estimates should be interpreted cautiously. Leave-one-out analyses confirmed robustness for 6MWT, 10MWT, BBS, and FMA (Supplementary 18, Tables S18.1–S18.11). Alternative taxonomy analyses (NE→RC, TOT/BT→FT, HIIT→AE, RAT/VRG→TAE) yielded similar results with only minor SUCRA shifts (Tables S18.12–S18.17). Excluding upper-extremity interventions caused moderate SUCRA changes but did not alter conclusions; total FMA could not be re-analyzed due to network disconnection (Tables S18.18–S18.22).

#### Publication bias

Egger's test indicated bias only for 6MWT (Supplementary 14). Trim-and-fill analysis showed minimal impact on effect sizes (e.g., AE vs. RC, MBE vs. RC), with consistent directionality. No significant bias was detected for other outcomes, supporting overall robustness (Supplementary 15).

#### Adverse events

Adverse events were inconsistently reported across included trials. Where available, most events were mild and transient (e.g., muscle soreness, fatigue), and no serious adverse events were directly attributed to the exercise interventions. However, the majority of studies did not provide systematic safety data, precluding pooled analyses.

## Discussion

Previous studies have consistently reported that gait training (GT), aerobic exercise (AE), and functional training (FT) are associated with improvements in walking endurance after stroke,^59^^,^^60^ and the present findings are broadly consistent with this literature. However, the comparative effectiveness of different exercise modalities remains debated, as several reviews have noted that no single intervention is uniformly superior and that treatment effects vary substantially across studies with different populations and intervention settings.^28^^,^^61^ This variability is also reflected in the between-study heterogeneity observed in the current analysis. Some trials and reviews have suggested that combining active exercise with adjunctive strategies may be associated with larger improvements in 6MWT performance.^62^^,^^63^ At the network level, our results are compatible with this pattern, although meta-regression analyses indicated that effect estimates differed according to clinical and contextual factors, including intervention duration, stroke severity, and the presence or absence of routine rehabilitation. These findings suggest that background rehabilitation and patient characteristics may partially modify the observed effects of exercise interventions on walking endurance, contributing to residual heterogeneity. Exploratory dose–response analyses further suggested a nonlinear association between exercise volume and walking endurance, with larger estimated effects observed at moderate exercise volumes (approximately 1000 METs-min/week), while estimates became increasingly uncertain at higher doses. These dose-related patterns should be interpreted cautiously, as data at higher volumes were sparse and confidence intervals were wide. From a mechanistic perspective, improvements in walking endurance likely arise from the interaction of multiple processes rather than a single dominant pathway. Task-specific practice in GT and FT may support motor relearning and activity-dependent neural plasticity,^64^^,^^65^ whereas AE may enhance cardiovascular capacity and metabolic efficiency during sustained walking.^28^ Adjunctive approaches such as electrical stimulation may influence afferent input and neuromuscular activation,^66^^,^^67^ and increased attention and engagement during combined or assisted training may also contribute to functional gains.^68^ The relative contribution of these mechanisms likely varies across study contexts, which may help to explain the heterogeneity observed across trials.

Previous randomized trials and systematic reviews have reported that high-intensity interval training (HIIT), water-based exercise, and mind–body exercise are associated with improvements in gait speed after stroke,^69^, ^70^, ^71^ and our findings are broadly consistent with this literature. However, prior studies have also highlighted substantial variability in gait speed outcomes, with no single intervention demonstrating consistent superiority across different study settings and populations.

Within this context, our network meta-analysis suggests that lower-limb training and traditional Chinese mind–body exercise (TCMEX), which have been less frequently examined in earlier reviews, may also be associated with improvements in 10MWT performance.^72^, ^73^, ^74^ Nevertheless, effect estimates varied across studies, and these findings should be interpreted as preliminary. Meta-regression analyses further indicated that intervention duration and stroke phase (subacute vs. chronic) were associated with variability in observed effects, suggesting that differences in training exposure and recovery stage may partly contribute to the observed heterogeneity in gait speed outcomes. Consistent with previous reports of greater responsiveness during the subacute phase compared with the chronic phase,^75^^,^^76^ these findings highlight the importance of stroke phase when interpreting gait speed responses to exercise interventions.

Exploratory dose–response analyses suggested a generally increasing trend in gait speed across the observed exercise volume range, although uncertainty increased at higher doses due to sparse data. This pattern indicates that gait speed improvements may be sensitive to accumulated training exposure; however, the available evidence is insufficient to define a clear dose threshold, and dose-related findings should therefore be interpreted cautiously. From a mechanistic perspective, improvements in gait speed likely arise through multiple complementary processes. Targeted lower-limb training may enhance muscle strength and rate of force development, facilitating faster step initiation and propulsion.^77^ Task-specific and repetitive gait practice may improve neuromuscular coordination and temporal control of lower-limb movements.^78^ Aerobic and interval-based exercise may increase metabolic efficiency and fatigue resistance, enabling higher walking speeds over short distances,^69^^,^^70^Mind–body exercise may additionally influence gait speed through improvements in postural control, attentional regulation, and movement integration, while technology-assisted interventions such as virtual reality may enhance sensory feedback and task engagement during gait training.^79^

This study showed that several exercise interventions were associated with improvements in balance as measured by the BBS, consistent with previous evidence highlighting the importance of core control, postural stability, and body awareness training in post-stroke rehabilitation.^16^^,^^80^ However, balance-related outcomes varied substantially across studies, indicating notable between-study heterogeneity.

Meta-regression analyses suggested that part of this variability was related to contextual and study-level characteristics. Larger BBS improvements were more frequently observed in studies conducted in higher-GDP settings and in samples with a higher proportion of female participants, whereas studies with larger sample sizes tended to report smaller pooled effects. These findings suggest that rehabilitation context, participant composition, and study design may influence observed balance outcomes and partly account for the heterogeneity reported across trials. From a mechanistic perspective, task-oriented training (TOT) may improve balance by embedding postural control demands within functional activities and reinforcing repetition-based motor learning.^81^^,^^82^ Core stability training (CST) and functional training (FT) may enhance balance through improved trunk control, proximal stability, and multisensory integration.^83^, ^84^, ^85^ Mind-body exercise (MBE) may additionally act through central regulation and attentional control mechanisms, which may contribute to the variability observed across studies.^17^^,^^86^^,^^87^ Exploratory dose–response analyses suggested that balance improvements tended to emerge beyond moderate exercise volumes (approximately ≥360 METs-min/week) before plateauing, although data at higher volumes were limited and estimates imprecise. Accordingly, both modality- and dose-related findings for BBS should be interpreted cautiously in light of the observed heterogeneity.

Previous studies have shown that functional training (FT), core stability training (CST), and mind-body exercise (MBE) are associated with improvements in overall motor function after stroke, as reflected by FMA scores.^88^^,^^89^ Our findings are broadly consistent with this literature, but effect sizes varied markedly across studies, indicating substantial between-study heterogeneity. FT is commonly understood to facilitate motor recovery through task-specific practice and neural reorganization across limb segments, which may explain its relatively consistent association with global FMA outcomes.^88^^,^^89^ Meta-regression further suggested that part of this variability was related to participant characteristics, particularly age, sex distribution, and stroke phase, although a large proportion of heterogeneity remained unexplained.

In contrast, evidence for MBE has been mixed. Prior studies suggest that its effectiveness may depend on cognitive engagement, attentional demands, and individual responsiveness,^90^^,^^91^ and our analysis similarly identified variable and unstable effect patterns for this modality across studies.^92^ Exploratory dose–response analyses suggested that larger total FMA gains tended to occur at higher accumulated exercise volumes; however, data at higher dose ranges were sparse and associated with wide uncertainty, limiting confidence in any apparent dose-related trend. Overall, these findings suggest that global motor recovery is more likely supported by integrative, multi-component rehabilitation strategies rather than reliance on a single exercise modality.

For lower-limb motor function (FMA-LE), earlier work has highlighted potential benefits of MBE through mechanisms related to proprioception, postural control, and sensorimotor integration.^18^ While our results were generally in line with these observations, heterogeneity was substantial and confidence intervals were wide, indicating considerable uncertainty across studies.^17^^,^^93^^,^^94^ Dose–response analyses suggested a possible inverted U-shaped pattern, with peak estimates at moderate exercise volumes (approximately 1000–1200 METs-min/week), followed by attenuation at higher doses; however, meta-regression did not identify significant moderators for FMA-LE, suggesting that sources of heterogeneity were not adequately explained by the available covariates. Aerobic exercise (AE) and water-based exercise (WA) showed comparatively more stable estimates, possibly due to more standardized movement execution and repetitive motor activation,^95^, ^96^, ^97^ although the overall strength of evidence remains limited. Neurophysiological facilitation (NPF) showed short-term benefits in a small number of trials, but imprecision precluded firm conclusions.

Regarding upper-limb motor recovery (FMA-UE), previous research has focused on MBE, CIMT, and CST, with highly variable findings.^94^^,^^98^ CIMT is supported by mechanistic evidence related to forced use and cortical reorganization,^99^^,^^100^ yet current pooled data did not demonstrate clear dose-dependent benefits, and effect estimates varied substantially across interventions and studies. Exploratory dose–response analyses suggested that FMA-UE improvements were more commonly observed at lower to moderate exercise volumes (approximately 500–1000 METs-min/week), with little additional gain at higher doses, but these patterns were unstable and based on limited data. The absence of significant moderators in meta-regression further suggests that upper-limb recovery may be influenced by unmeasured factors, such as task specificity, therapist involvement, or adherence, rather than exercise dose alone.

Overall, several exercise modalities, including ESX, HIIT, FT, MBE, CST, and LLT, were associated with functional improvements compared with routine care across multiple outcome domains, although no single modality can be considered universally optimal. These findings support the use of combined, task-oriented and neuromuscularly engaging approaches in stroke rehabilitation. Across outcomes, benefits were more often observed at moderate exercise volumes, whereas effects at very low or very high volumes were inconsistent. Given the substantial heterogeneity and uncertainty in the dose–response patterns, these observations should be interpreted as indicative rather than prescriptive, and do not define precise dose thresholds for clinical decision-making. Exercise prescription should therefore be individualized, taking into account stroke severity, clinical phase, comorbidities, baseline function, and tolerance, with gradual progression in the acute stage, structured but tolerable workloads in the subacute stage, and sustainable moderate–intensity activity in the chronic stage. Future studies using individual participant data meta-analyses and rigorously stratified randomized trials are needed to better clarify how exercise type, volume, and patient characteristics interact to influence post-stroke functional recovery.^101^^,^^102^

Despite the large number of included trials, several limitations should be acknowledged. First, only six functional outcomes provided sufficient data for dose–response analyses, which limits the generalizability of these findings across broader rehabilitation domains. Second, although overall model fit and global network heterogeneity were acceptable, moderate to high heterogeneity was observed for several specific interventions and outcome contrasts, indicating substantial between-study variability. To address this, meta-regression and sensitivity analyses were conducted to explore potential clinical and contextual sources of heterogeneity; however, only a subset of the observed variability could be explained, and residual heterogeneity remained in several analyses. Third, the precision of several estimates was constrained by small study sizes, heterogeneous reporting practices, and imperfect classification of exercise interventions, contributing to uncertainty in some comparisons. Exercise dose was derived from reported frequency, duration, and intensity, which may not fully capture true training exposure and could introduce misclassification. In addition, a proportion of trials were judged to have some concerns or high risk of bias, and the analyses were based on group-level averages rather than individual-level responses, limiting inferences about personalized treatment effects. Adverse events were infrequently and inconsistently reported, precluding firm conclusions regarding the safety profile of higher training volumes. Future research should incorporate more detailed and standardized reporting of intervention characteristics, systematically examine additional potential moderators, and apply individual-level analytic approaches to better explain heterogeneity in treatment response and improve clinical applicability.

### In conclusion

This study synthesized evidence from 317 RCTs using pairwise, network, and dose–response meta-analyses to compare 22 exercise interventions across six functional outcomes in stroke rehabilitation. Compared with routine care (RC), ESX, HIIT, FT, MBE, CST, and LLT showed relatively favorable effects across multiple domains, although the certainty of evidence varied by outcome and intervention. Exploratory dose–response analyses at the population level suggested that benefits were commonly observed within a moderate exercise volume range, most often between approximately 500 and 1200 METs-min/week. These estimates should be interpreted as average patterns rather than definitive thresholds, as the available data were limited and individual responses varied substantially across studies. Therefore, exercise prescriptions should not rely on fixed dose targets but instead be individualized according to stroke severity, clinical phase, safety, tolerability, and adherence, to ensure both effectiveness and feasibility in real-world rehabilitation practice.

## Contributors

Liqun Jiang contributed to the conceptualization, methodology, formal analysis, data curation, original draft preparation, and visualization, had full access to all the data in the study, and takes responsibility for the integrity of the data and the accuracy of the data analysis.

Huimin Ding participated in methodology development, formal analysis, investigation, and writing—review and editing.

Hyun Seo was involved in methodology, formal analysis, and data curation, and verified the extracted data and analytical results.

Buong-O Chun supervised the project, contributed to the conceptualization and project administration, and verified the underlying data and analytical procedures, and assisted with writing—review and editing.

Hyun Seo and Buong-O Chun contributed equally to this article.

All authors have read and approved the final version of the manuscript. Buong-O Chun is the corresponding author.

## Data sharing statement

All data supporting the findings of this study are available within the paper and its supplementary materials.

## Declaration of interests

All authors declare that they have no conflicts of interest.
