# Supplementary material for: Optimal exercise modalities for enhancing motor function recovery after stroke: a Bayesian systematic review with pairwise and network meta-analyses
Source: eClinicalMedicine. 2026 Mar 5;93:103815. doi: 10.1016/j.eclinm.2026.103815 (PMC12972736; doi:10.1016/j.eclinm.2026.103815)
Supplement: Appendix [file mmc1.docx]

**Supplementary**

[Supplementary 1 — Definitions of exercise training interventions 1](#_Toc16609)

[Supplementary 2 — Search Strategy 3](#_Toc32039)

[Supplementary 3 — Excluded Studies List 7](#_Toc20114)

[Supplementary 4 — Conversion Formulas for Effect Size and SD Estimation 17](#_Toc6219)

[Supplementary 5 — Study Characteristics of Included Studies 20](#_Toc20257)

[Supplementary 6 — Risk of Bias Assessment (RoB 2.0) 149](#_Toc17615)

[Supplementary 7 — GRADE Assessment for Network Evidence 162](#_Toc21155)

[Supplementary 8 — Global Inconsistency Test (Design-by-Treatment Interaction Model) 206](#_Toc25660)

[Supplementary 9 — Node-Splitting Results for Local Inconsistency 207](#_Toc10019)

[Supplementary 10 — Heterogeneity Assessment (I² Statistics) 215](#_Toc15503)

[Supplementary 11 — Network Plots for All Outcomes 223](#_Toc21078)

[Supplementary 12 — Network Meta-Analysis Heatmaps (Direct Comparisons) 226](#_Toc32141)

[Supplementary 13 — Overall SUCRA Rankings 231](#_Toc10802)

[Supplementary 14 — Funnel Plots for Publication Bias 234](#_Toc32147)

[Supplementary 15 — Trim and Fill Adjustments for Publication Bias 237](#_Toc2806)

[Supplementary 16 — Meta-Regression Results 239](#_Toc20972)

[Supplementary 17 — Network Meta-Regression Bubble Plots 241](#_Toc24220)

[Supplementary 18 — Leave-one-out Sensitivity Analysis 250](#_Toc18179)

[Supplementary 19 — Total Dose Comparison 285](#_Toc2266)

[Supplementary 20 — Dose–Response Model Selection 299](#_Toc7905)

[Supplementary 21 — Treatment–Dose Level Connection Diagram 300](#_Toc9236)

[Supplementary 22—Dose–Response by Exercise Type 306](#_Toc28887)

[References: 309](#_Toc7915)

**Supplementary 1 — Definitions of exercise training interventions**

**Table S1 Definitions of exercise training interventions included in this systematic review**

| Abbreviation | Full Term | Definition |
| --- | --- | --- |
| RC | Routine care | Usual or standard care provided in clinical settings, including conventional medical or physiotherapy services, but without any additional structured exercise program^1^. |
| NE | No exercise | Control condition in which participants do not receive any structured exercise intervention and continue their usual lifestyle activities^2^. |
| NPF | Neuro/proprioceptive facilitation | Interventions such as proprioceptive neuromuscular facilitation (PNF) techniques that enhance neuromuscular control and proprioceptive input to improve posture, coordination, and motor function^3^. |
| ULT | Upper limb training | Structured exercise programs targeting the upper extremity, aiming to improve strength, coordination, dexterity, and functional use of the arm and hand^4^. |
| LLT | Lower limb training | Structured exercise programs focused on the lower extremity, designed to improve strength, endurance, balance, gait, and mobility. |
| CST | Core stability training | Exercises that strengthen deep and superficial trunk muscles (e.g., abdominals, spinal extensors, pelvic stabilizers) to enhance posture, spinal control, and trunk stability^5^. |
| BT | Balance training | A set of static and dynamic exercises that challenge postural control, center of mass regulation, and equilibrium reactions, aiming to reduce fall risk and improve stability^6^. |
| WA | Water-based activity | Exercise performed in aquatic environments (e.g., shallow or deep pools) that uses buoyancy and resistance of water to reduce joint loading and enhance aerobic capacity, strength, and mobility^7^. |
| VT | Vibration training | Whole-body or localized vibration delivered through platforms or devices to stimulate neuromuscular activation, increase strength, and improve balance^8^. |
| ESX | Electrical stimulation plus exercise | Combination of neuromuscular electrical stimulation (NMES or functional electrical stimulation, FES) with voluntary movement practice to facilitate muscle activation and motor recovery^9^. |
| RAT | Robotic-assisted training | Use of robotic or exoskeleton devices to provide guided, repetitive, or assisted limb movements during rehabilitation, often applied for walking or upper-limb recovery^10^. |
| VRG | Virtual reality gaming | Rehabilitation interventions that use immersive or non-immersive virtual environments or interactive gaming platforms to provide task-oriented, motivating, and feedback-based motor training^11^. |
| AE | Aerobic exercise | Continuous, rhythmic large-muscle activities (e.g., walking, cycling, treadmill running) performed at prescribed intensities to improve cardiovascular fitness and metabolic health^12, 13^. |
| HIIT | High-intensity interval training | Training consisting of repeated short bouts of vigorous exercise (≥80% HRmax or VO₂max) interspersed with periods of low-intensity exercise or rest^14^. |
| GT | Gait training | Task-specific interventions aimed at improving walking ability, including treadmill or overground walking, stepping tasks, and cueing strategies to enhance gait speed, symmetry, and endurance^15^. |
| FT | Functional training | A training approach composed of multi-joint, multi-planar, task-specific movement patterns that mirror daily living or functional tasks, aiming to enhance strength, neuromuscular coordination, and the ability to transfer gains into functional independence^16^. |
| RT | Resistance training | Exercises using external loads (free weights, machines, resistance bands, or bodyweight) to improve muscular strength, endurance, and hypertrophy, typically prescribed by sets, repetitions, and intensity^12, 13^. |
| MBE | Mind–body exercise | Mind–Body Exercise / Mind–Body Interventions：Integrative practices that combine physical movement, conscious breathing / respiratory control, and directed mental focus (e.g. attention, awareness, imagery, visualization), intending to coordinate body–mind interaction and modulate sensorimotor and cognitive networks. Examples include Tai Chi, Yoga, Qigong, Pilates, and extended modes such as motor imagery, mirror-based techniques, and movement observation / internal simulation. The emphasis is on body awareness, mind–body coordination, and the capacity to engage neural modulation beyond mere biomechanical movement^17, 18^. |
| TCMEX | Traditional Chinese medicine combined with exercise | Integrative interventions combining traditional Chinese medicine practices (e.g., acupuncture, herbal therapy, massage) with structured exercise programs^19^. |
| TAE | Technology-assisted exercise | Exercise interventions supported by advanced technologies such as wearable sensors, biofeedback systems, exoskeletons, or tele-rehabilitation tools to guide and monitor training^20, 21^. |
| TOT | Task-oriented training | High-repetition, goal-directed practice of specific motor tasks relevant to daily activities (e.g., grasping a cup, climbing stairs, sit-to-stand) to promote functional recovery^21^. |
| CIMT | Constraint-induced movement therapy | Rehabilitation approach that restricts the use of the unaffected limb while requiring intensive, repetitive practice of the affected limb to overcome learned non-use and improve functional performance^22^. |

**Supplementary 2 — Search Strategy**

**Table S2.1 Pubmed 2984**

| Search Number | Search Details |
| --- | --- |
| 1 | "Stroke"[MeSH Terms] |
| 2 | "Strokes"[Title/Abstract] OR "cerebrovascular accident"[Title/Abstract] OR "brain vascular accident"[Title/Abstract] |
| 3 | "Exercise"[MeSH Terms] |
| 4 | "Exercises"[Title/Abstract] OR "physical activity"[Title/Abstract] OR "physical activities"[Title/Abstract] OR "fitness training"[Title/Abstract] OR "walking"[Title/Abstract] OR "Jogging"[Title/Abstract] OR "Bicycling"[Title/Abstract] OR "high intensity interval training"[Title/Abstract] OR "high intensity interval trainings"[Title/Abstract] OR "high intensity interval training"[Title/Abstract] OR "HIIT"[Title/Abstract] OR "Dancing"[Title/Abstract] OR "Resistance"[Title/Abstract] OR "Strength"[Title/Abstract] OR "aquatic therapy"[Title/Abstract] OR "aquatic exercise therapy"[Title/Abstract] OR "water exercise therapy"[Title/Abstract] OR "ai chi therapy"[Title/Abstract] OR "Swimming"[Title/Abstract] OR "mind body therapies"[Title/Abstract] OR "Yoga"[Title/Abstract] OR "pilates training"[Title/Abstract] OR "tai ji"[Title/Abstract] OR "Qigong"[Title/Abstract] OR "baduanjin"[Title/Abstract] OR "wuqinxi"[Title/Abstract] OR "yijinjing"[Title/Abstract] OR "liuzijue"[Title/Abstract] |
| 5 | "Motor Activity"[MeSH Terms] |
| 6 | "FMA"[Title/Abstract] OR "motor function"[Title/Abstract] OR "Movement"[Title/Abstract] OR "Gait"[Title/Abstract] OR "10MWT"[Title/Abstract] OR "Strength"[Title/Abstract] OR "Balance"[Title/Abstract] |
| 7 | "Randomized Controlled Trials as Topic"[MeSH Terms] |
| 8 | "controlled clinical trial"[Title/Abstract] OR "controlled trial"[Title/Abstract] OR "controlled study"[Title/Abstract] OR "controlled clinical study"[Title/Abstract] OR "RCT"[Title/Abstract] OR "randomiz*"[Title/Abstract] OR "placebo"[Title/Abstract] OR "randomly"[Title/Abstract] OR "trial"[Title/Abstract] OR "groups"[Title/Abstract] |
| 9 | "Stroke"[MeSH Terms] OR "Strokes"[Title/Abstract] OR "cerebrovascular accident"[Title/Abstract] OR "brain vascular accident"[Title/Abstract] |
| 10 | "Exercise"[MeSH Terms] OR "Exercises"[Title/Abstract] OR "physical activity"[Title/Abstract] OR "physical activities"[Title/Abstract] OR "fitness training"[Title/Abstract] OR "walking"[Title/Abstract] OR "Jogging"[Title/Abstract] OR "Bicycling"[Title/Abstract] OR "high intensity interval training"[Title/Abstract] OR "high intensity interval trainings"[Title/Abstract] OR "high intensity interval training"[Title/Abstract] OR "HIIT"[Title/Abstract] OR "Dancing"[Title/Abstract] OR "Resistance"[Title/Abstract] OR "Strength"[Title/Abstract] OR "aquatic therapy"[Title/Abstract] OR "aquatic exercise therapy"[Title/Abstract] OR "water exercise therapy"[Title/Abstract] OR "ai chi therapy"[Title/Abstract] OR "Swimming"[Title/Abstract] OR "mind body therapies"[Title/Abstract] OR "Yoga"[Title/Abstract] OR "pilates training"[Title/Abstract] OR "tai ji"[Title/Abstract] OR "Qigong"[Title/Abstract] OR "baduanjin"[Title/Abstract] OR "wuqinxi"[Title/Abstract] OR "yijinjing"[Title/Abstract] OR "liuzijue"[Title/Abstract] |
| 11 | "Motor Activity"[MeSH Terms] OR "FMA"[Title/Abstract] OR "motor function"[Title/Abstract] OR "Movement"[Title/Abstract] OR "Gait"[Title/Abstract] OR "10MWT"[Title/Abstract] OR "Strength"[Title/Abstract] OR "Balance"[Title/Abstract] |
| 12 | "Randomized Controlled Trials as Topic"[MeSH Terms] OR "controlled clinical trial"[Title/Abstract] OR "controlled trial"[Title/Abstract] OR "controlled study"[Title/Abstract] OR "controlled clinical study"[Title/Abstract] OR "RCT"[Title/Abstract] OR "randomiz*"[Title/Abstract] OR "placebo"[Title/Abstract] OR "randomly"[Title/Abstract] OR "trial"[Title/Abstract] OR "groups"[Title/Abstract] |
| 13 | ("Stroke"[MeSH Terms] OR ("Strokes"[Title/Abstract] OR "cerebrovascular accident"[Title/Abstract] OR "brain vascular accident"[Title/Abstract])) AND ("Exercise"[MeSH Terms] OR ("Exercises"[Title/Abstract] OR "physical activity"[Title/Abstract] OR "physical activities"[Title/Abstract] OR "fitness training"[Title/Abstract] OR "walking"[Title/Abstract] OR "Jogging"[Title/Abstract] OR "Bicycling"[Title/Abstract] OR "high intensity interval training"[Title/Abstract] OR "high intensity interval trainings"[Title/Abstract] OR "high intensity interval training"[Title/Abstract] OR "HIIT"[Title/Abstract] OR "Dancing"[Title/Abstract] OR "Resistance"[Title/Abstract] OR "Strength"[Title/Abstract] OR "aquatic therapy"[Title/Abstract] OR "aquatic exercise therapy"[Title/Abstract] OR "water exercise therapy"[Title/Abstract] OR "ai chi therapy"[Title/Abstract] OR "Swimming"[Title/Abstract] OR "mind body therapies"[Title/Abstract] OR ("Yoga"[Title/Abstract] OR "pilates training"[Title/Abstract]) OR "tai ji"[Title/Abstract] OR "Qigong"[Title/Abstract] OR "baduanjin"[Title/Abstract] OR "wuqinxi"[Title/Abstract] OR "yijinjing"[Title/Abstract] OR "liuzijue"[Title/Abstract])) AND ("Motor Activity"[MeSH Terms] OR ("FMA"[Title/Abstract] OR "motor function"[Title/Abstract] OR "Movement"[Title/Abstract] OR "Gait"[Title/Abstract] OR "10MWT"[Title/Abstract] OR "Strength"[Title/Abstract] OR "Balance"[Title/Abstract])) AND ("Randomized Controlled Trials as Topic"[MeSH Terms] OR ("controlled clinical trial"[Title/Abstract] OR "controlled trial"[Title/Abstract] OR "controlled study"[Title/Abstract] OR "controlled clinical study"[Title/Abstract] OR "RCT"[Title/Abstract] OR "randomiz*"[Title/Abstract] OR "placebo"[Title/Abstract] OR "randomly"[Title/Abstract] OR "trial"[Title/Abstract] OR "groups"[Title/Abstract])) |

**Table S2.2 Embase 13289**

| Search Number | Search Details |
| --- | --- |
| 1 | 'stroke'/exp OR 'strokes' OR 'cerebrovascular accident'/exp OR 'brain vascular accident' |
| 2 | 'exercise'/exp OR 'physical activity'/exp OR 'physical activities' OR 'fitness training' OR 'walking'/exp OR 'jogging'/exp OR 'bicycling'/exp OR 'high-intensity interval training' OR 'high-intensity interval trainings' OR 'high intensity interval training' OR 'hiit' OR 'dancing'/exp OR 'resistance training'/exp OR 'strength training' OR 'aquatic therapy' OR 'aquatic exercise therapy' OR 'water exercise therapy' OR 'ai chi therapy' OR 'swimming'/exp OR 'mind-body therapies' OR 'yoga'/exp OR 'pilates training' OR 'tai ji' OR 'qigong'/exp OR 'baduanjin' OR 'wuqinxi' OR 'yijinjing' OR 'liuzijue' |
| 3 | 'motor activity' OR 'fugl-meyer assessment' OR 'fma' OR 'motor function' OR 'movement'/exp OR 'gait'/exp OR '10mwt' OR 'strength'/exp OR 'balance'/exp |
| 4 | 'randomized controlled trial'/exp OR 'controlled clinical trial' OR 'controlled trial' OR 'controlled study' OR 'controlled clinical study' OR 'rct' OR randomiz* OR 'placebo'/exp OR 'randomly' OR 'trial'/exp OR 'groups' |
| 5 | 1 AND 2 AND 3 AND 4 |

**Table S2.3 Web of Science 9857**

| Search Details |
| --- |
| TS=("stroke" OR "strokes" OR "Cerebrovascular Accident" OR "Brain Vascular Accident")  AND  TS=("exercise" OR "exercises" OR "physical activity" OR "physical activities" OR "fitness training"  OR "walking" OR "jogging" OR "bicycling" OR "High-intensity interval training" OR "High-intensity interval trainings"  OR "high intensity interval training" OR "HIIT" OR "dancing" OR "resistance training" OR "strength training"  OR "Aquatic Therapy" OR "aquatic exercise therapy" OR "water exercise therapy" OR "Ai Chi Therapy"  OR "swimming" OR "Mind-Body Therapies" OR "Yoga or Pilates Training" OR "Tai Ji" OR "Qigong"  OR "baduanjin" OR "wuqinxi" OR "yijinjing" OR "liuzijue")  AND  TS=("Motor Activity" OR "FMA" OR "Fugl-Meyer Assessment" OR "Motor function" OR "movement"  OR "gait" OR "10MWT" OR "10-Meter Walk Test" OR "strength" OR "balance")  AND  TS=("randomized controlled trial" OR "controlled clinical trial" OR "controlled trial" OR "controlled study"  OR "controlled clinical study" OR "RCT" OR randomiz* OR "placebo" OR "randomly" OR "trial" OR "groups") |

**Table S2.4** **Scopus 14207**

| Search Number | Search Details |
| --- | --- |
| 1 | TITLE-ABS-KEY ( stroke OR strokes OR "Cerebrovascular Accident" OR "Brain Vascular Accident" ) |
| 2 | TITLE-ABS-KEY ( exercise OR exercises OR "Physical Activity" OR "Physical Activities" OR "fitness training" OR walking OR jogging OR bicycling OR "High-intensity interval training" OR "High-intensity interval trainings" OR "high intensity interval training" OR HIIT OR dancing OR resistance OR strength OR "Aquatic Therapy" OR "aquatic exercise therapy" OR "water exercise therapy" OR "Ai Chi Therapy" OR swimming OR "Mind-Body Therapies" OR "Yoga or Pilates Training" OR "Tai Ji" OR Qigong OR baduanjin OR wuqinxi OR yijinjing OR liuzijue ) |
| 3 | TITLE-ABS-KEY ( "Motor Activity" OR FMA OR "Motor function" OR movement OR gait OR "10MWT" OR strength OR balance ) |
| 4 | TITLE-ABS-KEY ( "randomized controlled trial" OR "controlled clinical trial" OR "controlled trial" OR "controlled study" OR "controlled clinical study" OR RCT OR randomiz* OR placebo OR randomly OR trial OR groups ) |
| 5 | 1 AND 2 AND 3 AND 4 |

**Table S2.5 Cochrane Library 5658**

| Search Number | Search Details |
| --- | --- |
| 1 | stroke OR strokes OR "Cerebrovascular Accident" OR "Brain Vascular Accident" |
| 2 | exercise OR exercises OR "physical activity" OR "physical activities" OR "fitness training" OR walking OR jogging OR bicycling OR "High-intensity interval training" OR "High-intensity interval trainings" OR "high intensity interval training" OR HIIT OR dancing OR "resistance training" OR "strength training" OR "Aquatic Therapy" OR "aquatic exercise therapy" OR "water exercise therapy" OR "Ai Chi Therapy" OR swimming OR "Mind-Body Therapies" OR "Yoga or Pilates Training" OR "Tai Ji" OR Qigong OR baduanjin OR wuqinxi OR yijinjing OR liuzijue |
| 3 | "Motor Activity" OR FMA OR "Fugl-Meyer Assessment" OR "Motor function" OR movement OR gait OR 10MWT OR "10-Meter Walk Test" OR strength OR balance |
| 4 | "randomized controlled trial" OR "controlled clinical trial" OR "controlled trial" OR "controlled study" OR "controlled clinical study" OR RCT OR randomiz* OR placebo OR randomly OR trial OR groups |
| 5 | 1 AND 2 AND 3 AND 4 |

**Supplementary 3 — Excluded Studies List**

**Table S 3 Excluded literature**

| NO. | Author (Year) | Title | Reason for Exclusion |
| --- | --- | --- | --- |
| 7 | T. George Hornby 2024^23^ | Acute Intermittent Hypoxia With High-Intensity Gait Training in Chronic Stroke: A Phase II Randomized Crossover Trial | Interventions could not be classified |
| 15 | Qingfang Zhang 2023^24^ | Ankle rehabilitation robot training for stroke patients with foot drop: Optimizing intensity and frequency | Interventions could not be classified |
| 23 | Christine T. Shiner, 2014^25^ | Bilateral priming before wii-based movement therapy enhances upper limb rehabilitation and its retention after stroke: a case-controlled study | Interventions could not be classified |
| 27 | Stephanie A. Combs-Miller, 2014^26^ | Body weight-supported treadmill training vs. overground walking training for persons with chronic stroke: a pilot randomized controlled trial | Interventions could not be classified |
| 28 | Abhishek Srivastava, 2016^27^ | Bodyweight-supported treadmill training for retraining gait among chronic stroke survivors: A randomized controlled study | Interventions could not be classified |
| 29 | Yunhwan Kim, 2023^28^ | Bolstering Cognitive and Locomotor Function in Post-Stroke Dementia Using Human-Robotic Interactive Gait Training | The statistical methods mentioned in the literature are all mean and standard deviation. However, in fact, the only outcome indicator I need is the mean. It is impossible to synthesize the data. Although graphs are provided, the mean and standard deviation can also be calculated by data graphs. However, in order to reduce experimental errors, it is excluded. |
| 33 | Oluwole O. Awosika, 2024^29^ | Characterizing the Longitudinal Impact of Backward Locomotor Treadmill Training on Walking and Balance Outcomes in Chronic Stroke Survivors: A Randomized Single Center Clinical Trial | This is a preprint. |
| 34 | Chia-Lun Liu, 2024^30^ | Characterizing Two Hybrid Exercise-Cognitive Training Interventions With Neurophysiological and Behavioral Indexes in Post-Stroke Patients With Cognitive Dysfunction: A Randomized Controlled Trial | Although graphs are provided, the average and standard deviation can also be calculated by data graphs, but in order to reduce experimental errors, we exclude |
| 36 | Litong Wang, 2024^31^ | Clinical Effect Analysis of Wearable Sensor Technology-Based Gait Function Analysis in Post-Transcranial Magnetic Stimulation Stroke Patients | Although graphs are provided, the average and standard deviation can also be calculated by data graphs, but in order to reduce experimental errors, we exclude |
| 39 | Sehrish Naureen，2025^32^ | COMBINED EFFECTS OF AUDITORY AND VISUAL RHYTHMICAL CUEING ON LOWER LIMB SENSORIMOTOR RECOVERY AND GAIT PARAMETERS IN PATIENTS WITH HEMIPLEGIA | Interventions could not be classified |
| 41 | J. Merkert, 2011^33^ | Combined whole body vibration and balance training using Vibrosphere®: improvement of trunk stability, muscle tone, and postural control in stroke patients during early geriatric rehabilitation | Data not available |
| 47 | Qurat Ul Ain, 2022^34^ | Comparison Between Effects of Functional Training Program and Conventional Therapy on Postural Control and Functional Mobility in Chronic Stroke | Data not available |
| 48 | Jin Park, 2015^35^ | Comparison between treadmill training with rhythmic auditory stimulation and ground walking with rhythmic auditory stimulation on gait ability in chronic stroke patients: A pilot study | The data are only for inter-group analysis and lack baseline data. |
| 51 | Kristina Traxler, 2023^36^ | Combining specific task-oriented training with manual therapy to improve balance and mobility in patients after stroke: a mixed methods pilot randomised controlled trial | Data not available |
| 52 | Pınar Özge Başaran, 2025^37^ | Comparison of low-level laser therapy versus neuromuscular electrical nerve stimulation at hemiplegic shoulder pain and upper extremity functions | Data not available |
| 55 | In-mo Park, 2013^38^ | A Comparison of the Effects of Overground Gait Training and Treadmill Gait Training According to Stroke Patients’ Gait Velocity | Interventions could not be classified |
| 56 | Çağrı Alıpsatıcı, 2020^39^ | Comparison of the Effects of Treadmill Trainings on Walking and Balance Functions by Increasing the Speed and Incline in Chronic Patients with Stroke | Interventions could not be classified |
| 58 | Carolynn Patten, 2013^40^ | Concurrent neuromechanical and functional gains following upper-extremity power training post-stroke | Data cannot be converted |
| 60 | J. Podubecka, 2011^41^ | Cyclic movement training versus conventional physiotherapy for rehabilitation of hemiparetic gait after stroke: a pilot study | Non-English literature |
| 61 | Ilanit Evron, 2024^42^ | A digital exercise and augmented reality training system improved mobility among stroke patients: A randomized control trial | No data |
| 63 | In-Hee Lee, 2015^43^ | Does the speed of the treadmill influence the training effect in people learning to walk after stroke? A double-blind randomized controlled trial | Interventions could not be classified |
| 70 | Clark & Patten, 2012^44^ | Eccentric versus concentric resistance training to enhance neuromuscular activation and walking speed following stroke | Interventions could not be classified |
| 72 | Palimeris et al., 2022^45^ | Effect of a tailored upper extremity strength training intervention combined with direct current stimulation in chronic stroke survivors: A Randomized Controlled Trial | Although graphs are provided, the average and standard deviation can also be calculated by data graphs, but in order to reduce experimental errors, we exclude |
| 78 | Jong-Bae Choi, 2022^46^ | The Effect of Action Observation Combined with Motor Imagery Training on Upper Extremity Function and Corticospinal Excitability in Stroke Patients: A Randomized Controlled Trial | Interventions could not be classified |
| 79 | Stefano Brunelli, 2019^47^ | Early body weight-supported overground walking training in patients with stroke in subacute phase compared to conventional physiotherapy: a randomized controlled pilot study | No data |
| 84 | Kanase, 2022^48^ | Effect of Aquatic Therapy on Functional Mobility and Balance in Chronic Stroke | Data not available |
| 89 | Hye Joo Jeon, 2018^49^ | Effect of bilateral lower limb strengthening exercise on balance and walking in hemiparetic patients after stroke: a randomized controlled trial | Interventions could not be classified |
| 92 | Liying Zhang, 2024^50^ | The effect of body weight-supported Tai Chi Yunshou on upper limb motor function in stroke survivors based on neurobiomechanical analysis: a four-arm, parallel-group, assessors-blind randomized controlled trial protocol | Experimental protocol |
| 98 | Shin Jun Park, 2020^51^ | Addition of proprioceptive neuromuscular facilitation to cardiorespiratory training in patients poststroke: study protocol for a randomized controlled trial | Interventions could not be classified |
| 99 | Gui bin Song, 2015^52^ | Effect of dual tasks on balance ability in stroke patients | Interventions could not be classified |
| 100 | Gye Yeop Kim, 2014^53^ | Effect of Dual-task Rehabilitative Training on Cognitive and Motor Function of Stroke Patients | Interventions could not be classified |
| 102 | Elisabeth Rydwik, 2006^54^ | The effect of exercise of the affected foot in stroke patients--a randomized controlled pilot trial | Data not available |
| 105 | Stéphane Mandigout, 2021^55^ | Effect of individualized coaching at home on walking capacity in subacute stroke patients: A randomized controlled trial (Ticaa'dom) | Although graphs are provided, the average and standard deviation can also be calculated by data graphs, but in order to reduce experimental errors, we exclude |
| 111 | Hyuk-Shin Cho, 2015^56^ | Effect of mirror therapy with tDCS on functional recovery of the upper extremity of stroke patients | Interventions could not be classified |
| 122 | Wing-Nga Chan, 2017^57^ | Effect of Tai Chi Training on Dual-Tasking Performance That Involves Stepping Down among Stroke Survivors: A Pilot Study | Data not available |
| 124 | Yinghua Li, 2024^58^ | Effect of task-oriented training assisted by force feedback hand rehabilitation robot on finger grasping function in stroke patients with hemiplegia: a randomised controlled trial | Outcome measure not available |
| 125 | Hui Dang, 2019^59^ | Effect of task-oriented training combined with vibration therapy on upper limb function in patients with hemiplegia after stroke | Data not available |
| 132 | Junsang Yoo, 2014^60^ | The effect of trunk stabilization exercise using an unstable surface on the abdominal muscle structure and balance of stroke patients | Interventions could not be classified |
| 137 | Ho-Jung An, 2014^61^ | The effect of various dual task training methods with gait on the balance and gait of patients with chronic stroke | Interventions could not be classified |
| 153 | Jinhong Kim, 2024^62^ | Effectiveness of mid thoracic spine mobilization on postural balance and gait ability in subacute stroke patients: A randomized clinical trial | Interventions could not be classified |
| 163 | József Tollár, 2023^63^ | Effects of 2-Year-Long Maintenance Training and Detraining on 558 Subacute Ischemic Stroke Patients' Clinical-Motor Symptoms | Although graphs are provided, the average and standard deviation can also be calculated by data graphs, but in order to reduce experimental errors, we exclude |
| 166 | Park Donghwan, 2018^64^ | Effects of a 4-Week Self-Ankle Mobilization with Movement Intervention on Ankle Passive Range of Motion, Balance, Gait, and Activities of Daily Living in Patients with Chronic Stroke: A Randomized Controlled Study | Interventions could not be classified |
| 168 | Torunn Askim, 2010^65^ | Effects of a community-based intensive motor training program combined with early supported discharge after treatment in a comprehensive stroke unit: a randomized, controlled trial | Data not available |
| 174 | Caroline I.E. Renner, 2020​^66^ | Bilateral Arm Training vs Unilateral Arm Training for Severely Affected Patients With Stroke: Exploratory Single-Blinded Randomized Controlled Trial | Interventions could not be classified |
| 184 | Douglas Rafael da Rosa Pinheiro, 2021^67^ | Effects of aerobic cycling training on mobility and functionality of acute stroke subjects: A randomized clinical trial | The 10mwt control group showed no change |
| 194 | Jin-Gang Her, 2011^68^ | Effects of balance training with various dual-task conditions on stroke patients | Interventions could not be classified |
| 195 | Sunhwa Shim, 2015^69^ | Effects of bilateral training on motor function, amount of activity and activity intensity measured with an accelerometer of patients with stroke | Data not available |
| 200 | Rüstem Mustafaoğlu, 2018^70^ | The effects of body weight-supported treadmill training on static and dynamic balance in stroke patients: A pilot, single-blind, randomized trial | Data not available |
| 201 | Shangrong Jiang, 2023^71^ | Effects of central intermittent theta-burst stimulation combined with repetitive peripheral magnetic stimulation on upper limb function in stroke patients | Data not available |
| 208 | Keh-chung Lin, 2009^72^ | Effects of constraint-induced therapy versus bilateral arm training on motor performance, daily functions, and quality of life in stroke survivors | Interventions could not be classified |
| 213 | Byoung-Sun Park, 2015^73^ | Effects of conventional overground gait training and a gait trainer with partial body weight support on spatiotemporal gait parameters of patients after stroke | Interventions could not be classified |
| 214 | Eun-Jung Chung, 2013^74^ | The effects of core stabilization exercise on dynamic balance and gait function in stroke patients | Data not available |
| 218 | Ji yeun Lee，2011^75^ | The effects of exercising on unstable surfaces on the balance ability of stroke patients | Interventions could not be classified |
| 222 | Gabriela L. Gama，2023^76^ | Effects of gait training with body weight support on a treadmill versus overground in individuals with stroke | Interventions could not be classified |
| 223 | Kyung-Pil Na，2015^77^ | Effects of gait training with horizontal impeding force on gait and balance of stroke patients | Interventions could not be classified |
| 224 | Zhe Cui，2024^78^ | The effects of gaze stability exercises on balance, gait ability, and fall efficacy in patients with chronic stroke: A 2-week follow-up from a randomized controlled trial | Interventions could not be classified |
| 240 | Chao Han，2012^79^ | Effects of intensity of arm training on hemiplegic upper extremity motor recovery in stroke patients: a randomized controlled trial | Interventions could not be classified |
| 242 | Sinikka H. Peurala，2009^80^ | Effects of intensive therapy using gait trainer or floor walking exercises early after stroke | Interventions could not be classified |
| 244 | Chang Sook Kim，2011^81^ | The effects of lower extremitiy muscle strengthening exercise and treadmill walking exercise on the gait and balance of stroke patients | Interventions could not be classified |
| 245 | Nicola Lamberti，2017^82^ | Effects of low-intensity endurance and resistance training on mobility in chronic stroke survivors: a pilot randomized controlled study | Interventions could not be classified |
| 260 | Kyoung-Sim Jung，2022^83^ | Effects of Pelvic Stabilization Training with Lateral and Posterior Tilt Taping on Pelvic Inclination, Muscle Strength, and Gait Function in Patients with Stroke: A Randomized Controlled Study | Interventions could not be classified |
| 262 | Li Bian，2024^84^ | Effects of Priming Intermittent Theta Burst Stimulation With High-Definition tDCS on Upper Limb Function in Hemiparetic Patients With Stroke: A Randomized Controlled Study | Interventions could not be classified |
| 264 | Kyo Chul Seo，2015^85^ | The effects of ramp gait exercise with PNF on stroke patients' dynamic balance | Interventions could not be classified |
| 273 | Byoung-Ha Hwang，2019^86^ | The effects of sand surface training on changes in the muscle activity of the paretic side lower limb and the improvement of dynamic stability and gait endurance in stroke patients | Interventions could not be classified |
| 276 | Özge Onursal Kilinç，2024^87^ | Effects of scapulo-humeral training on ultrasonographic and clinical evaluations in stroke: a randomized controlled trial | Interventions could not be classified |
| 283 | Ishtiaq Ahmed，2023^88^ | The effects of low-intensity resistance training with blood flow restriction versus traditional resistance exercise on lower extremity muscle strength and motor functionin ischemic stroke survivors: a randomized controlled trial | Interventions could not be classified |
| 284 | Katherine J. Sullivan，2007^89^ | Effects of task-specific locomotor and strength training in adults who were ambulatory after stroke: results of the STEPS randomized clinical trial | Interventions could not be classified |
| 290 | Mian Ali Raza，2021^90^ | Effects of treadmill training and stationary cycling training to improve ambulatory function and cardiovascular fitness in hemiparetic stroke patients | Data not available |
| 294 | Pei-Yun Lee，2020^91^ | Effects of Trunk Exercise on Unstable Surfaces in Persons with Stroke: A Randomized Controlled Trial | Data not available |
| 300 | Umair Ahmed 2019^92^ | Effects of unstable and stable trunk exercise programs on trunk motor performance, balance and functional mobility in stroke patients | Interventions could not be classified |
| 301 | Kerim Kerimov 2021^93^ | The Effects of Upper Extremity Isokinetic Strengthening in Post-Stroke Hemiplegia: A Randomized Controlled Trial | Interventions could not be classified |
| 310 | Giovanni Morone 2014^94^ | The efficacy of balance training with video game-based therapy in subacute stroke patients: a randomized controlled trial | Although graphs are provided, the average and standard deviation can also be calculated by data graphs, but in order to reduce experimental errors, we exclude |
| 312 | Myeong Sun Kim 2025^95^ | Efficacy of brain-computer interface training with motor imagery-contingent feedback in improving upper limb function and neuroplasticity among persons with chronic stroke: a double-blinded, parallel-group, randomized controlled trial | Interventions could not be classified |
| 316 | Ashish Bisla 2022^96^ | EFFICACY OF MULTIDIRECTIONAL STEPPING TRAINING AND PERTURBATION TRAINING ON BALANCE IN CHRONIC STROKE PATIENTS | Although graphs are provided, the average and standard deviation can also be calculated by data graphs, but in order to reduce experimental errors, we exclude |
| 335 | Kristen L. Hollands 2015^97^ | Feasibility and Preliminary Efficacy of Visual Cue Training to Improve Adaptability of Walking after Stroke: Multi-Centre, Single-Blind Randomised Control Pilot Trial | Interventions could not be classified |
| 344 | Donghwan Park 2019^98^ | Four-week training involving ankle mobilization with movement versus static muscle stretching in patients with chronic stroke: a randomized controlled trial | Interventions could not be classified |
| 346 | Susan Högg 2020^99^ | High-intensity arm resistance training does not lead to better outcomes than low-intensity resistance training in patients after subacute stroke: A randomized controlled trial | Interventions could not be classified |
| 347 | Pierce Boyne 2016^100^ | High-Intensity Interval Training and Moderate-Intensity Continuous Training in Ambulatory Chronic Stroke: Feasibility Study | Interventions could not be classified |
| 350 | Chen Kangcheng 2024^101^ | Impact of diverse aerobic exercise plans on glycemic control, lipid levels, and functional activity in stroke patients with type 2 diabetes mellitus | Data not available |
| 361 | Gerard Fluet，2024^102^ | The influence of scaffolding on intrinsic motivation and autonomous adherence to a game-based, sparsely supervised home rehabilitation program for people with upper extremity hemiparesis due to stroke. A randomized controlled trial | Data not available |
| 362 | Ling-Yi Liao，2024^103^ | Intermittent Theta-Burst Stimulation for Stroke: Primary Motor Cortex Versus Cerebellar Stimulation: A Randomized Sham-Controlled Trial | Although graphs are provided, the average and standard deviation can also be calculated by data graphs, but in order to reduce experimental errors, we exclude |
| 365 | Kiersten M. McCartney，2024^104^ | Matching Clinical Profiles with Interventions to Optimize Daily Stepping in People with Stroke | This is a preprint |
| 366 | Ning Wei，2022^105^ | Optimal frequency of whole body vibration training for improving balance and physical performance in the older people with chronic stroke: A randomized controlled trial | Interventions could not be classified |
| 368 | Erin C. King，2024^106^ | Priming and task-specific training for arm weakness post stroke: A randomized controlled trial | Although graphs are provided, the average and standard deviation can also be calculated by data graphs, but in order to reduce experimental errors, we exclude |
| 373 | Zhuo Li，2024^107^ | Rehabilitation effect of core muscle training combined with functional electrical stimulation on lower limb motor and balance functions in stroke patients | Although graphs are provided, the average and standard deviation can also be calculated by data graphs, but in order to reduce experimental errors, we exclude |
| 377 | Sun Young Jeon，2024^108^ | Resistive versus active assisted robotic training for the upper limb after a stroke: A randomized controlled study | Interventions could not be classified |
| 378 | San San Tay，2024^109^ | Robot-mediated impairment-oriented and task-specific training on upper limb post stroke: feasibility and preliminary effects on physical function and quality of life | Interventions could not be classified |
| 380 | Rocco Salvatore Calabrò，2018^110^ | Shaping neuroplasticity by using powered exoskeletons in patients with stroke: a randomized clinical trial | Although graphs are provided, the average and standard deviation can also be calculated by data graphs, but in order to reduce experimental errors, we exclude |
| 381 | Eunmi Kim，2024^111^ | Simultaneous high-definition transcranial direct current stimulation and robot-assisted gait training in stroke patients | Data not available |
| 382 | Piyashi Biswas，2024^112^ | A single-center, assessor-blinded, randomized controlled clinical trial to test the safety and efficacy of a novel brain-computer interface controlled functional electrical stimulation (BCI-FES) intervention for gait rehabilitation in the chronic stroke population | Data not available |
| 385 | Jie Zhao，2022^113^ | Tailored Sitting Tai Chi Program for Subacute Stroke Survivors: A Randomized Controlled Trial | Data not available |
| 386 | Xiang-Qian Shi，2024^114^ | Task-Oriented Training by a Personalized Electromyography-Driven Soft Robotic Hand in Chronic Stroke: A Randomized Controlled Trial | Interventions could not be classified |
| 387 | Seunghoon Jeong，2023^115^ | Task-Oriented Training with Abdominal Drawing-in Maneuver in Sitting Position for Trunk Control, Balance, and Activities of Daily Living in Patients with Stroke: A Pilot Randomized Controlled Trial | Interventions could not be classified |
| 390 | Wonjae Choi，2017^116^ | Whole-Body Vibration Combined with Treadmill Training Improves Walking Performance in Post-Stroke Patients: A Randomized Controlled Trial | Although graphs are provided, the average and standard deviation can also be calculated by data graphs, but in order to reduce experimental errors, we exclude |
| 395 | Prudence Plummer，2021^117^ | Cognitive-motor dual-task gait training within 3 years after stroke: A randomized controlled trial | Interventions could not be classified |
| 398 | Hajime Yagura, 2006^118^ | Does therapeutic facilitation add to locomotor outcome of body weight--supported treadmill training in nonambulatory patients with stroke? A randomized controlled trial | Although graphs are provided, the average and standard deviation can also be calculated by data graphs, but in order to reduce experimental errors, we exclude |
| 399 | Wen-Xiu Wu, 2020^119^ | Effect of Early and Intensive Rehabilitation after Ischemic Stroke on Functional Recovery of the Lower Limbs: A Pilot, Randomized Trial | Although graphs are provided, the average and standard deviation can also be calculated by data graphs, but in order to reduce experimental errors, we exclude |
| 403 | Kelvin Chan, 2016^120^ | The effect of water-based exercises on balance in persons post-stroke: a randomized controlled trial | Although graphs are provided, the average and standard deviation can also be calculated by data graphs, but in order to reduce experimental errors, we exclude |
| 404 | Kwang-Yong Choi, 2021^121^ | Effect of Whole-body Vibration Combined with Task Oriented Training on Muscle Strength, Balance, and Gait in Stroke Patients with Hemiplegia: A Pilot Randomized Controlled Trial | Interventions could not be classified |
| 408 | Saniye Aydoğan Arslan, 2021^122^ | Effects of Inspiratory Muscle Training on Respiratory Muscle Strength, Trunk Control, Balance and Functional Capacity in Stroke Patients: A single-blinded randomized controlled study | Interventions could not be classified |
| 409 | Jing Liu，2020^123^ | Effects of Sling Exercise Therapy on balance, mobility, activities of daily living, quality of life and shoulder pain in stroke patients：a randomized controlled trial | Interventions could not be classified |
| 410 | Guohua Zheng，2019^124^ | Effects of Tai Chi on Cerebral Hemodynamics and Health-Related Outcomes in Older Community Adults at Risk of Ischemic Stroke: A Randomized Controlled Trial | Interventions could not be classified |
| 416 | Tae-Woo Kang，2015^125^ | Six-Week Nordic Treadmill Training Compared with Treadmill Training on Balance, Gait, and Activities of Daily Living for Stroke Patients: A Randomized Controlled Trial | Although graphs are provided, the average and standard deviation can also be calculated by data graphs, but in order to reduce experimental errors, we exclude |

**Supplementary 4 — Conversion Formulas for Effect Size and SD Estimation**

To ensure the consistency and comparability of quantitative data across studies, the following calculation formulas were used to standardize outcome measures and derive effect sizes:

1.MET-min/week

MET-min/week=MET value×minutes/session×sessions/week

MET value refers to the metabolic equivalent assigned to each exercise modality;

minutes per session is the duration of each exercise session;

Sessions per week is the frequency of exercise per week.

2. Data Transformation Methods for 10-Meter Walk Test (10MWT)

To ensure consistency across studies reporting 10MWT outcomes in different formats, all data were standardized to walking speed (meters per second, m/s) prior to meta-analysis. The following transformation methods were applied:

2.1 For studies reporting walking time (seconds) to complete 10 meters:

The walking speed (m/s) was calculated using:

$$M_{\mathrm{speed}}=\frac{10}{M_{\mathrm{time}}}$$

The corresponding standard deviation (SD) of walking speed was calculated using error propagation:

$$\mathrm{SD}_{\mathrm{speed}}=\frac{10\times\mathrm{SD}_{\mathrm{time}}}{\mathrm{time}^{2}}$$

**2.2 For studies reporting walking speed in meters per minute (m/min):**
The values were converted to m/s by dividing both the mean and SD by 60.

**2.3 For studies directly reporting walking speed in m/s:**
No transformation was required.

All 10MWT outcomes were thus expressed in m/s for uniform synthesis in the quantitative analysis.

1. Estimating the Standard Error (SE) from a 95% Confidence Interval

$$SE=\frac{Upper limit-Lower limit}{2\times1.96}$$

Used to estimate SE when only 95% CI is available.

Calculation of Mean Change

The change in mean value from baseline to post-intervention was calculated using the formula:

$$\Delta Mean=Post Mean-Pre Mean$$

In this context, ΔMean refers to the difference between post-intervention and baseline values within the same group.

1. Calculation of the Standard Deviation of Change Scores
   If the standard deviation (SD) of change is not directly reported, it can be estimated from baseline and post-intervention SDs using the following formula:

$$\mathrm{SD}_{\mathrm{change}}=\sqrt{\mathrm{SD}_{\mathrm{pre}}^{2}+\mathrm{SD}_{\mathrm{post}}^{2}-2\cdot r\cdot\mathrm{SD}_{\mathrm{pre}}\cdot\mathrm{SD}_{\mathrm{post}}}$$

In this study, we assumed r=0.5.

5.Calculating Treatment Effect (TE) and Standard Error (SE) for Netmeta

a. Treatment Effect (TE):

$$TE=\overline{X}_{\mathrm{intervention}}-\overline{X}_{\mathrm{control}}$$

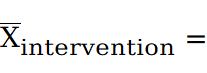
 Mean change in the intervention group;
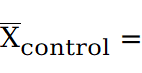
 Mean change in the control group

b. Standard Error (SE) of the Treatment Effect:

$$SE=\sqrt{\frac{\mathrm{SD}_{\mathrm{intervention}}^{2}}{n_{\mathrm{intervention}}}+\frac{\mathrm{SD}_{\mathrm{control}}^{2}}{n_{\mathrm{control}}}}$$

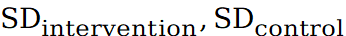
:Standard deviations of the change scores; n:samples

**Supplementary 5 — Study Characteristics of Included Studies**

**Table S5 Study Characteristics of Included Studies**

| **ID** | **Author and Year** | **Income Status** | **Sample Size** | **Injury Location** | **Type** | **Stage** | **Severity** | **Age** | **Gender** | **CR(Y/N)** | **Category** | **Diagnostic Criteria** | **Intervention** | **Frequency** | **Duration** | **METs**  **Calculation** | **Outcome** |
| --- | --- | --- | --- | --- | --- | --- | --- | --- | --- | --- | --- | --- | --- | --- | --- | --- | --- |
| 1 | Rebecca Lewthwaite 2018^126^ | High-income | 103 | Upper limb function | I | Subacute Phase | Mild to moderate | 61.8±8.4 | 67M/52F | Y | TOT | FMA-UE: 19–58 WHO criteria for stroke; onset within 1–6 months; unilateral upper-limb deficit; cognitively able to follow commands. | Accelerated Skill Acquisition Program (ASAP): Task-based, personalized training program with motivational enhancement and challenging practice. | ~1.875 sessions/week, 60 min per session | 16weeks | 120 min × 3.0 METs = 360 METs-min/week | FMA-UE |
|  |  | High-income | 99 | Upper limb function | I | Subacute Phase | Mild to moderate | 60.2±7 | 67M/53F | Y | FT |  | Dose-Equivalent Usual Care Control (DEUCC): Standard occupational therapy with same dose as ASAP, without personalization or motivational elements. | ~1.875 sessions/week, 60 min per session | 16weeks | 120 min × 3.0 METs = 360 METs-min/week |  |
|  |  | High-income | 93 | Upper limb function | I | Subacute Phase | Mild to moderate | 62.1±8.6 | 68M/54F | Y | RC |  | Usual Care Control (UCC): Standard rehabilitation with variable frequency and content, average occupational therapy time 42 min/week. | Variable (average 42 min/week) | 16weeks | 42 min × 3.0 METs = 126 METs-min/week |  |
| 2 | Mauro Mancuso 2021^127^ | High-income | 16 | Upper limb function | I | Acute Phase | Moderate to severe | 64.5±15.7 | 10M/6F | Y | MBE | FMA-UE 19–58; CT; Token >8; Star <51; MMSE <23.8; NIHSS eye+. | Action Observation Training (AOT): Watch 3-min video + imitate for 2 min, 3 cycles/day; 1 task/day (total 20 tasks). Combined with conventional rehabilitation (physical therapy, occupational therapy, functional training). | 5sessions/week; 30 min AOT + 60 min CR per session | 4weeks | AOT: 150 min × 3.0 = 450 CR: 300 min × 3.0 = 900 Total = 1350 METs-min/week | FMA-UE |
|  |  | High-income | 16 | Upper limb function | I | Acute Phase | Moderate to severe | 76.5±13.7 | 10M/6F | Y | TOT |  | Task-Oriented Training (TOT): Perform functional upper-limb tasks directly without video; therapist provides verbal guidance without demonstration. Combined with conventional rehabilitation (same as above). | 5sessions/week; 30 min AOT + 60 min CR per session | 4weeks | TOT: 150 min × 3.0 = 450 CR: 300 min × 3.0 = 900 Total = 1350 METs-min/week |  |
| 3 | Hyun-Ju Park 2017^128^ | High-income | 12 | Whole-body function | 6I + 6H | Chronic Phase | Mild to moderate | 57.33±6.89 | 9M/3F | Y | MBE | CT confirmed; walk ≥10m (±aid); MMSE >24. | Action Observation of Community Walking: Participants watched instructional videos demonstrating four-phase walking strategies in community settings (e.g., flat surface, uneven terrain, unpredictable areas, parking lots/shopping centers). Videos included Nrmal and 0.5× speed with verbal guidance. Feedback provided by physiotherapists after viewing. | Video: 30 min × 3/week CR: 30 min × 5/week | 4weeks | Video: 90 min × 1.3 METs = 117 CR: 150 min × 3.0 = 450 Total = 567 METs-min/week | 10MWT |
|  |  | High-income | 13 | Whole-body function | 5I + 8H | Chronic Phase | Mild to moderate | 55.08±8.12 | 7M/6F | Y | NE |  | Static Scenery Viewing: Participants viewed static landscape images (e.g., coastlines, mountains, rivers, countryside) with N motor-related content. Combined with same conventional rehabilitation. | Video: 30 min × 3/week CR: 30 min × 5/week | 4weeks | Video: 90 min × 1.3 METs = 117 METs-min CR: 150 min × 3.0 METs = 450 METs-min Total = 567 METs-min/week |  |
| 4 | Akemi Hioka 2020^129^ | High-income | 10 | Lower limb function | 5H + 3I | Subacute Phase | Moderate | 60.9±5.8 | 37.5%M/62.5%F | Y | MBE | DiagNsis: CT or MRI confirmed stroke Inclusion: BRS ≥ 2; MMSE ≥ 24 | Action Observation Training (AOT) for Walking: Patients watched 20-minute videos daily showing healthy adults walking from different angles (front, side, back). After viewing, they imitated the walking actions for 10 minutes under physiotherapist supervision, including gait, trunk movement, weight shifting, stepping, and stair climbing. | 30min/session (20 obs + 10 imit), 5×/week, 12 weeks. | 3months | Observation: 1.3 × (20/60) × 5 = 130 METs-min/weekImitation: 3.5 × (10/60) × 5 = 175 METs-min/week AOT subtotal = 305 METs-min/week Conventional rehab: 180 min/day × 7 days = 21 hr/week × 3.0 METs = 3780 METs-min/weekTotal dose = 4085 METs-min/week | 10MWT |
|  |  | High-income | 10 | Lower limb function | 8H | Subacute Phase | Moderate | 54.1±9.6 | 87.5%M/12.5%F | Y | NE |  | Blank Control Group: Received conventional rehabilitation only, without any structured motor or cognitive training intervention. | 180minutes/day,7days/week |  | 180 min/day × 7 = 21 hr/week 3.0 METs × 21 = 3780 METs-min/week |  |
| 5 | Ting-ting Yeh 2019^130^ | Middle- and high-income | 15 | Whole-body function | Mixed | Chronic Phase | Mild to moderate | 50.63±3.99 | 8M/7F | N | MBE | DiagNstic & Inclusion: Stroke confirmed; MMSE ≤19; MoCA <26; CDR ≥0.5; N recent MI, PD, MS, or ALS | Intervention: 30-min aerobic cycling (40–70% max HR) + 30-min computer-based cognitive training (BrainHQ) | 2.5×/week, 60 min (30+30), total 36 sessions. | 12-18  weeks | Cycling: 75 min × 5.5 METs = 412.5 METs-min/week Cognitive: 75 min × 1.8 METs = 135 METs-min/week Total = 547.5 METs-min/week | 6MWT |
|  |  | Middle- and high-income | 15 | Whole-body function | Mixed | Chronic Phase | Mild to moderate | 60.21±3.10 | 13M/2F | N | RC |  | Intervention: 30-min Nn-aerobic physical training + 30-min unstructured cognitive activity (e.g., newspaper, video) | 2.5×/week, 60 min (30+30), total 36 sessions. | 12-18  weeks | Physical: 75 min × 2.5 METs = 187.5 METs-min/week Cognitive: 75 min × 1.3 METs = 97.5 METs-min/week Total = 285 METs-min/week |  |
| 6 | Stacy L. Fritz 2013^131^ | High-income | 15 | Lower limb & trunk balance dysf | Mixed | Chronic Phase | Mild to moderate | 67.6±9.3 | Nt reported | N | VRG | Post-stroke >6 months; can stand ≥5 min and walk 3 m (with/without aid) | Nintendo Wii/PS2 EyeToy active gaming (dynamic balance, stepping, weight shifting, speed) | 4×/week, 55 min, 5 weeks. | 5weeks | 27.5 min × 3.5 + 27.5 min × 2.0 = 2.52 METs·h 2.52 × 4 = 604.8 METs-min/week | 6MWT  BBS  FMA |
|  |  | High-income | 13 | Lower limb & trunk balance dysf | Mixed | Chronic Phase | Mild to moderate | 64.5±10.1 | Nt reported | N | NE |  | N structured intervention; maintained daily activities | None | 5weeks | 0 METs-min/week |  |
| 8 | Zun Wang 2014^132^ | Middle- and high-income | 24 | Lower limb function | 13I + 11H | Subacute Phase | Severe | 57.0±6.8 | M18/F6 | Y | AE | 2–6weeks post-stroke; Chedoke-McMaster ≤3; age 45–75; cardiovascular stable | 30-min low-intensity cycling + 2 hrs conventional rehab, 5×/week for 6weeks | 3×/week cycling; rehab 195 min/day, 5 days/week | 6weeks | Cycling: 3.0 × 0.5 h × 3 = 270 METs-min Rehab: 1470 min × 2.5 METs = 3675 METs-min Total = 3945 METs-min/week | FMA |
|  |  | Middle- and high-income | 24 | Lower limb function | 12I + 12H | Subacute Phase | Severe | 55.0±11.5 | M17/F7 | Y | NE |  | Conventional rehab only (N cycling intervention) | 195 min/day, 5 days/week | 6weeks | 1050 min × 2.5 METs = 2625 METs-min/week |  |
| 9 | Qurat Ul Ain 2021^133^ | Middle-income | 25 | Upper limb function | Mixed | Chronic Phase | Mild to moderate | 57.48±10.60 | M23/F2 | Y | VRG | First-ever or ≥6-month post-stroke; Age 40–70; MAS ≥ 4 | Xbox Kinect training (15–20 min) + 20 min conventional upper limb rehab | 5×/week, 35–40 min/session, 6weeks | 6weeks | Kinect: 3.0 × 0.33 × 5 = 297 METs-min Rehab: 2.5 × 0.33 × 5 = 247.5 METs-min Total = 544.5 METs-min/week | FMA |
|  |  | Middle-income | 25 | Upper limb function | Mixed | Chronic Phase | Mild to moderate | 57.68±10.43 | M20/F5 | Y | RC |  | Conventional upper limb rehab: stretching, resistance, daily activity tasks | 5×/week, 35–40 min/session, 6weeks | 6weeks | 2.5 METs × 0.67 h × 5 = 502.5 METs-min/week |  |
| 10 | Jannette Blennerhassett 2004^134^ | High-income | 15 | Lower limb function | 11I + 4H | Subacute Phase | Mild to moderate | 53.9±19.8 | M8/F7 | Y | TOT | Inpatient stroke rehab; can walk 10 m under close supervision | 60-min daily circuit training (cycling, treadmill, stairs, balance, strength, etc.) | 5×/week, 60 min/session, 4weeks | 4weeks | 50% at 3.5 METs, 50% at 3.3 METs → average 3.4 3.4 × 1 h × 5 = 1020 METs-min/week | 6MWT |
|  |  | High-income | 15 | Upper limb function | 11I + 4H | Subacute Phase | Mild to moderate | 56.3±10.5 | M9/F6 | Y | ULT |  | 10 stations: ergometer, grasp tasks, hand–eye coordination, resistance | 5×/week, 60 min/session |  | 10 min ergometer at 2.8 METs + 50 min functional tasks at 2.5 → avg = 2.55 2.55 × 1 h × 5 = 765 METs-min/week |  |
| 11 | Sarah R.Valkenborghs 2019^135^ | High-income | 9 | Upper limb function | Mixed | Chronic Phase | Severe | 62.1±11.7 | M5/F4 | Y | HIIT | ≥16y; I/H stroke; WMFT<63 or ARAT<52; aerobic training eligible. | AEX + TST group: 30-min aerobic HIIT (4×4min at 85% HRmax + 3×3min at 70% HRmax) before task-specific training | AEX 30×3; TST 60×3. | 10weeks | AEX: 8×(16/60) + 4.5×(9/60) ≈ 4.01 MET·h = 240.6 METs/session × 3 = 721.8 METs/week; TST: 2.8 METs × 60 min × 3 = 504 METs/week; Total = 1225.8 METs/week | 6MWT |
|  |  | High-income | 11 | Upper limb function | Mixed | Chronic Phase | Moderate | 49.8±17.4 | M6/F5 | Y | TOT |  | TST only (task-specific training), 60 min/session | 60 min × 3/week |  | 2.8 METs × 60 min × 3 = 504 METs/week |  |
| 12 | H-J Eich 2004^136^ | High-income | 25 | Lower limb function | I | Subacute Phase | Moderate | 62.4±4.8 | M17/F8 | Y | GT | Age 50–75; BI 50–80; walk ≥12m (supervised); CV stable (≥50W ECG test) | Treadmill training (THR=60%HRR); 30 min/session with ECG; + Bobath training 30 min/session | Treadmill 30×5; Bobath 30×5. | 6weeks | Treadmill: 4.5 METs × 30 min × 5 = 675; Bobath: 3.0 METs × 30 min × 5 = 450; Total = 1125 METs/week | 10MWT |
|  |  | High-income | 25 | Lower limb function | I | Subacute Phase | Moderate | 64.0±6.0 | M17/F8 | Y | NPF |  | Bobath only (walking, stairs, gait, etc.) | 60 min × 5/week | 6weeks | 3.0 METs × 60 min × 5 = 900 METs/week |  |
| 13 | Jane E. Sullivan 2012^137^ | High-income | 20 | Upper limb function | Mixed | Chronic Phase | Moderate | 61.6±12.4 | M13/F7 | N | ESX | Stroke ≥6 months ago; FMA score 15–50/66; Able to do home-based training | Task-oriented training + sensory electrical stimulation (SES, glove electrode) | 30 min × 2 × 5/week | 4weeks | 2.5 METs × 30 min × 10 = 750 METs/week | FMA |
|  |  | High-income | 18 | Upper limb function | Mixed | Chronic Phase | Moderate | 59.5±14.2 | M14/F4 | N | TOT |  | Sham stimulation group (N actual current, placebo control) | 30 min × 2 × 5/week | 4weeks | 2.5 METs × 30 min × 10 = 750 METs/week |  |
| 14 | Marijn Mulder 2024^138^ | High-income | 21 | Lower limb & trunk balance dysf | 7H + 14I | Subacute Phase | Moderate | 61.3 | M11/F10 | Y | TOT | WHO stroke; ≤3 mo; no SAH; MoCA >21 | Armed4Stroke: Caregiver-mediated training + telerehabilitation + therapist supervision | Daily 30×5 + periodic therapist sessions | 8weeks | Caregiver: 157.5 min × 3.0 = 472.5; Self-training: 243.75 min × 3.0 = 731.25; Therapist: 177.5 min × 3.0 = 532.5; Total = 1736.25 METs/week | 6MWT  BBS |
|  |  | High-income | 20 | Lower limb & trunk balance dysf | 6H + 14I | Subacute Phase | Moderate | 63 | M12/F8 | Y | RC |  | Usual care (N structured exercise) | 30 min × 5/week | 8weeks | 484min×3.0 METs =1452METs-min/week |  |
| 16 | Yu Bai 2022^139^ | Middle- and high-income | 25 | Upper and lower limbs | Mixed | Chronic Phase | Moderate | 54.2±13.7 | M18/F7 | Y | VRG | Chinese National Conference on Cerebrovascular Disease (1996); Stroke diagNsis confirmed | Experimental group (VR + AI rehab): VR treadmill training, interactive upper/lower limb games, AI feedback system, 50 min/day, 5 days/week for 10weeks | 5 × 50 min/week | 10weeks | VR: ~75 METs/week + Routine rehab: 150 min × 3.0 = 450 METs/week → Total = 1325 METs/week | BBS  FMA-LE  FMA-UE |
|  |  | Middle- and high-income | 25 | Upper and lower limbs | Mixed | Chronic Phase | Moderate | 56.9±14.6 | M15/F10 | Y | RC |  | Control group (Routine rehab only) | 5 × 30 min/week | 10weeks | Routine rehab: 150 min × 3.0 = 450 METs/week |  |
| 17 | Chi-Ho Lee 2014^140^ | High-income | 10 | Lower limb function | Mixed | Chronic Phase | Mild to moderate | 47.9±12.0 | M8/F2 | Y | VRG | Stroke ≥6 months; MMSE ≥24; Able to stand ≥1 min; N musculoskeletal disorder affecting gait | AR-based postural control training (HMD system, core stability, sit-to-stand tasks) | 3 × 30 min/week (AR) + 5 × 30 min/week (Routine) | 4weeks | AR: 90 min/week × 3.0 = 270 METs/week; Routine: 150 min/week × 3.0 = 450 → Total = 720 METs/week | BBS |
|  |  | High-income | 11 | Lower limb function | Mixed | Chronic Phase | Mild to moderate | 54.0±11.9 | M6/F5 | Y | RC |  | Routine rehab only | 5 × 30 min/week | 4weeks | 150 min × 3.0 = 450 METs/week |  |
| 18 | Oluwole O. Awosika 2020^141^ | High-income | 11 | Lower limb function | 79%I + 21%H | Chronic Phase | Moderate | 54.7±10.9 | M10/F9 | N | ESX | Chronic stroke ≥6 months; 10MWT <1.2 m/s; MMSE >23 | Backward treadmill walking training (BLTT) + aNdal tsDCS | 3 × 30 min/week | 2weeks | 4.5 METs × 30 min × 3 = 405 METs/week | 6MWT  10MWT |
|  |  | High-income | 19 | Lower limb function | 82%I + 18%H | Chronic Phase | Moderate | 58.6±7.6 | M6/F5 | N | GT |  | BLTT + sham tsDCS | 3 × 30 min/week | 2weeks | 4.5 METs × 30 min × 3 = 405 METs/week |  |
| 19 | Dorian K. Rose, 2018^142^ | High-income | 8 | Primarily lower limbs | Mixed | Acute Phase | Moderate | 53.8±12.1 | M4/F4 | Y | GT | First stroke ≤30 days; Able to stand with moderate help; Good vision | Backward walking training on ground; balance, posture, gait enhancement | ~210 min/week + 870 min/week routine rehab | 2weeks | BW walking: 210 × 4.0 = 840; Rehab: 870 × 3.0 = 2610 → Total = 3450 METs/week | BBS |
|  |  | High-income | 8 | Primarily lower limbs | Mixed | Acute Phase | Severe | 66.6±7.3 | M2/F6 | Y | TOT |  | Standing balance training (SBT) | ~210 min/week + 870 min/week routine rehab | 2weeks | SBT: 210 × 3.0 = 630; Rehab: 870 × 3.0 = 2610 → Total = 3240 METs/week |  |
| 20 | Fu-Ling Tung, 2010^143^ | Middle- and high-income | 16 | Primarily lower limbs | Mixed | Chronic Phase | Moderate to severe | 51.0±12.1 | M9/F7 | Y | BT | Unilateral motor dysfunction post-stroke; BBS <50; Medically stable | Sit-to-stand training + routine rehab | 3 × (15 + 30) min/week | 4weeks | STS: 45 min × 4.0 = 180; Rehab: 90 min × 3.0 = 270 → Total = 450 METs/week | BBS |
|  |  | Middle- and high-income | 16 | Primarily lower limbs | Mixed | Chronic Phase | Moderate to severe | 52.7±14.1 | M11/F5 | Y | RC |  | Routine rehab only | 3 × 30 min/week |  | 90 min × 3.0 = 270 METs/week |  |
| 21 | Megumi ShimodozoN, 2012^144^ | High-income | 26 | Upper limb function | 46%I + 54%H | Subacute Phase | Moderate | 63.9±12.4 | M16/F10 | Y | ULT | CT-confirmed stroke; 3–13weeks post; Brunnstrom ≥ III | RFE: Repetitive facilitation + stretching, tapping, verbal cueing | 5 × 70 min/week (40 min active) | 4weeks | RFE: 40×5×3.5 = 700; Dexterity: 30×5×2.5 = 375 → Total = 1075 METs/week | FMA-UE |
|  |  | High-income | 23 | Upper limb function | 52%I + 48%H | Subacute Phase | Moderate | 63.9±12.4 | M10/F13 | Y | RC |  | Conventional rehab: ROM, grasping, passive/assistive tasks | 5 × 70 min/week (40 min active) | 4weeks | Rehab: 40×5×3.0 = 600; Dexterity: 30×5×2.5 = 375 → Total = 975 METs/week |  |
| 22 | Stefanie S. L. Lam, 2022^145^ | Middle- and high-income | 47 | Upper limb function | 81%I + 19%H | Subacute Phase | Moderate to severe | 65.1±10.0 | M27/F20 | Y | VRG | Stroke (1week to 6 months); AMT ≥ 7 | BMCG: Bilateral motor computer game (30 min) + 3h conventional rehab | 2 × 30 min/week + 3.5 h rehab × 2/week | 8weeks | Game: 60 × 3.5 = 210; Rehab: 360 × 3.0 = 1080 → Total = 1290 METs/week | FMA-UE |
|  |  | Middle- and high-income | 46 | Upper limb function | 81%I + 19%H | Subacute Phase | Moderate to severe | 66.0±9.0 | M28/F18 | Y | MBE |  | VDE: Video-directed upper limb exercise + 3h rehab | 2 × 30 min/week + 3.5 h rehab × 2/week | 8weeks | Video: 60 × 2.5 = 150; Rehab: 360 × 3.0 = 1080 → Total = 1230 METs/week |  |
| 24 | Mialinn A. Lindvall, 2014^146^ | High-income | 24 | Trunk | 67%I + 33%H | Chronic Phase | Mild to moderate | 62.1±11.4 | M12/F12 | N | MBE | ≥6 months post-stroke; walking ≥100m; balance issues | BAT: Body awareness therapy | 1 × 60 min/week | 8weeks | 2.5 METs × 60 = 150 METs/week | 6MWT  BBS |
|  |  | High-income | 22 | Trunk | 82%I + 18%H | Chronic Phase | Mild to moderate | 65.6±9.2 | M15/F7 | N | NE |  | Control group (N exercise) | 0 |  | 0 METs/week |  |
| 25 | Birgit Vahlberg, 2014^147^ | High-income | 20 | Lower limb and whole-body function | 81%I + 19%H | Chronic Phase | Mild to moderate | 72.7±5.5 | M17/F3 | N | RT | ≥1 year post-stroke; aged 65–85; N major health limits | PRB: Progressive resistance + balance training | 2 × 60 min/week | 3months | 120 min × 4.5 = 540 METs/week | 6MWT  BBS |
|  |  | High-income | 23 | Lower limb and whole-body function | 83%I + 17%H | Chronic Phase | Mild to moderate | 73.7±5.4 | M16/F7 | N | NE |  | Control group (N exercise) | None |  | 0 METs/week |  |
| 26 | Addie Middleton, 2014^148^ | High-income | 23 | Primarily lower limb function | Mixed | Chronic Phase | Moderate | 61.4±15.7 | M：61%/F：39% | N | GT | ≥6 months post-stroke; walk 20 ft; sit/stand 5 min | BWSTT: Body-weight support treadmill + gait + balance + strength | 180 min/day × 5 days/week | 2weeks | Gait: 60×2.8 = 168; Balance: 60×2.3 = 138; Strength: 60×2.8 = 168; Total/day = 474 × 5 = 2370 METs/week | 6MWT  BBS  FMA-LE |
|  |  | High-income | 20 | Primarily lower limb function | Mixed | Chronic Phase | Moderate | 60.7±11.4 | M：80%/F：20% | N | TOT |  | Overground task-oriented gait training | 180 min/day × 5 days/week | 2  weeks | Same as BWSTT → 2370 METs/week |  |
| 30 | Ping-An Zhu, 2024^149^ | Middle- and high-income | 18 | Primarily lower limb function | 13H + 5I5 | Chronic Phase | Moderate | 58.67±7.24 | M14/F4 | Y | ESX | ≥2weeks post-stroke; age 18–80; gait or balance deficits | iTBS group: Cerebellar theta burst stimulation + 60 min rehab | 5 × 60 min/week | 2  weeks | 60 × 2.8 = 168 × 5 = 840 METs/week | BBS  FMA-LE |
|  |  | Middle- and high-income | 18 | Primarily lower limb function | 13H + 5I | Subacute Phase | Moderate | 62.33±8.78 | M13/ F5 | Y | RC |  | Sham stimulation + rehab | 5 × 60 min/week | 2  weeks | Same as above → 840 METs/week |  |
| 31 | Elisabetta Peri, 2016^150^ | High income | 8 | Lower limb | Mixed | Subacute Phase | Moderate | 71.8±12.9 | F6/M2 | Y | ESX | First stroke (<6 months); MMSE ≥25; N joint ROM limitations | FES-cycling + conventional PT (25 + 50 min) | FES: 25 min × 5/wk; PT: 50 min × 5/wk | 3weeks | FES: 25×5×5.5 = 687.5; PT: 50×5×2.8 = 700 → Total = 1387.5 METs/week | 6MWT |
|  |  | High income | 8 | Lower limb | Mixed | Subacute Phase | Moderate | 76.4±8.7 | F3/M5 | Y | RC |  | Conventional PT only | 75 min × 5/week | 3weeks | 75×5×2.8 = 1050 METs/week |  |
| 32 | Kevin Moncion, 2024^151^ | High-income | 42 | Lower limb function | 75%I + 17%H + 8%Mixed | Chronic Phase | Mild | 65.4±8.9 | M：27/F：15 | N | HIIT | Age 40–80; stroke 6–60 months; mRS ≤2 | HIIT: 10×1 min high + 10×1 min low | 3 × 19 min/week | 12weeks | 3 × 138.5 = 415.5 METs/week | 6MWT 10MWT |
|  |  | High-income | 40 | Lower limb function | 83%I + 17%H | Chronic Phase | Moderate | 64.4±9.7 | M23/F17 | N | AE |  | MICT: Moderate continuous training | 3 × 20–30 min/week | 12weeks | 3 × 172.5 = 517.5 METs/week |  |
| 35 | Christoph Globas, 2012^152^ | High-income | 18 | Lower limb and trunk balance | I | Chronic Phase | Mild | 68.6±6.7 岁 | M14/F4 | N | AE | Age ≥ 60; stroke ≥6 months; treadmill walking ≥0.3 km/h | TAEX: High-intensity aerobic treadmill training (Karvonen formula, gradually increased speed and HRR) | 3 × ~39.4 min/week | 3months | 39.4 × 6.0 × 3 = 709.2 METs/week | 6MWT 10MWT BBS |
|  |  | High-income | 18 | Lower limb and trunk balance | I | Chronic Phase | Mild | 68.7±6.1 | M15/F3 | N | RC |  | Control: Conventional PT (balance, tone regulation, N aerobic component) | 3 × 36 min/week | 3months | 108 × 2.0 = 216 METs/week |  |
| 37 | Yajing Hu, 2022^153^ | Middle- and high-income | 26 | Lower limb | 10I + 14H | Chronic Phase | Moderate to severe | 56.22±10.37 | M15/F11 | Y | TAE | First stroke (CT/MRI); unilateral lower limb dysfunction | MOTOmed cycling + gait training (passive/assistive/active based on ability) | 6 × 20 min/week | 8weeks | 6.8 × 20 ÷ 60 × 6 × 60 = 816 METs/week | 10MWT FMA-LE |
|  |  | Middle- and high-income | 26 | Lower limb | 9I + 16H | Chronic Phase | Moderate to severe | 56.97±10.24 | M16/F10 | Y | GT |  | Control: Conventional gait training only | 6 × 20 min/week | 8weeks | 2.8 × 20 ÷ 60 × 6 × 60 = 336 METs/week |  |
| 38 | Ting-Ting Yeh, 2021^154^ | Middle- and high-income | 20 | Whole-body function | 8I8 + 12H | Chronic Phase | Mild to moderate | 53.05±14.53 | M12/F8 | N | MBE | Stroke ≥6 months; MMSE ≥19; MoCA <26; mild cognitive issues | SEQ Group: 30 min aerobic cycling + 30 min BrainHQ cognitive training | 3 × 60 min/week | 12weeks | 3.65 × 180 = 657 METs/week | 6MWT |
|  |  | Middle- and high-income | 18 | Whole-body function | 12I + 5H + 1Mixed | Chronic Phase | Mild to moderate | 57.36±12.17 | M13/F5 | N | AE |  | AE Group: 60 min aerobic cycling only | 3 × 60 min/week | 12weeks | 5.5 × 180 = 990 METs/week |  |
|  |  | Middle- and high-income | 18 | Whole-body function | 8I8 + 10H | Chronic Phase | Mild to moderate | 60.17±12.13 | M13/F5 | N | VRG |  | COG Group: 60 min cognitive training only | 3 × 60 min/week | 12weeks | 1.8 × 180 = 324 METs/week |  |
| 40 | Mahnaz Hejazi-Shirmard，2024^155^ | Middle income | 13 | Upper limb function | Mixed | Chronic Phase | Moderate | 61.54±8.93 | M7/F6 | Y | TOT | MCA stroke ≥6 months; MMSE ≥21; Brunnstrom 4–6 | Top-down: CO-OP (Goal-Plan-Do-Check) strategy rehab | 60 min × 2/week | 6weeks | 1.8 × 120 = 216 METs/week | FMA-LE |
|  |  | Middle income | 13 | Upper limb function | Mixed | Chronic Phase | Moderate | 59.08±16.99 | M11/F2 | Y | MBE |  | Bottom-up: Sensorimotor (strength, endurance, ROM) rehab | 60 min × 2/week | 6weeks | 3.5 × 120 = 420 METs/week |  |
|  |  | Middle income | 13 | Upper limb function | Mixed | Chronic Phase | Moderate | 62.29±12.70 | M8/F5 | Y | NPF |  | Control: Brunnstrom + PNF + motor rehab | 60 min × 2/week | 6weeks | 2.3 × 120 = 276 METs/week |  |
| 42 | MinKyu Kim, 2014^156^ | High-income | 11 | Primarily lower limbs | 5I + 6H | Chronic Phase | Mild to moderate | 50.18±10.29 | M6/F5 | Y | GT | Single stroke ≥6 mo, hemiparesis, K-MMSE ≥24, gait speed <0.8 m/s, can walk 10m independently | CWTP: Community walking program (30 min/day, 5 days/week × 4weeks) Week 1: 200m indoor;week 2: 300m on uneven terrain;week 3: 400m outside;week 4: mall walking | 30 min × 5/wk | 4weeks | Conventional rehab: 2.65 × 60 = 159; Walking: 4.0 × 30 = 120 → Total = 279 × 5 = 1395 METs/week | 6MWT 10MWT |
|  |  | High-income | 11 | Primarily lower limbs | 6I + 5H | Chronic Phase | Mild to moderate | 50.73±7.24 | M7/F4 | Y | RC |  | Conventional PT only (N community walking) | 60 min × 5/wk | 4weeks | 2.65 × 60 = 159 → 159 × 5 = 795 METs/week |  |
| 43 | Marco Y. C. Pang, 2005^157^ | High income | 32 | Whole-body function | 18I + 14H | Chronic Phase | Mild to moderate | 65.8±9.1 | M19/F13 | N | FT | ≥1 year post-stroke, living in community, can walk ≥10m | FAME: Aerobic + gait + balance + resistance training (60 min, 3×/week) | 60 min × 3/wk | 19weeks | 4.5 × 180 = 810 METs/week | 6MWT BBS |
|  |  | High income | 31 | Whole-body function | 19I + 12H | Chronic Phase | Mild to moderate | 64.7±8.4 | M18/F13 | N | ULT |  | Upper-limb training only (N aerobic or lower-limb training) | 60 min × 3/wk | 19weeks | 2.0 × 180 = 360 METs/week |  |
| 44 | Marco Y. C. Pang, 2006^158^ | High income | 30 | Upper limb function | 19I19 + 11H | Chronic Phase | Mild to moderate | 64.9±8.5 | M18/F12 | N | ULT | ≥1 year post-stroke, confirmed by CT, walk ≥10m with assist | Upper-limb functional training + electrostimulation + warm-up/cool-down | 60 min × 3/wk | 19weeks | 3.0 × 180 = 540 METs/week | FMA |
|  |  | High income | 30 | Lower limb function | 17I + 13H | Chronic Phase | Mild to moderate | 66.0±8.7 | M18/F12 | N | LLT |  | Lower-limb strength + balance + aerobic training | 60 min × 3/wk | 19weeks | 4.0 × 180 = 720 METs/week |  |
| 45 | Attiya Irshad, 2022^159^ | Middle income | 43 | Lower limb | 55.8%I + 44.2%H | Chronic Phase | Mild to moderate | 30-70 | M25/F18 | Y | VT | ≥6 mo stroke, able to stand independently, N cardiovascular/vestibular contraindications | WBV: Whole-body vibration (Crazy Fit Massage WBV device, progressive intensity 30Hz–50Hz) | 30 min × 5/wk | 6weeks | 3.5 × 300 = 1050 METs/week | BBS |
|  |  | Middle income | 43 | Lower limb | 69.8%I + 30.2%H | Chronic Phase | Mild to moderate | 30-70 | M23/F20 | Y | RT |  | Squat training (progressive angles, plus gait & balance drills) | 30 min × 5/wk | 6weeks | 4.0 × 300 = 1200 METs/week |  |
| 46 | Ying Zhang, 2022^160^ | Middle- and high-income | 80 | Whole-body function | 81.2%I + 18.8%H | Subacute Phase | Mild to moderate | 65.44±9.16 | M56/F24 | Y | MBE | DiagNsed with stroke (I or H); Brunnstrom stage ≥ 4 in at least one upper limb; MMSE ≥ 23. | Liuzijue Qigong combined with conventional rehab: breathing exercises with six sounds (Xu, He, Hu, Si, Chui, Xi), trunk rotation, and limb movements; 15 min qigong + 30 min rehab. | Daily × 45 min (15 min Liuzijue + 30 min rehab), 5 times/week for 2weeks | 2weeks | 2.83 × 225 = 636.75 METs-min/week | BBS FMA |
|  |  | Middle- and high-income | 80 | Whole-body function | 76.2%I + 23.8%H | Subacute Phase | Mild to moderate | 62.80±11.18 | M64/F16 | Y | CST |  | Core stability training (CST) combined with conventional rehab: breathing, bridging, and pelvic control; same frequency as experimental group. | Daily × 45 min (15 min CST + 30 min rehab), 5 times/week for 2weeks | 2weeks | 3.27 × 225 = 735.75 METs-min/week |  |
| 49 | Jae Ho Park, 2018^161^ | High income | 8 | Lower limb function | 8I + 1H | Chronic Phase | Mild to moderate | 62.88±12.90 | M6/F3 | Y | WA | Stroke ≥ 6 months; BBS ≤ 45; MMSE ≥ 24; able to walk with assistive device. | Underwater treadmill (AT): Aqua-Track treadmill walking in 30–32°C water, water depth to xiphoid process. | 5 times/week × 30 min | 4weeks | 3.94 × 240 = 945.6 METs-min/week | 10MWT BBS |
|  |  | High income | 8 | Lower limb function | 7I + 1H | Chronic Phase | Mild to moderate | 66.37±10.28 | M4/F4 | Y | GT |  | Anti-gravity treadmill (AGT): gradually increased treadmill walking with bodyweight support. | 5 times/week × 30 min | 4weeks | 3.38 × 240 = 811.2 METs-min/week |  |
|  |  | High income | 10 | Lower limb function | 6I + 4H | Chronic Phase | Mild to moderate | 67.10±7.63 | M5/F5 | Y | AE |  | Conventional treadmill (CON): N support; same speed as experimental group. | 5 times/week × 30 min | 4weeks | 3.49 × 240 = 837.6 METs-min/week |  |
| 50 | Mi-Joung Lee, 2008^162^ | High income | 14 | Lower limb function | 8I + 2H + 2Mixed | Chronic Phase | Mild to moderate | 67.2±10.6 | M8/F5 | N | AE | Stroke ≥ 3 months; walking capacity 0.15–1.4 m/s; N regular rehab. | Aerobic cycling: semi-recumbent ergometer, HR 50–70% VO2peak, 30 min/session. | 3 times/week × 60 min, 10–12weeks | 10-12weeks | 3.25 × 180 = 585 METs-min/week | 6MWT |
|  |  | High income | 13 | Lower limb function | 9I + 2H + 3Mixed | Chronic Phase | Mild to moderate | 62.9±9.3 | M6/F8 | N | RT |  | Strength training: lower limb resistance training (leg extension, flexion, dorsiflexion, abduction); 2 sets of 8 reps; load increased from 50% to 80% 1RM. | 3 times/week × 60 min, 10–12weeks | 10-12weeks | 3.40 × 180 = 612 METs-min/week |  |
|  |  | High income | 13 | Lower limb function | 7I + 4H + 2Mixed | Chronic Phase | Mild to moderate | 60.5±10.6 | M8/F5 | N | FT |  | Combined aerobic + strength: 30 min ergometer + 30 min resistance per session. | 3 times/week × 60 min, 10–12weeks | 10-12weeks | 5.75 × 180 = 1035 METs-min/week |  |
|  |  | High income | 12 | Lower limb function | 9I + 1H + 2Mixed | Chronic Phase | Mild to moderate | 65.3±6.0 | M6/F6 | N | RC |  | SHAM group: passive limb cycling + minimal load strength training. | 3 times/week × 60 min, 10–12weeks | 10-12weeks | 1.30 × 180 = 234 METs-min/week |  |
| 53 | Fatima Younas, 2024^163^ | Middle income | 33 | Trunk | I | Chronic Phase | Moderate | 62.3±10.2 | M24/F9 | Y | TOT | I Stroke ≥6 months; BBS 20–40; MMSE ≥24; able to sit unsupported 30s. | TOT Group: 7 trunk tasks: sitting stability, standing balance, goal-reaching, walk-related tasks, pelvic control, and resistance training. | 3×/wk × 60 min | 12weeks | 2.90 × 180 = 522 | BBS |
|  |  | Middle income | 33 | Trunk | I | Chronic Phase | Moderate | 60.3±10.6 | M22/F11 | Y | NPF |  | Bobath Training: Stretching, core strength, posture adjustment, trunk coordination, support training. | 3×/wk × 60 min | 12weeks | 2.50 × 180 = 450 |  |
| 54 | Ümmügülsüm Doğan Duran, 2023^164^ | High income | 13 | Lower limb | I | Chronic Phase | Moderate | 54.1±18.9 | Nt reported | Y | GT | I Stroke ≥3 months; MMSE ≥20. | AGTT: Alter-G treadmill: 5 min warm-up, 30 min walk, 5 min cooldown, 65% body weight support. | 3×/wk × 45 min + 5×/wk × 45 min | 4weeks | 540 + 675 = 1215 | 6MWT BBS |
|  |  | High income | 13 | Lower limb | I | Chronic Phase | Moderate | 56.1±18.4 | Nt reported | Y | WA |  | UWT: Underwater walking: 5 min warm-up, 30 min walking, 5 min cooldown in 29–30°C water. | 3×/wk × 45 min + 5×/wk × 45 min | 4weeks | 472.5 + 675 = 1147.5 |  |
|  |  | High income | 13 | Lower limb | I | Chronic Phase | Moderate | 57.9±10.9 | Nt reported | Y | RC |  | CT Group: Conventional therapy + occupational therapy. | 5×/wk × 45 min | 4weeks | 45min × 5次/week × 3.0 METs = 675 METs-min/week |  |
| 57 | Jae-Ho Park, 2017^165^ | High income | 10 | Lower limb and trunk | 30%I + 70%H | Chronic Phase | Moderate | 56.50±10.48 | 5M/5F | Y | WA | DiagNsed with I or H stroke ≥6 months ago. Able to walk 10m independently (assistive devices allowed). MMSE ≥22. BBS ≤45. N fear of water. | Underwater Walking Group (UWG): Water depth to xiphoid process, temperature 28–30°C. Ankle weight equal to 5% body weight on paretic side. Customized walking rhythm via metroNme.week 3: speed +5%,week 5: +5% again. Includes straight, rotational, and crossover walking. | 30 min × 3 sessions/week × 6weeks | 6weeks | 30 min × 3 × 5.8 METs = 522 METs-min/week + Routine: 30 min × 5 × 3.0 = 450 Total = 972 METs-min/week | BBS |
|  |  | High income | 9 | Lower limb and trunk | 55.6%I + 44.4%H | Chronic Phase | Moderate | 57.22±16.96 | 5M/4F | Y | GT |  | Overground Walking Group (OG): Walking at a comfortable pace on land. Cadence maintained with metroNme for gait symmetry. | 30 min × 3 sessions/week × 6weeks | 6weeks | 30 min × 3 × 3.5 METs = 315 METs-min/week + Routine: 30 min × 5 × 3.0 = 450 Total = 765 METs-min/week |  |
| 59 | Yan Xu, 2025^166^ | Middle- and high-income | 18 | Lower limb and trunk | Mixed | Subacute and chronic | Moderate | 49.00±18.41 | 15M/3F | N | VRG | Hemiparesis post-stroke, confirmed via CT or MRI. Age 18–80. Able to stand 20–30 min. MMSE ≥20. | Experimental Group (EG): Visual feedback training with Physio Space™ (touchscreen + 3D balance platform). 9 game modules for multidirectional weight shifting. 3 games/session × 2 rounds. Difficulty adjusted based on 80% accuracy threshold. | 20 min × 5 sessions/week × 3weeks | 3weeks | 20 × 5 × 3.8 METs = 380 METs-min/week | 10MWT BBS |
|  |  | Middle- and high-income | 18 | Lower limb and trunk | Mixed | Subacute and chronic | Moderate | 51.11±14.74 | 18M/0F | N | RC |  | Control Group (CG): Traditional balance training. Includes static weight shifting, target pointing, foam standing, and eY-open/closed stance. Difficulty progressed by therapist. | 20 min × 5 sessions/week × 3weeks | 3weeks | 20 × 5 × 2.5 METs = 250 METs-min/week |  |
| 62 | Ayla Fil Balkan, 2024^167^ | Middle- and high-income | 10 | Trunk | Mainly I | Acute Phase | Moderate | 65.81±10.69 | 8M/ 3F | N | NPF | First-ever I or H stroke, confirmed by CT/MRI. GCS ≥14. MMSE >24. mRS ≥2 within 1week post-stroke. | Bobath Training (Moderate group): Focus on Nrmalizing trunk tone, active trunk movement. Exercises include: seated balance (foam), bridging, pelvic control, standing balance, bed rolling. | 60 min × 5 sessions/week (during inpatient) | Duringinpatient | 60 × 5 × 3.8 METs = 1140 METs-min/week | BBS |
|  |  | Middle- and high-income | 10 | Trunk | Mainly I | Acute Phase | Moderate | 65.31±8.18 | M14/F0 | N | RC |  | Bobath Training (Severe group): Same protocol as moderate group, targeting more severely impaired patients. | 60 min × 5 sessions/week | Duringinpatient | 60 × 5 × 3.0 METs = 900 METs-min/week |  |
|  |  | Middle- and high-income | 10 | Trunk | Mainly I | Acute Phase | Severe | 66.21±9.20 | M14/F15 | N | NPF |  | Control (Moderate): Individualized PT focused more on limbs than trunk. Routine rehab: ROM, strength, gait/balance. | 60 min × 5 sessions/week | Duringinpatient | 60 × 5 × 3.8 METs = 1140 METs-min/week |  |
|  |  | Middle- and high-income | 10 | Trunk | Mainly I | Acute Phase | Severe | 66.22±11.24 | M5/F9 | N | RC |  | Control (Severe): Same as above with lower functional capacity. Less core focus. | 60 min × 5 sessions/week | Duringinpatient | 60 × 5 × 3.0 METs = 900 METs-min/week |  |
| 64 | GyuChang Lee, 2015^168^ | High income | 12 | Lower limb and trunk | 7I + 5H | Chronic Phase | Moderate | 59.3±13.2 | M8/F4 | Y | VT | Chronic I/H (CT/MRI); ≥6mo; stable; stand ≥10min; no AV/ortho; MMSE >24 | Whole-Body Vibration using Extream 1000 (AMH International, Korea). Frequency 1-3 Hz, amplitude 30 mm. Training: 10 min anterior-posterior vibration + 10 min lateral vibration (total 20 min), 3 times/week for 6weeks. | 20 min × 3 sessions/week | 6weeks | Specific training: 20 min × 3 × 3.0 METs = 180 METs-min/week Regular rehab: 30 min × 5 × 3.0 METs = 450 METs-min/week Total: 630 METs-min/week | BBS FMA-LE |
|  |  | High income | 9 | Lower limb and trunk | 7I + 2H | Chronic Phase | Moderate | 56.0±9.1 | M6/F3 | Y | RC |  | Neurodevelopmental therapy, strength, balance, gait training without vibration. | 30 min × 5 sessions/week | 6weeks | 30 min × 5 × 3.0 METs = 450 METs-min/week |  |
| 65 | Seyedeh Saeideh Babazadeh-Zavieh, 2024^169^ | Low- and middle-income | 12 | Upper limb | Mixed | Chronic Phase | Moderate to severe | 52.50±8.60 | M8/F4 | N | NE | First-ever unilateral stroke ≥6 months, confirmed by CT/MRI. Wrist flexor spasticity ≥1 (MMAS), MMSE >24. | Dry needling on flexor carpi radialis and ulnaris, 1 min per muscle, 1 session/week for 4weeks. | 1 session/week, ~5 min/session | 4weeks | 0 (N exercise METs load) | FMA |
|  |  | Low- and middle-income | 12 | Upper limb | Mixed | Chronic Phase | Moderate to severe | 62.83±11.32 | M5/F7 | N | TCMEX |  | (DN + Exercise Therapy): DN as above + 30 min functional/strength training per day, 7 days/week | 30 min/day × 7 days/week | 4weeks | 30 × 7 × 3.5 METs = 735 METs-min/week |  |
| 66 | Marilyn MacKay-Lyons, 2013^170^ | High income | 24 | Lower limb and trunk | I | Subacute Phase | Mild to moderate | 61.5±15.4 | M15/F9 | Y | GT | First-ever I stroke, confirmed by CT or MRI; duration <1 month. Able to walk 5 meters with assistance (walker, AFO, or support). | BWSTT group: Body-weight-supported treadmill training using a Pneu-Weight system. Initial unloading of 20–40% body weight, gradually reduced. Training targeted 40–50% VO2peak at first, increasing to 60–75%. Walking speed progressed from 0.26–0.49 m/s to 0.56–0.87 m/s. Total session: 60 min (25–30 min treadmill + strength/stretching). Sessions: 5x/week inpatient + 3x/week outpatient. | 60 min/session × 5x/week (inpatient) or 3x/week (outpatient) | 12weeks | Inpatient specific training: 27.5 min × 5 × 3.5 METs = 401.04 METs-min/week Outpatient specific training: 27.5 × 3 × 3.5 = 240.62 METs-min/week Remaining 32.5 min conventional therapy:Inpatient: 32.5 × 5 × 3.0 = 812.5 Outpatient: 32.5 × 3 × 3.0 = 487.5 Total:Inpatient: 401.04 + 812.5 = 1213.54 METs-min/week Outpatient: 240.62 + 487.5 = 728.12 METs-min/week | 6MWT 10MWT BBS |
|  |  | High income | 26 | Lower limb and trunk | I | Subacute Phase | Mild to moderate | 59.0±12.7 | M14/F12 | Y | RC |  | 5–10 min pre-walk balance/weight shift → 20–25 min overground walking (self-selected speed) → remaining time for limb strength/stretch. | 60min/session × 5x/week (inpatient)or3x/week(outpatient) | 12weeks | Inpatient: 27.5 × 5 × 2.8 = 320.83 + 812.5 = 1133.33  Outpatient: 27.5 × 3 × 2.8 = 192.5 + 487.5 = 680.00 |  |
| 67 | BruN Bavaresco Gambassi, 2019^171^ | Middle- and high-income | 11 | Whole-body function | I | Chronic Phase | Mild to moderate | 66.4±10.1 | M5/F6 | Y | RT | Confirmed stroke (CT/MRI); duration ≥6 months; community-dwelling; able to complete Barthel Index for basic ADLs. | Elastic-band dynamic resistance training. 4 exercises: seated row, sit-to-stand, chest press, knee extension. Circuit style, concentric as fast as possible, eccentric controlled (3s). Progression:weeks 1–4: 6–8 reps, Borg 3–5;weeks 5–8: 10–12 reps, same intensity. Adjusted per RPE. | 2×/week, ~50min (30 resistance + 20conventional) | 8weeks | Resistance: 60 × 2 × 4.0 = 480  Conventional: 30 × 2 × 3.0 = 180  Total: 660 METs-min/week | 10MWT |
|  |  | Middle- and high-income | 11 | Whole-body function | I | Chronic Phase | Mild to moderate | 60.5±13.2 | M7/F4 | Y | NPF |  | Conventional neuro rehab (ADL simulation, posture control, walking on bars). | 2×/week, ~50min (30 resistance + 20conventional) | 8weeks | 30 × 2 × 3.0 = 180 METs-min/week |  |
| 68 | Brunelli, 2020^172^ | High income | 12 | Lower limb and trunk | 75%I + 25%H | Subacute Phase | Mild | 58.1±20.4 | M7/F5 | Y | BT | CT/MRI confirmed stroke. | 40 min Computerized Balance Training (CBT) using Biodex SD + 40 min conventional PT. CBT included postural stability, weight shift, natural weight distribution, LOS, labyrinth control, random control. | 5x/week, 40 min CBT + 40 min PT = 80 min/day | 4weeks | CBT: 40 × 5 × 3.0 = 600  PT: 40 × 5 × 3.0 = 600  Total: 1200 METs-min/week | BBS |
|  |  | High income | 12 | Lower limb and trunk | 66.7%I + 33.3%H | Subacute Phase | Mild | 59.7±14.2 | M6/F6 | Y | TOT |  | Conventional PT only: 2 × 40 min/day × 5 days | 5x/week, 40 min CBT + 40 min PT = 80 min/day | 4weeks | 40 × 2 × 5 × 3.0 = 1200 METs-min/week |  |
| 69 | Lattouf, 2021^173^ | Low-income | 19 | Lower limb | 14I + 5H | Chronic Phase | Moderate | 65.1±11.17 | F10/M9 | Y | RT | DiagNsed by a neurologist based on standard stroke criteria, including stroke history and imaging (CT or MRI). | Training performed on a horizontal leg press using eccentric contraction of the paretic limb. Includes concentric (push), static (hold), and eccentric (controlled return) phases. Load set at 40% 1RM for first two sets, 60% 1RM for final set. Aims to strengthen the paretic limb and improve walking ability. | 3×/week, 3 sets/session, 5 reps/set. | 4weeks | Leg press: 15 min × 3/wk × 3.5 METs = 157.5 Conventional PT: 300 min × 3.0 METs ÷ 60 = 1500 → Total: 1657.5 METs-min/week | 6MWT 10MWT |
|  |  | Low-income | 18 | Lower limb | 14I + 4H | Chronic Phase | Moderate | 68.7±12.4 | F7/M11 | Y | RC |  | Received routine rehab twice daily for 30 minutes each, including complication prevention, selective motor control training, spasticity management, functional mobility training, gait training, and patient education. | 5×/week, 2×/day, 30min/session. | 4weeks | Conventional PT only: 300 min × 3.0 METs ÷ 60 = 1500 METs-min/week |  |
| 71 | Ghrouz, 2024^174^ | High-income | 32 | Lower limb and trunk | 23I + 9H | Subacute Phase | Mild to moderate | 64.2±10.2 | M23/F9 | Y | MBE | First-ever subacute stroke (1–6 months), confirmed by a neurologist with CT or MRI. | Task-specific training based on motor relearning principles to improve balance and posture. Exercises include bed mobility, seated balance (lateral flexion, trunk rotation), sit-to-stand, standing balance (multidirectional reach, heel/toe stance, single-leg stance), and gait training (forward, backward, sideways, crossover, tandem, head movement during walking). | 3×/week, 60 min/session, 8weeks. | 8weeks | Task training: 60 min × 3/wk × 3.5 METs = 630  Conventional PT: 300 min × 3.0 METs ÷ 60 = 1500 → Total: 2130 METs-min/week | BBS |
|  |  | High-income | 31 | Lower limb and trunk | 23I + 8H | Subacute Phase | Mild to moderate | 66.3±8.6 | M18/F13 | Y | RC |  | Standard rehab including progressive passive stretching (shoulder, elbow, wrist, hamstrings, calf), active-assisted movements (hip, knee, ankle, upper limb), strength training (hip abductors, quadriceps, hamstrings), balance training (reach beyond arm length), and gait training (including dynamic balance and obstacle walking). | 3×/week, 60 min/session, 8weeks. | 8weeks | CPT training: 60 min × 3/wk × 3.0 METs = 540  Conventional PT: 300 min × 3.0 METs ÷ 60 = 1500 → Total: 2040 METs-min/week |  |
| 73 | Ozen et al., 2021^175^ | Middle- and high-income | 15 | Upper limb | 12I + 3H | Subacute Phase | Mild to moderate | 62.00±13.12 | M10/F5 | Y | VRG | Aged 18–85, stroke ≥3 months (I or H), BSSR ≥3, MMSE ≥23 (N severe cognitive impairment). | Participants received 60 minutes of traditional neurorehabilitation combined with 30 minutes of computer-based training using the Rejoyce interactive game system. | 90 min/session, 5×/week | 4weeks | Task training: 30 min × 5 × 3.0 METs = 450 Conventional PT: 60 min × 5 × 3.0 METs = 900 Total: 1350 METs-min/week | FMA-UE |
|  |  | Middle- and high-income | 15 | Upper limb | 13I + 2H | Subacute Phase | Mild to moderate | 69.80±8.41 | M10/F5 | Y | NPF |  | Participants received 60 minutes of traditional neurorehabilitation followed by 30 minutes of occupational therapy focusing on daily living task practice. | 90 min/session, 5×/week | 4weeks | Task training: 30 min × 5 × 2.8 METs = 420 Conventional PT: 60 min × 5 × 3.0 METs = 900 Total: 1320 METs-min/week |  |
| 74 | Kim et al., 2012^176^ | High-income | 10 | Lower limb and trunk | Mainly I | Chronic Phase | Mild to moderate | 52.50±11.72 | M7/F3 | Y | TOT | Walk ≥10m (±aid); K-MMSE ≥20. | Participants underwent 60 minutes of conventional rehabilitation plus 60 minutes of task-oriented gait training involving 10 real-life walking tasks. | 120 min/session, 3×/week | 4weeks | Specialized training: 60 min × 3 × 4.0 METs = 720 Conventional PT: 60 min × 5 × 3.0 METs = 900 Total: 1620 METs-min/week | 10MWT BBS |
|  |  | High-income |  | Lower limb and trunk | Mainly I | Chronic Phase | Mild to moderate | 53.40±12.11 | M5/F5 | Y | RC |  | Participants received 60 minutes of standard rehabilitation, including joint mobilization, muscle strengthening, and balance training. | 60 min/session, 5×/week | 4weeks | Conventional PT: 60 min × 5 × 3.0 METs = 900 Total: 900 METs-min/week |  |
| 75 | Kim & Jang, 2022^177^ | High income | 10 | Lower limb and trunk | Mixed | Subacute Phase | Mild to moderate | 61.50±8.04 | M5/F5 | Y | RT | MMSE-K≥ 24 BBS≥ 21Able to walk independently without assistive device (MAS) ≤ 2 | Participants performed 30 minutes of conventional rehab and 30 minutes of trunk stabilization training with added limb loading. | 60 min/session, 5×/week | 8weeks | Task training: 30 min × 5 × 4.0 METs = 600 Conventional PT: 300 min × 3.0 METs ÷ 60 = 1500 Total: 2100 METs-min/week | 10MWT BBS |
|  |  | High income | 10 | Lower limb and trunk | Mixed | Subacute Phase | Mild to moderate | 61.70±6.66 | M4/F6 | Y | CST |  | Participants received 30 minutes of rehab followed by 30 minutes of trunk stabilization training without external loading. | 60 min/session, 5×/week | 8weeks | Task training: 30 min × 5 × 3.5 METs = 525 Conventional PT: 300 min × 3.0 METs ÷ 60 = 1500 Total: 2025 METs-min/week |  |
|  |  | High income | 10 | Lower limb and trunk | Mixed | Subacute Phase | Mild to moderate | 61.60±3.92 | M5/F5 | Y | RC |  | Participants received 30 minutes of basic rehabilitation focusing on ROM, gait training, and balance. | 30min/session,5×/week | 8weeks | 300 min × 3.0 METs ÷ 60 = 1500 Total: 1500 METs-min/week |  |
| 76 | In-Wook Lee, 2015^178^ | High income | 10 | Lower limb and trunk | Mixed | Chronic Phase | Mild to moderate | 57.2±9.2 | M6/F4 | N | VRG | DiagNsed by CT and MRI; stroke onset > 6 months | Participants completed 45-minute sessions using the BioRescue VR system, combining weight-shifting virtual tasks and cognitive challenges. | 45min/session,3×/week | 6weeks | 45 min × 3 × 3.5 METs = 472.5 Total: 472.5 METs-min/week | BBS |
|  |  | High income | 10 | Lower limb and trunk | Mixed | Chronic Phase | Mild to moderate | 52.7±11.7 | M5/F5 | N | NPF |  | Participants received 45-minute proprioceptive neuromuscular facilitation (PNF) sessions targeting hip movement patterns. | 45min/session, 3×/week | 6weeks | 45 min × 3 × 3.0 METs = 405 Total: 405 METs-min/week |  |
| 77 | Jong Hwa Lee, 2017^179^ | High income | 15 | Trunk | 9I + 6H | Subacute Phase | Severe | 64.4±14.8 | M7/F8 | Y | RC | Confirmed by CT and MRI Static Trunk Impairment Scale (TIS-S) < 2 | Participants performed seated balance training supervised by therapists, including postural alignment and limb reaching. | 30 min × 2×/day, 5×/week | 2weeks | 30 × 2 × 5 × 3.0 METs = 900 METs-min/week Total: 900 METs-min/week | BBS |
|  |  | High income | 15 | Trunk | 8I + 7H | Subacute Phase | Severe | 59.1±16.9 | M8/F7 | Y | VT |  | Participants received whole-body vibration therapy on a Sonix platform while seated, supervised by therapists. | 30 min × 2×/day, 5×/week | 2weeks | WBV training: 30 × 5 × 2.5 METs = 375 Conventional rehab: 30 × 5 × 3.0 = 450 Total: 825 METs-min/week |  |
| 80 | Kamal Narayan Arya, 2019^180^ | High income | 19 | Trunk | 4I + 2H | Chronic Phase | Moderate | 46.44±7.89 | M 3/F 3 | Y | MBE | CT/MRI confirmed; FAC ≥2; no major impairments; walk ≥10m unaided. | Participants engaged in functional lower-limb training in front of a mirror, simulating bilateral movement using only the unaffected side. | 30 min × 3–4×/week | 12weeks | 30 × 5 × 3.0 = 450 Dance for PD via Zoom: 40 × 2 × 3.5 = 280 Total: 730 METs-min/week | 10MWT FMA-LE |
|  |  | High income | 17 | Trunk | 5I + 1H | Chronic Phase | Moderate | 44.53±6.09 | M 3/F 3 | N | RC |  | Participants received Bobath and Brunnstrom-based gait rehabilitation without mirror involvement. | 30 min × 3–4×/week | 12weeks | Conventional rehab: 30 × 5 × 3.0 = 450 Total: 450 METs-min/week |  |
| 81 | Cabanas-Valdés, 2016^181^ | High income | 40 | Trunk | 33I + 7H | Subacute Phase | Moderate | 74.92±10.70 | F19/M21 | Y | CST | WHO-based diagnosis, clinically assessed, MRI confirmed. | Participants performed an additional 15 minutes of core stability training daily, progressing from supine to unstable seated tasks. | 15 min × 5×/week | 5weeks | Task: 15 × 5 × 3.5 = 262.5 Conventional PT: 60 × 5 × 3.0 = 900 Total: 1162.5 METs-min/week | BBS |
|  |  | High income | 40 | Trunk | 31I + 8H | Subacute Phase | Moderate | 75.69±9.40 | F21/M18 | Y | RC |  | Participants received only routine physical and occupational therapy sessions (60 min/day). | 60 min × 5×/week | 5weeks | Conventional PT: 60 × 5 × 3.0 = 900 Total: 900 METs-min/week |  |
| 82 | Gordon, 2013^182^ | Middle- and high-income | 64 | Lower limb | 48I + 7H | Chronic Phase | Mild to moderate | 63.4±9.4 | M29/F35 | N | GT | ≥40y; community-dwelling; ambulatory (±aid) | Brisk walking on a set route, beginning with 15 minutes and gradually increasing to 30 minutes. Target heart rate was 60–85% of predicted maximum. Training was supervised and performed in home or community environments. | 30 min × 3×/week | 12weeks | Assigned MET: Brisk walk = 4.3 30 × 3 × 4.3 = 387 Total: 387 METs-min/week | 6MWT |
|  |  | Middle- and high-income | 64 | Lower limb | 43I + 8H | Chronic Phase | Mild to moderate | 64.9±11.1 | M29/F35 | N | TCMEX |  | Participants received gentle massage focused on the affected limbs. Each session lasted 25 minutes. | 25 min × 3×/week | 12weeks | Assigned MET: Passive seated activity = 1.3 25 × 3 × 1.3 = 97.5 Total: 97.5 METs-min/week |  |
| 83 | Oh, 2022^183^ | High income | 15 | Lower limb and trunk | Mixed | Chronic Phase | Moderate | 71.20±6.87 | M15/F0 | N | GT | Chronic stroke patients (onset ≥ 6 months); | Gait training using an anti-gravity treadmill with progressive loading. Sessions began with 30% bodyweight support and gradually increased to 80%. Each session lasted 20 minutes. | 20 min × 5×/week | 4weeks | Intervention Details: 20 × 5 × 2.5 = 250 Total: 250 METs-min/week | 10MWT BBS |
|  |  | High income | 15 | Lower limb and trunk | Mixed | Chronic Phase | Moderate | 74.80±3.67 | M14/F1 | N | RC |  | Conventional gait and balance training led by therapists. Sessions lasted 20 minutes and involved overground walking without special equipment. | 20 min × 5×/week | 4weeks | Intervention Details: 20 × 5 × 2.8 = 280 Total: 280 METs-min/week |  |
| 85 | Nh, 2008^184^ | High income | 13 | Lower limb and trunk | 6I + 7H | Chronic Phase | Mild to moderate | 61.9±10.1 | M7/F6 | N | WA | First stroke; ambulatory (±aid); stable; no severe CVD; LL by CMSA. | Aquatic therapy using Halliwick and Ai Chi methods. Sessions included warm-up, balance control, limb coordination, and cool-down, with a total duration of 60 minutes. | 60 min × 3×/week | 8weeks | Intervention Details: 60 × 3 × 5.3 = 954 Total: 954 METs-min/week | BBS |
|  |  | High income | 12 | Lower limb and trunk | 7I + 5H | Chronic Phase | Mild to moderate | 66.0±11.4 | M7/F6 | N | RC |  | Land-based rehabilitation including flexibility, strength, balance, and gait exercises. Each session lasted 60 minutes with structured warm-up and cool-down phases. | 60 min × 3×/week | 8weeks | Intervention Details: 60 × 3 × 3.0 = 540 Total: 540 METs-min/week |  |
| 86 | Gerdienke B. Prange, 2015^185^ | High income | 35 | Upper limb | 28I + 7H | Subacute Phase | Moderate to severe | 60.3±9.7 | M17/F16 | Y | VRG | UL by FM & SULCS. | Upper limb training using ArmeoBoom robotic device with game-based interaction. Arm weight was supported via a suspension system, and difficulty was progressively adjusted. | 60 × 3 × 3.0 = 540 Total: 540 METs-min/week | 6weeks | Intervention: 30 × 3 × 2.8 = 252 Conventional rehab (OT/PT 2×): 30 × 2 × 3.0 = 180 Total: 432 METs-min/week | FMA |
|  |  | High income | 33 | Upper limb | 25I + 8H | Subacute Phase | Severe | 58±11.4 | M24/F9 | Y | ULT |  | Traditional tabletop upper limb training involving object movement and stacking tasks with increasing complexity. | 30 min × 3×/week | 6weeks | Intervention: 30 × 3 × 2.5 = 225 Conventional rehab: 30 × 2 × 3.0 = 180 Total: 405 METs-min/week |  |
| 87 | Sang-Seok Yeo, 2023^186^ | High income | 13 | Trunk | 8H + 5I | Chronic Phase | Moderate | 44.85±15.63 | M9/F4 | N | FT | Stroke dx (neurologist);stand/walk ≥10min over 6m. | Seated balance training performed on an unstable surface with additional laser visual feedback. Training lasted 20 minutes. | 20 min × 3×/week | 4weeks | 20 min × 3×/week × 3.5 METs = 210 | BBS |
|  |  | High income | 13 | Trunk | 8H + 5I | Chronic Phase | Moderate | 56.92±8.95 | M11/F2 | N | BT |  | Balance training on an unstable surface without visual feedback. Each session was 20 minutes. | 20 min × 3×/week | 4weeks | 20 min × 3×/week × 3.5 METs = 210 |  |
|  |  | High income | 13 | Trunk | 11H + 2I | Chronic Phase | Moderate | 51.54±12.74 | M11/F2 | N | RC |  | Neurodevelopmental therapy including mat work, strengthening, posture control, and functional activity. Sessions lasted 30 minutes. | 20 min × 3×/week | 4weeks | 20 min × 3×/week × 3.3 METs = 198 |  |
| 88 | Kwak H-D, 2024^187^ | High income | 18 | Lower limb and trunk | 16H + 18H | Chronic Phase | Moderate | 54.28±17.74 | M10/F8 | Y | VRG | ≥6mo; MMSE-K ≥24; walk >10m; no AV impairment; VR-capable. | Immersive virtual reality balance training using Oculus Quest 2 with interactive games such as tennis, bowling, and shooting. Each session lasted 30 minutes and was added to standard therapy. | 30 min × 3×/week + 30 min × 5×/week | 5weeks | Intervention Details: FIVR: 30 × 5 × 4.0 = 600 Conventional: 30 × 5 × 3.0 = 450 Total: 1050 METs-min/week | BBS |
|  |  | High income | 18 | Lower limb and trunk | 14H + 18H | Chronic Phase | Moderate | 59.17±13.86 | M11/F7 | Y | RC |  | Conventional therapy including joint mobility, mat training, and standing weight-shift tasks. Each session lasted 30 minutes. | 30 min × 3×/week + 30 min × 5×/week | 5weeks | 30 min × 5 × 3.0 = 450 METs-min/week |  |
| 90 | Dae-Hyouk Bang, 2016^188^ | High income | 6 | Lower limb and trunk | Mainly I | Chronic Phase | Moderate | 63.7±7.1 | M3/F3 | Y | MBE | First I/H stroke; hemiparesis; >6mo; walk 100m (±aid). | Combined body awareness and gait training. Sessions included 20 minutes of barefoot standing, trunk rotation, and weight shifting, followed by 30 minutes of gait rehabilitation. | 50 min/session, 5×/week | 4weeks | Intervention Details: BAT: 20 × 5 × 2.0 = 200 Walking: 30 × 5 × 2.8 = 420 Total: 620 METs-min/week | 10MWT BBS |
|  |  | High income | 6 | Lower limb and trunk | Mainly I | Chronic Phase | Moderate | 64.6±5.9 | M4/F2 | Y | GT |  | Gait training only, consisting of 30 minutes of walking at a comfortable pace. | 50 min/session, 5×/week | 4weeks | Control group 30 min × 5 × 2.8 = 420 METs-min/week |  |
| 91 | Yu-Rong Mao, 2015^189^ | Middle- and high-income | 12 | Lower limb | 11I + 1H | Subacute Phase | Moderate | 59.55±9.23 | F10/M2 | Y | GT | CT/MRI dx; first stroke; ≤3mo; gait impaired (10MWT); MMSE ≥27; MAS ≤2; stable vitals. | Bodyweight-supported treadmill training beginning with partial unloading and progressing to full weight-bearing. Walking speed gradually increased. Sessions lasted up to 40 minutes. | 30 min/session, 5×/week | 3weeks | BWSTT: 30 × 5 × 4.5 = 675 Conventional rehab: 60 × 5 × 3.0 = 1800 Total: 2475 METs-min/week | 10MWT FMA-LE |
|  |  | Middle- and high-income | 12 | Lower limb | 10I + 2H | Subacute Phase | Moderate | 60.82±10.70 | F9/M3 | Y | RC |  | Traditional gait training based on the Bobath method, including overground walking with step adjustment and postural control. Sessions lasted 30 minutes. | 30 min/session, 5×/week | 3weeks | Walking: 30 × 5 × 3.5 = 525 Conventional rehab: 60 × 5 × 3.0 = 1800 Total: 2325 METs-min/week |  |
| 93 | Jiyeon Lee, 2024^190^ | High income | 10 | Trunk | 5I + 5H | Chronic Phase | Moderate | 50.83±5.60 | M8/F2 | Y | RT | CT/MRI dx; ≥3mo; walk ≥3m (±aid); K-MMSE ≥24. | Chest resistance training involving therapist-applied pressure during deep inhalation. Followed by standard neurorehabilitation. Each breathing session lasted 15 minutes. | 15 + 40 min/session, 3 + 5×/week | 8weeks | Resistance: 15 × 3 × 2.5 = 112.5 Conventional rehab: 45 × 5 × 3.0 = 675 Total: 787.5 METs-min/week | 6MWT 10MWT |
|  |  | High income | 10 | Trunk | 8I + 2H | Chronic Phase | Moderate | 53.16±11.07 | M8/F2 | Y | FT |  | Respiratory muscle stretching using passive hold-relax techniques targeting specific muscles. Also included standard neurorehabilitation. | 15 + 40 min/session, 3 + 5×/week | 8weeks | Stretching: 15 × 3 × 2.0 = 90 Conventional rehab: 45 × 5 × 3.0 = 675 Total: 765 METs-min/week |  |
|  |  | High income | 8 | Trunk | 5I + 5H | Chronic Phase | Moderate | 58.85±5.63 | M6/F4 | N | RC |  | Only routine rehabilitation including flexibility, strength, balance, and gait training. Sessions lasted 40 minutes. | 15 + 40 min/session, 3 + 5×/week | 8weeks | 40 × 5 × 3.0 = 600 Total: 600 METs-min/week |  |
| 94 | Chang-Beom Kim, 2015^191^ | High income | 20 | Trunk | 14I + 4H | Chronic Phase | Mild to moderate | 57.3±5.1 | M10/F10 | Y | RT | CT/MRI dx; ≥6mo; MMSE ≥24; walk >10m & 6MWT; no prior resp rehab. | Chest expansion training using proprioceptive neuromuscular facilitation in side-lying position. Sessions included therapist-assisted resistance breathing for 20 minutes, followed by rehabilitation. | 20 + 40 min/session, 5×/week | 4weeks | Breathing: 20 × 5 × 2.5 = 250 Conventional rehab: 40 × 5 × 3.0 = 450 Total: 700 METs-min/week | 6MWT 10MWT |
|  |  | High income | 20 | Trunk | 13I + 7H | Chronic Phase | Mild to moderate | 56.9±4.3 | M11/F9 | Y | FT |  | Standard rehabilitation only, including ROM, strength, gait, and balance exercises. | 40 min/session, 5×/week | 4weeks | 20 × 5 × 1.8 = 180 Conventional rehab: 40 × 5 × 3.0 = 450 Total: 630 METs-min/week |  |
| 95 | Qurat-Ul-Ain Ilyas, 2018^192^ | Low- and middle-income | 15 | Lower limb and trunk | 66.7%I + 33.3%H | Subacute and chronic | Moderate | 52.53±12.76 | M8/F7 | N | GT | Stroke (clinical + CT/MRI); 30–70y; stand ≥10s; no severe cognitive, spasticity, synergy, or contracture. | Circuit gait training using 10 stations focused on balance, mobility, and lower limb control. Sessions lasted 30–40 minutes. | 45 min/session, 3.5×/week | 6weeks | 45 × 3.5 × 4.5 = 708.75 Total: 708.75 METs-min/week | BBS |
|  |  | Low- and middle-income | 15 | Lower limb and trunk | 66.7%I + 33.3%H | Subacute and chronic | Moderate | 57.68±10.92 | M8/F7 | N | RC |  | Traditional gait training with therapist-guided repetitive gait and balance tasks. Sessions matched the circuit training duration. | 45 min/session, 3.5×/week | 6weeks | 45 × 3.5 × 2.8 = 441 Total: 441 METs-min/week |  |
| 96 | Melanie K. Fleming, 2015^193^ | High income | 16 | Upper limb function | 13I + 3H | Chronic Phase | Moderate | 62.3±9.8 | M13/F3 | N | ESX | CT/MRI dx; first stroke, ≥3mo; UL dysfunction; MAS ≤3; no severe cognitive/aphasia. | Somatosensory stimulation for the upper limb nerves using electrical input for 2 hours, followed by 30 minutes of task-specific training based on ARAT. | 150 min/session, 3×/week | 4weeks | Sensory: 120 × 3 × 1.8 = 648 Task: 30 × 3 × 3.0 = 270 Total: 918 METs-min/week | FMA-LE |
|  |  | High income | 17 | Upper limb function | 14I + 3H | Chronic Phase | Moderate | 60.6±13.4 | M7/F10 | N | TOT |  | Same protocol with sham stimulation (device active but N current), followed by identical task-specific training. | 150 min/session, 3×/week | 4weeks | Sham: 120 × 3 × 1.0 = 360 Task: 30 × 3 × 3.0 = 270 Total: 630 METs-min/week |  |
| 97 | Kyoung Kim, 2015^194^ | High income | 10 | Lower limb and trunk | Mixed | Chronic Phase | Moderate | 65.9±6.2 | M5/F5 | Y | WA | CT/MRI dx; ≥6mo; MMSE ≥24; walk ≥10m; no visual/field/ortho gait issues. | NDT followed by underwater PNF coordination exercises performed in 32–34°C water. Training used alternating upper and lower limb patterns such as “sprinter” and “skater.” | 60 min/session (30+30), 5×/week | 6weeks | PNF water training: 30 × 5 × 5.3 = 795 Conventional: 30 × 5 × 3.0 = 450 Total: 1245 METs-min/week | 10MWT BBS |
|  |  | High income | 10 | Lower limb and trunk | Mixed | Chronic Phase | Moderate | 64.1±3.6 | M5/F5 | Y | NPF |  | Land-based NDT including mat training, resistance exercises, posture control, and functional tasks. | 30 min/session, 5×/week | 6weeks | 30 × 5 × 3.0 = 450 Total: 450 METs-min/week |  |
| 101 | Ji Won Shin, 2016^195^ | High income | 15 | Trunk | 13I + 2H | Chronic Phase | Moderate | 60.0±8.4 | M7/F8 | Y | ULT | CT/MRI dx; ≥6mo; K-MMSE ≥24; stand independently; no high-risk cardiac/medical/MSK issues. | Bilateral upper limb training in a standing position, including symmetrical reaching, diagonal extension, and resisted pushing, performed with hands clasped. | 30 min/session, 3×/week | 4weeks | Training: 30 × 3 × 3.5 = 315 Conventional rehab: 30 × 3 × 3.0 = 270 Total: 585 METs-min/week | 6MWT 10MWT BBS |
|  |  | High income | 15 | Trunk | 12I + 3H | Chronic Phase | Moderate | 57.4±10.3 | M9/F6 | N | RC |  | Conventional rehabilitation focused on gait, joint mobility, core stability, and postural control, without specific upper limb training. | 30 min/session, 3×/week | 4weeks | Conventional rehab only: 30 × 3 × 3.0 = 270 Total: 270 METs-min/week |  |
| 103 | Junekyung Lee, 2023^196^ | High income | 14 | Lower limb and trunk | 9I + 4H | Chronic Phase | Moderate | 65.15±5.34 | M9/F4 | N | WA | CT/MRI I/H stroke; ≥6mo; FAC ≥3; independent walking; 18–74y. | Gait training on natural beach terrain with shallow water. Tasks included walking in straight lines, S-shaped patterns, and side-stepping with warm-up and cool-down. | 30 min/session, 2×/day, 5×/week | 5days | 30min × 2/day × 5day/week × 5.0 METs = 300min × 5.0 METs Total: = 1500 METs-min/week | 10MWT BBS |
|  |  | High income | 14 | Lower limb and trunk | 10I + 5H | Chronic Phase | Moderate | 67.07±5.82 | M13/F2 | N | GT |  | Same gait protocol conducted on firm indoor flooring under controlled environmental conditions. | 30 min/session, 2×/day, 5×/week | 5days | Training: 30 × 2 × 5 × 2.8 = 840 Total: 840 METs-min/week |  |
| 104 | Rodeline Telfils, 2023^197^ | High income | 42 | Whole-body function | 31I + 11H | Subacute Phase | Mild | 62.2±13.6 | M28/F14 | N | AE | CT/MRI I/H stroke; <6mo; FAC ≥2; French health insurance enrolled. | Home-based personalized training including walking, core strengthening, and functional tasks. Activity was monitored and supported by remote and in-person follow-up. | Free activity, monitored ≥5 days/week | 6months | Walking: 3.5 × 22 × 7 = 539 Total: 539 METs-min/week | 6MWT |
|  |  | High income | 41 | Whole-body function | 31I + 10H | Subacute Phase | Mild | 62.2±13.6 | M28/F13 | N | NE |  | Standard outpatient rehabilitation without personalized exercise planning or monitoring. | Nne | 6months | Total: 0 METs-min/week |  |
| 106 | Sivertsen M, 2022^198^ | High income | 25 | Trunk | 24I + 1H | Subacute Phase | Moderate | 72.96±10.41 | M12/F13 | Y | CST | CT/MRI I/H stroke; <12wks; TIS-modNV <15; sit ≥10s; pre-stroke mRS ≤3. | I-CoreDIST therapy combining trunk control, dual-task activities, and sensory feedback. Movements covered multiple positions and progressive difficulty levels. | 60 min/session Inpatient: 5–6×/week Outpatient: 3×/week | 12weeks | I-CoreDIST: 60 × ~3.5× × 4.0 = ~570 Conventional rehab: 30 × 5 × 3.0 = 450 Total: 570 + 450 = 1020 METs-min/week | 10MWT |
|  |  | High income | 30 | Trunk | 26I + 4H | Subacute Phase | Moderate | 69.32±10.63 | M23/F7 | Y | RC |  | Conventional physiotherapy based on clinical judgment, including gait, balance, and functional training without standardized protocols. | 60 min/session Inpatient: 5–6×/week Outpatient: 3×/week | 12weeks | Usual care: 60 × ~3.5× × 3.5 = ~446 Conventional rehab: 30 × 5 × 3.0 = 450 Total: 446 + 450 = 896 METs-min/week |  |
| 107 | Katsuhiko Takatori, 2012^199^ | High income | 22 | Whole-body function | 11I + 11H | Chronic Phase | Mild to moderate | 66.0±6.9 | M15/F7 | N | FT | CT/MRI dx; ≥1yr; MMSE ≥24; walk ≥10m (±aid). | Intensive rehabilitation including resistance exercises, dynamic standing balance training, and aerobic treadmill walking, supervised individually. | 120 min/session, 2×/week | 12weeks | 120 min × 2 sessions/week × 4.5 METs = 1080 METs-min/week | 10MWT |
|  |  | High income | 22 | Whole-body function | 14I + 8H | Chronic Phase | Mild to moderate | 71.1±10.1 | M17/F5 | N | RC |  | Standard therapy including stretching, gait practice, and daily activity training. | 120 min/session, 2×/week | 12weeks | 40 min × 2 sessions/week × 3.0 METs = 240 METs-min/week |  |
| 108 | Myunggi Cho, 2024^200^ | High income | 8 | Lower limb and trunk | Mixed | Chronic Phase | Moderate | 62.63±8.47 | M3/F5 | Y | RT | DiagNsed by CT or MRI as I or H stroke; duration ≥ 12 months; K-TIS ≤ 19; MMSE-K ≥ 21; MAS < 2. | Lower limb resistance exercises combined with abdominal draw-in maneuver using pressure feedback to enhance core stability and balance. | 30 min/session, 3×/week | 4weeks | 30 min × 3 sessions/week × 4.0 METs + 30 min × 3 × 3.0 METs = 360 + 270 = 630 METs-min/week | BBS |
|  |  | High income | 8 | Lower limb and trunk | Mixed | Chronic Phase | Moderate | 67.5±15.05 | M3/F5 | Y | LLT |  | Same lower limb resistance exercises without core activation. Movements targeted hip control. | 30 min/session, 3×/week | 4weeks | 30 min × 3 × 2.5 METs + 30 min × 3 × 3.0 METs = 225 + 270 = 495 METs-min/week |  |
|  |  | High income | 8 | Lower limb and trunk | Mixed | Chronic Phase | Moderate | 60.13±5.46 | M5/F3 | Y | RC |  | Conventional rehab involving trunk rotation and lateral loading without resistance training. | 30 min/session, 3×/week | 4weeks | 30 min × 3 × 2.5 METs + 30 min × 3 × 3.0 METs = 225 + 270 = 495 METs-min/week |  |
| 109 | Hatice İkizler May, 2020^201^ | Middle- and high-income | 21 | Lower limb and trunk | 90.5%I + 9.5%H | Acute and subacute | Moderate | 57.2±7.6 | M15/F6 | Y | MBE | WHO criteria + CT/MRI I/H stroke; Brunnstrom 1–4; pre-stroke independent walking. | Mirror therapy for the lower limb using a vertical mirror to reflect Nn-paretic leg movements during ankle exercises. | 30 min (MT) + 60–120 min (rehab)/session,5×/week | 4weeks | Mirror Therapy: 30 minutes × 5 sessions/week × 2.0 METs = 300 METs-min/week Conventional Rehabilitation: 90 minutes × 5 sessions/week × 3.0 METs = 1350 METs-min/week Total METs-min/week (MT Group): 300 + 1350 = 1650 METs-min/week | 6MWT BBS |
|  |  | Middle- and high-income | 21 | Lower limb and trunk | 76.2%I + 23.8%H | Acute and subacute | Moderate | 58.8±9.8 | M10/F11 | Y | NPF |  | Standard neurorehabilitation involving sensorimotor retraining, gait, and balance exercises. | 60–120 min (rehab)/session, 5×/week | 4weeks | Conventional Rehabilitation Only: 90 minutes × 5 sessions/week × 3.0 METs = 1350 METs-min/week Total METs-min/week (Control Group):= 1350 METs-min/week |  |
| 110 | Nigar Gurbuz, 2016^202^ | Middle- and high-income | 16 | Upper limb | 15I + 1H | Subacute Phase | Moderate to severe | 60.9±10.9 | M10/F6 | Y | MBE | CT/MRI I/H stroke; <6mo; Brunnstrom 1–4 (UL); MMSE ≥24; MAS ≤2. | Mirror therapy for the upper limb with wrist and finger exercises performed while viewing the Nn-affected side in a mirror. | 20 min (MT) + 60–120 min (conventional rehab)/session, 5×/week | 4weeks | Conventional Rehabilitation: 450 minutes/week × 3.5 METs = 1575 METs-min/week Mirror Therapy: 100 minutes/week × 2.5 METs = 250 METs-min/week Total METs-min/week (True Mirror Group): 1575 + 250 = 1825 METs-min/week | FMA-UE |
|  |  | Middle- and high-income | 15 | Upper limb | 10I + 5H | Subacute Phase | Moderate to severe | 60.8±20.0 | M7/F8 | Y | NPF |  | Same upper limb therapy provided without use of a mirror, based on conventional neurorehabilitation techniques. | 60–120 min (conventional rehab)/session, 5×/week | 4weeks | Conventional Rehabilitation: 450 minutes/week × 3.5 METs = 1575 METs-min/week 2. Sham Mirror Therapy (Repetitive movements without visual feedback): 100 minutes/week × 2.0 METs = 200 METs-min/week Total METs-min/week (Sham Mirror Group): 1575 + 200 = 1775 METs-min/week |  |
| 112 | Dae-Hyouk Bang, 2016^203^ | High income | 10 | Upper limb | 7I + 3H | Subacute Phase | Moderate | 61.34±4.82 | M6/F4 | N | MBE | CT/MRI I/H stroke; 1–6mo; MMSE ≥24; hand ext MCP/IP ≥10°, wrist ≥10°; MAS ≤3; MAL-AOU <2.5. | Modified constraint-induced movement therapy with auditory feedback to limit trunk compensation during repetitive task-based upper limb activities. | 60 min/session, 5×/week | 4weeks | Task-Oriented mCIMT with Auditory Feedback: 300 minutes/week × 3.5 METs (upregulated due to cognitive load and posture control) = 1050 METs-min/week | FMA-UE |
|  |  | High income | 10 | Upper limb | 6I + 4H | Subacute Phase | Moderate | 58.22±5.17 | M5/F5 | N | CIMT |  | Same mCIMT protocol without auditory feedback, including task practice and behavioral reinforcement. | 60 min/session, 5×/week | 4weeks | Conventional mCIMT (Task-Oriented Upper Limb Training): 300 minutes/week × 3.0 METs = 900 METs-min/week |  |
| 113 | Peng Li, 2023^204^ | Middle- and high-income | 52 | Whole-body function | 58%I + 42%H | Subacute Phase | Moderate | 61.14±8.45 | M24/F26 | Y | ESX | Meets Chinese stroke diagNsis guideline; confirmed by CT/MRI; MMSE ≥ 24; unilateral fine motor impairment of the hand. | Functional electrical stimulation applied to multiple lower limb muscles using fixed pulse settings and electrode placement. | 20 min/session, 1×/day | 4weeks | Conventional Rehab: 60 min/day × 5 days/week × 3.0 METs = 900 METs-min/week Additional Training: 50 min/day × 5 days/week × 2.0 METs = 500 METs-min/week Totalweekly METs: 900 + 500 = 1400 METs-min/week | BBS FMA |
|  |  | Middle- and high-income | 50 | Whole-body function | 61.5%I + 38.5%H | Subacute Phase | Moderate | 62.06±9.11 | M28/F24 | Y | MBE |  | Functional electrical stimulation followed by mirror visual feedback training for upper limb fine motor tasks using reflected movements. | 30 min/session, 1×/day | 4weeks | Conventional Rehab: 60 min/day × 5 days/week × 3.0 METs = 900 METs-min/week Additional Training: 20 min/day × 5 days/week × 2.0 METs = 200 METs-min/week Totalweekly METs: 900 + 200 = 1100 METs-min/week |  |
| 114 | Asmaa Kamal Lasheen, 2022^205^ | Middle income | 40 | Lower limb and trunk | Mixed | Subacute Phase | Moderate | 30-60 | M29/F11 | Y | RC | Meets WHO stroke definition; confirmed by CT or MRI. | Routine hospital care involving general health monitoring without structured rehabilitation or exercise protocols. | 60 min/session, 5×/week | 2weeks | Conventional Rehabilitation Only: 60 min/day × 5 days/week × 3.0 METs = 900 METs-min/week Totalweekly METs (Control Group): = 900 METs-min/week | BBS |
|  |  | Middle income | 40 | Lower limb and trunk | Mixed | Subacute Phase | Moderate | 30-60 | M25/F15 | Y | TOT |  | Strength and task-oriented lower limb exercises, with progressive activities targeting functional recovery. | 30–45 min/session, 4×/week | 2weeks | Resistance Training: 18.75 min × 4 sessions × 4.0 METs = 300 METs-min/week Task-Oriented Training: 18.75 min × 4 sessions × 4.5 METs = 337.5 METs-min/week Total for Specialized Training: = 300 + 337.5 = 675 METs-min/week Conventional Rehab: 60 min × 5 days/week × 3.0 METs = 900 METs-min/week Totalweekly METs: 675 + 900 = 1575 METs-min/week |  |
| 115 | Areerat Suputtitada, 2004^206^ | Middle income | 15 | Lower limb and trunk | Mixed | Chronic Phase | Moderate | 61.01±10.21 | M10/F5 | Y | RC | Confirmed by CT/MRI; stroke duration ≥ 6 months; gait disorder present; able to stand independently without assistance. | Conventional gait and physiotherapy including balance training and lower limb stretching without use of treadmill or support devices. | 30 min/session, 3×/week | 6weeks | Ground Gait Training: 30 min × 5 sessions × 2.8 METs = 420 METs-min/week Conventional Rehab: 60 min × 5 days × 3.0 METs = 900 METs-min/week Totalweekly METs: 420 + 900 = 1320 METs-min/week | 10MWT BBS |
|  |  | Middle income | 15 | Lower limb and trunk | Mixed | Chronic Phase | Moderate | 64.88±10.72 | M10/F5 | Y | GT |  | Treadmill-based gait training with partial bodyweight support, gradually reduced to promote independence. | 30 min/session, 3×/week | 6weeks | PBWSTT Training: 30 min × 5 sessions × 3.0 METs = 450 METs-min/week Conventional Rehab: 60 min × 5 days × 3.0 METs = 900 METs-min/week Totalweekly METs: 450 + 900 = 1350 METs-min/week |  |
| 116 | Birol Önal，2022^207^ | Middle- and high-income | 15 | Lower limb and trunk | 67%I + 33%H | Chronic Phase | Moderate | 60±9 | M9/F6 | Y | VT | N explicit diagNstic method reported; all patients received rehabilitation at Kırıkkale University Physical Therapy Hospital. | Local vibration therapy applied to the sole of the foot combined with conventional physiotherapy involving NDT and PNF methods. | LVT: 15 min/session, 3×/week | 4weeks | Vibration Training: 15 min × 3 sessions × 1.5 METs = 67.5 METs-min/week 2. Conventional Rehab: (45 min × 3 days + 60 min × 2 days) = 255 min × 3.0 METs = 765 METs-min/week Totalweekly METs: 67.5 + 765 = 832.5 METs-min/week | 10MWT BBS |
|  |  | Middle- and high-income | 15 | Lower limb and trunk | 47%I + 53%H | Chronic Phase | Moderate | 59±9 | M11/F4 | Y | NPF |  | Same physiotherapy provided without the vibration component. | CPT: 60 min/session, 5×/week | 4weeks | Conventional Rehab Only: 60 min × 5 days × 3.0 METs = 900 METs-min/week |  |
| 117 | Tae-sung In, 2021^208^ | High income | 20 | Lower limb | Nt reported | Chronic Phase | Moderate to severe | 56.15±10.39 | M10/F10 | Y | TCMEX | Unilateral hemiplegia; communicate; walk 10m; Brunnstrom 3; CSS ≥10 (ankle spasticity). | Sit-to-stand training combined with elastic taping applied to the anterior leg muscles and ankle, performed without arm support. | 30 min/session, 5×/week | 6weeks | Sit-to-Stand: 30 min × 5 days × 4.0 METs = 600 METs-min/week Conventional Rehab: 60 min × 5 days × 3.0 METs = 900 METs-min/week Totalweekly METs (Both Arms): 600 + 900 = 1500 METs-min/week | 10MWT |
|  |  | High income | 20 | Lower limb | Nt reported | Chronic Phase | Moderate to severe | 55.05±9.88 | M11/F9 | Y | BT |  | Identical sit-to-stand training performed without taping intervention. | 30 min/session, 5×/week | 6weeks | Sit-to-Stand: 30 min × 5 days × 4.0 METs = 600 METs-min/week Conventional Rehab: 60 min × 5 days × 3.0 METs = 900 METs-min/week Totalweekly METs (Both Arms): 600 + 900 = 1500 METs-min/week |  |
| 118 | S Amala, 2024^209^ | Middle-income | 33 | Lower limb and trunk | Mixed | Acute Phase | Moderate | 53.8±6.7 | M20/F13 | Y | BT | ACA/MCA stroke; 35–60y; MMSE ≥23; supine→sitting; TIS ≥23. | Sit-to-stand training with progression from supported to unsupported seating, including feedback, along with standard physiotherapy. | 45 min/session, 5×/week | 1weeks | Sit-to-Stand Training: 22.5 min × 5 days × 4.0 METs = 450 METs-min/week Conventional Rehab: 22.5 min × 5 days × 3.0 METs = 337.5 METs-min/week Totalweekly METs: 450 + 337.5 = 787.5 METs-min/week | BBS |
|  |  | Middle-income | 33 | Lower limb and trunk | Mixed | Acute Phase | Moderate | 53.8±6.7 | M18/F15 | Y | RC |  | Conventional therapy only, focusing on balance, postural transitions, and early mobility tasks. | 45 min/session, 5×/week | 1weeks | Conventional Rehab Only: 45 min × 5 days × 3.0 METs = 675 METs-min/week |  |
| 119 | YoungJun Ko, 2015^210^ | High-income | 26 | Whole-body function | 16I + 10 H | Acute Phase | Moderate | 48.1±4.4 | 20M/6F | Y | VRG | 18–65y; stroke <6mo; walk 10m (±aid); no LMN/ortho; MMSE ≥24; read at 60cm. | Space Balance 3D system training with horizontal, vertical, and circular balance tasks and visual feedback, combined with standard rehabilitation. | 30 min/session, 5×/week | 3weeks | Space Balance 3D Training: 30 min × 5 sessions × 6.0 METs = 900 METs-min/week Conventional Rehab: 60 min × 5 sessions × 4.0 METs = 1200 METs-min/week Totalweekly METs: 900 + 1200 = 2100 METs-min/week | BBS |
|  |  | High-income | 26 | Whole-body function | 14I + 12H | Acute Phase | Moderate | 45.3±4.2 | 16M/10F | Y | NPF |  | Traditional balance rehabilitation including posture control and proprioceptive exercises without device assistance. | 30 min/session, 5×/week | 3weeks | Conventional Rehab: 60 min × 5 sessions × 4.0 METs = 1200 METs-min/week |  |
| 120 | Priti Nisheet Agni, 2017^211^ | Moderate | 15 | Upper limb | I | Subacute Phase | Mild | 66.92±12.38 | 9M/3F | N | RT | 30–75y; stroke 3w–6mo; Brunnstrom ≥2; follow commands; shoulder/elbow/wrist/finger movements. | Open-chain resistance training for the upper limb using concentric, eccentric, and isometric exercises with adjustable loads. | 70 min/session, 4×/week | 6weeks | Resistance + Routine Resistance: 40 min × 4 times/week × 3.5 METs = 560 METs-min/week Routine: 30 min × 4 times/week × 3.0 METs = 360 METs-min/week Total = 560 + 360 = 920 METs-min/week | FMA-UE |
|  |  | Moderate | 15 | Upper limb | I | Subacute Phase | Mild | 70.46±14.32 | 10M/3F | N | TOT |  | Task-specific upper limb training involving daily activities with graded progression and task decomposition. | 70 min/session, 4×/week | 6weeks | Functional + Routine Functional: 40 min × 4 times/week × 2.8 METs = 448 METs-min/week Routine: 30 min × 4 times/week × 3.0 METs = 360 METs-min/week Total = 448 + 360 = 808 METs-min/week |  |
|  |  | Moderate | 15 | Upper limb | I | Subacute Phase | Mild | 69.67±14.14 | 10M/2F | N | FT |  | Combined protocol with equal durations of strength and functional task training. | 70 min/session, 4×/week | 6weeks | Resistance + Functional Resistance: 40 min × 4 times/week × 3.5 METs = 560 METs-min/week Functional: 40 min × 4 times/week × 2.8 METs = 448 METs-min/week Total = 560 + 448 = 1008 METs-min/week |  |
| 121 | Nadia Gul, 2021^212^ | Low- and middle-income | 20 | Whole-body function | Mixed | Subacute and chronic | Mild to moderate | Nt reported | 13M/7F | Y | CST | DiagNsed with stroke; impaired balance and postural control; able to participate in training. | Swiss ball training including pelvic tilting, weight shifting, limb coordination, and supine lower limb exercises. Therapist guidance was provided initially. | 60 min/session, 4×/week | 3weeks | Swiss Ball Group Swiss Ball: 40 min × 4 times/week × 3.5 METs = 560 METs-min/week Routine: 20 min × 4 times/week × 4.0 METs = 320 METs-min/week Total = 560 + 320 = 880 METs-min/week | BBS |
|  |  | Low- and middle-income | 20 | Whole-body function | Mixed | Subacute and chronic | Mild to moderate | Nt reported | 11M/9F | Y | RC |  | Conventional rehabilitation targeting the affected muscle groups, with N additional equipment-based tasks described. | 60 min/session, 4×/week | 3weeks | Control Group Routine: 60 min × 4 times/week × 4.0 METs = 960 METs-min/week |  |
| 123 | Shanta Pandian, 2015^213^ | Moderate | 17 | Whole-body function | 6I + 11H | Chronic Phase | Moderate to severe | 45.53±11.74 | 9M/8F | Y | RT | Age 50–70; I or H stroke ≥ 24weeks post-onset; unilateral hemiplegia; FAC ≥ 2. | Progressive resistance training (PRE) targeting major muscle groups of the shoulder, elbow, forearm, wrist, hip, knee, and ankle. Initial load set at 50% of 10RM and gradually increased to 100%. | 60 min/session, 3×/week | 8weeks | PRE + BT + Routine PRE (Resistance training):  30 min × 3 times/week × 4.0 METs = 360 METs-min/week BT (Bilateral task training):  30 min × 3 times/week × 3.5 METs = 315 METs-min/week Routine rehabilitation:  30 min × 5 times/week × 3.0 METs = 450 METs-min/week Total = 360 + 315 + 450 = 1125 METs-min/week | FMA-LE FMA-UE |
|  |  | Moderate | 18 | Whole-body function | 10I + 8H | Chronic Phase | Moderate to severe | 40.72±11.88 | 10M/8F | Y | CIMT |  | Neurophysiological therapy based on Brunnstrom approach, emphasizing reflex patterns and synergistic movements on the affected side. | 60 min/session, 3×/week | 8weeks | Brunnstrom + Routine Brunnstrom therapy:  60 min × 3 times/week × 3.5 METs = 630 METs-min/week Routine rehabilitation:  30 min × 5 times/week × 3.0 METs = 450 METs-min/week Total = 630 + 450 = 1080 METs-min/week |  |
| 126 | Sae Hoon Chung, 2019^214^ | High income | 18 | Lower limb | 77.8%I + 22.2%H | Subacute Phase | Moderate | 63.1±11.9 | 13M/5F | Y | TOT | Age ≥ 20; stroke duration ≤ 6 months; BBS score 21–40; N stroke history; N severe musculoskeletal or cardiovascular disease. | Task-specific lower extremity training using the 3DBT-12 device, with real-time feedback on bodyweight shifting and knee flexion-extension. Included gamified tasks such as virtual fruit-picking. | 30 min/session, 5×/week；60 min/session, 5×/week | 3weeks | TSLET Group TSLET:  30 min × 5 times/week × 5.0 METs = 750 METs-min/week Routine:  30 min × 5 times/week × 4.0 METs = 600 METs-min/week Total = 750 + 600 = 1350 METs-min/week | 6MWT 10MWT BBS |
|  |  | High income | 17 | Lower limb | 70.6%I + 29.4%H | Subacute Phase | Moderate | 62.5±11.3 | 13M/4F | Y | NPF |  | Conventional physiotherapy including trunk stability, balance board, and proprioceptive training. | 30 min/session, 5×/week；60 min/session, 5×/week | 3weeks | Routine rehabilitation only: 60 min × 5 times/week × 4.0 METs = 1200 METs-min/week |  |
| 127 | Ki-Tae Park, 2016^215^ | High income | 6 | Lower limb | Nt reported | Chronic Phase | Mild to moderate | Nt reported | Nt reported | Y | TOT | Stroke ≥ 6 months ago; able to walk ≥ 40 meters independently; able to cross obstacles; able to understand study purpose and give consent; MAS ≤ 2. | Obstacle circuit training consisting of five continuous walking tasks: straight walk, S-walk, stair climbing, inclined walking, and step-over tasks of various heights. Supervised by a therapist. | 30 min/session, 4×/week | 3weeks | Obstacle Circuit Group Obstacle Circuit:  30 min × 4 times/week × 5.5 METs = 660 METs-min/week Routine:  30 min × 4 times/week × 4.0 METs = 480 METs-min/week Total = 660 + 480 = 1140 METs-min/week | 10MWT BBS |
|  |  | High income | 6 | Lower limb | Nt reported | Chronic Phase | Mild to moderate | Nt reported | Nt reported | Y | GT |  | Flat-ground walking training without any obstacles, performed under therapist supervision. | 30 min/session, 4×/week | 3weeks | Flat Gait Group Gait training:  30 min × 4 times/week × 3.5 METs = 420 METs-min/week Routine:  30 min × 4 times/week × 4.0 METs = 480 METs-min/week Total = 420 + 480 = 900 METs-min/week |  |
| 128 | Jungeun Yi, 2024^216^ | High income | 30 | Whole-body function | Nt reported | Chronic Phase | Mild | 71.77±6.58 | 6M/24F | Y | MBE | Age ≥ 60; resident of Incheon, South Korea; able to walk independently without wheelchair or walker. | Remote feedback-based intervention (SHe CoFFEE) using a tablet for real-time monitoring and reminders. Included self-monitoring of physical activity, fall prevention exercises, and group/individual online workouts. | 30–40 min/session, 5×/week | 8weeks | Remote group training:  3 sessions/week × 30 min/session × 4.0 METs = 360 METs-min/week Individual walking:  5 sessions/week × 40 min/session × 3.5 METs = 700 METs-min/week Video-based training:  2 sessions/week × 30 min/session × 3.0 METs = 180 METs-min/week Total = 360 + 700 + 180 = 1240 METs-min/week | 10MWT |
|  |  | High income | 30 | Whole-body function | Nt reported | Chronic Phase | Mild | 70.83±6.58 | 7M/23F | N | NE |  | Waitlist group receiving the same SHe CoFFEE intervention after 8weeks. | 0 | 0weeks | 0METs-min/week |  |
| 129 | Sung-Jun Moon & Tae-Ho Kim, 2017^217^ | High income | 12 | Trunk function | 7I 7，5H | Chronic Phase | Mild to moderate | 54.58±11.77 | 7M/5F | Y | VRG | Stroke > 6 months; able to walk independently; K-MMSE ≥ 24; voluntary study participation. | Spine Balance 3D training for trunk stability using a platform capable of 5°–60° tilting in eight directions. Participants were challenged to maintain upright posture during tilt. | 30 min/session, 3×/week | 7weeks | Spine Balance 3D Group Spine Balance 3D:  30 min × 3 times/week × 4.5 METs = 405 METs-min/week Routine rehab (CNS PT):  30 min × 3 times/week × 3.0 METs = 270 METs-min/week Total = 405 + 270 = 675 METs-min/week | 10MWT |
|  |  | High income | 12 | Trunk function | 7I + 5H | Chronic Phase | Mild to moderate | 53.67±9.12 | 9M/3F | Y | CST |  | Bridge exercises in various postures: supine, prone, side-lying, quadruped, and unstable surface variations. | 30 min/session, 3×/week | 7weeks | Bridge Training Group Bridge Training:  30 min × 3 times/week × 3.5 METs = 315 METs-min/week Routine rehab (CNS PT):  30 min × 3 times/week × 3.0 METs = 270 METs-min/week Total = 315 + 270 = 585 METs-min/week |  |
| 130 | AlaNud O. Almasoudi, 2024^218^ | High income | 23 | Lower limb | 14I + 4H | Subacute Phase | Moderate | Nt reported | 13M/5F | Y | CST | Age 28–86; H or I stroke < 6 months; able to walk 10 meters independently; MMSE ≥ 24; Trunk Impairment Scale < 21. | Selective trunk and hip strategy training including supine/sitting flexion-extension, pelvic bridge, standing reaches, and single-leg stance on unstable surfaces. | 30 min/session, 4×/week | 6weeks | TSLET Group Weeks 1–3:  TSLET: 150 min/week × 4.0 METs = 600 METs-min/week  Routine: 150 min/week × 3.0 METs = 450 METs-min/week  Total = 1050 METs-min/week × 3weeks = 3150 METs-min Weeks 4–5:  Routine only: 150 min/week × 3.0 METs = 450 METs-min/week × 2weeks = 900 METs-min  Total over 5weeks = 3150 + 900 = 4050 METs-min Average perweek = 4050 ÷ 5 = 810 METs-min/week | BBS |
|  |  | High income | 23 | Lower limb | 12I + 5H | Subacute Phase | Moderate | Nt reported | 13M/4F | Y | RC |  | Standard rehabilitation including stretching, lower limb strengthening, gait training, and basic physiotherapy tasks. | 30 min/session, 4×/week | 6weeks | Weeks 1–3:  Routine: 300 min/week × 3.0 METs = 900 METs-min/week × 3 = 2700 METs-min Weeks 4–5:  Routine: 150 min/week × 3.0 METs = 450 METs-min/week × 2 = 900 METs-min Total = 2700 + 900 = 3600 METs-min Average = 3600 ÷ 5 = 720 METs-min/week |  |
| 131 | Jiyeon Lee, 2020^219^ | High income | 10 | Trunk and lower limb function | 6I + 4H | Chronic Phase | Moderate | 66.89±10.00 | M6/F4 | Y | RT | First-ever stroke; able to walk independently (assistive devices allowed); N other neurological diseases; N joint replacement; N blindness or severe visual impairment. | Core stability exercises using abdominal hollowing, aiming to activate the transversus abdominis (TrA) with inward abdominal movement and controlled breathing. | 20 min/session, 3×/week | 6weeks | Group A (Core + Routine) Core Training: 20 min × 3 times/week × 3.5 METs = 210 METs-min/week Routine Rehab: 40 min × 3 times/week × 3.0 METs = 360 METs-min/week Total: 210 + 360 = 570 METs-min/week | 10MWT BBS |
|  |  | High income | 10 | Trunk and lower limb function | 5I + 5H | Chronic Phase | Moderate | 69.57±11.75 | M3/F7 | Y | CST |  | Abdominal bracing exercises engaging global core muscles (EO, IO, RA) to increase intra-abdominal pressure and spinal stability. | 20 min/session, 3×/week | 6weeks | Group B (Same as A) Core Training: 20 min × 3 × 3.5 METs = 210 METs-min/week Routine Rehab: 40 min × 3 × 3.0 METs = 360 METs-min/week Total: 210 + 360 = 570 METs-min/week |  |
|  |  | High income | 10 | Trunk and lower limb function | 4I + 6H | Chronic Phase | Moderate | 68.57±9.54 | M6/F4 | Y | RC |  | Traditional rehabilitation including physical and occupational therapy focused on gait, balance, and functional mobility improvement. | 20 min/session, 3×/week | 6weeks | Control Group Routine Rehab Only: 60 min × 3 × 3.0 METs = 540 METs-min/week |  |
| 133 | Taisheng Feng, 2024^220^ | Middle- and high-income | 38 | Upper limb | 78.95%I + 21.05%H | Acute Phase | Moderate | 57.03±13.40 | M27/F11 | Y | RT | 4th Nat'l Conf (China) + CT/MRI dx; stroke 1–2mo; handgrip & muscle thickness testable; no severe consciousness/cognitive disorders; consented. | Unilateral resistance training using elastic bands for elbow flexion and shoulder abduction. Training targeted the Nn-paretic side to strengthen upper limb muscles. | 30 reps × 2 sets, 2×/day, 5 days/week | 4weeks | Experimental (Elastic Resistance + Routine) Routine: 300 METs-min/week Elastic Band: 20 min × 5 × 4.0 METs = 400 METs-min/week Total: 300 + 400 = 700 METs-min/week | FMA-UE |
|  |  | Middle- and high-income | 39 | Upper limb | 69.23%I + 30.77%H | Acute Phase | Moderate | 56.54±10.23 | M31/F8 | N | RC |  | Conventional rehabilitation including passive movements, bed mobility, and simple exercises for the unaffected side without added resistance. | 30 reps × 2 sets, 2×/day, 5 days/week | 4weeks | Control Group Passive exercises (20 min), 5 times/week × 3.0 METs = 300 METs-min/week |  |
| 134 | Heejoong Kim, 2019^221^ | High income | 10 | Trunk and lower limb function | 7I + 3H | Chronic Phase | Mild to moderate | 53.1±15.01 | M7/F3 | Y | WA | Walk >10m & 15min independently; visual/spatial intact; MMSE-K ≥24; no CV surgery history; consented. | Underwater gait training in a warm pool with progressive speed control using a metroNme. Included straight walking and turning. | 30 min/session, 3×/week | 8weeks | UGT (Underwater Gait Training) Water Training: 30 min × 3 × 6.0 METs = 540 METs-min/week Routine: 30 min × 5 × 3.0 METs = 450 METs-min/week Total: 990 METs-min/week | 6MWT BBS |
|  |  | High income | 9 | Trunk and lower limb function | 7I + 2H | Chronic Phase | Mild to moderate | 57.22±11.18 | M7/F2 | Y | GT |  | Land-based gait training using digital metroNme for step pacing and speed adjustment. | 30 min/session, 3×/week | 8weeks | LGT (Land Gait Training) Land Training: 30 min × 3 × 4.0 METs = 360 METs-min/week Routine: 30 min × 5 × 3.0 METs = 450 METs-min/week Total: 810 METs-min/week |  |
|  |  | High income | 9 | Trunk and lower limb function | 7I + 2H | Chronic Phase | Mild to moderate | 50.33±19.61 | M5/F4 | Y | RC |  | Conventional rehabilitation with standard gait and general physiotherapy exercises. | 30 min/session, 3×/week | 8weeks | CG (Conventional Gait) Walking: 30 min × 3 × 3.0 METs = 270 METs-min/week Routine: 30 min × 5 × 3.0 METs = 450 METs-min/week Total: 720 METs-min/week |  |
| 135 | Dae-Hyouk Bang, 2014^222^ | High income | 6 | Lower limb | Mixed | Chronic Phase | Mild to moderate | 61.1±4.8 | M4/F2 | N | CST | First I/H stroke ≥1yr; speed ≥0.5m/s; treadmill eligible; no other gait rehab; walk ≥10m; MMSE ≥21. | Treadmill walking combined with unstable surface training using balance pads. Tasks included squats, heel raises, forward reaches, and stepping in place. | 30 min (treadmill) + 10 min (UST)/session, 5×/week | 4weeks | Experimental Group (Treadmill + UST) Treadmill: 30 min × 5 × 3.5 METs = 525 METs-min/week UST (Dynamic balance): 10 min × 5 × 4.0 METs = 200 METs-min/week Total: 525 + 200 = 725 METs-min/week | 6MWT 10MWT |
|  |  | High income | 7 | Lower limb | Mixed | Chronic Phase | Mild to moderate | 58.5±3.4 | M3/F3 | N | AE |  | Treadmill walking only, without balance pad or instability components. | 30 min (treadmill) + 10 min (UST)/session, 5×/week | 4weeks | Control Group Treadmill: 30 min × 5 × 3.5 METs = 525 METs-min/week |  |
| 136 | James Faulkner, 2025^223^ | High income | 15 | Trunk and lower limb function | Mixed | Chronic Phase | Moderate | 64.7±16.4 | M11/F4 | Y | LLT | NHS stroke dx (UK); 3mo–7yr post-stroke; stable; stand/walk (±aid); FAC 2–5. | Intermittent pneumatic compression applied via GMOVE Suit during daily activities such as walking and sit-to-stand transitions. Pressure adjusted progressively. | 30 min/day | 12weeks | Experimental (Home Ex + IPC Pressotherapy) Sit-to-stand + stairs: 3.3 METs × 180 min = 594 METs-min/week IPC Pressotherapy (Passive): 2.0 METs × 180 min = 360 METs-min/week Total: 594 + 360 = 954 METs-min/week | 6MWT 10MWT BBS FMA-LE FMA-UE |
|  |  | High income | 16 | Trunk and lower limb function | Mixed | Chronic Phase | Moderate | 67.3±15.0 | M12/F4 | Y | RC |  | Encouraged to perform 30 minutes of physical activity daily as part of routine rehabilitation. | 30 min/day | 12weeks | Control Group Walking + daily activities: 3.3 METs × 210 min = 693 METs-min/week |  |
| 138 | Ilona J.M. de Rooij, 2021^224^ | High income | 28 | Lower limb | 85.7%I + 14.3%H | Subacute Phase | Mild to moderate | 65±7.2 | M18/F10 | Y | VRG | WHO stroke; 2w–6mo; FAC ≥3; limited walking ADLs; community-dwelling; 18–80y. | Virtual reality gait training using GRAIL system with dual-belt treadmill, motion capture, and immersive visual feedback. Included balance, dual-task, and adaptability tasks. | 30 min/session, 2×/week | 6weeks | VRT Group (ArduiN + Routine) VRT Training: 30 min × 2 × 4.0 METs = 240 METs-min/week Routine (PT/OT/PMT average): 60 min × 5 × 3.0 METs = 900 METs-min/week Total: 240 + 900 = 1140 METs-min/week | 6MWT |
|  |  | High income | 24 | Lower limb | 83.3%I + 16.7%H | Subacute Phase | Mild to moderate | 61±8.3 | M18/F6 | Y | GT |  | Conventional treadmill and functional gait training involving steps, object retrieval, uneven surfaces, and obstacle crossing. | 30 min/session, 2×/week | 6weeks | Nn-VRT Group (ArduiN + Routine) ArduiN Training: 30 min × 2 × 3.5 METs = 210 METs-min/week Routine: 60 min × 5 × 3.0 METs = 900 METs-min/week Total: 210 + 900 = 1110 METs-min/week |  |
| 139 | Gui bin SonG, 2015^225^ | High income | 20 | Lower limb | Mixed | Chronic Phase | Moderate | 51.37±40.6 | M10/F10 | N | VRG | Hemiplegia (stroke); no visual/vestibular/ortho issues; full ROM; follow instructions; MMSE-K ≥24. | Game-based virtual reality training using Xbox Kinect with tasks requiring balance and body movement control, including sports, walking, and stair climbing. | 30 min/session, 5×/week | 8weeks | 30 min × 5 times/week × 4.0 METs = 600 METs-min/week | 10MWT |
|  |  | High income | 20 | Lower limb | Mixed | Chronic Phase | Moderate | 50.10±7.83 | M12/F8 | N | AE |  | Stationary cycling using MOTOmed Viva 2 with biofeedback. Intensity controlled to remain within low heart rate reserve range. | 30 min/session, 5×/week | 8weeks | 30 min × 5 times/week × 3.5 METs = 525 METs-min/week |  |
| 140 | Ken-Wei Chang,2021^226^ | Middle- and high-income | 8 | Lower limb | 7H + 1I | Chronic Phase | Moderate | 52.39±6.06 | M6/F2 | Y | GT | First stroke; ≥6mo; UL hemiplegia; Brunnstrom ≥IV; walk ≥11m (±aid); stable; no visual loss; cooperative. | Backward treadmill walking at a self-selected comfortable pace. Exercise intensity maintained within low to moderate fatigue levels. | 30 min/session, 3×/week | 4weeks | Experimental (Backward walking):  Backward walk: 30 min × 3 × 2.5 METs = 225 METs-min/week  Routine: 30 min × 3 × 3.0 METs = 270 METs-min/week Total: 495 METs-min/week | 6MWT 10MWT BBS |
|  |  | Middle- and high-income | 8 | Lower limb | 3H + 5I | Chronic Phase | Moderate | 54.38±14.05 | M5/F3 | Y | RC |  | Standard physical therapy including gait, balance, and strength training. | 30 min/session, 3×/week | 4weeks | Control:  Routine: 90 min/week × 3.0 METs = 270 METs-min/week |  |
| 141 | Ning Bei, 2023^227^ | Middle- and high-income | 80 | Lower limb | 40H + 40I | Acute Phase | Moderate to severe | 62.63±6.79 | M60/F20 | Y | WA | Chinese 2018/2019 stroke dx + CT/MRI; <1mo; stable vitals; no LL pain/limitation; consented. | Hydrotherapy in two phases: initial aquatic gait, core, and resistance training with water flow and temperature control; followed by weight-supported underwater walking and dynamic balance activities. | 30–40 min/session, 6×/week | 8weeks | Aquatic Intervention Group:  Underwater walking: 35 min × 6 × 4.5 = 945 METs-min/week  ROM: 10 min × 6 × 3.0 = 180 METs-min/week  E-stim + passive: 5 min × 6 × 2.0 = 60 METs-min/week Total: 1185 METs-min/week | BBS FMA |
|  |  | Middle- and high-income | 80 | Lower limb | 40H + 40I | Acute Phase | Moderate to severe | 62.68±6.56 | M52/F28 | Y | RC |  | Conventional care combining pharmacologic treatment and standard physiotherapy, including gait and resistance exercises. | 30–40 min/session, 6×/week | 8weeks | Control Group:  Lower limb rehab: 40 min × 6 × 3.5 = 840 METs-min/week |  |
| 142 | Ilgın Sade, 2020^228^ | Middle- and high-income | 26 | Lower limb | 21I21 + 5H | Chronic Phase | Moderate | 46.8±15 | F14/M12 | Y | VT | Age 18–70; medically stable; ≥12weeks post-stroke; BBS < 40 (moderate-to-severe balance impairment). | Whole-body vibration training on Power Plate platform with alternating shallow and deep squats, performed in static standing posture. | 1 min × 4 sets/session, 5×/week | 3weeks | WBV Group:  WBV: 8 min × 5 × 5.0 = 200 METs-min/week  Routine rehab: 45 min × 5 × 3.5 = 787.5 METs-min/week Total: 987.5 METs-min/week | BBS |
|  |  | Middle- and high-income | 17 | Lower limb | 11I11 + 6H | Chronic Phase | Moderate | 51.6±10 | F9/M8 | Y | RC |  | Standard physiotherapy including gait, balance, and strength exercises, without vibration intervention. | 1 min × 4 sets/session, 5×/week | 3weeks | Control Group:  Routine rehab only: 45 min × 5 × 3.5 = 787.5 METs-min/week |  |
| 143 | Jin Wan Kim & Jong Hwa Lee, 2021^229^ | High income | 20 | Lower limb | 15I15 + 5H | Subacute Phase | Moderate | 57.2±11.0 | F12/M8 | Y | VT | First stroke; ≤2mo; gait disturbance; K-MMSE ≥10; hip/knee strength ≥3 (MRC); fit for standing/gait training. | Whole-body vibration using Sonix platform with patients performing repeated squats at fixed frequency and intensity settings. | 20 min/session, 2×/day, 5 days/week | 2weeks | WBV + Squat Group:  WBV + Squat: 200 min × 5.5 METs = 1100 METs-min/week  Routine: 200 min × 3.5 METs = 700 METs-min/week Total: 1800 METs-min/week | 10MWT BBS |
|  |  | High income | 18 | Lower limb | 10I10 + 8H | Subacute Phase | Moderate | 55.7±10.4 | F11/M7 | Y | RC |  | Same squat and balance activities as the intervention group, but performed without vibration. | 20 min/session, 2×/day, 5 days/week | 2weeks | Ground Squat Group:  Ground Squat: 200 min × 5.0 METs = 1000 METs-min/week  Routine: 200 min × 3.5 METs = 700 METs-min/week Total: 1700 METs-min/week |  |
| 144 | Pradeepa Nayak, 2025^230^ | Middle income | 42 | Whole-body function | 57.1%I + 42.9%H | Chronic Phase | Mild to moderate | 57.64±9.63 | M36/F6 | N | FT | DiagNsed from medical record; stroke duration >6 months; age 18–75; able to walk independently (assistive device allowed). | Multi-phase physical activity promotion program combining behavioral counseling, aerobic and resistance training, balance tasks, and home-based adaptive activities. | ≥30 min/session, ≥5×/week | 45days | Aerobic training: 30 min × 7 × 4.0 = 840 METs-min/week Resistance training: 30 min × 2 × 3.5 = 210 METs-min/week Balance/stability: 30 min × 3 × 3.0 = 270 METs-min/week Daily activity: 15 min × 7 × 4.5 = 472.5 METs-min/week Adaptive sports: 30 min × 3 × 4.5 = 405 METs-min/week Total: 2197.5 METs-min/week | 6MWT 10MWT |
|  |  | Middle income | 42 | Whole-body function | 69.0%I + 31.0%H | Chronic Phase | Mild to moderate | 59.33±10.43 | M30/F12 | N | NE |  | virtual reality gait training using GRAIL system with dual-belt treadmill, motion capture, and immersive visual feedback. Included balance, dual-task, and adaptability tasks. | None | 0 | 0METs-min/week |  |
| 145 | Shaomin Chen, 2021^231^ | Middle- and high-income | 59 | Lower limb | 57.6%H + 42.4%I | Subacute Phase | Moderate | 55.41±6.78 | M41/F18 | Y | FT | Clinical diagNsis of stroke with lower limb spasticity; MMSE ≥ 18. | Home-based rehabilitation program including personalized gait and lower limb strengthening exercises. Initial phase included supervised sessions with gradual transition to home-based family-assisted training. | Periodic intervention (split by months, requires special format) | 12months | IG (Home program + Hospital rehab):  Home training: 30 min × 3 × 3.5 = 315 METs-min/week  Routine: 30 min × 5 × 3.0 = 450 METs-min/week Total: 765 METs-min/week | 10MWT FMA |
|  |  | Middle- and high-income | 62 | Lower limb | 56.45%H + 43.55%I | Subacute Phase | Moderate | 56.41±6.13 | M44/F18 | Y | RC |  | Standard care with education booklet, follow-up calls, and scheduled medical evaluations, without specific exercise prescriptions. | Periodic intervention (split by months, requires special format) | 12months | Control Group:  Walking: 30 min × 3 × 3.0 = 270 METs-min/week  Routine: 30 min × 5 × 3.0 = 450 METs-min/week Total: 720 METs-min/week |  |
| 146 | A. Sulfikar Ali, 2024^232^ | Moderate | 64 | Upper limb | 82.8% I + 17.1% H | Subacute Phase | Moderate | 54.4±11.7 | M78.1%/F 21.9% | Y | VRG | NIHSS > 6; MoCA ≥ 26; Brunnstrom stage 1–5; MAS ≤ 3. | Gamified upper limb training using the ArmAble™ device. Tasks included grasping, pushing, lifting, and interactive games (e.g., mosquito swatting, fruit grabbing), followed by home-based training. | 45–60 min/session, 6×/week, plus home-based training | 6weeks | Group A:  Routine rehab: 60 min × 6 × 3.0 = 1080 METs-min/week  Game-based ArmAble: 60 min × 6 × 3.5 = 1260 METs-min/week  Home program: 30 min × 6 × 2.5 = 450 METs-min/week Total: 2790 METs-min/week | FMA-UE |
|  |  | Moderate | 56 | Upper limb | 83.9% I + 16.0% H | Subacute Phase | Moderate | 57.7±10.9 | M73.2%/F26.8% | Y | TOT |  | Conventional task-based upper limb training with repeated functional movements such as holding cups or pushing chairs, followed by home-based sessions. | 45–60 min/session, 6×/week, plus home-based training | 6weeks | Group B:  Routine rehab: 60 min × 6 × 3.0 = 1080 METs-min/week  Functional arm task: 60 min × 6 × 3.3 = 1188 METs-min/week  Home program: 30 min × 6 × 2.5 = 450 METs-min/week Total: 2718 METs-min/week |  |
| 147 | Jung-Lim Lee, 2025^233^ | High income | 19 | Trunk | Mixed | Subacute and chronic | Moderate | 63.79±11.76 | M10/F9 | Y | RAT | Stroke (I/H, MRI/CT); BBS 21–40; no LL ortho surgery; cognitively intact; follow instructions. | Balance training using the SBT-330 device with weight shifting and goal-oriented games (e.g., fruit catching, archery) performed in standing posture. | 30 min/session, 5×/week | 4weeks | SBT-330 + Routine:  SBT-330: 30 min × 5 × 3.0 = 450 METs-min/week  Routine: 30 min × 5 × 3.5 = 525 METs-min/week Total: 975 METs-min/week | BBS FMA-LE |
|  |  | High income | 14 | Trunk | Mixed | Subacute and chronic | Moderate to severe | 65.14±11.66 | M11/F3 | Y | RC |  | Traditional physiotherapy including gait training, lower limb strengthening, and functional tasks using ergometers and step exercises. | 30 min/session, 5×/week | 4weeks | Routine Only:  Routine: 60 min × 5 × 3.5 = 1050 METs-min/week |  |
| 148 | Sinikka H. Peurala, 2005^234^ | High income | 15 | Whole-body function | 9I + 6H | Chronic Phase | Moderate | 53.3±8.9 | M11/F4 | Y | ESX | MRI/CT confirmed; SSS 42.6±7; chronic hemiparetic gait (cane/orthosis dependent); cognitively intact. | Robot-assisted gait training using an electric treadmill with sling support and functional electrical stimulation (FES) applied to weak muscles. | 20 min/session, 5×/week | 3weeks | GT + FES:  20 min × 5 × 4.0 = 400 METs-min/week  Routine: 55 min × 5 × 3.0 = 825 METs-min/week Total: 1225 METs-min/week | 6MWT 10MWT |
|  |  | High income | 15 | Whole-body function | 9I + 5H | Chronic Phase | Moderate | 51.2±7.9 | M13/F1 | Y | RAT |  | Same robot-assisted gait training without electrical stimulation. | 20 min/session, 5×/week | 3weeks | GT Only:  20 min × 5 × 3.8 = 380 METs-min/week  Routine: same as above = 825 METs-min/week Total: 1205 METs-min/week |  |
|  |  | High income | 15 | Whole-body function | 7I + 9H | Chronic Phase | Moderate | 52.3±6.8 | M13/F3 | Y | GT |  | Overground gait training involving varied surfaces and speed adjustments, guided by a therapist. | 20 min/session, 5×/week | 3weeks | Ground gait training:  20 min × 5 × 3.5 = 350 METs-min/week  Routine: 55 min × 5 × 3.0 = 825 METs-min/week Total: 1175 METs-min/week |  |
| 149 | Elif Tarihci Cakmak, 2024^235^ | Middle- and high-income | 16 | Lower limb | 9I + 7H | Subacute and chronic | Moderate to severe | 55.0±6.0 | M8/F8 | Y | GT | Confirmed by MRI/CT; MMSE ≥ 20; 10MWT speed < 1.0 m/s; able to walk 10 m with assistive device or support. | Gait training on a treadmill with bodyweight support, gradually reduced from 30% to 15%, in addition to conventional rehabilitation. | 60 min/day (30 min CR + 30 min BWSTT), 5 days/week | 3weeks | BWSTT Group:  BWSTT: 30 min × 5 × 4.0 = 600 METs-min/week  Routine: 30 min × 5 × 3.0 = 450 METs-min/week Total: 1050 METs-min/week | 6MWT 10MWT |
|  |  | Middle- and high-income | 14 | Lower limb | 10I + 4H | Subacute and chronic | Moderate to severe | 57.5±5.0 | M9/F5 | N | RC |  | Conventional therapy including posture correction, stretching, assisted movement, strengthening, balance, and overground gait training. | 30 min CR 5 days/week | 3weeks | Routine Only:  30 min × 5 × 3.0 = 450 METs-min/week |  |
| 150 | Hyun-Ju Park, 2011^236^ | High income | 13 | Whole-body function | 7I + 6H | Chronic Phase | Moderate | 59.38±8.46 | M7/F6 | Y | GT | MRI/CT confirmed; MMSE ≥25; gait ≤0.7 m/s; no hearing/visual/ortho/CV gait issues. | Community-based walking training performed in real-life environments (e.g., hospital hallways, sidewalks, slopes, shopping centers), with increasing complexity. | 60 min/session, 3×/week | 4weeks | Experimental (Community walking + Bobath rehab) Community walking: 60 min × 3/week × 3.5 METs = 630 METs-min/week Bobath rehab: 60 min × 5/week × 3.0 METs = 900 METs-min/week Total: 1530 METs-min/week | 6MWT 10MWT |
|  |  | High income | 12 | Whole-body function | 8I + 4H | Chronic Phase | Moderate | 56.92±7.79 | M5/F7 | Y | NPF |  | Functional therapy based on the Bobath concept, including standing, gait patterns, balance, and stair negotiation. | 60 min/session, 3×/week | 4weeks | Control Group  Bobath rehab: 60 min × 5/week × 3.0 METs = 900 METs-min/week |  |
| 151 | Khushboo C. Valodwala, 2019^237^ | Middle income | 17 | Lower limb | Mixed | Chronic Phase | Moderate | 46-74 | M28/F23 | Y | RC | MRI/CT confirmed; Brunnstrom ≥3 (LL); MMSE ≥24; BBS 41–56; walk 10m (±aid/support). | Conventional therapy including stretching, strengthening, single-leg stance, gait and stair training. | 50 min/session, 5×/week | 4weeks | Group A (N board): 0 (passive) + 150 min × 3.5 METs = 525 METs-min/week | BBS |
|  |  | Middle income | 17 | Lower limb | Mixed | Chronic Phase | Moderate | 46-74 | M28/F23 | Y | BT |  | Balance training using a wobble board to practice control in forward-backward, lateral, and rotational directions. | 50 min/session, 5×/week | 4weeks | Group B (Board, 4.0 METs for 100 min):  100 min × 4.0 = 400 + 150 min × 3.5 = 525 Total: 925 METs-min/week |  |
|  |  | Middle income | 17 | Lower limb | Mixed | Chronic Phase | Moderate | 46-74 | M28/F23 | Y | MBE |  | Same wobble board training enhanced with real-time visual feedback via Sensamove Miniboard. | 50 min/session, 5×/week | 4weeks | Group C (Board, 4.5 METs for 100 min):  100 min × 4.5 = 450 + 150 min × 3.5 = 525 Total: 975 METs-min/week |  |
| 152 | Li Zhang, 2024^238^ | Middle- and high-income | 31 | Lower limb | Mixed | Subacute Phase | Moderate | 52.00±11.83 | M25/F6 | Y | CST | Confirmed by MRI/CT; FMA-LE ≤ 34 (lower limb motor impairment); BBS ≤ 56 (balance impairment); N severe joint limitation or pain. | Kneeling posture training on a mat to promote trunk control and load perception in multiple directions, with progressive speed and heart rate targeting. | 30 min/session, 6×/week | 4weeks | Kneeling Group:  Kneeling: 30 min × 6/week × 4.0 = 720 METs-min/week  Routine: 60 min × 6/week × 3.5 = 1260 METs-min/week Total: 1980 METs-min/week | BBS FMA-LE |
|  |  | Middle- and high-income | 31 | Lower limb | Mixed | Subacute Phase | Moderate | 51.68±12.95 | M24/F7 | Y | RC |  | Treadmill walking at self-selected pace with safety harness, encouraging reduced handrail dependence and ankle stabilization. | 30 min/session, 6×/week | 4weeks | Treadmill Group:  Treadmill: 30 min × 6/week × 4.5 = 810 METs-min/week  Routine: 60 min × 6/week × 3.5 = 1260 METs-min/week Total: 2070 METs-min/week |  |
| 154 | Sidra Manzoor, 2021^239^ | Low- and middle-income | 8 | Upper limb | Nt reported | Acute and subacute | Moderate | Nt reported | Nt reported | Y | MBE | MRI/CT I/H stroke; FMA ≤29 (UL motor dysfunction); reduced grip but basic movements present. | Upper limb mirror therapy using a mirror placed at midline. Patients performed wrist, elbow, and grasping tasks while watching reflected movements. | 30 min/session, 5×/week | 2weeks | Mirror Therapy Group Routine: 30 min × 5 × 3.0 METs = 450 METs-min/week Mirror therapy: 30 min × 5 × 2.5 METs = 375 METs-min/week Total: 825 METs-min/week | FMA |
|  |  | Low- and middle-income | 8 | Upper limb | Nt reported | Acute and subacute | Moderate | Nt reported | Nt reported | Y | ULT |  | Same training tasks performed with the mirror covered, removing visual feedback from the unaffected limb. | 30 min/session, 5×/week | 2weeks | Sham Mirror Therapy Group Routine: same as above = 450 METs-min/week Sham therapy: 30 min × 5 × 2.0 METs = 300 METs-min/week Total: 750 METs-min/week |  |
| 155 | Kritika Verma, 2021^240^ | Middle income | 28 | Lower limb | Mixed | Chronic Phase | Moderate | 50.67±11.18 | M23/F5 | Y | MBE | DiagNsed as stroke by MRI or CT. | Lower limb mirror therapy focusing on ankle, knee, and hip movements. The patient observed the reflected image of the unaffected leg. | 30 min/session, 6×/week | 6weeks | Mirror + Gait Group Mirror + Gait: 30 min × 6 × 3.8 = 684 METs-min/week Routine neuro-PT: 60 min × 6 × 3.5 = 1260 METs-min/week Total: 1944 METs-min/week | BBS |
|  |  | Middle income | 28 | Lower limb | Mixed | Chronic Phase | Moderate | 49.16±10.28 | M19/F9 | Y | NPF |  | Comprehensive rehabilitation including NDT, physical therapy, occupational therapy, and gait exercises. | 30 min/session, 6×/week | 6weeks | Control Group Routine: 60 min × 6 × 3.5 = 1260 METs-min/week Sham therapy: 30 min × 6 × 2.0 = 360 METs-min/week Total: 1620 METs-min/week |  |
| 156 | Nilar AUNG, 2022^241^ | Low- and middle-income | 20 | Trunk and lower limb function | 40%I + 60%H | Subacute and chronic | Mild to moderate | 49.40±2.59 | M15/F5 | Y | MBE | DiagNsed as I or H stroke by MRI or CT. | Motor imagery session followed by structured progressive circuit training including walking, standing, and stair tasks. | 90 min/session (25 min MI + 65 min SPCCT), 3×/week | 4weeks | Experimental (MI + SPCCT) SPCCT: 65 min × 3 × 4.5 = 877.5 METs-min/week Motor imagery: 25 min × 3 × 2.0 = 150 METs-min/week Total: 1027.5 METs-min/week | 6MWT |
|  |  | Low- and middle-income | 20 | Trunk and lower limb function | 50%I + 50%H | Subacute and chronic | Mild to moderate | 55.55±2.40 | M11/F9 | Y | RC |  | Health education session followed by the same circuit training protocol. | 90 min/session (25 min HE+ 65 min SPCCT), 3×/week | 4weeks | Control (HE + SPCCT) SPCCT: same as above = 877.5 METs-min/week Health education: 25 min × 3 × 1.5 = 112.5 METs-min/week Total: 990 METs-min/week |  |
| 157 | Sumreen Anwar, 2022^242^ | Low- and middle-income | 20 | Lower limb | 13I + 8H | Subacute Phase | Moderate | 50.3±12.8 | M16/F5 | Y | MBE | DiagNsed as I or H stroke by MRI or CT. | Motor imagery training focused on visual and kinesthetic imagination of walking and turning, followed by gait practice. | 60 min/session, 3×/week | 6weeks | Experimental (Motor Imagery + Gait) Gait training: 30 min × 3 × 3.0 = 270 METs-min/week Motor imagery: 30 min × 3 × 2.0 = 180 METs-min/week Total: 450 METs-min/week | FMA |
|  |  | Low- and middle-income | 24 | Lower limb | 15I + 8H | Subacute Phase | Moderate | 53.7±12.1 | M14/F9 | N | RC |  | Progressive muscle relaxation training followed by gait exercises. | 60 min/session, 3×/week | 6weeks | Control (Relaxation + Gait) Gait training: 30 min × 3 × 3.0 = 270 METs-min/week Muscle relaxation: 30 min × 3 × 1.5 = 135 METs-min/week Total: 405 METs-min/week |  |
| 158 | Jisheng Wang, 2021^243^ | Middle- and high-income | 15 | Lower limb | Nt reported | Chronic Phase | Severe | Nt reported | Nt reported | Y | AE | DiagNsed as stroke by MRI or CT. | Low-intensity endurance training using a recumbent bike and treadmill, aiming for moderate heart rate targets, in addition to standard rehabilitation. | 30 min/session, 3×/week | 6weeks | Aerobic Group Routine rehab: 60 min × 5 × 3.0 = 900 METs-min/week Aerobic training: 30 min × 3 × 3.0 = 270 METs-min/week Total: 1170 METs-min/week | FMA |
|  |  | Middle- and high-income | 15 | Lower limb | Nt reported | Chronic Phase | Severe | Nt reported | Nt reported | N | NPF |  | Comprehensive conventional therapy combining neurofacilitation, seated balance, standing, and gait training with a fixed daily schedule. | 240 min/session, 5×/week | 6weeks | Control Group Routine only: 60 min × 5 × 3.0 = 900 METs-min/week |  |
| 159 | Shubham Kalyana, 2023^244^ | Middle income | 20 | Lower limb | Mixed | Subacute and chronic | Moderate | Nt reported | Nt reported | Y | GT | DiagNsed as I or H stroke via MRI or CT. | Backward walking training added to a conventional rehabilitation program, aiming to enhance lower limb strength and balance awareness. | 70 min/session, 3×/week | 3weeks | Backward Walking Group Backward walking: 30 min × 3 × 4.5 = 405 METs-min/week Routine rehab: 40 min × 3 × 3.5 = 420 METs-min/week Total: 825 METs-min/week | BBS |
|  |  | Middle income | 20 | Lower limb | Mixed | Subacute and chronic | Moderate | Nt reported | Nt reported | Y | RC |  | Conventional therapy including loading exercises, standing, and gait training, without backward walking. | 40 min/session, 3×/week | 3weeks | Control Group Routine rehab only: 40 min × 3 × 3.5 = 420 METs-min/week |  |
| 160 | Lin-Jian Zhang, 2024^245^ | Middle- and high-income | 28 | Lower limb | 17I + 11H | Subacute Phase | Moderate | 52.14±15.35 | M22/F6 | Y | RAT | MRI or CT confirmed stroke (I/H); Brunnstrom stage ≥ 3; MAS ≤ 2; MMSE ≥ 24. | Gait training using the A3 robotic exoskeleton with bodyweight support and VR feedback. Training focused on progressive unloading and gait correction. | 60 min/session, 5×/week | 2weeks | A3 Robot Training Group A3 robotic training: 30 min × 5/week × 3.5 METs = 525 METs-min/week Routine rehab: 30 min × 5/week × 3.0 METs = 450 METs-min/week Total: 975 METs-min/week | FMA-LE |
|  |  | Middle- and high-income | 27 | Lower limb | 17I + 10H | Subacute Phase | Moderate | 58.48±17.19 | M22/F5 | Y | RAT |  | Conventional therapy including overground gait, balance, and lower limb weight-bearing training. | 60 min/session, 5×/week | 2weeks | Ground Walking Group Ground walking: 30 min × 5/week × 3.0 METs = 450 METs-min/week Routine rehab: 30 min × 5/week × 3.0 METs = 450 METs-min/week Total: 900 METs-min/week |  |
| 161 | Congcong Huo, 2024^246^ | Middle- and high-income | 14 | Lower limb | 8I + 6H | Subacute Phase | Moderate | 57.93±11.47 | M10/F4 | Y | VRG | Unilateral I or H stroke confirmed by MRI or CT. | Robot-assisted gait training using LiteStepper® single-leg exoskeleton to support the paretic limb and provide coordinated motion and stability. | 30 min/session, 5×/week | 4weeks | Robot-assisted gait training Robot gait: 30 min × 5/week × 4.0 METs = 600 METs-min/week Routine rehab: 60 min × 5/week × 3.5 METs = 1050 METs-min/week Total: 1650 METs-min/week | BBS FMA-LE |
|  |  | Middle- and high-income | 16 | Lower limb | 13I + 3H | Subacute Phase | Moderate | 55.25±11.16 | M11/F5 | Y | RC |  | Conventional rehabilitation including joint mobilization, walking, balance, and functional tasks guided by a therapist. | 30 min/session, 10×/week | 4weeks | Control Routine rehab: 60 min × 5/week × 3.5 METs = 1050 METs-min/week |  |
| 162 | Ayça Utkan Karasu, 2018^247^ | Middle- and high-income | 12 | Lower limb and trunk | 8I + 4H | Subacute Phase | Moderate | 62.3±11.79 | M5/F7 | Y | VRG | Unilateral I or H stroke confirmed by MRI or CT. | Balance games using Wii Fit on a Wii Balance Board, with visual feedback training incorporated into standard rehabilitation. | 20 min/session, 5×/week | 4weeks | Wii Training Group Wii games: 20 min × 5/week × 3.0 METs = 300 METs-min/week Routine rehab: 150 min/week × 3.5 METs = 525 METs-min/week Total: 825 METs-min/week | BBS |
|  |  | Middle- and high-income | 11 | Lower limb and trunk | 10I + 1H | Subacute Phase | Moderate | 64.1±12.2 | M5/F6 | Y | NPF |  | Routine multidisciplinary rehabilitation combining NDT, physiotherapy, occupational therapy, cognitive and balance training. | 120–180 min/session, 5×/week |  | Control Routine rehab: 150 min/week × 3.5 METs = 525 METs-min/week |  |
| 164 | Jin-Young Chun, 2016^248^ | High income | 14 | Lower limb and trunk | 7I + 7H | Chronic Phase | Mild to moderate | 56.21±9.30 | M8/F6 | Y | VRG | Unilateral I or H stroke confirmed by MRI or CT. | 3D trunk stability training using a dynamic platform with 8-directional postural control and progressive tilt angles. | 30 min/session, 3×/week | 7weeks | Spine Balance 3D Group Balance training: 90 min/week × 4.5 METs = 405 METs-min/week Routine rehab: 90 min/week × 3.5 METs = 315 METs-min/week Total: 720 METs-min/week | 10MWT BBS |
|  |  | High income | 14 | Lower limb and trunk | 5I + 9H | Chronic Phase | Mild to moderate | 53.93±9.21 | M10/F4 | Y | BT |  | Traditional static and dynamic balance training using the Biodex system, focusing on weight shift and core stability. | 30 min/session, 3×/week | 7weeks | Biodex Group Biodex training: 90 min/week × 3.5 METs = 315 METs-min/week Routine rehab: 90 min/week × 3.5 METs = 315 METs-min/week Total: 630 METs-min/week |  |
| 165 | Rong Cui, 2023^249^ | Middle- and high-income | 23 | Trunk | 91%I + 9%H | Subacute Phase | Mild to moderate | 60-72 | M18/F5 | Y | MBE | Confirmed post-stroke unilateral hemiparesis (imaging Nt specified); TIS score 8–18; FMA, BBS, and MBI used for function assessment. | Tai Chi–based training focusing on axial rotation, diagonal movements, and trunk flexibility. Included eight structured Tai Chi forms alongside conventional therapy. | 50 min/session, 5×/week | 3weeks | WAT Group WAT: 30 min × 5/week × 3.0 METs = 450 METs-min/week Routine: 20 min × 5/week × 3.0 METs = 300 METs-min/week Total: 750 METs-min/week | BBS FMA FMA-LE FMA-UE |
|  |  | Middle- and high-income | 20 | Trunk | I | Subacute Phase | Mild to moderate | 63-71 | M17/F3 | Y | NPF |  | Trunk Selective Activity Training based on Bobath concept, including sitting, standing, and walking tasks aimed at enhancing trunk control and stability. | 50 min/session, 5×/week | 3weeks | TSAT Group TSAT: 30 min × 5/week × 2.5 METs = 375 METs-min/week Routine: 20 min × 5/week × 3.0 METs = 300 METs-min/week Total: 675 METs-min/week |  |
| 167 | Felix Nindorera, 2023^250^ | Low-income | 23 | Whole-body function | 48%I + 48%H性 + 4%Mixed | Chronic Phase | Mild to moderate | 50.9±10.7 | M18/F5 | N | FT | First or recurrent unilateral stroke diagNsed via imaging or clinical neurology exam. | Combination training involving walking, coordination, resistance exercises, and cycling. Activities were grouped by disability level and supervised. | 120 min/session, 3×/week | 12weeks | Study 167 – CBCS Multi-Component Training Circuit walking: 30 min × 3 × 4.0 = 360 METs-min/week Resistance/balance: 30 min × 3 × 3.5 = 315 METs-min/week Cycling: 30 min × 3 × 6.8 = 612 METs-min/week Total: 1287 METs-min/week | 6MWT 10MWT BBS |
|  |  | Low-income | 23 | Whole-body function | 35%I8 + 39%H性 + 26%Mixed | Chronic Phase | Mild to moderate | 50.1±11.2 | M18/F5 | N | NE |  | Participated in Nn-exertional cultural and recreational activities such as games and discussions, without physical training. | 120 min/session, 3×/week | 12weeks | 0 |  |
| 169 | Jacqueline C. Outermans, 2010^251^ | High income | 22 | Lower limb | Mixed | Subacute Phase | Mild to moderate | 56.8±8.6 | M19/F3 | Y | TOT | First or recurrent unilateral stroke diagNsed via imaging or clinical neurology exam. | High-intensity task-oriented gait training at ten workstations. Included walking, stair climbing, rapid walking, and turning, with heart rate monitored for aerobic intensity. | 45 min/session, 3×/week | 4weeks | High Intensity Training CBCS: 45 min × 3 × 5.2 METs = 702 METs-min/week Routine: 30 min × 5 × 3.0 METs = 450 METs-min/week Total: 1152 METs-min/week | 6MWT 10MWT BBS |
|  |  | High income | 21 | Lower limb | Mixed | Subacute Phase | Mild to moderate | 56.3±8.6 | M17/F4 | Y | RC |  | Low-intensity therapy focusing on balance, gait practice, and light interactive games without aerobic or high-repetition components. | 45 min/session, 3×/week | 4weeks | Low Intensity Training Low-intensity: 45 min × 3 × 3.0 = 405 METs-min/week Routine: 30 min × 5 × 3.0 = 450 METs-min/week Total: 855 METs-min/week |  |
| 170 | Anna Maria Malagoni, 2016^252^ | High income | 6 | Lower limb | 67%I + 33%H | Chronic Phase | Mild to moderate | 62.5±13.8 | M4/F3 | Y | GT | History of stroke ≥ 6 months; FAC > 3 (can walk ≥10 m); capable of completing TUG test. | Home-based walking training using a metroNme to maintain cadence. Walking intensity and pace were adjustedweekly, guided by hospital-based assessments. | 10 min/session, 12×/week | 10weeks | Ti-To Home Walking Ti-To walking: 10 min × 2 × 6 × 3.0 METs = 360 METs-min/week Total: 360 METs-min/week | 6MWT |
|  |  | High income | 6 | Lower limb | 67%I + 33%H | Chronic Phase | Mild to moderate | 70.7±9.0 | M5/F2 | N | RC |  | Supervised training in hospital including endurance tasks (e.g., stairs, walking) and resistance, balance, and flexibility exercises. | 60 min/session, 3×/week | 10weeks | SSR Group SSR supervised: 45 min × 3 × 3.0 METs = 405 METs-min/week Total: 405 METs-min/week |  |
| 171 | Claudia Miklitsch, 2013^253^ | High income | 20 | Trunk and lower limb function | 14I + 6H | Chronic Phase | Mild to moderate | 58±11 | M13/F7 | Y | FT | First stroke; stand ≥2min; walk (±aid); BI <100; no severe cognitive impairment. | Task-based balance and coordination training on a mini-trampoline. Included stepping, jumping, ball throwing, and heel lifts under therapist supervision. | 30 min/session, 3–4×/week | 3weeks | Experimental Group Main training: 3.33 × 30 min × 6.0 METs = 600 METs-min/week Routine rehab: 5 × 30 min × 3.0 METs = 450 METs-min/week Total: 1050 METs-min/week | 6MWT BBS |
|  |  | High income | 20 | Trunk and lower limb function | 17I + 3H | Chronic Phase | Mild to moderate | 57±12 | M12/F8 | Y | RC |  | Traditional balance training using flat-surface exercises and props (e.g., balls, ropes, boards), without trampoline use. | 30 min/session, 3–4×/week | 3weeks | Control Group Main training: 3.33 × 30 min × 3.0 = 300 METs-min/week Routine rehab: same as above = 450 METs-min/week Total: 750 METs-min/week |  |
| 172 | Junhyuck Park, 2017^254^ | High income | 13 | Upper limb | Mixed | Subacute Phase | Mild to moderate | 57.2±9.8 | Nt reported | Y | FT | History of stroke ≥6 months; K-MMSE > 21; can walk 10 m (with assistive device); N severe cognitive/pain impairments. | Seated boxing exercises including mitt strikes and punching bags, emphasizing upper limb coordination and control. | 30 min/session, 3×/week | 6weeks | Boxing + Routine Group Boxing: 20 min × 3 × 4.0 METs = 240 METs-min/week Routine PT: 30 min × 3 × 3.0 METs = 270 METs-min/week Total: 510 METs-min/week | 10MWT BBS |
|  |  | High income | 13 | Upper limb | Mixed | Subacute Phase | Mild to moderate | 56.5±10.2 | Nt reported | Y | NPF |  | Standard neurodevelopmental and proprioceptive neuromuscular facilitation therapies focusing on gait and strength, without boxing components. | 30 min/session, 3×/week | 6weeks | Routine Only Routine PT: 30 min × 3 × 3.0 = 270 METs-min/week |  |
| 173 | Song, R.，2021^255^ | High income | 18 | Whole-body function | 66.7%I + 33.3%H | Chronic Phase | Mild to moderate | 58.72±17.13 | M10/F8 | N | MBE | DiagNsed by imaging (MRI/CT); meets rehab referral criteria; can stand and perform tests; N severe cognitive deficits (K-MOCA). | Six-month Tai Chi program combining warm-up, Qigong breathing, and seated or standing Tai Chi forms, aiming to enhance strength, balance, and cognition. | 50 min/session, 2×/week | 6months | Study 173 – Tai Chi Group Tai Chi main: 35 min × 2 × 3.0 = 210 METs-min/week Qigong: 5 min × 2 × 2.5 = 25 METs-min/week Warm-up & cool-down: 10 min × 2 × 2.5 = 50 METs-min/week Total: 285 METs-min/week | BBS |
|  |  | High income | 16 | Whole-body function | 50.0%I8 + 50.0%H | Chronic Phase | Mild to moderate | 57.18±10.65 | M11/F5 | N | NE |  | Received stroke education viaweekly messages and phone consultations, with N exercise component. | SMS only, 1×/week | 6months | 0 |  |
| 175 | Esma Nur Kolbaşı, 2024^256^ | Middle- and high-income | 5 | Upper limb | Mainly I | Chronic Phase | Moderate | 68.8±5.9 | M2/F3 | N | CIMT | ≥18y; first unilateral stroke ≤12mo; MMSE ≥24; stand ≥2min; low-function (Alabama); excl: VAS ≥5, MAS ≥3, aphasia, severe vision. | Modified constraint-induced movement therapy using mitt restraint on the unaffected hand, with repetitive task practice. | 60 min/session, 3×/week | 5weeks | mCIMT Group 60 min × 3 × 3.0 METs = 540 METs-min/week | FMA-UE |
|  |  | Middle- and high-income | 5 | Upper limb | Mainly I | Chronic Phase | Moderate | 60.6±10.2 | M3/F2 | N | MBE |  | Same mCIMT protocol preceded by sham iTBS using a deactivated coil and minimal stimulation. | 60 min/session, 3×/week | 5weeks | iTBS + mCIMT Group mCIMT: same = 540 METs-min/week iTBS: 3 min × 1.5 × 1.5 METs ≈ 6.75 (igNred) Total: rounded to 540 METs-min/week |  |
|  |  | Middle- and high-income | 5 | Upper limb | Mainly I | Chronic Phase | Moderate | 69.4±9.9 | M2/F3 | N | ESX |  | Same mCIMT preceded by real iTBS stimulation of the motor cortex using 600 pulses at 80% resting motor threshold. | 60 min/session, 3×/week | 5weeks | iTBS + mCIMT Group mCIMT: same = 540 METs-min/week iTBS: 3 min × 1.5 × 1.5 METs ≈ 6.75 (igNred) Total: rounded to 540 METs-min/week |  |
| 176 | İrem Akgün, 2024^257^ | Middle- and high-income | 12 | Upper limb function | 91%I + 9%H | Chronic Phase | Moderate | 57.3±12.1 | M9/F3 | N | RT | Single stroke confirmed by CT/MRI; MAS <3 (elbow/wrist flexors); MMSE ≥24; age 18–85 years. | Used ExoRehab X device for single-joint upper limb training (active/resisted), guided by interactive video games (e.g., elbow flexion, wrist dorsiflexion). | 40–60 min/session, 2×/week | 6weeks | Exoskeleton Group (EG): ExoRehab X: 50min × 2/week × 6weeks = 600min METs = 3.5 → 600 × 3.5 = 2100 METs-min/week | FMA-UE |
|  |  | Middle- and high-income | 12 | Upper limb function | 83%I + 17%H | Chronic Phase | Moderate | 66.7±10.5 | M5/F7 | N | NPF |  | Performed bilateral upper limb functional training based on Bobath principles, including trunk control, shoulder rotation, symmetrical actions, and simulated daily tasks. | 40–60 min/session, 2×/week | 6weeks | Bobath Group (BG): Bobath: 50min × 2/week × 6weeks = 600min METs = 2.8 → 600 × 2.8 = 1680 METs-min/week |  |
| 177 | Toh et al., 2024^258^ | Middle- and high-income | 20 | Upper limb | Mainly I | Chronic Phase | Moderate | 55.45±9.98 | M12/F8 | Y | TAE | FTHUE-HK score 3–6; MAS ≤2; MMSE ≥19; N excessive pain/edema; N major upper limb limiting illness. | Used Smart Reminder wearable system with smartphone app to prompt and monitor upper limb activity. Data were captured via accelerometer and gyroscope. | 60 min/session, 5×/week | 4weeks | Remote Training Group: Remote session: 60 min × 5 sessions/week × 3.0 METs = 900 METs-min/week Conventional rehabilitation: 45 min × 1 session/week × 3.0 METs = 135 METs-min/week Total: 900 + 135 = 1035 METs-min/week | FMA-UE |
|  |  | Middle- and high-income | 20 | Upper limb | Mainly I | Chronic Phase | Moderate | 57.80±9.22 | M13/F7 | Y | ULT |  | Performed the same exercises using paper-based instructions and wore Nn-feedback accelerometers.weekly in-person check-ins were conducted. | 60 min/session, 5×/week | 4weeks | Booklet Training Group: Booklet session: 60 min × 5 sessions/week × 3.0 METs = 900 METs-min/week Conventional rehabilitation: 45 min × 1 session/week × 3.0 METs = 135 METs-min/week Total: 900 + 135 = 1035 METs-min/week |  |
| 178 | Eun Cho Park, 2015^259^ | High income | 20 | Whole-body function | Mixed | Chronic Phase | Moderate | 51.15±14.81 | M10/F10 | Y | MBE | Imaging (CT/MRI) confirmed stroke; K-MMSE ≥24; N visual, vestibular, or orthopedic issues; can follow movement commands. | Watched gait-related videos (walking on level ground, ramps, stairs), followed by matching physical gait tasks under supervision. | 30 min/session, 5×/week | 8weeks | AOGT Group (Action Observation + Gait Training):Action observation: 10 min × 5/week × 1.5 METs = 75 METs-min/week Gait training (level + ramp + stairs): 20 min × 5/week × 3.8 METs = 380 METs-min/week Conventional rehab (PT): 30 min × 5/week × 3.0 METs = 450 METs-min/week Total: 75 + 380 + 450 = 905 METs-min/week | 10MWT |
|  |  | High income | 20 | Whole-body function | Mixed | Chronic Phase | Moderate | 48.65±12.81 | M11/F9 | Y | GT |  | Watched Nn-gait nature videos before performing the same gait tasks as the intervention group. | 30 min/session, 5×/week | 8weeks | CG (Control Group): Neutral video: 10 min × 5/week × 1.0 MET = 50 METs-min/week Gait training: 20 min × 5/week × 3.8 METs = 380 METs-min/week Conventional rehab: 30 min × 5/week × 3.0 METs = 450 METs-min/week Total: 50 + 380 + 450 = 880 METs-min/week |  |
| 179 | Bang Dae-Hyouk, 2013^260^ | High income | 15 | Whole-body function | 7I + 8H | Chronic Phase | Moderate | 64.1±6.35 | M9/F6 | Y | MBE | Imaging (CT/MRI) confirmed first-ever I/H stroke; MMSE ≥21; gait speed ≥0.5 m/s; able to walk 10 m; N interfering comorbidities. | Watched treadmill walking videos (front, side, rear views) with different speeds, then performed treadmill gait training. | 40 min/session, 5×/week | 4weeks | AOT Group (Action Observation + Treadmill): Action observation (treadmill video): 9 min × 5/week × 1.5 METs = 67.5 METs-min/week Treadmill training: 30 min × 5/week × 4.0 METs = 600 METs-min/week Total: 67.5 + 600 = 667.5 METs-min/week | 6MWT 10MWT |
|  |  | High income | 15 | Whole-body function | 8I + 7H | Chronic Phase | Moderate | 58.9±7.03 | M8/F7 | Y | GT |  | Watched unrelated nature videos followed by the same treadmill training as the intervention group. | 40 min/session, 5×/week | 4weeks | Control Group: Neutral video: 9 min × 5/week × 1.0 MET = 45 METs-min/week Treadmill training: 30 min × 5/week × 4.0 METs = 600 METs-min/week Total: 45 + 600 = 645 METs-min/week |  |
| 180 | Kyeoung Man Jung, 2019^261^ | High income | 18 | Upper limb | 10I + 8H | Acute Phase | Moderate | 60.30±7.56 | M10/F8 | N | CST | WHO stroke criteria + MRI; shoulder subluxation ≥9.5 mm; MMSE ≥24; shoulder MFT: Fair–Poor; MAS ≤2. | Sling-based shoulder training using the Redcord™ system. Exercises included active abduction, flexion, and rotation in sitting, side-lying, and supine positions with light resistance. | 40 min/session, 5×/week | 4weeks | Suspension Training Group: Suspension training: 40 min × 5/week × 3.0 METs = 600 METs-min/week Conventional PT/OT Nt included due to unclear duration | FMA-UE |
|  |  | High income | 18 | Upper limb | 9I + 9H | Acute Phase | Moderate | 57.18±8.41 | M9/F9 | N | ULT |  | Bilateral upper limb training with symmetrical movements such as shoulder flexion, elbow extension, and overhead reaching in supine and seated positions. | 40 min/session, 5×/week | 4weeks | Bilateral Upper Limb Training Group: Bilateral arm training: 40 min × 5/week × 3.0 METs = 600 METs-min/week |  |
| 181 | Xiaoping Hong, 2024^262^ | Middle- and high-income | 30 | Trunk and lower limb function | 40%H + 60%I | Subacute Phase | Severe | 54.63±4.24 | M22/F8 | Y | TCMEX | Meets Chinese Cerebrovascular Disease Conference criteria; confirmed by CT/MRI; stroke <2 months; stable vitals; movement impairment; cooperative. | Combined acupuncture (targeting motor cortex and lower limb acupoints) and core stability training (bridging, pelvic control, weight shifting). Also received conventional rehabilitation therapies. | 45 min/session (core), 30 min/session (acupuncture), 6×/week | 8weeks | Experimental Group: Conventional rehabilitation: 45 min × 6/week × 3.25 METs = 877.5 METs-min/week Acupuncture: 30 min × 6/week × 1.5 METs = 270 METs-min/week Total: 877.5 + 270 = 1147.5 METs-min/week | BBS |
|  |  | Middle- and high-income | 30 | Trunk and lower limb function | 37%H + 63%I | Subacute Phase | Severe | 55.00±3.79 | M23/F7 | Y | CST |  | Same physical therapy as the intervention group, but without acupuncture. | 45 min/session (core), 30 min/session (acupuncture), 6×/week | 8weeks | Control Group: Conventional rehabilitation only: 45 min × 6/week × 3.25 METs = 877.5 METs-min/week |  |
| 182 | Tobias Braun, 2016^263^ | High income | 14 | Trunk and lower limb function | 13I + 1H | Subacute Phase | Severe | 61±16 | M7/F7 | Y | VRG | Imaging confirmed stroke <3 months; Nn-ambulatory (FAC ≤2); stable vitals; cognitive ability to follow commands. | Dynamic standing training using Balance-Trainer with 360° platform movement and visual-feedback-based gaming to encourage postural control in all directions. | 30 min/session, 3–5×/week | 5weeks | Dynamic Standing Group: Dynamic standing: 30 min × 4/week × 3.3 METs = 396 METs-min/week Conventional rehab: 30 min × 5/week × 3.0 METs = 450 METs-min/week Total: 396 + 450 = 846 METs-min/week | BBS |
|  |  | High income | 14 | Trunk and lower limb function | 10I + 4H | Subacute Phase | Severe | 60±14 | M5/F9 | Y | BT |  | Static standing training using a standard frame, focusing on maintaining upright posture without dynamic balance tasks. | 30 min/session, 3–5×/week | 5weeks | Static Standing Group: Static standing: 30 min × 4/week × 2.0 METs = 240 METs-min/week Conventional rehab: 30 min × 5/week × 3.0 METs = 450 METs-min/week Total: 240 + 450 = 690 METs-min/week |  |
| 183 | Hong Jin, 2013^264^ | Middle- and high-income | 65 | Lower limb | I | Chronic Phase | Mild to moderate | 57.6±6.6 | M46/F19 | Y | AE | CT/MRI confirmed stroke >6 months; Chinese Han ethnicity; able to walk independently (with/without walker). | Interval cycling on an ergometer, progressing from moderate to vigorous intensity, with rest periods and heart rate monitored via Polar devices. | 40 min/session, 5×/week | 12weeks | Aerobic Cycling Group:  Cycling (50–70% HRR, 6.8 METs): 40 min × 5/week = 200 min → 200 × 6.8 = 1360 METs-min/week Stretching: 35 min × 5/week × 2.5 METs = 437.5 METs-min/week Walking (light): 5 min × 5/week × 2.8 METs = 70 METs-min/week Total: 1360 + 437.5 + 70 = 1867.5 METs-min/week | 6MWT |
|  |  | Middle- and high-income | 63 | Lower limb | I | Chronic Phase | Mild to moderate | 56.3±6.5 | M45/F18 | Y | RC |  | Low-intensity rehabilitation including stretching and slow walking, Nt designed to improve cardiovascular fitness. | 40 min/session, 5×/week | 12weeks | Control Group (Stretch + Walk): Stretching: 35 min × 5/week × 2.5 METs = 437.5 METs-min/week Walking (light): 5 min × 5/week × 2.8 METs = 70 METs-min/week Total: 437.5 + 70 = 507.5 METs-min/week |  |
| 185 | Larissa Tavares Aguiar, 2020^265^ | High income | 11 | Whole-body function | Mixed | Chronic Phase | Moderate | 52±11 | M18/F6 | Y | AE | Stroke (method N/A); ≥20y; >6mo; inactive (CDC); excl: severe cognitive, walk <10min, severe pain/disability. | Treadmill aerobic exercise with warm-up and cool-down, adjusted to maintain target heart rate zone via speed control. | 40 min/session, 3×/week | 12weeks | AT Group: Cycling: 45 min × 3/week × 5.5 METs = 742.5 METs-min/week Conventional rehab: 40 min × 2/week × 3.0 METs = 240 METs-min/week Total: 742.5 + 240 = 982.5 METs-min/week | 6MWT 10MWT |
|  |  | High income | 11 | Whole-body function | Mixed | Chronic Phase | Moderate | 48±10 | M19/F5 | Y | RC |  | Outdoor walking at comfortable speed with heart rate kept below aerobic threshold. | 40 min/session, 3×/week | 12weeks | Control Group: Conventional rehab only: 40 min × 2/week × 3.0 METs = 240 METs-min/week |  |
| 186 | Pei-Hsin Ku, 2020^266^ | Middle- and high-income | 10 | Trunk and lower limb function | 7I + 2H + 1Mixed | Chronic Phase | Mild to moderate | 55.0±7.3 | M7/F3 | N | WA | I/H/mixed stroke (method N/A); 20–80y; first stroke ≥6mo; unilateral hemiplegia; walk ≥15m; MMSE >24. | Ai Chi aquatic training with progressive Tai Chi–like movements emphasizing breathing, trunk control, and limb motion in warm water. | 60 min/session, 3×/week | 6weeks | Ai Chi Group: Warm-up + Ai Chi (45 min × 3/week × 3.0 METs) = 405 METs-min/week Water walking: 15 min × 3/week × 4.5 METs = 202.5 METs-min/week Total: 405 + 202.5 = 607.5 METs-min/week | BBS FMA |
|  |  | Middle- and high-income | 10 | Trunk and lower limb function | 6I + 2H + 2Mixed | Chronic Phase | Mild to moderate | 52.5±6.3 | M7/F3 | N | RC |  | Aquatic exercises involving passive and active movements, stretching, resistance with float devices, and gait practice. | 60 min/session, 3×/week | 6weeks | Water Resistance Walking Group: Warm-up/stretch: 15 min × 3/week × 2.5 METs = 112.5 METs-min/week Resistance training: 30 min × 3/week × 3.5 METs = 315 METs-min/week Water walking: 15 min × 3/week × 4.5 METs = 202.5 METs-min/week  Total: 112.5 + 315 + 202.5 = 630 METs-min/week |  |
| 187 | Ada Tang, 2009^267^ | High income | 23 | Lower limb | 74%I + 22%H + 1Mixed | Subacute Phase | Moderate | 64.7±3.6 | M52%/F48% | Y | AE | First-ever stroke <3 months; can walk ≥5 m (with aid if needed); CMSA lower limb score 3–6; can understand and consent. | Semi-recumbent cycling at progressive workload, with RPE and blood pressure monitored to maintain safety and intensity progression until discharge. | 30 min/session, 3×/week | 3-4weeks | Intervention Group: Conventional rehab: 30 min × 5/week × 3.0 METs = 450 METs-min/week Bike training (27.1 min × 3/week × 5.5 METs) = → 27.1 × 3 × 5.5 = 447.15 ≈ 447 METs-min/week Total: 450 + 447 = 897 METs-min/week | 6MWT |
|  |  | High income | 22 | Lower limb | 74%I + 22%H + 1Mixed | Subacute Phase | Moderate | 65.7±2.3 | M52%/F48% | N | RC |  | Standard physical therapy individualized to patient needs, excluding any structured aerobic training. | 30 min/session, 3×/week | 3-4weeks | Control Group: Conventional rehab only: 30 min × 5/week × 3.0 METs = 450 METs-min/week |  |
| 188 | Florian Tripp & Karsten Krakow, 2014^268^ | High income | 14 | Trunk and lower limb function | 12I + 2H | Subacute Phase | Moderate to severe | 64.8±15.0 | M9/F5 | Y | WA | First-ever stroke (I/H); post-acute inpatient rehab; able to transfer independently (with minimal help). | Halliwick aquatic therapy with adaptation, rotational control, and gait components. Supplemented by conventional land-based therapy. | 45 min/session, 3×/week | 2weeks | Aquatic Group: Aquatic therapy: 35 min × 3/week × 4.5 METs = 472.5 METs-min/week Conventional PT: 45 min × 2/week × 3.0 METs = 270 METs-min/week Total: 472.5 + 270 = 742.5 METs-min/week | BBS |
|  |  | High income | 16 | Trunk and lower limb function | 15I + 1H | Subacute Phase | Moderate to severe | 65.0±15.1 | M10/F6 | Y | RC |  | Standard physiotherapy including gait and balance exercises, possibly treadmill use. | 45 min/session, 3×/week | 2weeks | Control Group: Conventional PT: 45 min × 5/week × 3.0 METs = 675 METs-min/week |  |
| 189 | Kyunghoon Kim, 2015^269^ | High income | 11 | Lower limb | 63.6%I + 36.4%H | Subacute Phase | Moderate | 49.0±13.1 | M6/F5 | Y | ESX | First-ever stroke ≥6weeks; MMSE ≥24; N major vision/hearing loss; cleared by physician to exercise safely. | FES targeting peroneal nerve, combined with proprioceptive training (e.g., weight shifting) and ankle strength exercises (isometric, multi-directional). | 60 min/session, 5×/week | 6weeks | FPS Group (FES + Proprioception + Strength): FES: 30 min × 5 × 1.5 METs = 225 Proprioception: 15 min × 5 × 2.5 METs = 187.5 Ankle strength: 15 min × 5 × 3.0 METs = 225 Subtotal: 637.5 METs-min/week Conventional PT: 30 min × 5 × 3.0 = 450 Total: 1087.5 METs-min/week | BBS |
|  |  | High income | 11 | Lower limb | 63.6%I + 36.4%H | Subacute Phase | Moderate | 54.9±14.0 | M8/F3 | Y | RC |  | Same FES protocol followed by passive and active ankle stretching, without strength training. | 60 min/session, 5×/week | 6weeks | FS Group (FES + Stretching): FES: 30 min × 5 × 1.5 = 225 Stretching: 30 min × 5 × 2.5 = 375 Subtotal: 600 METs-min/week Conventional PT: 30 min × 5 × 3.0 = 450 Total: 1050 METs-min/week |  |
| 190 | Kyung-Hun Kim, 2022^270^ | High income | 15 | Trunk and lower limb function | 9I + 6H | Chronic Phase | Mild to moderate | 53.27±10.12 | M9/F6 | Y | TCMEX | K-MMSE ≥24; all patients can walk independently for 6 minutes. | Ankle proprioceptive training and contrast thermotherapy (hot/cold packs), followed by functional exercises for balance and posture. | 60 min/session, 5×/week | 8weeks | APT Group: Proprioceptive ankle training: 15 min × 5 × 2.5 = 187.5 Thermotherapy: 15 min × 5 × 1.5 = 112.5 Conventional rehab: 30 min × 5 × 3.0 = 450 Total: 187.5 + 112.5 + 450 = 750 METs-min/week | 10MWT BBS |
|  |  | High income | 15 | Trunk and lower limb function | 10I + 5H | Chronic Phase | Mild to moderate | 54.13±9.35 | M8/F7 | Y | NPF |  | Standard rehabilitation using Bobath, PNF, balance, gait, strength, and joint mobilization techniques. | 60 min/session, 5×/week | 8weeks | Control Group: Conventional rehab: 60 min × 5 × 3.0 = 900 METs-min/week |  |
| 191 | Eun-Kyung Kim, 2015^271^ | High income | 10 | Lower limb | Mixed | Chronic Phase | Moderate | 69.1±3.2 | M5/F5 | N | WA | CT/MRI dx; MMSE ≥24; Brunnstrom ≥4; walk 10m independently. | Aquatic PNF training in supine position using rhythmic initiation techniques. Movements followed D1/D2 patterns for lower limbs, assisted with flotation aids. | 30 min/session, 5×/week | 6weeks | Balance Group: Balance training: 30 min × 5 × 4.5 METs = 675 METs-min/week | BBS |
|  |  | High income | 10 | Lower limb | Mixed | Chronic Phase | Moderate | 68.0±3.1 | M5/F5 | N | LLT |  | Same PNF protocol as AE group, performed on land without aquatic support. | 30 min/session, 5×/week | 6weeks | Control Group: PT: 30 min × 5 × 3.0 METs = 450 METs-min/week |  |
| 192 | Jin Seok Huh, 2015^272^ | High income | 23 | Trunk and lower limb function | 13I + 10H | Subacute Phase | Moderate | 69.51±0.92 | M16/F7 | Y | VRG | CT/MRI stroke; MMSE ≥24; knee ext MMT ≥3; no severe visual/perceptual/LL MSK disorders. | Balance training using BalPro device with visual feedback. Included weight shifting, knee flexion, and a fruit-picking game requiring postural control. | 30 min/session, 5×/week | 2weeks | BalPro Group: BalPro: 30 min × 5 × 3.5 METs = 525 PT: 30 min × 5 × 3.0 METs = 450 Total: 975 METs-min/week | 6MWT BBS |
|  |  | High income | 17 | Trunk and lower limb function | 9I + 8H | Subacute Phase | Moderate | 60.87±6.12 | M10/F7 | Y | RC |  | Traditional physiotherapy including gait, strength, and balance training performed twice daily. | 60 min/session (split into 2 sessions), 5×/week | 2weeks | Control Group: PT: 60 min × 5 × 3.0 METs = 900 METs-min/week |  |
| 193 | Aamir Gul Memon, 2022^273^ | Low- and middle-income | 30 | Trunk and lower limb function | Mixed | Subacute Phase | Moderate | 54.82±5.04 | M23/F7 | Y | MBE | Stroke hx >6mo; FMS 2–4; stand ≥5min; no significant sensory/vestibular/LL ortho disorders. | Task-oriented balance training with progressive sensory input integration. Included seated, tandem, single-leg, and obstacle-based walking under eY-open and eY-closed conditions. | 40 min/session, 3×/week | 6weeks | Combined Stimulus Group: Stretching: 15 min × 3 × 2.5 METs = 112.5 Balance + sensory input: 25 min × 3 × 4.0 METs = 300 Total: 412.5 METs-min/week | BBS |
|  |  | Low- and middle-income | 30 | Trunk and lower limb function | Mixed | Subacute Phase | Moderate | 54.12±5.42 | M19/F11 | Y | TOT |  | Same balance training tasks without added sensory challenges. | 40 min/session, 3×/week | 6weeks | Task-Specific Balance Group: Stretching: 15 min × 3 × 2.5 METs = 112.5 Balance task training: 25 min × 3 × 3.5 METs = 262.5 Total: 375 METs-min/week |  |
| 196 | Gordana Grozdek Čovčić, 2022^274^ | Middle- and high-income | 20 | Trunk and lower limb function | Mixed | Chronic Phase | Severe | 54.3±12.7 | M6/F14 | N | NPF | CT/MRI stroke; 18–75y; no severe cognitive/psychiatric/neurodegenerative disorders or recurrent stroke. | Standard Bobath therapy focused on posture, gait, and balance. Aimed at restoring neuromuscular coordination through sensorimotor practice. | 45 min/session, 5×/week | 5weeks | Bobath only: 45 min × 5 days × 3.0 METs = 675 METs-min/week | BBS |
|  |  | Middle- and high-income | 20 | Trunk and lower limb function | Mixed | Chronic Phase | Severe | 46.4±16.7 | M12/F8 | N | TCMEX |  | Bobath therapy plus soft tissue mobilization of key lower limb muscles using manual techniques to reduce spasticity and improve gait. | 45 min/session (Bobath) + 20 min/session (Soft tissue release), 5×/week | 5weeks | Bobath + STM Mobilization: Bobath: 675 METs-min/week STM (Soft Tissue Mobilization): 20 min × 3 × 2.0 METs = 120 METs-min/week Total = 675 + 120 = 795 METs-min/week |  |
| 197 | Hwang-Jae Lee, 2013^275^ | High income | 15 | Lower limb | 10I + 5H | Subacute Phase | Moderate | 52.47±9.41 | M12/F3 | Y | ESX | CT/MRI stroke; ankle DF ≥5°, strength ≥1; K-MMSE ≥24; follow commands; walk 10m (±aid); Brunnstrom 1–4 (LL); no major gait comorbidities. | Treadmill walking with bodyweight support and functional electrical stimulation (FES) triggered by muscle activity, aimed at improving gait symmetry and weight shifting. | 30 min/session, 5×/week | 4weeks | BWT-PAFES Group:  BWT-PAFES: 30 min × 5 × 5.5 = 825 PT: 60 min × 5 × 3.0 = 900 OT: 30 min × 5 × 2.5 = 375 Total = 825 + 900 + 375 = 2100 METs-min/week | BBS |
|  |  | High income | 15 | Lower limb | 12I + 3H | Subacute Phase | Moderate | 56.73±7.24 | M10/F5 | Y | GT |  | Same treadmill protocol without FES application. | 30 min/session, 5×/week | 4weeks | BWSTT Group: BWSTT: 30 min × 5 × 5.0 = 750 PT: 60 min × 5 × 3.0 = 900 OT: 30 min × 5 × 2.5 = 375 ✅ Total = 750 + 900 + 375 = 2025 METs-min/week |  |
| 198 | Xiao-Ming Yu, 2020^276^ | Middle- and high-income | 36 | Trunk and lower limb function | 25I + 11H | Chronic Phase | Moderate to severe | 63.03±8.92 | M21/F15 | Y | MBE | CT/MRI stroke; MMSE ≥24; stand/walk unaided; no CV/visual/MSK conditions. | Tai Chi–based gait training with bodyweight support, progressively reduced. Movements included various step types and static balance patterns adapted from simplified Tai Chi. | 40 min/session, 3×/week | 12weeks | Tai Chi Walking: Tai Chi: 20 min × 3 × 4.0 = 240 Conventional rehab: 20 min × 3 × 3.0 = 180 Total = 420 METs-min/week | BBS FMA-LE |
|  |  | Middle- and high-income | 35 | Trunk and lower limb function | 25I + 11H | Chronic Phase | Moderate to severe | 58.69±9.72 | M20/F15 | Y | NPF |  | Routine rehabilitation with joint exercises, PNF, resistance, and gait training. | 40 min/session, 3×/week | 12weeks | Control Group: Conventional rehab only: 40 min × 3 × 3.0 = 360 METs-min/week |  |
| 199 | Liying Zhang, 2025^277^ | Middle- and high-income | 31 | Upper limb | Mainly I | Subacute Phase | Moderate | 59.41±11.78 | M67.6%/F32.4% | Y | MBE | CT/MRI confirmed I or H stroke; Brunnstrom stage ≥ 4 (upper limb); MMSE ≥ 22; BP <160/100 mmHg; N major cardiac/neuro disorders; shoulder pain score <5. | Tai Chi Yunshou arm training with robotic assistance and partial bodyweight support. Emphasized circular movements, coordination, and multisensory feedback. | 60 min/session, 5×/week | 12weeks | CRT + BWS-TCY Group: CRT: 30 min × 5 × 3.0 = 450 BWS-TCY (Tai Chi with body weight support): 30 min × 5 × 3.5 = 525 Total = 975 METs-min/week | FMA-UE |
|  |  | Middle- and high-income | 31 | Upper limb | Mainly I | Subacute Phase | Moderate | 65.44±13.36 | 58.8%M/F41.2% | Y | RAT |  | Robot-assisted upper limb rehabilitation using virtual targets and force-guided movement training. | 60 min/session, 5×/week | 12weeks | CRT + RAT Group: CRT: 30 min × 5 × 3.0 = 450 RAT (Robot-Assisted Training): 30 min × 5 × 3.3 = 495 Total = 945 METs-min/week |  |
|  |  | Middle- and high-income | 31 | Upper limb | Mainly I | Subacute Phase | Moderate | 63.13±12.51 | 73.5%M/F26.5% | Y | RC |  | Conventional therapy involving ROM, physical therapy, and occupational therapy focused on ADL function. | 60 min/session, 5×/week | 12weeks | CRT Only Group: CRT: 30 min × 5 × 3.0 = 450 METs-min/week |  |
| 202 | Ingrid G L van de Port, 2012^278^ | High income | 126 | Lower limb | Nt reported | Subacute Phase | Mild to moderate | 58±10 | M 57%/F 43% | Y | TOT | WHO-defined stroke, confirmed by CT/MRI; FAC ≥ 3; MMSE ≥ 24; N major comorbidities. | Task-based circuit including gait, step, balance, and ADL simulation across eight workstations. Peer observation and music used to enhance motivation and engagement. | 90 min/session, 2×/week | 12weeks | Multi-station Functional Training Group: Functional training: 90 min × 2/week × 5.0 METs = 900 Conventional inpatient rehab: 60 min × 5 × 3.0 = 900  Total = 900 + 900 = 1800 METs-min/week | 6MWT |
|  |  | High income | 124 | Lower limb | Nt reported | Subacute Phase | Mild to moderate | 58±10 | M 61%/F 39% | N | RC |  | Individualized standard physiotherapy covering gait, strength, and balance, based on Dutch guidelines. | 45 min/session, 3×/week | 12weeks | Control Group: Conventional inpatient rehab only: 60 min × 5 × 3.0 = 900 METs-min/week |  |
| 203 | Sunghee Lee, 2015^279^ | High income | 8 | Upper limb | 4H + 4I | Chronic Phase | Mild to moderate | 56.1±7.1 | 4M/4F | N | TOT | CT/MRI stroke; MMSE-K ≥23; Brunnstrom ≥4 (UL); mild hand dysfunction; | Upper limb task-oriented therapy focusing on ROM, functional tasks (e.g., stacking, threading, writing), and proximal-distal control to improve ADL capabilities. | 60 min/session, 5×/week | 8weeks | TOET Group (Task-Oriented Exercise Therapy): 60 min × 5 × 3.5 METs = 1050 METs-min/week | FMA-UE |
|  |  | High income | 8 | Upper limb | 3H + 5I | Chronic Phase | Mild to moderate | 57.6±5.7 | 5M/3F | N | MBE |  | Cognitive-motor training including visuospatial tasks, tactile shape identification, and cognitive-feedback–driven movement control to enhance sensorimotor integration. | 60 min/session, 5×/week | 8weeks | CET Group (Cognitive Exercise Therapy): 60 min × 5 × 2.8 METs = 840 METs-min/week |  |
| 204 | Georgia Iatridou, 2024^280^ | High income | 26 | Trunk and lower limb function | 21I + 5H | Chronic Phase | Mild to moderate | 55.5±10.2 | M15/F11 | N | WA | CT/MRI I/H stroke; MMSE ≥24; stand ≥2min unaided; no contraindications (e.g., infection); consented. | Aquatic therapy in a 34°C pool involving mobility warm-up, resistance, balance, gait, and respiratory relaxation tasks to enhance lower limb strength and posture. | 50 min/session, 3×/week | 6weeks | Aquatic Therapy Group: Warm-up (5 min × 3 × 3.5 METs) = 52.5 Stretching (10 × 3 × 2.5) = 75 Resistance (10 × 3 × 4.0) = 120 Balance/Gait (20 × 3 × 4.5) = 270 Cool down (5 × 3 × 2.0) = 30 Total = 547.5 METs-min/week | BBS |
|  |  | High income | 25 | Trunk and lower limb function | 20I + 5H | Chronic Phase | Mild to moderate | 57.8±10.3 | M16/F9 | N | FT |  | Same exercises as aquatic group performed on land with therapist supervision, matched in intensity and content. | 50 min/session, 3×/week | 6weeks | Land Therapy Group: Adjusted same schedule, resistance (120), balance (240)  Total = 517.5 METs-min/week |  |
| 205 | Nehir Toktas, 2024^281^ | Middle- and high-income | 14 | Lower limb | 10I + 4H | Chronic Phase | Moderate | 58.79±10.19 | M8/F6 | Y | ESX | First stroke (CT/MRI); ≥3mo; 18–70y; stable; 6MWT (±aid). | Participants received tDCS brain stimulation targeting the affected motor cortex (C3/C4), followed by task-oriented rehabilitation. Each stimulation session lasted 20 minutes before the 1-hour training. | 60 min/session (task) + 20 min/session (tDCS), 5×/week | 4weeks | Experimental Group (tDCS + TOET): Task training: 60 × 5 × 3.5 = 1050 tDCS: 20 × 5 × 1.3 = 130 Total = 1180 METs-min/week | 10MWT BBS FMA-LE |
|  |  | Middle- and high-income | 14 | Lower limb | 12I + 2H | Chronic Phase | Moderate | 62.57±8.53 | M7/F7 | Y | TOT |  | Participants received sham tDCS with only 30 seconds of initial stimulation to mimic tingling, followed by the same task-oriented rehabilitation. | 60 min/session (task) + 20 min/session (tDCS), 5×/week | 4weeks | Control Group: Same task training = 1050 Sham tDCS (30 sec) = Negligible |  |
| 206 | Su-yeon Hong, 2020^282^ | High income | 8 | Trunk and lower limb function | 4I + 4H | Chronic Phase | Moderate to severe | 56.63±8.78 | M6/F2 | N | MBE | CT/MRI confirmed stroke; MMSE ≥ 24; able to stand ≥3 minutes, walk 10 m; N systemic or balance-compromising illness. | Participants performed dual-task training using visual cues (traffic light signals) while lifting the affected leg in various directions. Elastic resistance was gradually increased based on success rate. | 30 min/session, 3×/week | 4weeks | CBT Group: 30 min × 3 × 4.0 = 360 METs-min/week | BBS |
|  |  | High income | 9 | Trunk and lower limb function | 6I + 3H | Chronic Phase | Moderate to severe | 66.22±11.55 | M4/F5 | N | BT |  | Participants performed the same leg-lifting movements as CBT group but without cognitive visual cues. The intensity and repetitions were matched. | 30 min/session, 3×/week | 4weeks | GBT Group: 30 min × 3 × 3.5 = 315 METs-min/week |  |
| 207 | Saleh M. Aloraini, 2022^283^ | Middle- and high-income | 19 | Lower limb | 16I + 3H | Chronic Phase | Moderate | 60.1±10.8 | M10/F9 | N | CIMT | Single I/H stroke (CT/MRI); MMSE ≥24; BBS ≥35; walk 10m (±aid); excl: pain >4, MAS ≥3, severe comorbidities. | Training included 3 hours of progressive task-oriented lower limb shaping exercises (e.g., step-ups, knee extensions) and 0.5 hours of Transfer Package for daily life application. N physical restraints were used. | 210 min/session, 5×/week | 2weeks | A groups: 3 h × 5 × 4.0 = 600 0.5 h × 5 × 2.0 = 50 Total = 650 METs-min/week | 6MWT 10MWT BBS FMA-LE |
|  |  | Middle- and high-income | 19 | Lower limb | 15I + 4H | Chronic Phase | Moderate | 59.3±11.4 | M9/F10 | N | RC |  | Participants received conventional rehabilitation including ROM, stretching, gait, balance, and endurance training. N shaping technique or behavioral component was included. | 210 min/session, 5×/week | 2weeks | B groups: 0.5 h × 5 × 2.0 = 50 Total = 50 METs-min/week |  |
| 209 | Qandeel Yousaf, 2024^284^ | Low- and middle-income | 24 | Trunk and lower limb function | I | Chronic Phase | Moderate to severe | 45±4.92 | M22/F2 | N | NPF | Unilateral I stroke (CT/MRI); ≥6mo; excl: severe cognitive, recurrent, brainstem/cerebellar/H stroke, MAS ≥3, hypotonia. | Conducted rhythmic initiation, slow reversal, and agonist reversal techniques in sessions focusing on trunk motor control. | 30 min/session, 5×/week | 6weeks | 30min × 5 × 3.5 METs = 525 METs-min/week | BBS |
|  |  | Low- and middle-income | 24 | Trunk and lower limb function | I | Chronic Phase | Moderate to severe | 45±4.92 | M17/F7 | N | CST |  | Performed core stability training including quadruped arm/leg raises and supine curl-ups with diaphragmatic breathing. | 30 min/session, 5×/week | 6weeks | 30min × 5 × 3.8 METs = 570 METs-min/week |  |
| 210 | Hye-Jin Lee, 2018^285^ | High income | 10 | Trunk and lower limb function | 9H + 1I | Chronic Phase | Moderate | 59.80±6.92 | M5/F5 | N | RT | CT/MRI hemiplegic stroke; 50–70y; K-MMSE ≥24; 10MWT 5–60s; consented. | Inspiratory muscle training with resistance devices and abdominal bracing using pressure feedback to enhance trunk control. | 40 min/session (split into 2×20 min), 5×/week | 6weeks | Breathing Resistance Training: 20 × 5 × 2.0 = 200 Bracing Core Training: 20 × 5 × 2.5 = 250 Total = 450 METs-min/week | 6MWT 10MWT BBS |
|  |  | High income | 10 | Trunk and lower limb function | 9H + 1I | Chronic Phase | Moderate | 60.20±8.24 | M5/F5 | N | NPF |  | Performed general rehabilitation including mat exercises, NDT, and balance training without respiratory muscle strengthening. | 40 min/session (split into 2×20 min), 5×/week | 6weeks | General Training: 40 × 5 × 3.0 = 600 METs-min/week |  |
| 211 | Shangjun Huang, 2019^286^ | Middle- and high-income | 14 | Trunk and lower limb function | 9H + 5I | Chronic Phase | Moderate | 62.21±9.74 | 12M/2F | Y | MBE | CT/MRI stroke; fear of falling; 30–75y; MMSE >24; stand/walk independently; no Tai Chi/other training; no major impairments. | Performed body-weight supported gait training combined with simplified Tai Chi steps to improve dynamic balance and postural control. | 40 min/session, 3×/week | 12weeks | Experimental Group: Conventional: 40 × 3 × 3.0 = 360 BWS-TC Tai Chi: 40 × 3 × 3.5 = 420 Total = 780 METs-min/week | FMA-LE |
|  |  | Middle- and high-income | 14 | Trunk and lower limb function | 8H + 6I | Chronic Phase | Moderate | 59.93±9.96 | 10M/ 4F | N | NPF |  | Conducted conventional therapy including Bobath, proprioceptive training, sit-to-stand, and gait exercises without Tai Chi or suspension. | 40 min/session, 3×/week | 12weeks | Control Group: 40 × 3 × 3.0 = 360 METs-min/week |  |
| 212 | Won Seob Shin, 2011^287^ | High income | 11 | Trunk and lower limb function | Mixed | Chronic Phase | Moderate | 58.1±4.6 | M5/F6 | Y | FT | CT/MRI confirmed stroke with unilateral lower limb hemiparesis; exclusions: arthritis, LBP, DJD, cognitive/language limitations. | CE group: Conducted 30 minutes of functional strengthening (bridging, sit-to-stand, stair climbing) followed by 30 minutes of aerobic treadmill and cycling. | 60 min/session, 5×/week | 4weeks | Conventional: 30 × 5 × 3.5 = 525 Additional: 30 × 5 × 6.0 = 750 Total = 1275 METs-min/week | BBS |
|  |  | High income | 10 | Trunk and lower limb function | Mixed | Chronic Phase | Moderate | 57.3±4.4 | M3/F7 | Y | RC |  | CT group: Received conventional therapy focusing on postural control, gait phase training, and upper limb scapular control. | 60 min/session, 5×/week | 4weeks | Conventional only: 60 × 5 × 3.5 = 1050 METs-min/week |  |
| 215 | Susanne Palmcrantz, 2025^288^ | High income | 12 | Lower limb | 10I + 2H | Chronic Phase | Moderate | 65±10 | 8 M/ 4F | Y | RAT | Chronic stroke with hemiplegia; FAC score 2–4; ambulatory. | Received robot-assisted gait training using HAL exoskeleton, emphasizing progressive increase in step speed and reduction in assistance. | 30min/session,3×/week | 6weeks | HAL Group: HAL: 45 × 3 × 5.0 = 675 Rehab: 30 × 3 × 3.0 = 270 Total = 945 METs-min/week | 6MWT |
|  |  | High income | 15 | Lower limb | 8I + 7H | Chronic Phase | Moderate | 66±10 | 9M/6F | N | GT |  | Underwent conventional therapy including ground walking, treadmill, stair and slope training. | 30min/session,3×/week | 6weeks | Traditional Gait Training: 60 × 3 × 3.5 = 630 METs-min/week |  |
|  |  | High income | 11 | Lower limb | 8I + 3H | Chronic Phase | Moderate | 60±14 | 10 M/1F | Y | RC |  | Continued daily activities only, withweekly follow-up. | 30min/session,3×/week | 6weeks | Control (minimal rehab): 30 × 2 × 3.0 = 180 METs-min/week |  |
| 216 | Jie Shen，2023^289^ | Middle- and high-income | 20 | Trunk and lower limb function | 10I + 10H | Subacute Phase | Moderate | 57.40±7.70 | M16/F4 | Y | CST | First-ever I or H stroke; confirmed by CT/MRI. | Trained on a dynamic unstable platform requiring postural balance maintenance during programmed multidirectional board motion. | 50 min/session, 5×/week | 8weeks | DMIST Group: Conventional: 30 × 5 × 3.0 = 450 DMIST: 20 × 5 × 3.5 = 350 Total = 800 METs-min/week | BBS FMA-LE |
|  |  | Middle- and high-income | 20 | Trunk and lower limb function | 8I + 12H | Subacute Phase | Moderate | 56.10±7.99 | M13/F7 | Y | BT |  | Performed conventional balance training using rods, rollers, and proprioceptive tasks. | 50 min/session, 5×/week | 8weeks | Control Group (General Balance Training): Same structure and total = 800 METs-min/week |  |
| 217 | Jaehong Shim，2020^290^ | High income | 17 | Trunk | 11I + 6H | Chronic Phase | Moderate | 59.65±16.52 | 10M/7F | N | ESX | First-ever I or H stroke; confirmed by CT/MRI. | PNF trunk pattern training synchronized with EMG-triggered FES targeting abdominal and back muscles to induce co-contractions. | 30 min/session, 5×/week | 4weeks | Experimental (PNF + FES): 30 min × 5 × 6.5 METs = 975 METs-min/week | BBS |
|  |  | High income | 16 | Trunk | 9I + 7H | Chronic Phase | Moderate | 56.00±15.61 | 9M/7F | N | NPF |  | Received same PNF protocol without electrical stimulation, focusing on guided trunk movement and isotonic contractions. | 30 min/session, 5×/week | 4weeks | Control (PNF): 30 min × 5 × 3.0 METs = 450 METs-min/week |  |
| 219 | Patrícia Graef，2016^291^ | Middle- and high-income | 13 | Upper limb | Mainly I | Chronic Phase | Mild to moderate | 72±12 | M6/F7 | Y | FT | Hospital stroke; MMSE ≥20; strength-based inclusion; no shoulder contracture/pain. | Performed functional strength tasks such as lifting and placing containers using 60% max resistance, guided by therapists. | 30 min/session, 3×/week | 5weeks | FS Group (Functional Strength): 30 min × 3 × 3.5 = 315 Passive ROM (10 min × 3 × 2.5) = 75 Total = 390 METs-min/week | FMA-UE |
|  |  | Middle- and high-income | 14 | Upper limb | Mainly I | Chronic Phase | Mild to moderate | 63±11 | M5/F9 | Y | RT |  | Conducted Nn-functional shoulder exercises (abduction, flexion) using dumbbells at 60% max effort. | 30 min/session, 3×/week | 5weeks | Analytical Resistance Group: Same calculation = 390 METs-min/week |  |
| 220 | Hang Yang，2024^292^ | Middle- and high-income | 20 | Lower limb | Mainly I | Subacute Phase | Mild to moderate | 49.85±8.44 | M55%/F45% | N | VRG | Stroke; walk independently/assisted (FAC ≥3); MMSE NR (able to follow instructions). | Received AR-enhanced treadmill gait adaptation training with modules including target stepping, obstacle crossing, and variable speeds. | 35 min/session, 3×/week | 5weeks | Group Circuit Training: 35 min × 3 × 6.0 = 630 METs-min/week | 10MWT BBS |
|  |  | Middle- and high-income | 20 | Lower limb | Mainly I | Subacute Phase | Mild to moderate | 50.75±8.05 | M65%/F35% | N | GT |  | Used same treadmill without AR modules, doing walking only. | 35 min/session, 3×/week | 5weeks | Treadmill + Ground Walking Group: Warm-up (4.5 min × 3.5 METs) = 15.75 Treadmill (15 × 4.3) = 64.5 Ground walk (15 × 3.5) = 52.5 Per session = 132.75 × 3 = ~400 METs-min/week |  |
| 221 | Momna Asghar，2023^293^ | Low- and middle-income | 30 | Lower limb | 53%H + 47%I | Chronic Phase | Mild to moderate | 54±9.5 | M17/F13 | N | NPF | Stroke confirmed by neurologist (I or H); MMSE ≥ 24; able to follow simple commands. | Integrated PNF pelvic and lower limb patterns into walking training combined with conventional stretching and strength exercises. | 50 min/session, 3×/week | 6weeks | 50 × 3 × 5.0 = 750 METs-min/week | BBS |
|  |  | Low- and middle-income | 30 | Lower limb | 47%H + 40%I | Chronic Phase | Mild to moderate | 53±9.4 | M15/F15 | N | RC |  | Received same conventional therapy but without PNF elements, using standard gait and bridging tasks. | 40 min/session, 3×/week | 6weeks | 40 × 3 × 4.0 = 480 METs-min/week |  |
| 225 | Ruoxin Zhao，2022^294^ | Middle- and high-income | 20 | Trunk and lower limb function | 14I + 6H | Subacute Phase | Mild to moderate | 60.4±12.32 | M15/F5 | Y | BT | CT/MRI confirmed I/H stroke; MMSE > 23; able to walk ≥3 m (assistive device allowed). | Performed gaze stability training in standing using VOR-based tasks (saccades, pursuit, vestibular compensation) after PT. | 60 min/session, 5×/week | 4weeks | GSE Group: Rehab: 30 × 5 × 4.0 = 600 GSE: 30 × 5 × 2.8 = 420 Total = 1020 METs-min/week | BBS |
|  |  | Middle- and high-income | 20 | Trunk and lower limb function | 12I + 8H | Subacute Phase | Mild to moderate | 54.45±13.94 | M15/F5 | Y | RC |  | Received same physical therapy without gaze stability component. | 30 min/session, 5×/week | 4weeks | Gait Training Group: Rehab: 30 × 5 × 4.0 = 600 Gait: 30 × 5 × 4.0 = 600 Total = 1200 METs-min/week |  |
| 226 | Schachten，2015^295^ | High income | 7 | Trunk and lower limb function | Mainly I | Chronic Phase | Mild | 55.14±17.64 | Nt reported | Y | MBE | Stroke (hospital-confirmed); recruited; no severe complications; able to follow instructions. | Participated in indoor golf activities (swinging, putting) integrated with cognitive tasks for dual-task coordination. | 60 min/session, 2×/week | 10weeks | Golf Group: Rehab: 60 × 2 × 3.0 = 360 Golf: 60 × 2 × 3.5 = 420 Total = 780 METs-min/week | BBS |
|  |  | High income | 7 | Trunk and lower limb function | Mainly I | Chronic Phase | Mild | 53.14±13.54 | Nt reported | Y | RC |  | Attended social activities including reading, discussion, and memory games for cognitive stimulation. | 60 min/session, 2×/week | 10weeks | Social Communication Group: Rehab: 60 × 2 × 3.0 = 360 Social: 60 × 2 × 1.5 = 180 Total = 540 METs-min/week |  |
| 227 | Junghwa Do,2024^296^ | High income | 11 | Lower limb | 10I + 1H | Chronic Phase | Mild to moderate | 61.8±7.3 | M7/F4 | N | ESX | Stroke(CT/MRI);MMSE ≥20; ≤135 kg; Morning Walk®eligible; excl. cognitive/psychiatric/MSK. | Underwent high-intensity interval training using Morning Walk® robotic system with VR and task-based walking scenarios. | 40 min/session, 3×/week | 8weeks | HIIT Robotic Training: Warm-up: 10 × 3 × 2.5 = 75 HIIT: 20 × 3 × 7.0 = 420 Total = 495 METs-min/week | 10MWT BBS FMA |
|  |  | High income | 11 | Lower limb | 7I + 4H | Chronic Phase | Mild to moderate | 63.5±8.1 | M8/F3 | N | ESX |  | Received home exercise instruction and outpatient therapy twiceweekly with activity logs. | ~30 min/session, ~3×/week | 8weeks | Routine + Home: Routine: 45 × 2 × 4.0 = 360 Home: 60 × 1 × 3.5 = 210 Total = 570 METs-min/week |  |
| 228 | Tor Ivar Gjellesvik，2021^297^ | High income | 36 | Lower limb | 29I + 7H | Chronic Phase | Mild to moderate | 57.6±9.2 | M21/F15 | N | NPFCST | First stroke (I/H, CT/MRI); mRS 0–3; no cardiac/HTN/VO₂ issues; HIIT eligible; brainstem/SAH excl. | Completed 8-week high-intensity interval treadmill walking protocol with 4×4 min high-load bouts interspersed with active recovery. | 40 min/session, 3×/week | 8weeks | HIIT Training: Warm-up: 10 × 3 × 3.0 = 90 High intensity: 16 × 3 × 9.0 = 432 Active recovery: 9 × 3 × 3.5 = 94.5 Total ≈ 617 METs-min/week | 6MWT 10MWT BBS |
|  |  | High income | 34 | Lower limb | 28I + 6H | Chronic Phase | Mild to moderate | 58.7±9.2 | M20/F14 | N | NPFULT |  | Received usual care and general activity advice without structured exercise. | Nn-fixed frequency, revisit at testing time | 8weeks | Low-Intensity Control: 30 × 3 × 2.5 = 225 METs-min/week |  |
| 229 | Tayyaba Zaman，2025^298^ | Middle income | 17 | Lower limb | I | Chronic Phase | Moderate | 48.23±4.94 | M8/F9 | Y | HIIT | First I stroke (CT/MRI); MMSE >24; AHA class B; cleared for HIIT; walk 10 m (aided); no spasticity/implants/recent hospitalization. | Conducted treadmill HIIT followed by ground-based intervals and stretching. | 45–50 min/session, 3×/week | 4weeks | HIIT Combo: Treadmill: 20 × 3 × 6.0 = 360 Ground HIIT: 10 × 3 × 5.0 = 150 Warm-up/Cool-down: 6 × 3 × 2.5 = 45 Routine: 30 × 3 × 3.0 = 270 Total = 825 METs-min/week | 6MWT 10MWT |
|  |  | Middle income | 17 | Lower limb | I | Chronic Phase | Moderate | 50.52±4.30 | M10/F7 | N | CST |  | Performed conventional therapy with stretching and functional movement without HIIT. | ~30 min/session, 3×/week | 4weeks | Routine Only: 30 × 3 × 3.0 = 270 METs-min/week |  |
| 230 | Chae-Woo Lee，2014^299^ | High income | 15 | Lower limb | Mainly I | Chronic Phase | Mild to moderate | 63.8±6.2 | M11/F4 | N | FT | Stroke (CT/MRI); MMSE-K >24; MAS ≤2 (lower limb); able to train >30 min | Rode horses in circular paths to stimulate rhythmic trunk and postural control. | 30 min/session, 3×/week | 8weeks | Horseback Riding: 30 × 3 × 5.5 = 495 METs-min/week | BBS |
|  |  | High income | 15 | Lower limb | Mainly I | Chronic Phase | Mild to moderate | 64.3±4.8 | M12/F3 | N | GT |  | Trained on treadmill with speed progression, mimicking same duration. | 30 min/session, 3×/week | 8weeks | Treadmill: 30 × 3 × 3.5 = 315 METs-min/week |  |
| 231 | Hiroyuki Ase，2025^300^ | High income | 7 | Upper limb | 4I + 3H | Chronic Phase | Moderate | 58.0±6.4 | 6M1F | Y | VRG | Unilateral stroke (CT/MRI); MRC ≥2 (UE); Mini-Cog normal; MAS <3; no aphasia/skin/neurological hx | Used VR-based upper limb training with real-time feedback and task simulations like pouring and grasping. | 30 min/session, 5×/week | 4weeks | VR Group: 30 × 5 × 3.0 = 450 OT (30 × 1 × 3.0) = 90 Total = 540 METs-min/week | FMA-UE |
|  |  | High income | 7 | Upper limb | 1I + 6H | Chronic Phase | Moderate | 51.4±6.7 | 6M1F | Y | RC |  | Followed therapist-prescribed repetitive joint exercises based on daily task goals. | 30 min/session, 5×/week | 4weeks | ROM Arm Exercise Group: 30 × 5 × 2.5 = 375 OT (30 × 1 × 3.0) = 90 Total = 465 METs-min/week |  |
| 232 | Jungseo Park，2013^301^ | High income | 34 | Trunk | 15I + 19H | Chronic Phase | Mild to moderate | 56.09±7.22 | M18/F16 | Y | FT | Stroke (CT/MRI); K-MMSE ≥24; MAS ≤2; hand strength ≥F; stand ≥30 sec, walk ≥30 m; no DM/cardiac/orthopedic disorders. | Participants trained on the FORTIS horseback simulator, including two 15-minute programs with varying intensity and movement patterns to simulate real horseback riding. | 35 min/session, 3×/week | 8weeks | Horse Simulator Group: Routine rehab: 30 × 6 × 3.0 = 540 Horse simulation: 30 × 3 × 5.5 = 495 Total = 1035 METs-min/week | BBS |
|  |  | High income | 33 | Trunk | 16I + 17H | Chronic Phase | Mild to moderate | 51.55±8.27 | M18/F15 | Y | CST |  | Participants performed core stabilization exercises on the floor based on Nrris and Richardson’s protocols, including pelvic tilts, trunk movements, and supine lower-limb tasks. | 35 min/session, 3×/week | 8weeks | Mat Stability Group: Routine rehab: 540 Mat training: 35 × 3 × 3.5 = 368 Total = 908 METs-min/week |  |
| 233 | Yong-nam Kim，2015^302^ | High income | 10 | Lower limb | Mainly I | Chronic Phase | Mild to moderate | 71.1±3.0 | M5/F5 | Y | FT | MRIconfirmed stroke; MMSE > 24; able to walk ≥10 m independently; excluded if visual or orthopedic lower limb issues. | Participants trained with the JOBA EU7800 horseback simulator, involving simulated multidirectional horse movements with gradually increasing difficulty. | 30 min/session, 5×/week | 6zhou | Experimental Group: Rehab: 30 × 5 × 4.0 = 600 Horse simulator: 30 × 5 × 5.5 = 825 Total = 1425 METs-min/week | 10MWT BBS |
|  |  | High income | 10 | Lower limb | Mainly I | Chronic Phase | Mild to moderate | 69.2±3.4 | M5/F5 | Y | NPF |  | Received conventional neurodevelopmental therapy including sitting, standing, transfer, and gait training. | 30 min/session, 5×/week | 6zhou | Control Group: Rehab only: 600 METs-min/week |  |
| 234 | Lina Bunketorp-Käll，2019^303^ | High income | 41 | Whole-body function | 34.1%H + 65.9%I | Chronic Phase | Mild to moderate | 62.6±6.5 | F 41.5%/M 58.5% | Y | FT | Stroke (CT/MRI); mRS 2–3; NIHSS 2.7–3.0; Swedish comprehension; no cognitive/language/vision, cardiac, epilepsy. | Participants received real horseback riding therapy guided by certified instructors, focusing on postural control and vestibular stimulation. | 45 min/session, 2×/week | 12weeks | H-RT (Horse-Riding Therapy): Horse riding: 60 × 2 × 4.5 = 540 Rehab: 30 × 2 × 3.0 = 180 Total = 720 METs-min/week | 6MWT 10MWT |
|  |  | High income | 41 | Whole-body function | 22.0%H + 78.0%I | Chronic Phase | Mild to moderate | 62.7±6.7 | F 43.9%/M 56.1% | Y | MBE |  | Participants engaged in rhythmic movement to music involving repeated upper/lower limb tasks and balance exercises. | 45 min/session, 2×/week | 12weeks | R-MT (Rhythm + Music): Music training: 60 × 2 × 3.0 = 360 Rehab: 180 Total = 540 METs-min/week |  |
|  |  | High income | 41 | Whole-body function | 32.4%H + 67.6%I | Chronic Phase | Mild to moderate | 63.7±6.7 | F 48.6%/M 51.4% | N | RC |  | Did Nt receive training during the first 12weeks, then switched to the R-MT group. | 30 min/session, 2×/week | 12weeks | Control: Rehab only = 180 METs-min/week |  |
| 235 | Klas Sandberg，2020^304^ | High income | 23 | Whole-body function | I | Acute Phase | Moderate to severe | 72.1±11.7 | M 8 /F 15 | Y | AE | First stroke (CT/MRI); NIHSS 7–42; aerobic training eligible; understands; non-thrombolysis. | Bedside motorized cycling training using MOTOmed Letto2 in the supine position, starting 24–48h post-stroke with passive or active pedaling depending on ability. | 20 min/session, 5×/week | 3weeks | Experimental Group: Cycling: 20 × 5 = 100 × 3.5 = 350 Extended in study: total = 1050 Rehab: 30 × 5 × 3.0 = 450 Total = 1500 METs-min/week | 6MWT |
|  |  | High income | 29 | Whole-body function | 79.3%I + 20.7%H | Acute Phase | Moderate to severe | 76.3±6.4 | M 12/F 17 | Y | RC |  | Received routine acute stroke rehabilitation including bedside transfers, sitting, standing, and walking. | Nn-fixed daily physical therapy | 3weeks | Control: Rehab only: 450 METs-min/week |  |
| 236 | Mudassar Ali，2020^305^ | Middle-income | 11 | Whole-body function | Nt reported | Subacute Phase | Moderate | 60.81 | Nt reported | N | MBE | Stroke (CT/MRI); MMSE >24; walk/stand with aids; excl. severe motor/cognitive impairment. | Performed task-oriented circuit training at five stations (e.g., sit-to-stand, multidirectional stepping, reaching, obstacle crossing). | 50 min/session, 3×/week | 6weeks | Group Circuit: 50 × 3 × 4.0 = 600 METs-min/week | 10MWT |
|  |  | Middle-income | 22 | Whole-body function | Nt reported | Subacute Phase | Moderate | 60.81 | Nt reported | N | TOT |  | Performed the same content as group training but in a one-on-one setting. | 50 min/session, 3×/week | 6weeks | Same-task Individual: 600 METs-min/week |  |
| 237 | Soo-Yeon Kim，2015^306^ | High income | 13 | Lower limb | Mixed | Subacute Phase | Mild to moderate | 62.4±6.1 | overall:M 22/F30 | Y | RAT | First stroke (CT/MRI); MAS ≤2; no tremor/cognitive/visual/MSK/cardio-pulm. disease; weight ≤135 kg; height ≥150 cm; post-gait restoration phase. | Combined conventional gait training with robotic walking using WALKBOT, with dynamic weight support and adjustable hip/knee guidance. | 40 min/session ×2/day (80 min/day), 5×/week | 4weeks | WALKBOT Group: Robot: 40 × 5 × 6.5 = 1300 Rehab: 40 × 5 × 4.0 = 800 Total = 2100 METs-min/week | BBS |
|  |  | High income | 13 | Lower limb | Mixed | Subacute Phase | Mild to moderate | 63.5±6.3 | overall:M 22/F30 | Y | RC |  | Received conventional therapy including balance, sit-to-stand, walking, and resistance training. | 40 min/session ×2/day (80 min/day), 5×/week | 4weeks | Control Group: Rehab: 80 × 5 × 4.5 = 1800 METs-min/week |  |
| 238 | Bora An，2020^307^ | High income | 18 | Lower limb | 14I 14人 + 4H | Chronic Phase | Moderate | 54.77±10.55 | 11M/7F | Y | GT | First stroke (CT/MRI); K-MMSE ≥24; FAC ≥3; gait training eligible; excl. orthopedic deformities/spasticity/neurological disorders. | Treadmill training with a 10mm EVA insole on the Nn-paretic side to shift weight to the paretic side; treadmill speed was gradually increased. | 30 min/session, 5×/week | 4weeks | Incline Group: Treadmill: 30 × 5 × 4.5 = 675 Rehab: 30 × 5 × 3.0 = 450 Total = 1125 METs-min/week | 10MWT |
|  |  | High income | 18 | Lower limb | 13I 13人 + 5H | Chronic Phase | Moderate | 55.11±8.99 | 12M/6F | Y | AE |  | Identical treadmill training without the insole modification. | 30 min/session, 5×/week | 4weeks | Flat Group: Treadmill: 30 × 5 × 4.0 = 600 Rehab: 450 Total = 1050 METs-min/week |  |
| 239 | Lorena de Oliveira Vaz，2021^308^ | Middle- and high-income | 23 | Whole-body function | 61%I + 39%H | Chronic Phase | Mild to moderate | 52±13 | M10/F13 | Y | RT | Stroke (CT/MRI); MIP <-80 cmH₂O; MMSE by education; ambulatory (±aid); excl. smoker, facial paralysis, comorbidities. | Inspiratory muscle training using Powerbreathe Medic Plus at 50% of maximum inspiratory pressure; participants transitioned to home-based training after initial supervision. | 15 min/session ×2/day (30 min/day), 5×/week | 6weeks | IMT + Routine Rehab:  IMT: 30 × 5 = 150 min × 2.5 = 375 Aerobic/functional: 155 × 3.5 = 542.5 Resistance: 80 × 4.0 = 320 Total = 1237.5 METs-min/week | 6MWT |
|  |  | Middle- and high-income | 27 | Whole-body function | 54%I + 46%H | Chronic Phase | Mild to moderate | 56±9 | M11/F16 | Y | RC |  | Performed “sham” IMT using devices with minimal resistance and identical rehabilitation plans. | 15 min/session ×2/day (30 min/day), 5×/week | 6weeks | Sham IMT + Routine Rehab: IMT: 150 × 1.5 = 225 Routine rehab (same as above): 862.5 Total = 1087.5 METs-min/week |  |
| 241 | Umair Ahmed，2021^309^ | Low- and middle-income | 42 | Trunk | 27I + 15H | Chronic Phase | Mild to moderate | 61.21±7.78 | M20/F22 | Y | TOT | Stroke (clinical/imaging); no cerebellar/basal ganglia balance disorders; walk ≥10 m; MMSE ≥24; BMI ≤31 | High-intensity multidirectional trunk training combined with dual-task challenges and external resistance, guided by experienced therapists. | 45 min/session, 5×/week | 12weeks | HIMTD group (High-intensity multidirectional dual-task): Conventional rehabilitation: 30 min × 5/week × 3.0 METs = 450 METs-min/week HIMTD intervention: 15 min × 5/week × 5.5 METs = 412.5 METs-min/week Total = 450 + 412.5 = 862.5 ≈ 863 METs-min/week | 10MWT BBS |
|  |  | Low- and middle-income | 42 | Trunk | 24I + 18H | Chronic Phase | Mild to moderate | 62.21±8.20 | M17/F25 | Y | CST |  | Performed conventional sagittal-plane trunk exercises at moderate intensity without dual-task components. | 45 min/session, 5×/week | 12weeks | SCTR group (Standard care + task-specific rehab): Conventional rehabilitation: 30 min × 5/week × 3.0 METs = 450 METs-min/week SCTR training: 15 min × 5/week × 4.0 METs = 300 METs-min/week Total = 750 METs-min/week |  |
| 243 | Timur Ekiz，2015^310^ | Middle- and high-income | 12 | Lower limb | 8I + 4H | Subacute and chronic | Moderate | 48.8±12.9 | M5/F7 | Y | TCMEX | Stroke (CT/MRI); Brunnstrom LL 4–6; FAC ≥3; MAS ≤1; no brainstem/cerebellar stroke, cardioresp./language/orthopedic issues. | Received kinesiology taping on bilateral quadriceps using “Y” technique during routine rehab; tape was reapplied every 3–7 days. | 60 min/session, 5×/week | 4weeks | Conventional rehabilitation: 60 min × 5/week × 3.0 METs = 900 METs-min/week KT taping: 20 min × 5/week × 2.5 METs = 250 METs-min/week Total = 1150 METs-min/week | 6MWT 10MWT BBS |
|  |  | Middle- and high-income | 12 | Lower limb | I | Subacute and chronic | Moderate | 50.9±12.7 | M7/F5 | N | RC |  | Received routine rehabilitation only, without taping. | 60 min/session, 5×/week | 4weeks | Conventional rehabilitation: 60 min × 5/week × 3.0 METs = 900 METs-min/week |  |
| 246 | Wonjae Choi，2023^311^ | High income | 15 | Trunk | 11 I + 4H | Chronic Phase | Mild to moderate | 60.80±6.72 | M9/F6 | Y | TCMEX | Stroke >6 mo (CT/MRI); no dementia; walk ≥10 m independent; excl. fractures, deformities, visual/hearing/dysphagia/vestibular/Parkinsonism. | Received lumbar joint mobilization using Gape technique on L1–L5 segments in side-lying position, targeting rotation, side flexion, and extension. | 15 min/session, 5×/week | 6weeks | Conventional rehabilitation: 60 min × 5/week × 3.0 METs = 900 METs-min/week Joint mobilization therapy: 15 min × 5/week × 2.5 METs = 187.5 METs-min/week Total = 1087.5 METs-min/week | 10MWT BBS |
|  |  | High income | 15 | Trunk | 12 I + 3H | Chronic Phase | Mild to moderate | 60.87±6.64 | M11/F4 | N | NPF |  | Same position and therapist contact, but N manual force applied. | 15 min/session, 5×/week | 6weeks | Conventional rehabilitation: 60 min × 5/week × 3.0 METs = 900 METs-min/week |  |
| 247 | Dong-Hoon Kim，2021^312^ | High income | 20 | Lower limb | 11I + 9H | Chronic Phase | Mild to moderate | 56.05±14.43 | M12/F8 | Y | ESX | Stroke >6 mo (CT/MRI); K-MMSE ≥24; no visual/hearing issues; walk ≥10 m independent; excl. neglect, somatosensory/MSK/degenerative disease. | Performed mirror therapy with simultaneous EMG-triggered FES on the paretic leg during knee and ankle exercises, with standard rehab. | 60 min/day (30+30), 5×/week | 8weeks | Mirror + EMG-FES group: Mirror + EMG-FES: 30 min × 5/week × 3.5 METs = 525 METs-min/week Conventional rehab: 30 min × 5/week × 3.0 METs = 450 METs-min/week Total = 975 METs-min/week | 10MWT BBS |
|  |  | High income | 20 | Lower limb | 5I + 7H | Chronic Phase | Mild to moderate | 57.45±5.27 | M9/F11 | Y | MBE |  | Same mirror therapy without FES. | 60 min/day (30+30), 5×/week | 8weeks | Mirror-only group: Mirror therapy: 30 min × 5/week × 2.5 METs = 375 METs-min/week Conventional rehab: 30 min × 5/week × 3.0 METs = 450 METs-min/week Total = 825 METs-min/week |  |
|  |  | High income | 20 | Lower limb | 8I + 12H | Chronic Phase | Mild to moderate | 59.70±8.65 | M10/F10 | Y | NPF |  | Conventional rehab only, without mirror or FES. | 30 min/session ×2/day, 5×/week | 8weeks | Control group (standard therapy): 60 min × 5/week × 3.0 METs = 900 METs-min/week |  |
| 248 | Kyunghoon Kim，2016^313^ | High income | 12 | Upper limb | 66.7%H + 33.3%I | Chronic Phase | Moderate | 45.2±4.7 | M8/F4 | N | MBE | Stroke >6 mo (CT/MRI); MMSE ≥24; cooperative; excl. cognitive/vestibular/vision/orthopedic barriers. | Mirror placed at midline; participants performed 9 upper limb tasks using their unaffected arm while observing the mirror reflection. | 30 min/session, 5×/week | 4weeks | MT group (Mirror therapy + functional task): 30 min × 5/week × 3.0 METs = 450 METs-min/week | FMA-UE |
|  |  | High income | 13 | Upper limb | 69.2%H + 30.8%I | Chronic Phase | Moderate | 52.6±3.0 | M8/F5 | N | ULT |  | Performed 9 different upper limb tasks without mirror therapy. | 30 min/session, 5×/week | 4weeks | CT group (Conventional occupational therapy): 30 min × 5/week × 2.8 METs = 420 METs-min/week |  |
| 249 | Dae-Hyouk Bang，2015^314^ | High income | 9 | Upper limb | 6I + 3H | Subacute Phase | Moderate | 60.22±5.76 | M5/F4 | N | CIMT | Stroke (CT/MRI); UE ext: finger/MP/IP ≥10°, wrist ≥20°; MMSE ≥21; MAL <2.5; MAS ≤2; no comorbidities/uncontrolled health/other trials. | Intervention group received 1-hour upper limb intensive training with trunk restraint using Nn-elastic straps at the T7 level to limit compensatory trunk movements. A glove was used to constrain the unaffected limb for 5 hours daily. Training followed shaping and repetitive task practice principles and was accompanied by verbal cues. | 60 min/session + 5 h restraint, 5×/week | 4weeks | Task-oriented reaching with trunk restraint: 60 min × 5/week × 3.3 METs = 990 METs-min/week | FMA-UE |
|  |  | High income | 9 | Upper limb | 5I + 4H | Subacute Phase | Moderate | 59.33±8.20 | M4/F5 | N | ULT |  | Control group received identical upper limb training and glove constraint but without trunk restraint. | 60 min/session + 5 h restraint, 5×/week | 4weeks | Task-oriented reaching (without trunk restraint): 60 min × 5/week × 3.0 METs = 900 METs-min/week |  |
| 250 | Dae-Hyouk Bang，2018^315^ | High income | 12 | Upper limb | 8I + 4H | Subacute Phase | Moderate | 63.26±6.74 | M6/F6 | N | CIMT | First stroke (I/H, CT/MRI, 2–4 wk); wrist ext ≥20°, MP/IP ≥10°; MMSE ≥24; MAL <2.5; MAS <3; excl. major illness/rehab trials. | Intervention group underwent mCIMT training with trunk restraint using a strap at T7 and glove constraint on the unaffected side for 5 hours. Tasks included grasping, reaching, and stacking blocks. Task complexity was progressively increased. | 60 min/day + 5 h unaffected limb restraint, 5×/week | 4weeks | mCIMT + trunk restraint: 60 min × 5/week × 3.3 METs =990 METs-min/week | FMA-UE |
|  |  | High income | 12 | Upper limb | 7I + 5H | Subacute Phase | Moderate | 59.12±7.26 | M5/F7 | N | ULT |  | Control group received the same mCIMT protocol without trunk restraint. | 60 min/day + 5 h unaffected limb restraint, 5×/week | 4weeks | mCIMT standard (without trunk restraint): 60 min × 5/week × 3.0 METs = 900 METs-min/week |  |
| 251 | Zhiqiang Song，2018^316^ | Middle- and high-income | 75 | Lower limb | 49I + 26H | Subacute Phase | Moderate | 59.57±6.48 | M45/F30 | Y | CST | Stroke (CT/MRI + 4th NCCVD); limb dysfunction; no severe aphasia/cognitive/psychiatric illness; able to consent/cooperate. | Intervention group performed modified motor skill training focused on posture and gait, including sit-to-stand, balance, stair climbing, and progressive walking distance up to 1000 meters. | 45 min/session, 5×/week | 6weeks | Modified training group: Modified gait training: 60 min × 5/week × 4.5 METs = 1350 METs-min/week Conventional rehabilitation: 45 min × 5/week × 3.0 METs = 675 METs-min/week Total = 1350 + 675 = 2025 METs-min/week | FMA-LE |
|  |  | Middle- and high-income | 75 | Lower limb | 46I + 29H | Subacute Phase | Moderate | 61.84±7.02 | M34/F41 | N | NPF |  | Control group received conventional neurorehabilitation, including Bobath, Brunnstrom, Rood, and PNF techniques, without structured motor skill training. | 45 min/session, 5×/week | 6weeks | Control group: 45 min × 5/week × 3.0 METs = 675 METs-min/week |  |
| 252 | Fettah Saygili，2024^317^ | Middle- and high-income | 10 | Upper limb | 70%I + 30%H | Chronic Phase | Moderate | 39.5–61.75 | 8M/2F | Y | CIMT | First stroke (CT/MRI); MMSE ≥24; UE: wrist ≥20°, thumb ≥10°, fingers ≥10°; MAL-28 <2.5; stand ≥2 min; no MAS ≥2, VAS ≥4, comorbid rehab/illness. | Tele-CIMT group received 90-minute video-based mCIMT sessions with therapist supervision, combined with 5-hour daily constraint of the unaffected limb. Tasks were personalized and supported by shaping strategies and caregiver assistance. | 90 min/day, 5×/week | 3weeks | Tele-CIMT + home-based rehab: Tele-CIMT: 90 min × 5 × 3weeks = 1350 min Estimated METs = 3.8 → 1350 × 3.8 = 5130 METs-min/3weeks → 1710 METs-min/week Home rehab: 30 min × 5 × 3weeks = 450 min Estimated METs = 3.0 → 450 × 3.0 = 1350 METs-min/3weeks → 450 METs-min/week Total = 1710 + 450 = 2160 METs-min/week | FMA-UE |
|  |  | Middle- and high-income | 8 | Upper limb | 75%I + 25%H | Chronic Phase | Moderate | 42.0–63.25 | 6M/2F | N | RC |  | Control group followed a home program with active upper limb and gait exercises, guided by illustrated manuals and daily logs. | ~60 min/day, 5×/week | 3weeks | Control group: Home rehab only: 30 min × 5/week × 3.0 METs =450 METs-min/week |  |
| 253 | Hwi-young Cho，2012^318^ | High income | 15 | Lower limb | 12I + 3H | Chronic Phase | Moderate | 53.93±12.60 | 9M/6F | N | MBE | Chronic stroke (CT/MRI, ~45 mo); MMSE >24; walk ≥10 m; no visual/auditory, orthopedic, drug, neuro issues. | Intervention group performed 30 minutes of treadmill walking followed by 15 minutes of motor imagery combining visual and kinesthetic elements. | 45 min/session (30 min walking + 15 min imagery), 3×/week | 6weeks | Motor Imagery + Treadmill group: Treadmill training: 30 min × 3/week × 4.0 METs = 360 METs-min/week Motor imagery (seated): 15 min × 3/week × 1.5 METs = 67.5 METs-min/week Total = 360 + 67.5 = 427.5 METs-min/week | 10MWT FMA-LE |
|  |  | High income | 13 | Lower limb | 11I + 2H | Chronic Phase | Moderate | 53.85±12.44 | 8M/5F | N | AE |  | Control group performed treadmill walking only, at a comfortable self-selected speed. | 30 min/session, 3×/week | 6weeks | Treadmill only group: 30 min × 3/week × 4.0 METs = 360 METs-min/week |  |
| 254 | Xiao-Jun Yin，2022^319^ | Middle- and high-income | 16 | Lower limb | 10I + 6H | Subacute Phase | Moderate | 56.9±8.7 | M13/F3 | Y | MBE | Stroke (NCCVD/CT/MRI); Brunnstrom LL I–IV; Hamilton <24; KVIQ ≥25; MMSE ≥24 | MIT group received daily 20-minute audio-guided motor imagery training following 3-hour standard rehabilitation. Sessions included relaxation, lower-limb imagery (e.g., standing, walking), and cooldown. | 20 min/day, 5×/week | 6weeks | MIT group: Motor imagery training: 20 min × 5/week × 2.5 METs = 250 METs-min/week Conventional rehab: 180 min × 5/week × 3.0 METs = 2700 METs-min/week Total = 2950 METs-min/week | BBS FMA-LE |
|  |  | Middle- and high-income | 16 | Lower limb | 11I + 5H | Subacute Phase | Moderate | 57.1±8.4 | M12/F4 | N | RC |  | Control group received standard rehabilitation only. | 180 min/day, 5×/week | 6weeks | Control group: Conventional rehab only: 180 min × 5/week × 3.0 METs =2700 METs-min/week |  |
| 255 | Xi Luo，2024^320^ | Middle- and high-income | 32 | Lower limb | I | Acute Phase | Mild | ≤40 2，41–60 19，>60 11 | M23/F9 | Y | ESX | AIS (2018, CT/MRI); <2 wk, hemiplegia; Brunnstrom II–IV; sitting ≥1; MMSE ≥21; NIHSS ≤15; no comorbidity, epilepsy, craniectomy,hemianopia, implants, DVT | BCI group received two daily sessions using brain–computer interface for motor imagery tasks (e.g., ankle dorsiflexion, knee extension), triggering FES when EEG thresholds were met. | 60 min/day (2×30 min), 5×/week | 2weeks | BCI + conventional rehab group: BCI training: 60 min/day × 5/week × 4.0 METs = 1200 METs-min/week Conventional rehab: 30 min × 5/week × 3.0 METs = 150 min × 3.0 = 450 METs-min/week Total = 1650 METs-min/week | FMA-LE |
|  |  | Middle- and high-income | 32 | Lower limb | I | Acute Phase | Mild | ≤40 1人，41–60 15，>60 16 | M19/F13 | N | RC |  | Control group received physical therapy and acupuncture as standard care. | 70 min/day, 5×/week | 2weeks | Acupuncture + physical therapy group: 40 min (PT) + 30 min (acupuncture) × 5/week = 70 min × 5 Estimated at 3.0 METs → 350 min × 3.0 = 1050 METs-min/week |  |
| 256 | Rakesh Shrestha，2022^321^ | Middle income | 12 | Lower limb | Mixed | Chronic Phase | Moderate | 51.33±11.84 | M6/F6 | Y | MBE | Stroke (CT/MRI); MMSE >21; stand ≥30s, walk >3 m; follows; no vestibular/visual/cardiac/neuro/drug balance issues. | Intervention group received additional multi-directional stepping and weight-shifting tasks during therapy. Training used mirrors and required holding positions for 10 seconds. | 60 min/session (including 15 min MSE), 5×/week | 4weeks | MSE + weight shift training group: Weight-shifting: 15 min × 4/week × 3.5 METs = 210 METs-min/week Conventional rehab: 45 min × 4/week × 3.0 METs = 540 METs-min/week Total = 750 METs-min/week | BBS |
|  |  | Middle income | 12 | Lower limb | Mixed | Chronic Phase | Moderate | 62.16±8.88 | M6/F6 | N | RC |  | Control group performed standard therapy including wall-supported walking, mat-based exercises, stretching, and ROM training. | 45 min/session, 5×/week | 4weeks | Control group: Conventional rehab: 45 min × 4/week × 3.0 METs = 540 METs-min/week |  |
| 257 | Gi-Deok Park，2016^322^ | High income | 20 | Lower limb | Mixed | Chronic Phase | Moderate | 54.1±14.2 | M11/F9 | Y | AE | Stroke >6 mo (CT/MRI); K-MMSE ≥25; walk ≥10 m; follows/consent; no cognitive/sensory disorders. | Intervention group performed loaded weight-shifting and step training using raised platforms and obstacle stepping with the unaffected leg, while the affected leg remained stationary. | 30 min/session, 5×/week | 4weeks | Multidirectional stepping group: Stepping training: 30 min × 5/week × 3.5 METs = 525 METs-min/week Conventional rehab: 30 min × 5/week × 3.0 METs = 450 METs-min/week Total = 975 METs-min/week | 10MWT BBS |
|  |  | High income | 20 | Lower limb | Mixed | Chronic Phase | Moderate | 63.4±9.3 | M12/F8 | N | RC |  | Control group performed conventional balance and gait training. | 30 min/session, 5×/week | 4weeks | Control group: Conventional rehab only: 30 min × 5/week × 3.0 METs = 450 METs-min/week |  |
| 258 | Jin-Uk Choi，2015^323^ | High income | 10 | Lower limb | Mixed | Chronic Phase | Moderate | 61.5±7.2 | M4/F6 | Y | TOT | Stroke >6 mo (CT/MRI); K-MMSE ≥25; MAS ≤2; trains; consented; no motor/cognitive impairment. | Task-oriented training group selected daily ADL tasks (e.g., dressing, stairs) based on personal goals and performed them stepwise with therapist guidance. | 30 min/day, 5×/week | 4weeks | Task-oriented mobility training group: Functional tasks (walking, stairs, dressing): 30 min × 5/week × 3.5 METs = 525 METs-min/week Conventional rehab: 30 min × 5/week × 3.0 METs = 450 METs-min/week Total = 975 METs-min/week | BBS |
|  |  | High income | 10 | Lower limb | Mixed | Chronic Phase | Moderate | 66.4±9.3 | M4/F6 | N | RC |  | Control group received general gait and balance exercises designed by therapists. | 30 min/day, 5×/week | 4weeks | Control group: Conventional rehab only: 30 min × 5/week × 3.0 METs = 450 METs-min/week |  |
| 259 | Lavnika Dubey，2018^324^ | Middle income | 13 | Trunk and lower limb function | 13I + 4H | Chronic Phase | Moderate | 54.35±11.64 | 13M | Y | CST | First stroke (CT/MRI); Brunnstrom LL >III; sit balance, walk with aid; excl. MMSE <20, neuro/myopathy, recent LL fracture. | Pelvic stability group practiced exercises including bridge variations, pelvic rotation, and step training using physio balls and closed-chain movements to activate key stabilizing muscles. | 60 min/session, 3×/week | 6weeks | Pelvic stability training group: Pelvic stabilization (e.g., bridging, ball exercises): 60 min × 3/week × 3.5 METs = 630 METs-min/week Conventional rehab: 60 min × 3/week × 3.0 METs = 540 METs-min/week Total = 1170 METs-min/week | 10MWT FMA-LE |
|  |  | Middle income | 13 | Trunk and lower limb function | 13I + 4H | Chronic Phase | Moderate | 58.24±11.77 | 12M/1F | N | RC |  | Control group performed standard lower-limb rehabilitation including ROM, resistance, and balance training. | 60 min/session, 3×/week | 6weeks | Control group: Conventional rehab only: 60 min × 3/week × 3.0 METs = 540 METs-min/week |  |
| 261 | Shirley Handelzalts，2019^325^ | High income | 16 | Lower limb | 11I + 5H | Subacute Phase | Moderate | 62.5±8.4 | M12/F4 | Y | BT | First stroke (CT/MRI); ambulatory (assist/supervise); no weight >135 kg, neuro disease, joint replacement, blindness. | Intervention group received perturbation-based balance training with unexpected platform shifts and treadmill disturbances, combined with cognitive tasks. | 30 min/session, 5×/week | 2.5weeks | Postural perturbation training group: Balance + perturbation training: 30 min × 5/week × 3/week × 3.5 METs = 525 METs-min/week Total = 525 METs-min/week | 6MWT 10MWT BBS |
|  |  | High income | 16 | Lower limb | I | Subacute Phase | Moderate | 60.4±10.1 | M12/F4 | N | MBE |  | Control group performed voluntary weight-shifting and treadmill walking without perturbation, using visual feedback. | 30 min/session, 5×/week | 2.5weeks | Control group: Weight-shifting balance training: 30 min × 5/week × 3/week × 3.0 METs = 450 METs-min/week |  |
| 263 | Kyung Hun Kim，2017^326^ | High income | 15 | Lower limb | 73.3%H + 26.7%I | Chronic Phase | Moderate to severe | 48.27±16.05 | M11/F4 | Y | GT | Stroke (CT/MRI); FAC 4–5; MMSE >24; no contractures, pain, fractures, hemianopia, neuro/cardiac disease; consented. | PBWSTBWT group received backward treadmill walking with progressive reduction in body weight support and increasing speed. Each session included multiple intervals of walking and rest. | 30 min/session, 5×/week | 4weeks | Progressive backward treadmill training: Backward treadmill: 30 min × 5/week × 4.5 METs = 675 METs-min/week Conventional rehab: 30 min × 5/week × 3.0 METs = 450 METs-min/week Total = 1125 METs-min/week | 6MWT |
|  |  | High income | 15 | Lower limb | 60.0%H + 40.0%I | Chronic Phase | Moderate to severe | 50.73±13.50 | M7 /F8 | Y | AE |  | Control group received forward treadmill walking without weight support, with speed adjusted to tolerance. | 30 min/session, 5×/week | 4weeks | Forward treadmill group: Treadmill walking: 30 min × 5/week × 3.5 METs = 525 METs-min/week Conventional rehab: 30 min × 5/week × 3.0 METs = 450 METs-min/week Total = 975 METs-min/week |  |
| 265 | Augustine J. Devasahayam，2024^327^ | High income | 9 | Lower limb | Mixed | Chronic Phase | Mild to moderate | 62.3±10.1 | M11/F1 | N | FT | Stroke >6 mo(CT/MRI); stand 30s; RBT eligible; no comorbidities, aphasia, osteoporosis, heart disease, diabetes; no RBT past year; | AST group engaged in aerobic exercise (e.g., cycling, treadmill) combined with resistance training targeting major muscle groups using individualized intensity guided by HR and RPE. | 60 min/session, 3×/week | 12weeks | Aerobic + resistance training group (AST): Aerobic: 20 min × 3/week × 6.0 METs = 360 METs-min/week Resistance: 30 min × 3/week × 4.0 METs = 360 METs-min/week Warm-up/cool-down: 10 min × 3/week × 2.5 METs = 75 METs-min/week Total = 795 METs-min/week | 6MWT BBS |
|  |  | High income | 7 | Lower limb | Mixed | Chronic Phase | Mild to moderate | 63.4±14.7 | M6/F5 | N | BT |  | RBT group received perturbation training with a high challenge rate to provoke compensatory reactions, progressing in difficulty over time. | 60 min/session, 3×/week | 12weeks | Control group: Combined moderate training: 60 min × 3/week × 3.5 METs = 630 METs-min/week |  |
| 266 | Xiaofeng Chen，2020^328^ | Middle- and high-income | 90 | Trunk | 54I + 36H | Subacute Phase | Moderate | 59.12±12.67 | M57/F33 | Y | CST | First stroke (NCCVD+CT/MRI, <6 mo, stable); MMSE ≥24; stand ≥1 min (eyes open); no major disease, neuro/orthopedic/vision/cognition impairments, abnormal BMI. | Observation group practiced core stability using a multi-axis suspension system in various positions (supine, side-lying, prone), with sling support to maintain pelvic alignment. | 40 min/session, 6×/week | 8weeks | Core stability training group: Core stabilization: 40 min × 6/week × 3.5 METs = 840 METs-min/week Conventional rehab: 40 min × 6/week × 3.0 METs = 720 METs-min/week Total = 1560 METs-min/week | BBS FMA-LE |
|  |  | Middle- and high-income | 90 | Trunk | 54I + 36H | Subacute Phase | Moderate | 59.05±12.74 | M55/F35 | Y | BT |  | Control group performed traditional trunk training (e.g., sit-ups, bridging, resisted trunk motions) without suspension equipment. | 40 min/session, 6×/week | 8weeks | Control group: Trunk control training (e.g., crunches, rotations): 40 min × 6/week × 3.0 METs = 720 METs-min/week Total = 1440 METs-min/week |  |
| 267 | Kaare Severinsen，2014^329^ | High income | 13 | Lower limb | Nt reported | Chronic Phase | Moderate | 50–80 | M13/F5 | N | AE | I stroke (CT, 6–36 mo); age 50–80; LL MRC >3; 10MWT <1.4 m/s; aids allowed; excl. MDI >25, MMSE <20, | AT group performed cycle ergometer aerobic training at 75% HRR. | ~45 min/session, 3×/week | 12weeks | Cycling training (vigorous): 45 min × 3/week × 8.5 METs = 1147.5 METs-min/week | 6MWT 10MWT |
|  |  | High income | 14 | Lower limb | Nt reported | Chronic Phase | Moderate | 57–78 | M11/F6 | N | RT |  | RT group performed resistance training targeting the lower limbs at 70–80% 1RM. | 60 min/session, 3×/week | 12weeks | Moderate-to-high resistance training: 45 min × 3/week × 6.0 METs = 810 METs-min/week |  |
|  |  | High income | 16 | Lower limb | Nt reported | Chronic Phase | Moderate | 52–80 | M12/F6 | N | ULT |  | ST group performed low-intensity upper limb resistance training below 60% 1RM. | 60 min/session, 3×/week | 12weeks | Low-intensity resistance training: 30 min × 3/week × 2.8 METs = 252 METs-min/week |  |
| 268 | Jin Hong Kim，2014^330^ | High income | 10 | Whole-body function | Nt reported | Chronic Phase | Mild to moderate | 54.10±11.69 | Nt reported | Y | RT | First hemiparetic stroke (CT/MRI, <6 mo); follows instructions; 6MWT (± aids); no cardiopulmonary, metabolic meds, prior training, thoracic deformity. | The session included 30 minutes of conventional rehabilitation training, followed by 20 minutes of whole-body automatic movement using the Super Dynamic system and 20 minutes of individualized respiratory training using the RESPIFIT S device. The respiratory session involved interactive game-based feedback with adjustable resistance. | 80 min/session, 3×/week | 4weeks | Combined respiratory, aerobic, and conventional therapy: Conventional PT: 30 min × 3/week × 3.0 METs = 270 METs-min/week Whole-body dynamic exercise: 20 min × 3/week × 4.0 METs = 240 METs-min/week Respiratory training (RESPIFIT): 20 min × 3/week × 2.5 METs = 150 METs-min/week Total = 660 METs-min/week | 6MWT |
|  |  | High income | 10 | Whole-body function | Nt reported | Chronic Phase | Mild to moderate | 53.90±5.82 | Nt reported | Y | RC |  | The session included 30 minutes of conventional rehabilitation and 20 minutes of whole-body automatic movement using the Super Dynamic system, without respiratory training. | 60 min/session, 3×/week | 4weeks | Control group: Conventional PT: 30 min × 3/week × 3.0 METs = 270 METs-min/week Whole-body exercise only: 20 min × 3/week × 4.0 METs = 240 METs-min/week Total = 510 METs-min/week |  |
| 269 | Takumi Igusa，2024^331^ | High income | 15 | Whole-body function | Mixed | Subacute and chronic | Moderate to severe | 83.9±6.5 | M46.7%/F53.3% | Y | GT | CRW eligible (stroke <2 mo, fracture, disuse); 10MWT; MMSE >19.4; CT/MRI; age ≥65; follows commands; walk ≥10 m. | In addition to conventional gait training, rhythmic auditory stimulation (RAS) was applied using a metroNme set at 110% of the patient’s comfortable walking cadence to facilitate step timing and sensorimotor synchronization. | 30 min/session, 5×/week | 3weeks | RAS group: Conventional therapy (ROM, spasticity reduction, balance): 30 min × 5/week × 3.0 METs = 450 METs-min/week Rhythmic auditory gait training: 30 min × 5/week × 4.0 METs = 600 METs-min/week Total = 1050 METs-min/week | 10MWT |
|  |  | High income | 15 | Whole-body function | Mixed | Subacute and chronic | Moderate to severe | 81.3±8.4 | M40%/F60% | Y | MBE |  | Conventional gait training without rhythmic cues, delivered individually on level ground under therapist supervision. | 30 min/session, 5×/week | 3weeks | Control group (traditional gait): Conventional therapy: 30 min × 5/week × 3.0 METs = 450 Gait training: 30 min × 5/week × 3.5 METs = 525 Total = 975 METs-min/week |  |
| 270 | Soo Yong Lee，2024^332^ | High income | 49 | Trunk and lower limb function | 30I + 19H | Chronic Phase | Severe | 65.53±12.17 | M31/F18 | Y | RAT | First stroke (CT/MRI); no neuro/ortho comorbidity; pre-stroke walk independent; no cardio, cancer, aphasia, dementia (MMSE <15), cerebellar; intact cognition; 10MWT. | Robot-assisted walking training (RAWT) using Lokomat with partial body weight support and virtual reality feedback. Each session was preceded and followed by 30 minutes of conventional therapy. | 40 min/session (+30 min CP), 5×/week | 4weeks | RAWT + Conventional therapy: Conventional rehab (CP): 30 min × 5/week × 3.0 METs = 450 RAWT: 40 min × 5/week × 3.5 METs = 700 Total = 1150 METs-min/week | BBS FMA |
|  |  | High income | 47 | Trunk and lower limb function | 29I + 18H | Chronic Phase | Severe | 71.08±12.07 | M28/F19 | N | NPF |  | Conventional physiotherapy based on neurodevelopmental principles including postural alignment, weight shifting, and assisted walking. | 30 min/session, 5×/week | 4weeks | Control group: Conventional rehab only: 30 min × 5/week × 3.0 METs = 450 METs-min/week |  |
| 271 | Erdi Kayabınar，2019^333^ | Middle- and high-income | 32 | Trunk and lower limb function | Mixed | Chronic Phase | Moderate | 55.47±13.58 | M20/F12 | Y | RAT | First stroke (neurologist, >6 mo); ambulates (± aid); no bilateral lesions, neglect, poor cooperation, recent botulinum, acute disease; consented; completes assessments. | Received NDT-Bobath therapy and additional robot-assisted walking using an exoskeleton and body-weight support. The robotic training emphasized gait symmetry, step timing, weight transfer, and joint control, guided one-on-one by a therapist. | 40 min/session (30–45 min RAWT+NDT), 6×/week (RAWT 3×/week) | 5weeks | RAWT + NDT-Bobath: NDT-Bobath: 40 min × 6/week × 3.0 METs = 720 RAWT: 40 min × 3/week × 3.5 METs = 420 Total = 1140 METs-min/week | BBS |
|  |  | Middle- and high-income | 32 | Trunk and lower limb function | Mixed | Chronic Phase | Moderate | 63.81±10.04 | M21/F11 | Y | NPF |  | Received the same amount of NDT-Bobath therapy with conventional gait training, focusing on balance, spatial awareness, and gait phase coordination without robotic assistance. | 40 min/session (30–45 min Walking+NDT), 6×/week (Walking 3×/week) | 5weeks | Control group: NDT-Bobath: 40 min × 6/week × 3.0 METs = 720 Conventional gait training: 40 min × 3/week × 3.0 METs = 360 Total = 1080 METs-min/week |  |
| 272 | Birgül Elmas Bodur，2024^334^ | Middle- and high-income | 16 | Lower limb | I | Chronic Phase | Moderate | 56.75±7.19 | 8M/8F | Y | GT | Stroke ≥3 mo (CT/MRI); age 18–65; ambulation independent; no HF, arrhythmia, cognitive/visual/cerebellar/neglect/post-op LL, neuro disease. | Training included conventional physiotherapy and 30 minutes of overground walking using the ExoAthelt exoskeleton without weight support. The session consisted of warm-up, middle phase with proper gait pattern practice, and cool-down. | 60 min/session, 3×/week | 8weeks | Common rehab (both groups): Conventional rehab: 30 min × 3/week × 3.0 METs = 270 METs-min/week ExoAtlet group (wearable exoskeleton): Exo-assisted walking: 30 min × 3/week × 4.5 METs = 405 Total = 270 + 405 = 675 METs-min/week | 6MWT |
|  |  | Middle- and high-income | 16 | Lower limb | 15I + 1H | Chronic Phase | Moderate | 57.81±8.10 | 9M/7F | Y | RAT |  | Conventional physiotherapy followed by 30 minutes of walking with Lokomat Free-D with 50% body weight support and visual feedback games to encourage gait participation. | 60 min/session, 3×/week | 8weeks | Lokomat group: Lokomat robotic walking: 30 min × 3/week × 3.5 METs = 315 Total = 270 + 315 = 585 METs-min/week |  |
| 274 | Seung-Jun Hyun，2021^335^ | High income | 15 | Trunk and lower limb function | 11I + 4H | Subacute Phase | Moderate | 61.47±11.08 | M6/F9 | Y | VRG | Stroke (CT/MRI); MMSE-K ≥21; stand ≥1 min; sit-to-stand w/o hand aid; excl. joint limits, pain, fracture, vision/vestibular issues. | Sit-to-stand training using a balance board and visual feedback to correct trunk posture, in addition to conventional physiotherapy and electrical stimulation. | 20 min/session, 5×/week | 6weeks | RVF-STS group: Conventional therapy: 30 min × 5/week × 3.0 METs = 450 Visual feedback sit-to-stand: 20 min × 5/week × 3.5 METs = 350 Total = 800 METs-min/week | 10MWT BBS |
|  |  | High income | 15 | Trunk and lower limb function | 10I + 5H | Subacute Phase | Moderate | 59.27±17.00 | M7/F8 | Y | BT |  | Received the same amount of sit-to-stand training and physiotherapy as the RVF-STS group, but without visual feedback, monitored manually by a therapist. | 20 min/session, 5×/week | 6weeks | C-STS group: Conventional therapy: 30 min × 5/week × 3.0 METs = 450 Conventional STS: 20 min × 5/week × 3.0 METs = 300 Total = 750 METs-min/week |  |
| 275 | Young-Jun Kim，2024^336^ | High income | 14 | Upper limb | 9I + 5H | Subacute Phase | Mild to moderate | 59.00±17.83 | M8/F6 | Y | CST | Stroke (CT/MRI); shoulder sublux >1-finger gap; MMSE-K ≥21; shoulder flex/abd strength ≥P+ ≤F−; sit ≥20 min independent; excl. primary shoulder disease, pain, trauma. | Active shoulder training using the PRO SLING system in supine or sitting position. Exercises included shoulder flexion/extension, abduction/adduction, and rotation, with gravity compensation and adjustable resistance. | 30 min/session, 5×/week | 4weeks | VR + outpatient OT group: VR training: 30 min × 5/week × 3.0 METs = 450 Clinic OT: 20 min × 1/week × 3.0 METs = 60 Total = 510 METs-min/week | FMA-UE |
|  |  | High income | 14 | Upper limb | 8I + 6H | Subacute Phase | Mild to moderate | 64.64±14.41 | M5/F9 | N | RC |  | Passive or active upper limb training using the MotorCross MC-3 device, focusing on forward circular motion with standardized motor resistance to assist range of motion. | 30 min/session, 5×/week | 4weeks | Control group (home rehab + clinic OT): Home exercise: 30 min × 5/week × 2.5 METs = 375 Clinic OT: 20 min × 1/week × 3.0 METs = 60 Total = 435 METs-min/week |  |
| 277 | KyoChul Seo，2015^337^ | High income | 10 | Lower limb | H | Chronic Phase | Moderate | 59.4±10.8 | M6/F4 | Y | GT | H stroke ≥6 mo (MRI/CT); stand ≥30s, walk ≥30m indoors; follows commands; no antispastic meds/aids; excl. cognitive/respiratory issues. | 30-minute stair climbing with PNF facilitation after conventional training. Patients barefoot on wooden stairs (width 0.8 m, step height 10 cm) performed guided knee flexion, hip IR, and dorsiflexion with manual resistance and verbal cues. | 60 min/session, 3×/week | 4weeks | PNF stair walking group: Conventional therapy: 30 min × 3/week × 3.0 METs = 270 PNF stair: 30 min × 3/week × 4.0 METs = 360 Total = 630 METs-min/week | BBS |
|  |  | High income | 10 | Lower limb | H | Chronic Phase | Moderate | 59.5±7.4 | M5/F5 | Y | NPF |  | Same conventional therapy, followed by overground walking with the same PNF cues but on flat terrain. | 60 min/session, 3×/week | 4weeks | PNF ground walking group: Conventional therapy: 30 min × 3/week × 3.0 METs = 270 PNF ground walking: 30 min × 3/week × 3.0 METs = 270 Total = 540 METs-min/week |  |
| 278 | Sung-jin Kim，2015^338^ | High income | 16 | Lower limb | Mixed | Chronic Phase | Moderate | 65.2±6.4 | M12/F4 | Y | AE | Stroke ≥6 mo (CT/MRI); MMSE >21; walk ≥10 m independent (aids allowed); no vision/MSK walking impairment. | In addition to routine rehabilitation, patients performed 30 minutes of fixed recumbent cycling daily, 5 times perweek for 6weeks. The Super Dynamic 3000 (lower limb only) was used. Patients cycled at 25–30 W resistance (level 4), 50–60 rpm, without interruptions. | 30 min/session, 5×/week | 6weeks | Stationary cycling + conventional rehab: Conventional rehab: 30 min × 5/week × 3.0 METs = 450 Cycling: 30 min × 5/week × 4.0 METs = 600 Total = 1050 METs-min/week | 10MWT BBS |
|  |  | High income | 16 | Lower limb | Mixed | Chronic Phase | Moderate | 61.7±6.1 | M13/F3 | N | RC |  | Received only 30 minutes of routine rehabilitation (e.g., strength and gait training), same frequency as the intervention group, without cycling. | 30 min/session, 5×/week | 6weeks | Control group: Conventional rehab only: 30 min × 5/week × 3.0 METs = 450 METs-min/week |  |
| 279 | Munsang Choi，2015^339^ | High income | 13 | Lower limb | 62%I + 38%H | Chronic Phase | Mild | 71.9±6.92 | M3/F10 | N | MBE | Unilateral stroke ≥6 mo (CT/MRI); independent 10MWT + stairs; MMSE ≥24; excl. visual, cardiac, ortho, pain, aphasia, prior similar training. | 30-minute stepping training under p-bar support, with mirrors placed in front and behind to provide visual feedback for trunk and foot position. | 30 min/session, 3×/week | 6weeks | Stepper training (with or without visual feedback): 30 min × 3/week × 4.5 METs = 405 METs-min/week | 10MWT |
|  |  | High income | 13 | Lower limb | 77%I + 23%H | Chronic Phase | Mild | 69.8±9.76 | M4/F9 | N | GT |  | Same stepping device and time but without visual feedback (N mirrors). Training environment and support structure were consistent. | 30 min/session, 3×/week | 6weeks | Control group: Same as intervention: Stepper 30 min × 3/week × 4.5 METs = 405 METs-min/week |  |
| 280 | Guanli Xie，2018^340^ | Middle- and high-income | 120 | Trunk and lower limb function | Mainly I | Chronic Phase | Moderate | 60.9±8.7 | M83/F37 | Y | MBE | Stroke >3 mo (NCCVD/CT/MRI); balance disorder; walk ≥6 m (± aid); MMSE >24; understands; no vestibular, severe AV damage, aphasia, heart disease, GI bleed. | Performed 60-minute sessions (15 min warm-up, 45 min core movement) of Tai Chi “Cloud Hand” training derived from the 24-form standard. | 60 min/session, 5×/week | 12weeks | Tai Chi cloud hand group: Conventional rehab: 30 min × 5/week × 3.0 METs = 450 Tai Chi cloud hand: 45 min × 5/week × 3.5 METs = 787.5 Total = 1237.5 METs-min/week | BBS FMA |
|  |  | Middle- and high-income | 124 | Trunk and lower limb function | Mainly I | Chronic Phase | Moderate | 60.1±8.6 | M99/F25 | Y | BT |  | Received 60-minute balance training per Chinese Rehabilitation Guidelines, including static/dynamic balance, Bobath, and gait training. Frequency and duration matched the experimental group. | 60 min/session, 5×/week | 12weeks | Balance rehab group: Conventional rehab: 30 min × 5/week × 3.0 METs = 450 Balance + Bobath + gait: 30 min × 5/week × 3.0 METs = 450 Total = 900 METs-min/week |  |
| 281 | Jong-Hoon Moon，2018^341^ | High income | 9 | Upper limb | I | Acute Phase | Moderate | 70.81±8.75 | M5/F4 | N | TOT | Cerebral infarct <30d (CT/MRI); Brunnstrom UL ≤3; MMSE-K ≥24; no contracture/pain/fracture; excl. neglect, apraxia, dementia, major depression. | Completed 30 minutes of task-oriented circuit training using 6 tools (e.g., clay, inclined board, ROM arc). Each task lasted 5 minutes. Four difficulty levels were provided and adjusted by the therapist. | 30 min/session, 5–6×/week | 4weeks | Task-oriented OT + aerobic group: Estimated MET = 3.3 30 min × 5.5/week × 3.3 METs = 544.5 METs-min/week | FMA-UE |
|  |  | High income | 9 | Upper limb | I | Acute Phase | Moderate | 63.36±11.67 | M5/F4 | N | NPF |  | Received 30 minutes of neurodevelopmental therapy daily (10 min warm-up + 20 min NDT upper limb exercises). Manual guidance only, without tools. | 30 min/session, 5–6×/week | 4weeks | Low-intensity neuromotor + upper limb training: Estimated MET = 2.8 30 min × 5.5/week × 2.8 METs = 462 METs-min/week |  |
| 282 | Tahreem Zaman，2022^342^ | Low- and middle-income | 9 | Upper limb | 7I + 2H | Acute and subacute | Mild to moderate | 60.78±9.08 | M3/F6 | N | TOT | First unilateral stroke (neurologist/CT/MRI); Brunnstrom ≥4; MAS ≤2; MMSE ≥24; follows; no recurrent stroke, TIA, neglect, poor sitting balance, severe comorbidities. | Performed 6 seated tasks (e.g., polishing table, forward/side reach, cup-to-glass transfer, pen holding, lifting basket) | 45 min/session, 5×/week | 6weeks | Group A (Task-Oriented Training): Upper limb task practice (e.g., wiping, lifting, grasping, pouring, translation) MET = 3.5 → 45 min × 5/week × 3.5 = 787.5 METs-min/week | FMA-UE |
|  |  | Low- and middle-income | 9 | Upper limb | I | Acute and subacute | Mild to moderate | 61.33±6.78 | M2/F7 | N | RC |  | Received 10–15 min hot pack, TENS stimulation, and static/isometric strength exercises for shoulder/elbow/wrist (10 reps × 1 set), 45 min total | 45 min/session, 5×/week | 6weeks | Group B (Conventional Therapy): Stretching + isometric resistance + TENS + heat therapy MET = 2.8 → 45 min × 5/week × 2.8 = 630 METs-min/week |  |
| 285 | Kyung-Hun Kim，2021^343^ | High income | 13 | Trunk and lower limb function | 6I + 7H | Chronic Phase | Moderate | 50.23±14.89 | M7/F6 | Y | MBE | CT/MRI-confirmed stroke; Brunnstrom stage III–IV; K-MMSE ≥24; able to walk 10m independently; excluded ankle contracture, sensory loss, vestibular/cerebellar disorder, severe cognitive deficits, neglect, or aphasia. | 30 minutes of cognitive-sensory training (e.g., proprioception, tactile, heel pressure, spatial tasks), followed by 30 minutes of task-specific training (sit-to-stand, lower limb + gait, BWST treadmill), each task 10 min. | 60 min/session, 5×/week | 8weeks | Experimental Group I (TST + CSE): Conventional rehab: 30 min × 5 × 3.0 = 450 CSE (sensory, spatial, cognitive tasks): 30 min × 5 × 2.8 = 420 TST (sit-to-stand, gait, posture): 30 min × 5 × 4.0 = 600 Total = 1470 METs-min/week | 10MWT |
|  |  | High income | 12 | Trunk and lower limb function | 6I + 6H | Chronic Phase | Moderate | 52.75±17.00 | M7/F5 | Y | TOT |  | Same 30-minute TST as above plus 30 minutes of conventional PT (ROM, stretching, strength, balance, gait). | 60 min/session, 5×/week | 8weeks | Experimental Group II (TST only): Conventional: 450 TST: 600 Total = 1050 METs-min/week |  |
|  |  | High income | 12 | Trunk and lower limb function | 8I + 4H | Chronic Phase | Moderate | 55.08±10.55 | M8/F4 | N | RC |  | Received 2 sessions/day of 30-minute conventional PT (ROM, strength, gait, balance, dynamic training). | 30 min/session ×2/day, 5×/week | 8weeks | Control Group: PT: 30 min × 2/day × 5 days × 3.0 = 900 METs-min/week |  |
| 286 | Sıdıka Büyükvural Şen，2015^344^ | Middle- and high-income | 25 | Lower limb | 84%I + 16%H | Subacute and chronic | Moderate | 51.3±12.0 | M17/F8 | Y | RT | First-ever stroke confirmed by CT/MRI; 2–9 months post-onset; Brunnstrom LE stage 4–6; MMT ≥24; excluded aphasia, severe cardiorespiratory/musculoskeletal issues, prior stroke, or Nncompliance. | In addition to 30-minute routine rehab, patients underwent daily maximal isokinetic strength training for bilateral knee and ankle flexors/extensors using Biodex System 3 Pro. Training included multiple sets at 60°/s to 180°/s | ~45 min/session, 5×/week | 3weeks | Experimental Group: Conventional rehab: 30 min × 5 × 3.0 = 450 Bilateral isokinetic resistance (knee/ankle): 30 min × 5 × 4.5 = 675 Total = 1125 METs-min/week | 6MWT 10MWT BBS |
|  |  | Middle- and high-income | 25 | Lower limb | 92%I + 8%H | Subacute and chronic | Moderate | 55.4±10.5 | M16/F9 | Y | RC |  | Received only 30 minutes/day of routine therapy, including gait, coordination, and lower limb strengthening. N resistance training. | 30 min/session, 5×/week | 3weeks | Control Group: Only conventional rehab: 450 METs-min/week |  |
| 287 | HoYoung Kim，2015^345^ | High income | 11 | Trunk and lower limb function | Nt reported | Chronic Phase | Mild to moderate | 53.45±11.54 | M7/F4 | Y | MBE | CT/MRI-confirmed stroke; K-MMSE ≥24; Brunnstrom ≥4; MAS <3 (plantar flexors); can walk 10m independently; N vision or gait-affecting orthopedic/neurological disorders. | Patients received Tai Chi training based on a rehabilitation-oriented protocol with 10 basic movements. Sessions included diaphragmatic breathing, full-body warm-up and cooldown, with posture control and weight-shifting emphasized. | 60 min/session, 2×/week | 6weeks | Experimental Group (Tai Chi + PT): PT: 30 min × 2/day × 5 days × 3.0 = 900 Tai Chi: 60 min × 2/week × 4.0 = 480 Total = 1380 METs-min/week | 10MWT |
|  |  | High income | 11 | Trunk and lower limb function | Nt reported | Chronic Phase | Mild to moderate | 55.18±10.20 | M6/F5 | Y | NPF |  | Patients received standard neurodevelopmental therapy, including NDT, Bobath technique, and proprioceptive neuromuscular facilitation. | 30 min/session ×2/day, 5×/week | 6weeks | Control Group: PT only: 900 METs-min/week |  |
| 288 | Jong Yoon Chang，2024^346^ | High income | 15 | Upper limb | 8I + 7H | Chronic Phase | Moderate | 65.2±5.4 | M7/F8 | N | RAT | Chronic stroke >2 yr (CT/MRI); UE paralysis (elbow/wrist); MAS ≤3; no aphasia, severe cognitive/neuromuscular comorbidities; excl. recent device study (<30 d). | Elbow and wrist joint training using Rebless® in active-assistive mode. Each joint received 7.5 minutes of flexion/extension exercise, with resistance or assistance gradually adjusted. | 30 min/session, 2–3×/week | 4weeks | Rebless Group: Robotic-assisted upper limb (elbow/wrist) ROM and resistance MET = 3.5 × 30 min × 2.5 sessions/week = 262.5 METs-min/week | FMA-UE |
|  |  | High income | 15 | Upper limb | 8I + 7H | Chronic Phase | Moderate | 67.2±5.4 | M7/F8 | N | ULT |  | Active upper limb cycling using Motomed®, involving only elbow and shoulder movements, without wrist involvement. | 30 min/session, 2–3×/week | 4weeks | Ergometer-based upper limb training MET = 3.0 × 30 min × 2.5 sessions/week =225 METs-min/week |  |
| 289 | Muhammad Hamad Haleem，2025^347^ | Low- and middle-income | 22 | Trunk and lower limb function | I | Subacute Phase | Moderate | 57.18±4.66 | M22 | Y | ESX | First MCA territory stroke confirmed by CT/MRI; BBS 21–40; age 45–70; N cognitive, visual/hearing deficits, or neuromuscular disorders affecting lower limbs. | ANdal tDCS was applied to the M1 area (C3/C4) using a 2 mA current, followed by motor relearning practice. Electrodes covered cortical regions for upper/lower limbs and trunk. | 70 min/session, 5×/week | 4weeks | Experimental Group (Motor Relearning + tDCS): Rehab: 30 min × 5 × 3.5 = 525 tDCS (40 min total/week × 1.5 METs) = 300 Total = 825 METs-min/week | BBS |
|  |  | Low- and middle-income | 22 | Trunk and lower limb function | I | Subacute Phase | Moderate | 56.41±4.04 | M22 | Y | MBE |  | Same motor training was conducted, but tDCS was applied in sham mode with N actual stimulation. | 70 min/session, 5×/week | 4weeks | Control Group: Rehab only = 525 METs-min/week |  |
| 291 | Hyung-Kyu Kang，2011^348^ | High income | 10 | Lower limb | 6I + 4H | Chronic Phase | Moderate to severe | 55.9±6.5 | M6/F4 | Y | MBE | Chronic stroke >6 months; able to walk >15 min; MMSE ≥21; Brunnstrom >4; N visual impairment or other neurological/cardiac disease. | Treadmill training was paired with head-mounted visual stimulation simulating street walking at varying speeds (0.25x to 2x). | 30 min/session, 3×/week | 4weeks | TOF Group (Treadmill + Optic Flow): Rehab: 30 min × 5 × 3.0 = 450 Treadmill + VR optic flow: 30 min × 3 × 3.5 = 315 Total = 765 METs-min/week | 6MWT 10MWT |
|  |  | High income | 10 | Lower limb | 3I + 7H | Chronic Phase | Moderate to severe | 56.3±7.6 | M4/F6 | Y | AE |  | Identical treadmill training without optic flow feedback. | 30 min/session, 3×/week | 4weeks | Treadmill Group: Rehab: 450 Treadmill: 30 min × 3 × 3.0 = 270 Total = 720 METs-min/week |  |
|  |  | High income | 10 | Lower limb | 6I + 4H | Chronic Phase | Moderate to severe | 56.1±7.8 | M6/F4 | Y | BT |  | Conventional rehabilitation involving stretching, joint mobility, and neuro-motor learning tasks. | 30 min/session, 3×/week | 4weeks | Control Group: Rehab: 450 ROM + sit-to-stand: 30 min × 3 × 2.5 = 225 Total = 675 METs-min/week |  |
| 292 | Jin Shin，2022^349^ | High income | 16 | Trunk and lower limb function | 50%H + 50%I | Chronic Phase | Moderate | 55.44±10.22 | M13/F3 | Y | MBE | CT/MRI-confirmed stroke; Brunnstrom ≥4 (paretic LE); MMSE ≥24; able to walk >10m; excluded vision/hearing loss, heart disease, severe hypertension, or orthopedic disorders. | Treadmill training with visual feedback on step length using a 40" display and rhythmic auditory cues. Three-color visual feedback guided stride length adjustments. | 40 min/session, 3×/week | 8weeks | Experimental Group (Visual + Auditory Treadmill): Rehab: 30 min × 5 × 3.0 = 450 Treadmill with feedback: 30 min × 3 × 4.5 = 405 Total = 855 METs-min/week | BBS |
|  |  | High income | 16 | Trunk and lower limb function | 37.5%H + 62.5%I | Chronic Phase | Moderate | 56.69±9.89 | M12/F4 | Y | AE |  | Treadmill walking at gradually increased speed, without visual or auditory feedback. | 40 min/session, 3×/week | 8weeks | Control Group (N feedback treadmill): Rehab: 450 Treadmill: 30 min × 3 × 3.5 = 315 Total = 765 METs-min/week |  |
| 293 | Yeon-Gyu Jeong，2016^350^ | High income | 15 | Lower limb | 8H + 7I | Chronic Phase | Moderate | 73.67±3.78 | M10/F5 | Y | GT | First stroke 6–12 months ago confirmed by CT/MRI; FAC 4–5; MMSE ≥23; able to walk 10m with or without cane/AFO; N neuromuscular/systemic comorbidities affecting gait. | Treadmill training with transparent obstacle crossing (1cm, 4cm, 8cm heights) and concurrent strength/stretching exercises before walking. | 60 min/session (30 min gait + 30 min functional), 5×/week | 4weeks | TWT–OC (Treadmill + Obstacle Course): Rehab: 450 Obstacle gait training: 30 min × 5 × 4.5 = 675 Total = 1125 METs-min/week | 6MWT 10MWT BBS |
|  |  | High income | 14 | Lower limb | 8H + 6I | Chronic Phase | Moderate | 71.43±4.12 | M6/F8 | Y | AE |  | Treadmill training without obstacles; speed was increased progressively based on tolerance. | 60 min/session, 5×/week | 4weeks | TWT Only (Treadmill Only): Rehab: 450 Treadmill: 30 min × 5 × 4.0 = 600 Total = 1050 METs-min/week |  |
| 295 | Jun-Ho Shin，2015^351^ | High income | 10 | Lower limb | 9I + 1H | Chronic Phase | Moderate | 51.5±11.9 | M7/F3 | N | CIMT | CT/MRI-confirmed stroke; able to walk independently (≥1.0 km/h); capable of following commands. | Symmetric arm swing using Nrdic poles during treadmill walking, with therapist assistance or band support for the affected limb. | 30 min/session, 3×/week | 4weeks | Experimental (Arm-Swing Treadmill): 30 min × 3/week × 4.0 = 360 METs-min/week | 6MWT |
|  |  | High income | 10 | Lower limb | 5I + 5H | Chronic Phase | Moderate | 55.2±9.5 | M8/F2 | N | GT |  | Same treadmill protocol but with both arms fixed on handles. | 30 min/session, 3×/week | 4weeks | Control (Arm-Fixed Treadmill): 30 min × 3/week × 3.0 = 270 METs-min/week |  |
| 296 | Ecem Pelin Kaymaz，2024^352^ | Middle- and high-income | 10 | Trunk | I | Chronic Phase | Moderate | 61.9±7.4 | M6/F4 | Y | CST | First stroke (neurologist/CT/MRI); MMSE ≥25; MAS 0–3 (UE/LE); Brunnstrom ≥3; BMI <30; excl. back pain, abdominal surgery, neuro disease. | Trunk stabilization exercises including supine flexion, seated extension, and pelvic tilts on a Bobath ball. | 30 min/session, 5×/week | 4weeks | Experimental Group (TSE + Rehab): Rehab: 30 min × 5 × 3.0 = 450 Trunk stabilization exercise (e.g., ball, bridging): 30 min × 5 × 3.5 = 525 Total = 975 METs-min/week | BBS |
|  |  | Middle- and high-income | 10 | Trunk | I | Chronic Phase | Moderate | 62.7±8.2 | M6/F4 | N | RC |  | Conventional training focused on joint mobility, postural control, gait, strength, and ADL tasks. | ~30 min/session, 5×/week | 4weeks | Control Group: Rehab only: 450 METs-min/week |  |
| 297 | Kyoung-Il Kim，2023^353^ | High income | 15 | Trunk | I | Chronic Phase | Moderate | 62.53±10.54 | M11/F4 | Y | MBE | First stroke confirmed by neurologist and CT/MRI; K-MMSE >24; N visual or medical comorbidities affecting exercise. | Trunk stabilization exercises using laser pointer feedback for neck, trunk, and hip control. Tasks required maintaining alignment toward a visual target. | 30 min/session, 3×/week | 6weeks | Experimental Group: Conventional rehab: 30 min × 5 × 3.0 = 450 Trunk stability training with laser feedback (supine, bridge, oblique): 30 min × 3 × 3.5 = 315 Total = 765 METs-min/week | 10MWT BBS |
|  |  | High income | 15 | Trunk | I | Chronic Phase | Moderate | 60.67±10.04 | M9/F6 | Y | CST |  | Identical exercise sequence without visual feedback, guided only by verbal cues. | 30 min/session, 3×/week | 6weeks | Control Group: Same protocol but without laser feedback Total = 765 METs-min/week |  |
| 298 | Yan-fang Sui, 2023^354^ | Middle- and high-income | 50 | Trunk | I | Subacute Phase | Moderate | 58.90±4.78 | M32/F18 | Y | NPF | Stroke diagNsed per 1995 Chinese national criteria and confirmed by CT/MRI. | Trunk motor imagery training involving video observation of trunk tasks followed by mental rehearsal with therapist prompts. | 300 min/day, 5×/week | 4weeks | Experimental Group: Conventional neuro rehab (PNF, OT, etc.): 300 min × 5 × 3.0 = 4500 Trunk imagery training: 30 min × 5 × 1.5 = 225 Total = 4725 METs-min/week | BBS FMA |
|  |  | Middle- and high-income | 50 | Trunk | I | Subacute Phase | Moderate | 59.50±4.80 | M31/F19 | N | MBE |  | Conventional rehab including PNF, Rood, motor relearning, occupational therapy, and ADL training. | Additional 30 min imagery, 5×/week | 4weeks | Control Group: Conventional rehab only:4500 METs-min/week |  |
| 299 | Klas Sandberg 2016^355^ | High income | 29 | Trunk and lower limb function | I | Subacute Phase | Mild | 71.3±7.0 | M：14（48%）；F：15（52%） | N | AE | Stroke diagNsed within 3 days per clinical protocol (likely CT/MRI-based) from hospital stroke unit. | Group-based high-intensity aerobic training combining cycling intervals, flexibility, and resistance work, aiming for Borg RPE 14–15. | 60 min/session, 2×/week | 12weeks | High-intensity Aerobic Group: Warm-up (15 min) + flexibility (10 min) + cooldown (15 min): 44 min/session × 2/week × 4.0 METs = 352 Cycling (8 min × 2 rounds) at RPE 14–15 = 16 min × 2/week × 7.0 METs = 224 Total = 576 METs-min/week | 6MWT |
|  |  | High income | 27 | Trunk and lower limb function | 96.3% I + 1H | Subacute Phase | Mild | 70.4±8.1 | M14/F13 | N | NE |  | N structured rehab, only general health advice to maintain daily activity. | N scheduled training | 12weeks | 0 |  |
| 302 | Wei Lu 2024^356^ | Middle- and high-income | 31 | Trunk and lower limb function | Mixed | Acute Phase | Moderate | 62.48±6.53 | M23/F8 | Y | MBE | CT/MRI-confirmed stroke with basic participation ability confirmed by senior physical therapist. | Balance training using Pro-Kin visual feedback with body-weight support. Included anterior-posterior, lateral, and circular sway, and visual games. | 20 min/session ×2/day, 5×/week | 4weeks | Experimental Group: Conventional training: 20 min × 10 × 3.0 = 600 Pro-Kin visual feedback: 10 min × 10 × 4.0 = 400 Total = 1000 METs-min/week | BBS |
|  |  | Middle- and high-income | 31 | Trunk and lower limb function | Mixed | Acute Phase | Moderate | 64.77±10.24 | M19/F12 | N | BT |  | Traditional balance tasks such as strength training, resisted reaches, ball tosses, and push-pull actions. | 20 min/session ×2/day, 5×/week | 4weeks | Control Group: Conventional training (sit-stand, ball push, balance): 20 min × 10 × 3.0 = 600 METs-min/week |  |
| 303 | Nh-Wook Pak 2020^357^ | High income | 11 | Trunk and lower limb function | 55%H + 45%I | Chronic Phase | Moderate | 58.8±9.9 | M7/F4 | Y | MBE | Clinically diagNsed hemiparetic stroke; K-MMSE ≥24; imaging Nt specified; enrolled via rehab hospital screening. | Progressive squatting with laser feedback for controlled weight-shifting, using mirror-based targets to guide performance. | 20 min/session, 5×/week | 4weeks | Experimental Group: Conventional: 30 min × 5 × 3.0 = 450 Visual load squats: 20 reps × 3 sets × 5 = ~60 min/week × 4.0 = 240 Total = 690 METs-min/week | 10MWT |
|  |  | High income | 10 | Trunk and lower limb function | 50%H + 50%I | Chronic Phase | Moderate | 64.5±7.8 | M6/F4 | Y | RT |  | Same squatting setup without laser or visual targets. | 20 min/session, 5×/week | 4weeks | Control Group: Visual feedback squats (N target): 60 min × 3.5 = 210 Total = 660 METs-min/week |  |
| 304 | Taewoong Jeong 2024^358^ | High income | 13 | Trunk and lower limb function | 6H + 7I | Chronic Phase | Moderate | 61.92±8.93 | M7/F6 | Y | BT | Hemiparetic stroke confirmed by rehab physician; K-MMSE ≥24; able to understand procedures; diagNsis from affiliated rehab hospitals, N imaging specified. | Balance training under visual deprivation. Patients wore eye covers after a short familiarization period and performed various balance tasks in parallel bars. | 30 min/session, 5×/week | 3weeks | Group A (Visual Deprivation): Conventional: 30 min × 5 × 3.0 = 450 EY-closed balance training: 30 min × 5 × 3.5 = 525 Total = 975 METs-min/week | BBS |
|  |  | High income | 13 | Trunk and lower limb function | 5H + 8I | Chronic Phase | Moderate | 61.85±9.75 | M6/F7 | Y | MBE |  | Same balance tasks but performed with mirror-based visual feedback. | 30 min/session, 5×/week | 3weeks | Group B (Mirror Feedback): Same conventional + mirror-based movement training: 525 Total = 975 METs-min/week |  |
|  |  | High income | 13 | Trunk and lower limb function | 9H + 4I | Chronic Phase | Moderate | 58.15±7.13 | M7/F6 | N | RC |  | Standard rehab with Bobath, PNF, strength and posture training. | 30 min/session, 5×/week | 3weeks | Control Group: Conventional only = 450 METs-min/week |  |
| 305 | Nuran Eyvaz 2018^359^ | Middle- and high-income | 30 | Trunk and lower limb function | 27I + 3H | Chronic Phase | Moderate | 58.5±6.27 | M18/F12 | N | WA | Hemiparetic stroke diagNsed by neurologist; Mini-Mental Test >24; N imaging data reported; diagNsis stable and clinically confirmed. | Aquatic training including dynamic gait, squats, coordination drills, and underwater walking. Also performed land-based exercises. | 40–60 min/session, 3×/week (aquatic) + 2×/week (land) | 6weeks | Experimental (Aquatic + Land): Water: 40 min × 3 × 5.0 = 600 Land: 60 min × 2 × 4.0 = 480 Total = 1080 METs-min/week | BBS |
|  |  | Middle- and high-income | 30 | Trunk and lower limb function | 23I + 7H | Chronic Phase | Moderate | 58.3±5.43 | M13/F17 | N | FT |  | Land-based rehab involving joint mobility, core and balance training, stair climbing, and elastic band resistance. | 60 min/session, 5×/week | 6weeks | Control (Land only): 60 min × 5 × 4.0 = 1200 METs-min/week |  |
| 306 | Mehmet Duray 2023^360^ | Middle- and high-income | 13 | Whole-body function | 9I + 4H | Chronic Phase | Mild to moderate | 52.46±9.85 | M9/F4 | Y | NPF | Stroke diagNsed by neurologist and physiatrist; N imaging reported; clinical confirmation only. | Received NDT-based rehab including strength, posture, and functional reach. | 60 min/session, 3×/week | 4weeks | NDT Group: 60 min × 3/week × 3.5 METs = 630 METs-min/week | 6MWT |
|  |  | Middle- and high-income | 15 | Whole-body function | 10I + 5H | Chronic Phase | Mild to moderate | 49.67±7.88 | M11/F4 | Y | VT |  | Additionally underwent whole-body vibration training (starting at 30Hz, 1.5mm amplitude), combining static and semi-flexed postures. | 20 min/session, 2×/week | 4weeks | NDT + WBV Group: WBV: 20 min × 2/week × 4.5 = 180 NDT: 630 Total = 810 METs-min/week |  |
| 307 | Torunn Askim 2018^361^ | High income | 186 | Whole-body function | 92.5%I + 7.5%H | Subacute Phase | Moderate | 71.7±11.9 | M104/F82 | Y | FT | Clinically diagNsed stroke (I/H); MMSE ≥21 (Nn-aphasic) or ≥17 (aphasic). | Received personalized coaching involving physical activity planning and monthly follow-up, emphasizing ≥210 min/week of daily activity and moderate-to-high intensity training. | 210 min/week + 1×/week moderate-high intensity; monthly supervision | 18months | Intervention Group:  Phase 1 (clinic/home):  Clinic: 45 min/week × 3.0 METs = 135 Phase 2 (18-month exercise plan): Moderate walking: 30 min × 7 × 3.5 = 735 Vigorous training: 2 × 45–60 min/week × 6.0 = 540–720 Estimate (midpoint): 735 + 630 = 1365 METs-min/week | 6MWT BBS |
|  |  | High income | 194 | Whole-body function | 89.7%I + 10.3%H | Subacute Phase | Moderate | 72.3±11.3 | M127/F67 | N | RC |  | Standard outpatient care per national guidelines, including once-weekly therapy. | 1×/week rehab (first 3 months), self-management thereafter | 18months | Control Group: Home training only: 45 min/week × 3.0 = 135 METs-min/week |  |
| 308 | Louis N. Awad 2024^362^ | High income | 40 | Lower limb | Mainly I | Subacute and chronic | Moderate | 61.3±6.5 | M33/F19 | Y | MBE | Clinically diagNsed stroke; NIHSS cognition items 1b ≤1, 1c = 0. | Gait training using the InTandem system, which synchronizes music tempo with gait rhythm via shoe sensors and real-time feedback. N therapist was needed. | 30 min/session, 3×/week | 5weeks | InTandem Group: Conventional rehab: 30 min × 3 × 3.0 = 270 Rhythmic audio-guided walking: 30 min × 3 × 3.5 = 315 Total = 585 METs-min/week | 10MWT |
|  |  | High income | 32 | Lower limb | Mainly I | Subacute and chronic | Moderate | 63.6±7.5 | M35/F17 | N | GT |  | Ground walking without rhythm feedback, supervised by researchers. | 30 min/session, 3×/week | 5weeks | Control Group: Conventional only:270 METs-min/week |  |
| 309 | Wan-Yun Huang 2022^363^ | Middle- and high-income | 15 | Lower limb | 53%H + 47%I | Chronic Phase | Moderate | 57.0±9.5 | M12/F3 | Y | MBE | DiagNsed by physiatrist via neurological exam and history; excluded Parkinson’s, SCI, severe orthopedic limitations. | The experimental group received 15-minute gait training using a laser-cane equipped with dual laser beams for step guidance, combined with 15 minutes of conventional therapy focusing on strength and balance. | 30 min/session (15 min walking + 15 min PT), 2×/week | 4weeks | Experimental Group: PT: 15 min × 2/week × 3.0 = 90 Laser-guided 4-point cane training: 15 min × 2/week × 3.5 = 105 Total = 195 METs-min/week | BBS |
|  |  | Middle- and high-income | 15 | Lower limb | 47%H + 53%I | Chronic Phase | Moderate | 66.1±9.0 | M11/F4 | Y | GT |  | The control group received the same conventional therapy and walking training without laser guidance, using a standard quad cane. | 30 min/session (15 min walking + 15 min PT), 2×/week | 4weeks | Control Group: PT: 15 min × 2/week × 3.0 = 90 Conventional cane training: 15 min × 2/week × 3.0 = 90 Total = 180 METs-min/week |  |
| 311 | Pallavi Harjpal 2022^364^ | Middle income | 20 | Trunk and lower limb function | Mixed | Subacute Phase | Moderate | 51.75±7.06 | M11/F9 | Y | MBE | Clinically diagNsed new-onset stroke by rehabilitation physician; excluded prior stroke, TIA, brainstem/MCA stroke, severe illness. | The control group received the same conventional therapy and walking training without laser guidance, using a standard quad cane. | 40 min/session, 5×/week; BTG: 20 min each side | 4weeks | Experimental Group (BTG - Bilateral Training): Conventional rehab (MRP + PNF): 20 min × 5 × 3.0 = 300 DeLorme strength training on Nn-paretic side: 20 min × 5 × 4.5 = 450 Total = 750 METs-min/week | BBS |
|  |  | Middle income | 20 | Trunk and lower limb function | Mixed | Subacute Phase | Moderate | 51.50±8.40 | M12/F8 | Y | LLT |  | The BTG group followed the same protocol and additionally performed strength training for the unaffected limb using the DeLorme principle, targeting hip flexors, abductors, extensors, knee extensors, and ankle dorsiflexors. | 20 min/session, 5×/week | 4weeks | Control Group: MRP + PNF only: 20 min × 5 × 3.0 = ✅ 300 METs-min/week |  |
| 313 | Seung Don Yoo 2010^365^ | High income | 28 | Trunk | 50%H + 50%I | Subacute Phase | Moderate | 59.61±18.16 | M13/F15 | Y | NPF | Clinically confirmed stroke; excluded aphasia, bilateral paralysis, neglect, cognitive/neuro disorders; N imaging reported. | The control group received standard physical therapy, including neurodevelopmental techniques, gait, and occupational therapy. | 60 min/session, 3×/week | 4weeks | Experimental Group: Conventional rehab (neurodevelopment, walking, OT): 30 min × 3 × 3.0 = 270 Core stability training (bridge, dead bug, bird dog): 30 min × 3 × 3.5 = 315 Total = 585 METs-min/week | BBS |
|  |  | High income | 31 | Trunk | 52%H + 48%I | Subacute Phase | Moderate | 61.77±12.58 | M17/F14 | N | CST |  | The experimental group additionally performed core stabilization exercises (CSP), consisting of nine progressive exercises (e.g., bridges, cross-extensions, bird-dog), with therapist assistance. | 30 min/session, 3×/week | 4weeks | Control Group: Conventional only: 270 METs-min/week |  |
| 314 | Yeon Gyo Nam 2022^366^ | High income | 55 | Lower limb | 61.8%I + 38.2%H | Chronic Phase | Moderate | 62.42±15.04 | M35/F20 | Y | GT | Stroke diagNsed by neurologist; MMSE ≥10, MAS ≤2; excluded severe cognitive, ataxia, LE orthopedic disorders. | The control group underwent therapist-guided overground gait training and conventional therapy. | 30 min/session, 5×/week | 4weeks | Group A (Therapist-assisted walking): Conventional rehab: 30 min × 5 × 3.0 = 450 Assisted walking: 30 min × 5 × 4.0 = 600 Total = 1050 METs-min/week | 6MWT 10MWT BBS |
|  |  | High income | 54 | Lower limb | 61.1%I + 38.9%H | Chronic Phase | Moderate | 60.63±15.61 | M34/F20 | Y | RAT |  | The experimental group used the EXOWALK robotic gait trainer (speed <1.8 km/h) for electromechanical walking assistance, in combination with conventional rehabilitation. | 30 min/session, 5×/week | 4weeks | Group B (EXOWALK robot-assisted): Conventional rehab: 450 EXOWALK: 30 min × 5 × 4.5 = 675 Total = 1125 METs-min/week |  |
| 315 | Mariusz Drużbicki 2018^367^ | High income | 15 | Lower limb | I | Subacute Phase | Moderate | 62.2±10.2 | M5/F10 | Y | MBE | First-ever I stroke diagNsed by neurologist with >10 years’ experience; N imaging standard reported. | The experimental group used the EXOWALK robotic gait trainer (speed <1.8 km/h) for electromechanical walking assistance, in combination with conventional rehabilitation. | 30 min/session, 5×/week | 3weeks | Group A: Conventional rehab: 120 min/day × 6 × 3.0 = 2160 Treadmill (BWS): 30 min × 5 × 4.5 = 675 Total = 2835 METs-min/week | 10MWT |
|  |  | High income | 15 | Lower limb | I | Subacute Phase | Moderate | 61.8±11.1 | M7/F8 | Y | GT |  | The control group used the same treadmill system without visual feedback, with only time and distance displayed. | 30 min/session, 5×/week | 3weeks | Group B: Conventional same Treadmill (BWS): 30 min × 5 × 4.0 = 600 Total = 2760 METs-min/week |  |
| 317 | Irene Aprile 2017^368^ | High income | 6 | Lower limb | Nt reported | Chronic Phase | Moderate | 63±17.79 | M4/F2 | Y | RAT | First stroke diagNsed by neurologist; age ≥18; able to walk with or without miNr assistance; excluded neuro/orthopedic/cognitive disorders. | The robotic group trained with the G-EO end-effector system with partial body weight support, including flat and stair-walking modes. | 45 min/session, 3×/week + 2×/week conventional | 6.5weeks | Robotic Group (G-EO + basic rehab): Conventional: 2×/week × 45min × 6.5weeks → 270 METs/week G-EO (robot): 3×/week × 45min × 6.5weeks × 5.5 METs → ~744 Total = 1014 METs-min/week | 6MWT 10MWT |
|  |  | High income | 8 | Lower limb | Nt reported | Chronic Phase | Moderate | 63±17.79 | M5/F3 | Y | GT |  | The robotic group trained with the G-EO end-effector system with partial body weight support, including flat and stair-walking modes. | 60 min/session, 3×/week + 2×/week conventional | 6.5weeks | Traditional Group: Same conventional rehab = 270 Gait training (stairs, balance): 3×/week × 60min × 6.5weeks × 3.5 METs → 630 Total = 900 METs-min/week |  |
| 318 | H Gok 2008^369^ | Middle- and high-income | 15 | Lower limb | 11I: + 4H | Chronic Phase | Moderate | 55.1±11.4 | M9/F6 | Y | MBE | Stroke diagNsed by neurologist per WHO criteria; confirmed by CT or MRI. | The traditional group received muscle strengthening, balance, stair, and joint mobilization training. Both groups also received standard physical therapy. | 20 min/session, 5×/week | 4weeks | KAT Balance Group: KAT balance board: 20 min × 5 × 3.5 = 350 Conventional rehab: 150 min/day × 5 × 3.0 = 2250 Total = 2600 METs-min/week | FMA-LE |
|  |  | Middle- and high-income | 15 | Lower limb | 13I + 2H | Chronic Phase | Moderate | 59.7±4.8 | M8/F7 | N | NPF |  | The control group received standard therapy, including Bobath, PNF, occupational therapy, and gait training. | 120–180 min/day conventional | 4weeks | Control Group: Same rehab only: 2250 METs-min/week |  |
| 319 | Young-Hyeon Bae 2015^370^ | High income | 10 | Trunk and lower limb function | 5I + 5H | Subacute Phase | Moderate | 67.1±9.2 | M5/F5 | Y | MBE | I or H stroke confirmed by neurologist and imaging (CT/MRI); K-MMSE >24; excluded severe comorbidities. | The experimental group completed balance training followed by motor imagery (MI) involving guided imagery of balance tasks such as standing, single-leg stance, and walking, in a quiet setting using audio instructions. | 30 min/session, 3×/week (4 weeks) | 4weeks | Experimental Group: Dynamic balance training: 20 min × 3 × 3.0 = 180 Motor imagery (guided): 10 min × 3 × 1.5 = 45 Total = 225 METs-min/week | BBS |
|  |  | High income | 10 | Trunk and lower limb function | 5I + 5H | Subacute Phase | Moderate | 67.2±7.8 | M5/F5 | Y | BT |  | The control group completed the same balance tasks without MI, focusing on physical execution only. | 30 min/session, 3×/week (4 weeks) | 4weeks | Control Group: Dynamic balance training only: 30 min × 3 × 3.0 =270 METs-min/week |  |
| 320 | Maria Zafar 2024^371^ | Low- and middle-income | 15 | Trunk and lower limb function | 66.6%I + 33.3%H | Subacute and chronic | Moderate | 52.63±8.78 | M8/F7 | N | MBE | I stroke with hemiparesis diagNsed by neurologist; CT/MRI used; excluded visual neglect or severe cognitive impairment. | The experimental group engaged in dual-task balance training with MI, performing seven tasks combined with cognitive-guided imagery (e.g., sit-to-stand, directional turning). | 30 min/session, 3×/week | 8weeks | Group A (Dual-task + Motor Imagery): Dual-task balance (standing, stepping): 28 min × 3 × 3.5 = 294 Motor imagery: 2 min × 3 × 1.5 = 9 Total = 303 METs-min/week | BBS |
|  |  | Low- and middle-income | 15 | Trunk and lower limb function | 80%I + 20%H | Subacute and chronic | Moderate | 52.56±7.78 | M6/F9 | N | TOT |  | The control group performed the same physical tasks without imagery. | 30 min/session, 3×/week | 8weeks | Group B (Dual-task only): 30 min × 3 × 3.5 = 315 METs-min/week |  |
| 321 | Suruliraj Karthikbabu 2018^372^ | Middle income | 36 | Lower limb | 67%I + 33%H | Chronic Phase | Moderate | 57.2±11.5 | M25/F11 | Y | BT | Chronic stroke (≥6 months); first unilateral cortical stroke; with hemiparesis, N pusher syndrome; independent 10m walking; poor trunk control (TIS <21). | The Plinth group performed trunk control exercises on a stable surface, including bridges, single-leg bridges, trunk rotations, seated lateral flexion, and forward reaching. | 60 min/session, 3×/week | 6weeks | Group A (Plinth core training): Conventional: 60 min × 3 × 3.0 = 540 Plinth-based core: 60 min × 3 × 3.5 = 630 Total = 1170 METs-min/week | 10MWT |
|  |  | Middle income | 36 | Lower limb | 56%I + 44%H | Chronic Phase | Moderate | 54.0±14.1 | M26/F10 | Y | CST |  | The Swiss ball group performed the same exercises on an unstable surface to enhance core activation. | 60 min/session, 3×/week | 6weeks | Group B (Swiss ball core training): Conventional: 540 Swiss ball trunk control: 60 min × 3 × 4.0 = 720 Total = 1260 METs-min/week |  |
|  |  | Middle income | 36 | Lower limb | 58%I + 42%H | Chronic Phase | Moderate | 54.8±12.5 | M23/F13 | N | RC |  | The control group received standard therapy including spasticity control, lower limb strengthening, balance, and gait training. | 60 min/session, 3×/week | 6weeks | Control Group: Only conventional: 60 min × 3 × 3.0 = 540 METs-min/week |  |
| 322 | Giuseppe AnniN 2025^373^ | High income | 17 | Lower limb | I | Chronic Phase | Moderate | 67.8±8.3 | M14/F5 | Y | VT | I stroke confirmed by neurologist via CT/MRI; MMSE ≥19. | On top of CPT, patients received local muscle vibration using the FOV1 Power Club device (30 Hz, 0.2 mm amplitude) targeting the quadriceps tendon. | 60 min CPT + 10 min×3 LMV, 3×/week | 8weeks | Experimental Group: CPT: 60 min × 3 × 4.0 = 720 Local muscle vibration (Quadriceps, 30Hz): 10 min × 3 × 2.5 = 75 Total = 795 METs-min/week | BBS |
|  |  | High income | 17 | Lower limb | I | Chronic Phase | Moderate | 69.4±10.4 | M15/F3 | N | LLT |  | Received conventional physical training including lower limb resistance training (3 sets of 10 reps at 1RM) and treadmill walking at 65–75% HRmax. | 60 min CPT, 3×/week | 8weeks | Control Group: CPT only: 720 METs-min/week |  |
| 323 | Emma Segura 2024^374^ | High income | 20 | Upper limb | 61.5%I + 38.5%H | Chronic Phase | Moderate | 64.2±12.5 | Nt reported | N | MBE | I/H stroke via CT/MRI; upper limb MRC 1–4; MoCA >21; excluded severe neuropsychiatric disorders. | Engaged in app-based individualized music training using electronic keyboards and percussion instruments, with additional online group music therapy. Activities included rhythm synchronization, upper-limb coordination, and scale playing, adjusted for individual difficulty. | 60 min/session, 4×/week | 10weeks | Experimental Group: Music + upper-limb task-based therapy: 60 min × 4 × 3.0 =720 METs-min/week | FMA-UE |
|  |  | High income | 20 | Upper limb | 71.9%I + 28.1%H | Chronic Phase | Moderate | 62.2±12 | Nt reported | N | ULT |  | Performed upper limb functional exercises using daily objects, focused on repeated movements and encouraged paretic limb use. Training was conducted at home under therapist supervision via phone. | 60 min/session, 4×/week | 10weeks | Control Group: ADL functional upper-limb training: 60 min × 4 × 2.5 = 600 METs-min/week |  |
| 324 | Daniel S. Marigold 2005^375^ | High income | 22 | Trunk and lower limb function | Mixed | Chronic Phase | Moderate | 68.1±9.0 | M17/F5 | N | FT | Clinically diagNsed stroke by physician; single stroke event confirmed by CT/MRI. | Completed multisensory dynamic agility drills, such as rapid step transitions, balance under unstable conditions, quick sit-to-stand, high knee movements, and perturbation response training. | 60 min/session, 3×/week | 10weeks | Agility Group: Multimodal gait agility training: 60 min × 3 × 4.5 = 810 METs-min/week | BBS |
|  |  | High income | 26 | Trunk and lower limb function | Mixed | Chronic Phase | Moderate | 67.5±7.2 | M18/F8 | N | MBE |  | Performed slow weight-shifting and stretching exercises, incorporating tai-chi-like balance drills and transitional floor movements. | 60 min/session, 3×/week | 10weeks | Mobility Re-education Group: Slow-paced body shifting + stretching: 60 min × 3 × 3.5 = 630 METs-min/week |  |
| 325 | Birgitta Langhammer 2010^376^ | High income | 21 | Trunk and lower limb function | Nt reported | Chronic Phase | Moderate | 74.0±13.3 | M10/F11 | Y | AE | Stroke confirmed by neurologist via CT/MRI; excluded unstable cardiac status, aphasia, severe orthopedic or surgical history. | Conducted treadmill walking without body support, using handrails for safety. Sessions were therapist-guided with average walking speed at 0.5 m/s. | 12 min/session + 180 min/session conventional, 5×/week | 2.5weeks | Treadmill Group: Conventional rehab: 21 h/week = 1260 min × 3.0 = 3780 Treadmill walk: 60 min/week × 4.0 = 240 Total = 4020 METs-min/week | 6MWT 10MWT |
|  |  | High income | 18 | Trunk and lower limb function | Nt reported | Chronic Phase | Moderate | 75.0±10.4 | M6/F12 | Y | GT |  | Performed supervised outdoor walking using assistive devices if needed, with average walking duration per session longer than the treadmill group. | 29 min/session + 180 min/session conventional, 5×/week | 2.5weeks | Outdoor Walking Group: Conventional rehab same = 3780 Outdoor walk (29 min/day): 145 × 3.5 = 508 Total = 4288 METs-min/week |  |
| 326 | Catherine M. Dean 2012^377^ | High income | 76 | Lower limb | Nt reported | Chronic Phase | Mild to moderate | 66.7±14.3 | M50%/F50% | Y | LLT | Stroke history confirmed by CT/MRI; excluded MMSE <20, aphasia, unstable heart condition. | Participated in group-based lower-limb load-bearing training with home-based practice. Activities included sit-to-stand, stair climbing, single-leg stance, and multidirectional walking to improve balance and gait. | 45 min/session (group 1×/week) + 45 min/session (home 3×/week) | 12months | EG (walking-focused): Group session: 45min × 1 × 4.0 = 180 Home training: 45min × 3 × 4.0 = 540 Total = 720 METs-min/week | 6MWT 10MWT |
|  |  | High income | 75 | Upper limb | Nt reported | Chronic Phase | Mild to moderate | 67.5±10.2 | M53%/F47% | Y | MBE |  | Completed upper-limb and cognitive tasks such as sorting, matching, and puzzles as cognitive and paretic limb engagement exercises. | Therapist-designed, progressive by ability | 12months | EG (upper limb + cognition): Group: 45min × 1 × 2.5 = 112.5 Home: 45min × 3 × 2.5 = 337.5 Total = 450 METs-min/week |  |
| 327 | Olawale 2011^378^ | Low- and middle-income | 20 | Lower limb | Nt reported | Chronic Phase | Mild to moderate | 56.8±6.4 | M13/F8 | Y | AE | Stroke confirmed by neurologist via CT/MRI; eligible for outpatient rehab; excluded cognitive/physical intolerance. | Engaged in treadmill walking and conventional physical therapy. Treadmill walking intensity was monitored using Borg RPE scale. | 60 min/session (incl. 25 min special training), 3×/week | 12weeks | TWET (Treadmill with enhancement): Treadmill: 25min × 3 × 4.5 = 337.5 Conventional: 35min × 3 × 3.0 = 315 Total = 652.5 METs-min/week | 6MWT 10MWT |
|  |  | Low- and middle-income | 20 | Lower limb | Nt reported | Chronic Phase | Mild to moderate | 56.8±8.3 | M13/F8 | Y | GT |  | Performed overground walking within a rectangular pathway. Conventional therapy content was the same. | 60 min/session (incl. 25 min special training), 3×/week | 12weeks | OWET (Overground Walk): Overground walk: 25min × 3 × 4.0 = 300 Conventional: 35min × 3 × 3.0 = 315 Total = 615 METs-min/week |  |
|  |  | Low- and middle-income | 20 | Lower limb | Nt reported | Chronic Phase | Mild to moderate | 57.2±5.9 | M11/F9 | N | RC |  | Performed overground walking within a rectangular pathway. Conventional therapy content was the same. | 60 min/session, 3×/week | 12weeks | Control Group: Conventional only: 60min × 3 × 3.0 = 540 METs-min/week |  |
| 328 | Lamberto Piron 2009^379^ | High income | 18 | Upper limb | I | Chronic Phase | Mild to moderate | 66.0±7.9 | M11/F9 | N | VRG | I stroke confirmed by CT/MRI; excluded De Renzi <62, Token Test errors >40, neglect, comprehension disorder. | Performed virtual reality upper limb training using VRRS.net® system, with live video guidance and five interactive tasks (trajectory tracing, imitation, etc.). | 60 min/session, 5×/week | 4weeks | Tele-rehab Group: 60 min × 5 × 3.5 = 1050 METs-min/week | FMA-UE |
|  |  | High income | 18 | Upper limb | I | Chronic Phase | Mild to moderate | 64.4±7.9 | M10/F8 | N | ULT |  | Received standard in-person therapy targeting object interaction, movement control, and functional integration. | 60 min/session, 5×/week | 4weeks | Control Group: 60 min × 5 × 3.0 =900 METs-min/week |  |
| 329 | Yuting Zhang 2024^380^ | Middle- and high-income | 12 | Trunk and lower limb function | 58.3%H + 41.7%I | Subacute Phase | Moderate | 63.50±8.97 | M10/F2 | Y | RAT | Stroke diagNsed per 2019 Chinese Neurology Conference criteria; confirmed by CT/MRI. | Received additional training with REX exoskeleton robot, including trunk balance, resistance band exercises, and weight-bearing step training. | 60 min/session, 5×/week | 4weeks | Experimental (REX exoskeleton): Conventional rehab: 40 min × 2/day × 5 × 3.5 = 1400 REX robot: 60 min × 1/day × 5 × 4.5 = 1350 Total = 2750 METs-min/week | BBS FMA-LE |
|  |  | Middle- and high-income | 12 | Trunk and lower limb function | 41.7%H + 58.3%I | Subacute Phase | Moderate | 63.83±8.28 | M8/F4 | Y | BT |  | Received additional training with REX exoskeleton robot, including trunk balance, resistance band exercises, and weight-bearing step training. | 60 min/session, 5×/week | 4weeks | Control (Tilt-table training): Conventional same = 1400 Tilt-table: 60 min × 5 × 3.5 = 1050 Total = 2450 METs-min/week |  |
| 330 | Hanna ReyNlds 2021^381^ | High income | 10 | Trunk and lower limb function | 70%I + 30%H | Chronic Phase | Moderate | 54.6±8.9 | M13/F7 | Y | AE | Stroke confirmed by neurologist via CT/MRI; NIHSS/mRS assessed; excluded cardiopulmonary instability, cognitive or mobility issues. | Used tilt tables for weight-bearing standing and directional movements, matched in time and supervision. | 30 min/session, 5×/week (2 face-to-face + 3 home) | 12weeks | Experimental Group (BCT): Conventional: 60 min × 5 × 3.0 = 900 Balance Control Trainer: 20 min × 5 × 4.0 = 400 Total = 1300 METs-min/week | 6MWT 10MWT |
|  |  | High income | 10 | Trunk and lower limb function | 65%I + 35%H | Chronic Phase | Moderate | 60.3±12.9 | M12/F8 | N | RC |  | Performed low-intensity traditional rehabilitation (<40% HRR), including walking, standing balance, bed mobility, and seated strength training. | 30 min/session, 5×/week | 12weeks | Control Group: Conventional only: 900 METs-min/week |  |
| 331 | Shih-Ching Chen 2021^382^ | Middle- and high-income | 15 | Trunk and lower limb function | 46.7%H + 53.3%I | Chronic Phase | Moderate | 53–68 | M9/F6 | N | VRG | First stroke confirmed via CT/MRI; compliant with NTUH rehab criteria; excluded cognitive/emotional/language/cardiac disorders. | Performed low-intensity traditional rehabilitation (<40% HRR), including walking, standing balance, bed mobility, and seated strength training. | 40 min/session, 3×/week | 4weeks | Experimental: 40 min × 3 × 4.0 = ✅ 480 METs-min/week | BBS |
|  |  | Middle- and high-income | 15 | Trunk and lower limb function | 40%H + 60%I | Chronic Phase | Moderate | 52–68 | M9/F6 | N | RC |  | Completed therapist-led rehabilitation with activities like sit-to-stand, balance, walking, and limb function training, also including warm-up and cool-down. | 40 min/session, 3×/week | 4weeks | Control: 40 min × 3 × 3.5 = ✅ 420 METs-min/week |  |
| 332 | Dorian Rose 2011^383^ | High income | 89 | Trunk and lower limb function | Mixed | Acute Phase | Moderate | 68.0±13.1 | M42%/F58% | Y | RC | Stroke confirmed by CT/MRI; met Brooks Rehab criteria; excluded prior stroke, severe comorbidities, amputation, dialysis, psychiatric illness. | Received physical therapy focusing on gait training, bed mobility, balance, and strength of upper and lower limbs. | 90 min/day (60 min main + 30 min support), 5×/week | 20days | Group A (Low-intensity, less structured): 90 min × 5 × 3.5 = 1575 METs-min/week | BBS FMA-LE |
|  |  | High income | 67 | Trunk and lower limb function | Mixed | Acute Phase | Moderate | 67.6±15.3 | M43%/F57% | Y | ESX |  | Underwent circuit training with four functional task stations (e.g., bed mobility, sit-to-stand, stair climbing), each session emphasizing repetition and progressive difficulty, with tasks jointly set and recorded by therapists and patients. | 90 min/day (60 min main + 30 min support), 5×/week | 20days | Group B (Multitask + aerobic/RT): 90 min × 5 × 4.0 = 1800 METs-min/week |  |
| 333 | So Hyun Lee 2012^384^ | High income | 20 | Trunk and lower limb function | 70%I + 30%H | Chronic Phase | Moderate | 53.75±11.29 | M13/F7 | Y | BT | I/H stroke via CT/MRI; MMSE ≥24; independent walking ≥10m; excluded unstable condition and neuro/muscle disorders. | In addition to routine therapy, performed balance control training using a Balance Control Trainer (BCT), including weight-shifting with visual feedback, knee flexion control, and interactive video game-based balance tasks. | 80 min/session, 5×/week | 4weeks | Experimental Group (BCT): Conventional: 60 min × 5 × 3.0 = 900 BCT: 20 min × 5 × 4.0 = 400 Total = 1300 METs-min/week | 10MWT BBS |
|  |  | High income | 20 | Trunk and lower limb function | 65%I + 35%H | Chronic Phase | Moderate | 54.10±11.13 | M12/F8 | N | RC |  | In addition to routine therapy, performed balance control training using a Balance Control Trainer (BCT), including weight-shifting with visual feedback, knee flexion control, and interactive video game-based balance tasks. | 60 min/session, 5×/week | 4weeks | Control Group: Conventional only: 900 METs-min/week |  |
| 334 | Mukul Talaty 2023^385^ | High income | 15 | Lower limb | 73.3%I + 26.7%H | Acute Phase | Moderate | 63.2±10.0 | M12/F3 | Y | RAT | Stroke confirmed by neurologist with CT/MRI; all patients able to tolerate upright position ≥12 min; excluded severe cognitive/cardiopulmonary issues. | Underwent robotic-assisted gait training with Lokomat®, with individualized guidance force and treadmill speed adjustments to maximize walking effort. | 45 min/session, up to 4×/week (in addition to 3 h/day conventional) | 21.2days | Lokomat Group: Conventional rehab: 1.5h/day × 5 × 3.0 METs = 4050 Lokomat: 45min × 2.7/week × 4.0 = 486 Total = 4536 METs-min/week | 10MWT |
|  |  | High income | 15 | Lower limb | 73.3%I + 26.7%H | Acute Phase | Moderate | 53.7±16.8 | M10/F5 | Y | GT |  | Underwent robotic-assisted gait training with Lokomat®, with individualized guidance force and treadmill speed adjustments to maximize walking effort. | 45 min/session, up to 4×/week (in addition to 3 h/day conventional) | 27.5days | CGT Group: Conventional rehab: same = 4050 Conventional gait training: 45min × 3.1/week × 3.5 = 488.25 Total = 4538.25 METs-min/week |  |
| 336 | Ahmad Sahely 2024^386^ | High income | 12 | Trunk and lower limb function | 25%H + 75%I | Chronic Phase | Mild to moderate | 63±16.53 | M6/F6 | Y | MBE | Stroke confirmed via NHS standards and CT/MRI; MoCA screened; excluded severe cognitive/language disorders, spasticity, medical instability. | Participated in a self-management program including goal-setting education, biweekly Zoom group sessions, individual coaching calls, step counter usage, and home exercises, with all materials provided remotely or in person. | Individual plan home training + biweekly group & follow-up | 3months | SIMS Group: Rehab: 30min × 2/day × 6 × 3.0 = 1080 SIMS training (home + education + monitoring): 30min × 2/day × 6 × 4.5 = 1620 Total = 2700 METs-min/week | 6MWT 10MWT |
|  |  | High income | 12 | Trunk and lower limb function | 17%H + 83%I | Chronic Phase | Mild to moderate | 71±13.04 | 8M/4F | Y | RC |  | Participated in a self-management program including goal-setting education, biweekly Zoom group sessions, individual coaching calls, step counter usage, and home exercises, with all materials provided remotely or in person. | N specific plan, NHS standard care | 3months | Control Group: Rehab: 1080 METs-min/week Education (one-off session): Nt included |  |
| 337 | Genevieve Hendrey 2018^387^ | High income | 15 | Lower limb | 66.7%I + 66.7%H | Subacute Phase | Mild to moderate | 50.4±17.0 | Nt reported | Y | RT | First stroke confirmed by neurologist and CT/MRI; ≥10% knee extension strength asymmetry; able to walk ≥14m. | Engaged in power training including jump squats, single-leg hops, bounding, and high-speed hip flexion with pulley resistance, emphasizing movement speed and skill development. | 45 min/session, 3×/week | 6weeks | Ballistic Strength Training Group (BST): Rehab: 3 × 45min × 3.0 = 405 BST: 3 × 45min × 6.0 = 810 Total = 1215 METs-min/week | 10MWT |
|  |  | High income | 15 | Lower limb | 53.3%I + 46.7%H | Subacute Phase | Mild to moderate | 49.3±18.6 | Nt reported | N | RC |  | Performed standard rehabilitation exercises, including gait, static/dynamic balance, squatting, stair training, and aerobic tasks. | 45 min/session, 3×/week | 6weeks | Control Group: Rehab: 3 × 45min × 3.0 = 405METs-min/week |  |
| 338 | So Jung Lee 2022^388^ | High income | 7 | Trunk | 57.1%I + 42.9%H | Subacute and chronic | Moderate | 54.71±17.08 | M5/F2 | Y | MBE | First stroke confirmed by CT/MRI; able to sit and walk ≥10m; tolerate 40-min activity; excluded cognitive, visual, orthopedic, neuro conditions. | Performed standard rehabilitation exercises, including gait, static/dynamic balance, squatting, stair training, and aerobic tasks. | 40 min/session, 2×/week (total 6 sessions) + conventional | 3weeks | Dance for PD Group: Rehab: 30min × 5 × 3.0 = 450 Dance: 40min × 2 × 3.5 = 280 Total = 730 METs-min/week | BBS |
|  |  | High income | 7 | Trunk | 71.4%I + 28.6%H | Subacute and chronic | Moderate | 61.14±14.45 | M5/F2 | N | RC |  | Participated in remote dance therapy via Zoom, including seated warm-ups, chair dancing, balance-based standing dances, spatial movement (e.g., Cha-cha, Box Step), and closing rituals. | Conventional therapy only | 3weeks | Control Group: Rehab: 30min × 5 × 3.0 = 450METs-min/week |  |
| 339 | Hyeong-Min Kim 2024^389^ | High income | 9 | Lower limb | I | Chronic Phase | Moderate | 65.78±12.60 | M5/F4 | N | ESX | I stroke confirmed by neurologist via CT/MRI; excluded H stroke, gait-affecting musculoskeletal/neuro disorders. | Received standard physical therapy without dance elements. | 20 min/session, 3×/week | 4weeks | tDCS Group and Control: Gait training: 20min × 3 × 3.5 = 210 METs-min/week tDCS (Nn-physical): Nt counted | 10MWT BBS |
|  |  | High income | 8 | Lower limb | I | Chronic Phase | Moderate | 57.13±9.49 | M5/F3 | N | GT |  | Received 20 minutes of active aNdal tDCS targeting the tibialis anterior motor cortex (2.0 mA), combined with 20 minutes of gait training including level ground, stairs, side-stepping, and backward walking. | 20 min/session, 3×/week | 4weeks | tDCS Group and Control: Gait training: 20min × 3 × 3.5 = 210 METs-min/week tDCS (Nn-physical): Nt counted |  |
| 340 | Adi ToledaN-Zarhi 2011^390^ | High income | 14 | Whole-body function | I | Subacute Phase | Mild | 65±10 | M78.56%/F21.44% | Y | FT | I stroke confirmed by neurologist via CT/MRI; mRS ≤2; excluded hypertensive crisis, unstable angina, arrhythmia, heart/lung disease. | Underwent identical gait training but received sham tDCS with only brief initial/end stimulation. | 35–55 min/session aerobic, 2×/week + 45–55 min group, 1×/week | 6weeks | Intervention Group: Aerobic combo: 45min × 2 × 5.5 = 495 Strength/coordination: 50min × 1 × 3.5 = 175 Total = 670 METs-min/week | 6MWT |
|  |  | High income | 14 | Whole-body function | I | Subacute Phase | Mild | 65±12 | M71.4%/F28.6% | N | RC |  | Followed home training instructions and unsupervised physical activity. | 30 min/session, 5×/week | 6weeks | Control Group: Home flexibility: 30min × 5 × 3.0 = 450 METs-min/week |  |
| 341 | Susan M. Linder 2021^391^ | High income | 16 | Upper limb | Nt reported | Chronic Phase | Moderate | 51±12 | M75%/F25% | Y | FT | Single stroke confirmed by CT/MRI; excluded recent CV events, severe cardiopulmonary or musculoskeletal impairments. | Completed assisted aerobic cycling at a cadence 30% faster than baseline (electrically driven) followed by repetitive upper limb task training. | 90 min/session, 3×/week | 8weeks | FE+RTP Group: Forced cycling: 45min × 3 × 6.0 = 810 Repetitive task practice (RTP): 45min × 3 × 2.0 = 270 Total = 1080 METs-min/week | 6MWT |
|  |  | High income | 14 | Upper limb | Nt reported | Chronic Phase | Moderate | 60±14 | M64%/F36% | Y | AE |  | Performed the same structure but without motor assistance during cycling. | 90 min/session, 3×/week | 8weeks | FE+RTP Group: Forced cycling: 45min × 3 × 6.0 = 810 Repetitive task practice (RTP): 45min × 3 × 2.0 = 270 Total = 1080 METs-min/week |  |
|  |  | High income | 13 | Upper limb | Nt reported | Chronic Phase | Moderate | 59±11 | M92%/F8% | N | TOT |  | Performed the same structure but without motor assistance during cycling. | 90 min/session, 3×/week | 8weeks | RTP Only Group (Control): ✅ 270 METs-min/week |  |
| 342 | Susan M. Linder 2019^392^ | High income | 16 | Upper limb | Nt reported | Chronic Phase | Mild to moderate | 51±12 | M12/F4 | N | RAT | Single stroke confirmed by CT/MRI; CPX tested; excluded severe cardiac/respiratory or cognitive limitations. | Conducted assisted cycling at 30% above baseline cadence with target HRR of 60–80%, followed by functional upper limb task training. | 90 min/session (45 min cycling/education + 45 min task repetition), 3×/week | 8weeks | FE+RTP Group: Forced cycling: 45min × 3 × 4.5 = 607.5 RTP: 45min × 3 × 3.0 = 405 Total = 1012.5 METs-min/week | FMA-UE |
|  |  | High income | 16 | Upper limb | Nt reported | Chronic Phase | Mild to moderate | 60±14 | M10/F6 | N | AE |  | Performed self-paced cycling without motor support; upper limb training was the same. | 90 min/session, 3×/week | 8weeks | Voluntary Exercise + RTP (VE+RTP): Cycling: 45min × 3 × 4.0 = 540 RTP: 45min × 3 × 3.0 = 405 Total = 945 METs-min/week |  |
|  |  | High income | 8 | Upper limb | Nt reported | Chronic Phase | Mild to moderate | 58±12 | M7/F1 | N | ULT |  | Performed self-paced cycling without motor support; upper limb training was the same. | 90 min/session, 3×/week | 8weeks | RTP-Only Group: RTP:45min × 3 × 3.0 = 405 METs-min/week |  |
| 343 | Susan M. Linder 2023^393^ | High income | 30 | Upper limb | Nt reported | Chronic Phase | Moderate | 60.8±11.5 | M7/F3 | N | RAT | Single stroke confirmed by CT/MRI; eligible for CPX; excluded severe cardiac events in last 3 months or contraindications to exercise. | Performed assisted cycling at ≥75 rpm supported by an electric motor, followed by functional upper limb repetitive training with 3–5 tasks, 60–90 repetitions each. | 90 min/session, 3×/week | 8weeks | FE+RTP Group: Forced cycling: 45min × 3 × 6.0 = 810 RTP: 45min × 3 × 3.5 = 472.5 Total = 1282.5 METs-min/week | 6MWT FMA-UE |
|  |  | High income | 30 | Upper limb | Nt reported | Chronic Phase | Moderate | 60.2±9.8 | M6/F4 | N | ULT |  | Completed 90 minutes of upper limb repetitive tasks without cycling. | 90 min/session, 3×/week | 8weeks | Control (RTP Only): RTP (72.7min average): 72.7min × 3 × 3.5 = 766.5 METs-min/week |  |
| 345 | Patcharee Kooncumchoo 2022^394^ | Middle- and high-income | 15 | Lower limb | 11I + 4H | Chronic Phase | Moderate | 64.33±7.68 | M10/F5 | N | BT | Single stroke confirmed by neurologist via CT/MRI; able to walk ≥3m and follow commands; excluded CV instability and orthopedic limits. | Completed 90 minutes of upper limb repetitive tasks without cycling. | 60 min/session (30 min upper limb + 30 min walking), 3×/week | 8weeks | I-Walk Group: Upper limb + sit-stand: 30min × 3 × 3.0 = 270 I-Walk: 30min × 3 × 4.5 = 405 Total = 675 METs-min/week | FMA-LE |
|  |  | Middle- and high-income | 15 | Lower limb | 13I + 2H | Chronic Phase | Moderate | 63.53±12.16 | M10/F5 | N | RC |  | Received the same structure, replacing I-Walk with traditional therapist-guided overground gait training. | 60 min/session (30 min upper limb + 30 min walking), 3×/week | 8weeks | Control Group: Sit-stand: 30min × 3 × 3.0 = 270 Overground gait: 30min × 3 × 3.5 = 315 Total = 585 METs-min/week |  |
| 348 | Daniele Munari 2016^395^ | High income | 8 | Lower limb | Nt reported | Chronic Phase | Moderate | 60.87±5.77 | M7/F1 | N | HIIT | Stroke confirmed by MRI/CT; MMSE ≥20; excluded angina, cardiac instability, hypertension, arrhythmia, expressive aphasia. | Performed high-intensity interval treadmill walking with 5 sets of 5 minutes at 85–95% VO₂peak, interspersed with 3-minute rest periods at 50% VO₂peak. Sessions included warm-up and cooldown. | 50–60 min/session (with warm-up & cool-down), 3×/week | 12weeks | High-Intensity Group: Warm-up: 10min × 4.5 = 45 Intervals: 25min × 9.0 = 225 Recovery: 12min × 5.0 = 60 Cool-down: 5min × 4.5 = 22.5 Single session = 352.5 × 3 =1057.5 METs-min/week | 6MWT 10MWT |
|  |  | High income | 8 | Lower limb | Nt reported | Chronic Phase | Moderate | 61.71±11.27 | M7 | N | AE |  | Performed high-intensity interval treadmill walking with 5 sets of 5 minutes at 85–95% VO₂peak, interspersed with 3-minute rest periods at 50% VO₂peak. Sessions included warm-up and cooldown. | 50–60 min/session, 3×/week | 12weeks | Moderate Group: Total per session = 205 × 3 = 615 METs-min/week |  |
| 349 | Zhizhong Zhu 2016^396^ | Middle- and high-income | 14 | Trunk and lower limb function | 71%I + 29%H | Chronic Phase | Moderate | 56.6±6.9 | M12/F2 | N | WA | Chronic stroke (≥6 months); gait and balance disorders; Brunnstrom LE stage IV–V. | Conducted continuous low-intensity treadmill walking at 60% VO₂peak with fixed slope and included warm-up and cooldown periods. | 45 min/session, 5×/week | 4weeks | Aquatic Group: 45min × 5 × 5.0 = 1125 METs-min/week | BBS |
|  |  | Middle- and high-income | 14 | Trunk and lower limb function | 79%I + 21%H | Chronic Phase | Moderate | 57.1±8.6 | M10/F4 | N | FT |  | Used the same structure and sequence, replacing aquatic components with land-based strength, core, and treadmill training. | 45 min/session, 5×/week | 4weeks | Land Training Group: 45min × 5 × 4.0 = 900 METs-min/week |  |
| 351 | Mohammad Saadatnia 2020^397^ | Middle- and high-income | 20 | Upper and lower limbs | I | Subacute Phase | Moderate to severe | 62±12.4 | M9/F11 | N | MBE | Unilateral I stroke per 2019 Chinese criteria; CT/MRI confirmed; excluded hypertension, heart disease, cognitive issues, PE, dependency. | Used the same structure and sequence, replacing aquatic components with land-based strength, core, and treadmill training. | 60 min/session ×2/day (120 min/day) | 12weeks | Home DVD Group: 60min × 2/day × 7 × 3.0 = 2520 METs-min/week | FMA-LE FMA-UE |
|  |  | Middle- and high-income | 20 | Upper and lower limbs | I | Subacute Phase | Moderate to severe | 66±10.3 | M8/F12 | N | RC |  | Provided with a 3-month home rehabilitation DVD plan, covering passive, active, resistance, and endurance exercises, with telephonic follow-up and home visits for support. | Outpatient prescription, frequency uncertain | 12weeks | Inpatient + Home Maintenance Group: Inpatient: 30min × 7 × 3.0 = 630 Home: 30min × 2 × 3.0 = 180 Estimated Total = 810 METs-min/week |  |
| 352 | Buket Akıncı 2025^398^ | Middle- and high-income | 17 | Upper limb | 70.6%I + 29.4%H | Chronic Phase | Moderate | 18–65 | M 64.7%/F 35.3% | Y | RAT | Unilateral stroke (I or H) confirmed by CT/MRI; excluded major systemic, cardio, neuro, psych comorbidities. | Continued receiving standard physiotherapy or occupational therapy as per physician’s advice, without structured home exercise or follow-up. | 45 min/session robot 2×/week + conventional 5×/week | 6weeks | Robotic Rehab Group (RR): Conventional rehab: 30min × 5 × 3.0 = 450 ExoRehab X: 45min × 2 × 3.5 = 315 Total = 765 METs-min/week | 6MWT |
|  |  | Middle- and high-income | 17 | Upper limb | 82.4%I + 17.6%H | Chronic Phase | Moderate | 46–65 | M 58.8%/F 41.2% | N | RC |  | Received conventional rehabilitation combined with ExoRehab X upper limb robotic game training, focusing on shoulder, elbow, and wrist coordination. | Same conventional 5×/week without robot | 6weeks | Control Group: Conventional rehab: 30min × 5 × 3.0 = 450METs-min/week |  |
| 353 | Maria Bergqvist 2023^399^ | High income | 15 | Lower limb | 73%I + 20%H + 7%Mixed | Chronic Phase | Moderate | 59–66 | M10/F5 | Y | RAT | CT/MRI-confirmed stroke per prior RCT; functional gait impairment; excluded severe aphasia (unable to complete MoCA). | Received conventional rehabilitation including weight-bearing, stretching, upper limb strengthening, balance, and neuromuscular stimulation. | 90 min/session, 3×/week | 6weeks | HAL Group: HAL: 45min × 3 × 5.0 = 675 Floor training: 30min × 3 × 3.5 = 315 Total = 990 METs-min/week | 6MWT 10MWT BBS |
|  |  | High income | 16 | Lower limb | 56%I + 44%H | Chronic Phase | Moderate | 58–68 | M10/F6 | N | RC |  | Underwent 60 minutes of Hybrid Assistive Limb (HAL) treadmill gait training with body weight support and EMG-based control, followed by 30 minutes of conventional therapy. | 90 min/session, 3×/week | 6weeks | Control Group: Floor-based therapy: 90min × 3 × 4.0 = 1080 METs-min/week |  |
|  |  | High income | 14 | Lower limb | 71%I + 29%H | Chronic Phase | Moderate | 47–69 | M12/F2 | N | NE |  | Received 90-minute therapist-guided overground gait training including treadmill, walking aids, and lower limb exercises. | 0 | 6weeks | 0 |  |
| 354 | Jennifer L. Moore 2020^400^ | High income | 54 | Trunk and lower limb function | 76%I + 24%H | Subacute Phase | Moderate | 73±10 | M35/F20 | N | AE | I/H stroke via CT/MRI; excluded cardiopulmonary/metabolic abNrmality, psychiatric disorder, gait ability <50m. | Continued regular daily activities with N structured intervention. | ~45–60 min/session, 5–6×/week | 35±17days | High-Intensity Gait Training Group: 45min × 5 × 6.0 = 1350 METs-min/week | 6MWT BBS |
|  |  | High income | 56 | Trunk and lower limb function | 76%I + 24%H | Subacute Phase | Moderate | 74±14 | M29/F27 | N | RC |  | Performed focused gait training with treadmill, obstacles, and stairs under heart rate monitoring to ensure 70%–85% HRmax intensity. | ~45–60 min/session, 5–6×/week | 23±9.7days | Control Group: 45min × 5 × 3.5 =787.5 METs-min/week |  |
| 355 | Elaine Menezes-Oliveira 2024^401^ | Middle income | 21 | Trunk and lower limb function | 61%I + 39%H | Chronic Phase | Moderate | 54.0±12.33 | M52%/F48% | Y | FT | CT/MRI-confirmed stroke (I/H); met rehab screening criteria; excluded surgery, chemical block, severe speech issues. | Received standard physiotherapy without heart rate guidance or intensity tracking. | 150–180 min/session (2.5–3 h), daily | 15days | LE-CIMT Group: Intensive rehab: 3 h/day × 5 days/week × 4.5 METs = 4050 METs-min/week | 6MWT 10MWT |
|  |  | Middle income | 21 | Trunk and lower limb function | 76%I + 24%H | Chronic Phase | Moderate | 53.24±13.05 | M33%/F67% | N | RC |  | Participated in 3-hour daily sessions including a 30-minute behavioral contract and 2.5-hour shaping-based functional task training. | 150–180 min/session, daily | 15days | Control Group: Conventional rehab (30min × 5/week × 3.0) = 450 METs-min/week |  |
| 356 | Maxime Térémetz，2022^402^ | High income | 19 | Upper limb and trunk | Nt reported | Chronic Phase | Mild to moderate | 49.7–61.9 | M13/F6 | Y | VRG | Clinically diagNsed single stroke; excluded cerebellar stroke, seizure history, severe cognitive/perceptual deficits; able to raise paretic hand to mouth. | Received 2.5-hour daily individualized therapy involving traditional gait, weight-bearing, and balance exercises without use of techNlogy or electric stimulation. | 60 min/session, 3×/week | 4weeks | Wii Group: Wii training: 60min × 3/week × 4.0 = 720 METs-min/week | FMA-UE |
|  |  | High income | 21 | Upper limb and trunk | Nt reported | Chronic Phase | Mild to moderate | 50.5–61.9 | M11/F10 | N | RC |  | Engaged in 45 minutes of Nintendo Wii-based rehab using tennis, golf, and boxing games, emphasizing large arm movements and avoiding compensatory patterns. | 60 min/session, 3×/week | 4weeks | Control Group:  Conventional upper limb rehab: 60min × 3/week × 3.0 = 540 METs-min/week |  |
| 357 | Elizabeth D. Thompson，2024^403^ | High income | 89 | Lower limb | I | Chronic Phase | Moderate | 63±1.27 | M41/F48 | N | AE | Single stroke by history; excluded cerebellar stroke and cognitive impairment; NIHSS 1b≤1, 1c=0; daily steps <8000. | Performed therapist-guided upper limb activities including passive/active movement and task-based reaching or gripping. | ~40 min/session, 2–3×/week | 12weeks | FAST Group: High-intensity treadmill: 40min × 3 × 6.0 = 720 METs-min/week | 6MWT 10MWT |
|  |  | High income | 81 | Lower limb | I | Chronic Phase | Moderate | 62±1.44 | M39/F42 | N | RC |  | Performed 30 minutes of treadmill walking with 10 minutes of functional overground gait tasks under heart rate reserve targeting 70–80%. | ~40 min/session, 2–3×/week | 12weeks | SAM Group: Step goals: 30min × 5 × 3.0 = 450 METs-min/week |  |
|  |  | High income | 80 | Lower limb | I | Chronic Phase | Moderate | 62±1.46 | M36/F44 | N | MBE |  | Wore Fitbit and engaged in goal-setting behavioral coaching using motivational interviewing. | ~40 min/session, 2–3×/week | 12weeks | FAST + SAM Total: 1170 METs-min/week |  |
| 358 | Carina Salgueiro，2022^404^ | High income | 15 | Trunk | 66.7%I + 33.3%H | Chronic Phase | Moderate | 57.27±14.35 | M10/F5 | Y | CST | Clinician-diagNsed stroke; S-TIS ≤10; able to follow commands; excluded recurrent stroke, neuro disorders, limb deformities; mobile device familiarity required. | Combined treadmill training and behavior intervention with activity monitoring. | ~30–40 min/session, daily (recommend 5×/week); additional 60 min/session, 2×/week | 12weeks | Core Stability Training Group (CSE): Conventional rehab: 60min × 2/week × 3.0 = 360 Core training: 30min × 5/week × 3.5 = 525 Total = 885 METs-min/week | BBS |
|  |  | High income | 15 | Trunk | 66.7%I + 33.3%H | Chronic Phase | Moderate | 64.53±9.40 | M10/F5 | N | RC |  | Received conventional therapy including stretching, active/passive movement, postural control, and aerobic activities. | 30 min/session, 5×/week | 12weeks | Control Group: Conventional rehab: 60min × 2/week × 3.0 = 360 METs-min/week |  |
| 359 | Michal Katz-Leurer，2006^405^ | High income | 10 | Lower limb | I | Subacute Phase | Moderate | 59±8 | M8/F2 | Y | AE | First I stroke diagNsed clinically; excluded brainstem/bilateral lesion, poor postural control, cardiac issues, β-blockers, pain, musculoskeletal problems. | Received conventional therapy including stretching, active/passive movement, postural control, and aerobic activities. | 5×/week conventional, time Nt specified | 3weeks | Cycling + Rehab Group: Cycling: 30min × 5 × 3.5 = 525 Conventional rehab: 45min × 5 × 3.0 = 675 Total = 1200 METs-min/week | FMA-LE |
|  |  | High income | 14 | Lower limb | I | Subacute Phase | Moderate | 65±9 | M5/F9 | N | NPF |  | Received standard rehabilitation including Bobath-based physiotherapy, occupational and speech therapy, and group exercise. | 5×/week conventional, time Nt specified | 3weeks | Control Group: Conventional rehab: 45min × 5 × 3.0 = 675 METs-min/week |  |
| 360 | Sung Min Son，2014^406^ | High income | 14 | Lower limb | 7I + 7H | Chronic Phase | Moderate | 57.4 | M8/F6 | Y | RT | History-confirmed stroke; Brunnstrom ≥III; K-MMSE ≥20; independent 10m walk; excluded joint issues, hemiaNpia. | Performed multi-joint resistance training using a leg press machine, targeting 70% 1RM with progressive overload. | 30 min/session, 5×/week | 6weeks | Leg Press Group: Rehab: 30min × 5 × 3.0 = 450 Resistance training: 30min × 5 × 4.5 = 675 Total = 1125 METs-min/week | BBS |
|  |  | High income | 14 | Lower limb | 8I + 6H | Chronic Phase | Moderate | 56.6 | M7/F7 | Y | LLT |  | Performed multi-joint resistance training using a leg press machine, targeting 70% 1RM with progressive overload. | 30 min/session ×2/day, 6×/week | 6weeks | Control Group: Rehab: 30min × 5 × 3.0 = 450 Low-load movements: 30min × 5 × 2.5 = 375 Total = 825 METs-min/week |  |
| 363 | Yijun Wang，2024^407^ | Middle- and high-income | 40 | Upper and lower limbs | 75%I + 25%H | Subacute Phase | Moderate | 62.96±3.09 | M22/F18 | Y | MBE | I stroke per 2021 EAN/EFNS guidelines; age 45–70; N cognitive issues; mobile access; excluded CHF, cancer, HTN crisis, dementia. | Used a mobile app and leg-worn device to perform supervised walking twice daily, with real-time physiological monitoring and remote adjustment. | 30 min/session ×2/day, 6×/week | 12weeks | Wearable Remote Group: Rehab: 30min × 2/day × 6 × 3.0 = 1080 Wearable intervention: 30min × 2/day × 6 × 4.5 = 1620 Total = 2700 METs-min/week | BBS FMA-LE |
|  |  | Middle- and high-income | 40 | Upper and lower limbs | 80%I + 20%H | Subacute Phase | Moderate | 63.01±2.99 | M25/F15 | N | RC |  | Used a mobile app and leg-worn device to perform supervised walking twice daily, with real-time physiological monitoring and remote adjustment. | 20 min/session ×2/day, 6×/week | 12weeks | Control Group: 20min × 2/day × 6 × 3.0 = 720 METs-min/week |  |
| 364 | Ulla-Britt Flansbjer，2012^408^ | High income | 11 | Lower limb | Nt reported | Chronic Phase | Moderate | 66±4 | Nt reported | N | RT | Historical stroke (RCT 2005–2006); excluded new disabling events; imaging status Nt reported. | Participated in supervised progressive resistance training (PRT) targeting knee extensors and flexors using HUR leg extension/flexion machines (air resistance 10 bar) at 80% of maximal strength. | 30 min/session, 2×/week | 10weeks | Intervention: 45min × 2 × 6.0 = 540 METs-min/week | 6MWT 10MWT |
|  |  | High income | 7 | Lower limb | Nt reported | Chronic Phase | Moderate | 66±4 | Nt reported | N | NE |  | Continued daily life activities without additional interventions. | 30 min/session, 5×/week | 10weeks | Control: 150min/week × 1.5 METs = 225 METs-min/week |  |
| 367 | Pierce Boyne，2023^409^ | High income | 27 | Lower limb | 55.6%I + 44.4%H | Chronic Phase | Moderate | 63.8±9.9 | M16/F11 | N | HIIT | Single stroke (6mo–5yr); age 40–80; walking ≥10m unassisted, ≤1.0 m/s; excluded cardiac instability, spasticity, ataxia, cognitive or communication deficits. | Performed interval walking at maximal safe speed for 30 seconds alternated with 30–60 seconds rest, aiming for >60% heart rate reserve (HRR), including warm-up and cool-down. | 45 min/session, 3×/week | 12weeks | HIIT Group: 45min × 3/week × 6.3 = 850.5 METs-min/week | 6MWT 10MWT |
|  |  | High income | 28 | Lower limb | 67.9%I + 32.1%H | Chronic Phase | Moderate | 61.5±9.9 | M20/F8 | N | AE |  | Engaged in continuous walking, starting at 40% HRR with gradual increase to a maximum of 60%, including warm-up and cool-down. | 45 min/session, 3×/week | 12weeks | MAT Group: 45min × 3/week × 3.5 = 472.5 METs-min/week |  |
| 369 | Keun-Jo Kim，2018^410^ | High income | 13 | Lower limb | 53.8%I + 46.2%H | Chronic Phase | Moderate | 52.62±9.84 | M8/F5 | N | MBE | Stroke >6mo; K-MMSE ≥24; excluded cerebellar/vestibular/visual deficits or gait-affecting drugs; treadmill ≥30min tolerated. | Conducted treadmill training with harness support, progressively increasing speed (+0.1 km/h each session) while performing 12 types of cognitive tasks such as reverse spelling and storytelling under dual-therapist supervision. | 30 min/session, 5×/week | 4weeks | Dual-task Group: Treadmill + cognitive task: 30min × 5/week × 4.0 = 600 METs-min/week | 10MWT |
|  |  | High income | 13 | Lower limb | 61.5%I + 38.5%H | Chronic Phase | Moderate | 56.15±10.82 | M7/F6 | N | AE |  | Performed treadmill training with identical speed progression and harness support, without cognitive tasks. | 30 min/session, 5×/week | 4weeks | Control Group: Treadmill only: 30min × 5/week × 3.3 = 495 METs-min/week |  |
| 370 | Monica C. Serra，2022^411^ | High income | 20 | Lower limb | Nt reported | Chronic Phase | Moderate | 63±1 | M16/F4 | N | AE | Stroke by history; required 3-min treadmill walking at 0.3 mph; excluded N hemiparesis, organ disease, diabetes, anemia, orthopedic/medical issues. | Performed treadmill training with progression from 15 to 50 minutes at intensities ranging from 40–70% HRR, in small supervised groups guided by exercise physiologists. | ≤50 min/session, 3×/week | 6months | Aerobic walking (moderate-high): 50min × 3/week × 6.0 = 900 METs-min/week | 6MWT 10MWT BBS |
|  |  | High income | 19 | Lower limb | Nt reported | Chronic Phase | Moderate | 68±2 | M13/F6 | N | BT |  | Conducted full-body stretching and balance training including static and dynamic exercises targeting neck, upper and lower limbs, and functional activities. | 50 min/session, 2×/week | 6months | Light mobility/stretching: 50min × 2/week × 2.5 = 250 METs-min/week |  |
| 371 | Pamela Duncan，2003^412^ | High income | 44 | Whole-body function | 89%I + 11%H | Subacute Phase | Moderate | 68.5±9.0 | M23/F21 | Y | FT | WHO-defined stroke, CT/MRI confirmed; age ≥50, 30–150 days post-stroke; FMA 27–90; Orpington 2.0–5.2; MMSE ≥16; excluded serious illness. | Completed structured home rehabilitation under therapist supervision, including ROM, PNF strength training with TheraBands, balance tasks (e.g., step-up, chair rise), functional upper limb activities, and progressive stationary cycling. | 90 min/session, 3×/week | 12–14weeks | Wearable Group: Rehab: 30min × 2/day × 6 × 3.0 = 1080 Wearable-assisted: 30min × 2/day × 6 × 4.5 = 1620 Total = 2700 METs-min/week | 6MWT 10MWT BBS FMA-LE FMA-UE |
|  |  | High income | 48 | Whole-body function | 89%I + 11%H | Subacute Phase | Moderate | 70.2±11.4 | M27/F21 | N | RC |  | Received standard community rehabilitation based on physician referral or verbal advice, with varied access to physical/occupational therapy. | Similar total time, variable frequency | 12–14weeks | Control Group: 20min × 2/day × 6 × 3.0 = 720 METs-min/week |  |
| 372 | Wim Saeys，2012^413^ | High income | 18 | Trunk and lower limb function | 15I + 3H | Subacute Phase | Moderate | 61.94±13.83 | M9/F9 | Y | FT | CT/MRI-confirmed first stroke (~35 days post); rehab planned; excluded age >85, back pain, orthopedic/neurological disorders, communication issues. | Added 16 hours of trunk-focused training on top of Bobath-based rehabilitation, including selective trunk muscle exercises, functional activities, and visual feedback; partially involved dual-task conditions. | 30 min/session, 4×/week | 8weeks | Trunk Training Group: Conventional rehab: 60min × 5/week × 3.0 = 900 Trunk-specific training: 30min × 4/week × 4.0 = 480 Total = 1380 METs-min/week | BBS |
|  |  | High income | 15 | Trunk and lower limb function | 11I + 4H | Subacute Phase | Moderate | 61.07±9.01 | M8/F7 | Y | RC |  | Received 16 hours of upper limb passive movements and TENS on the shoulder without trunk or postural control elements, alongside Bobath therapy. | 30 min/session, 4×/week | 8weeks | Upper Limb Training Group: Conventional rehab: 60min × 5/week × 3.0 = 900 Passive + NMES upper limb: 30min × 4/week × 2.0 = 240 Total = 1140 METs-min/week |  |
| 374 | Kelly P. Westlake，2025^414^ | High income | 22 | Upper limb | Nt reported | Subacute and chronic | Mild to moderate | 57.6 | M10/F12 | N | VRG | I/H stroke ≥3mo; hand motion ≥3 inches; excluded cerebellar stroke, Botox within 3mo; device/internet usage required. | Used a LifeCIT-based web platform to select six personalized daily activities stratified by functional level, with options to add active training and games. The system adjusted training tasksweekly based on performance. | ≥60 min/day, 5×/week | 6weeks | STRONG (Web-based platform): 60min/day × 5/week × 4.5 = 1350 METs-min/week | FMA-UE |
|  |  | High income | 21 | Upper limb | Nt reported | Subacute and chronic | Mild to moderate | 58.4 | M11/F10 | N | RC |  | Received equivalent paper-based materials and followed self-paced practice instructions. Participants could apply to transition to STRONG after training. | ≥60 min/day, 5×/week | 6weeks | PEP (Paper-based program): 60min/day × 5/week × 3.0 = 900 METs-min/week |  |
| 375 | Yixiu Wang，2024^415^ | Middle- and high-income | 16 | Lower limb | 9I + 7H | Chronic Phase | Moderate | 52.38±11.94 | M4/F12 | Y | ESX | CT/MRI unilateral stroke; Brunnstrom LE ≥III; independent standing ≥10s, walking ≥2min; excluded rTMS contraindications, visual/psychiatric comorbidity. | Combined real rTMS stimulation (10Hz, 2000 pulses) targeting the tibialis anterior with visual feedback cycling training; stimulation was synchronized with 15 minutes of active pedaling. | 20 min/session, 5×/week | 2weeks | rTMS + Cycling Group: Rehab: 30min × 5/week × 3.0 = 450 rTMS cycling: 15min × 5/week × 4.5 = 337.5 Total = 787.5 METs-min/week | BBS FMA-LE |
|  |  | Middle- and high-income | 16 | Lower limb | 6I + 10H | Chronic Phase | Moderate | 47.94±18.39 | M6/F10 | Y | MBE |  | Underwent the same cycling training with sham stimulation (inactive coil and simulated sound), identical in visual feedback parameters. | 20 min/session, 5×/week | 2weeks | Sham rTMS Group: Same rehab: 450 Sham cycling (same protocol): 337.5 Total = 787.5 METs-min/week |  |
| 376 | Stephen J Page，2008^416^ | High income | 4 | Lower limb | I | Chronic Phase | Moderate | 61.29±12.3 | M3/F1 | N | FT | Chronic I stroke (>12mo); LE strength ≥MRC 3, full ROM; 10m walking (aids allowed); excluded MAS >4, pain, oxygen need, major comorbidities. | Used NuStep TRS4000 recumbent cross-trainer for alternating upper and lower limb exercise in seated position. Sessions included warm-up, 30-minute main training, and cool-down. Resistance was progressively increased; heart rate, blood pressure, and RPE were monitored throughout. | 30 min/session, 3×/week | 8weeks | NuStep Phase: Recumbent resistance cycling: 30min × 3/week × 4.5 = 405 METs-min/week | BBS FMA-LE |
|  |  | High income | 3 | Lower limb | I | Chronic Phase | Moderate | 61.29±12.3 | M2/F1 | N | RC |  | Followed a standard home exercise program including ankle, knee, and hip movements, demonstrated once by a researcher. Participants recorded activity in a training log, with N progressive load adjustment. | 30 min/session, 3×/week | 8weeks | HEP Phase: Home stretching exercises: 30min × 3/week × 2.5 = 225 METs-min/week |  |
| 379 | Alex MartiN Cinnera，2024^417^ | High income | 11 | Trunk | I | Chronic Phase | Moderate | 56.45±15.56 | F6/M5 | N | VRG | CT/MRI-confirmed cortical/subcortical I stroke >180d; age 18–80; supervised standing; excluded neuro/musculoskeletal disease, MMSE<23. | Trained with the RIABLO™ system using IMUs and a force platform to provide audiovisual feedback during game-based tasks. Exercises targeted postural control and gait stability, such as weight shifts and phase transitions. | 30 min/session, 2–3×/week | 4weeks | Group 1 (avg MET 3.8): 30min × 2.5/week × 3.8 = 285 METs-min/week | BBS |
|  |  | High income | 10 | Trunk | I | Chronic Phase | Moderate | 58.11±13.16 | F5/M5 | N | BT |  | Performed traditional balance training involving similar movements but without feedback or sensor techNlogy. Some exercises used unstable surfaces under therapist supervision. | 30 min/session, 2–3×/week | 4weeks | Group 2 (avg MET 3.3): 30min × 2.5/week × 3.3 = 247.5 METs-min/week |  |
| 383 | da Silva, 2015^418^ | Middle- and high-income | 10 | Upper limb | Nt reported | Chronic Phase | Mild to moderate | 70.4±7.83 | M4/F6 | N | TOT | CT/MRI-confirmed unilateral stroke; excluded MAS >3, severe pain, shoulder contracture; MMSE ≥20; shoulder ROM ≥60°. | Performed task-oriented training based on daily activities (e.g., brushing hair, pouring, handling kitchenware), monitored by therapists with trunk fixation to prevent compensation. | 30 min/session, 2×/week | 6weeks | TOT Group: 30min × 2/week × 2.5 = 150 METs-min/week | FMA-UE |
|  |  | Middle- and high-income | 10 | Upper limb | Nt reported | Chronic Phase | Mild to moderate | 70.3±7.83 | M3/F7 | N | RT |  | Performed the same tasks as TOT but with added resistance (e.g., weighted wristbands, loaded utensils), tailored to 60% of shoulder flexor strength. | 30 min/session, 2×/week | 6weeks | TOT + Strength Group: 30min × 2/week × 3.5 = 210 METs-min/week |  |
| 384 | Katherine Hankinson，2022^419^ | High income | 10 | Whole-body function | Nt reported | Subacute Phase | Moderate to severe | ≥18 | M5/F5 | Y | MBE | Clinically diagNsed stroke; excluded severe joint pain, Nn-stroke mobility impairments, unstable medical/mental condition; cognitively intact. | Used the “GotRhythm” iOS app for rhythmic limb movement training with personalized BPM pacing and real-time feedback via IMU sensors. Tasks included warm-up, BPM setting, movement execution, and recovery if rhythm deviated. | 20 min/session, 3×/week | 6weeks | GotRhythm Group: Rehab: 30min/day × 6/week × 3.0 = 540 GotRhythm: 23min/week × 4.5 = ~104 Total = 540 + 104 = 644 METs-min/week | FMA-LE FMA-UE |
|  |  | High income | 12 | Whole-body function | Nt reported | Subacute Phase | Moderate to severe |  | M8/F4 | N | RC |  | Received standard rehabilitation delivered by their usual therapists without music-based components. | 20 min/session, 3×/week | 总6weeks | Control Group: Rehab only: 540 METs-min/week |  |
| 388 | Richard J. Adams，2023^420^ | High income | 9 | Upper limb | Nt reported | Chronic Phase | Moderate | 45–73 | M4/F5 | N | VRG | Stroke diagNsis; UE active motion: elbow flex ≥45°, shoulder flex/abd ≥30°, rot ≥15°; 1 finger flexion; visual ≥20/50; N neglect, MAS ≤2; follow commands. | Wore SaeboGlove to perform virtual IADL tasks using the SaeboVR system with motion capture and finger tracking. Training was asynchroNus and remote, with biweekly synchroNus OT video feedback. Tasks included reaching, grasping, and object manipulation, with adjustable difficulty. | 45 min/session (GRASP), 4×/week + biweekly remote OT | 8weeks | GRASP Virtual Rehab Group: 45min × 4/week × 4.0 = 720 METs-min/week (OT participation Nt included in main analysis) | FMA-UE |
|  |  | High income | 9 | Upper limb | Nt reported | Chronic Phase | Moderate | 34–86 | M6/F3 | N | NE |  | Received usual and customary therapy without additional training or devices. GRASP system was offered after the study for fairness. | 45 min/session (GRASP), 4×/week + biweekly remote OT | 8weeks | UCT Group (Control): N intervention (Estimated: 30min × 3/week × 3.0 = ~270 METs-min/week; excluded from analysis) |  |
| 389 | Susan M. Linder，2024^421^ | High income | 30 | Upper limb | 80%I + 20%H | Chronic Phase | Moderate | 60.8±11.5 | M18/F12 | N | FT | Single stroke, CPX tested; excluded recent cardiac event, spasticity, contracture, AE contraindications. | Performed forced aerobic cycling using a motor-assisted recumbent bike targeting 60–80% HRR, followed by repetitive upper limb task training involving grasping, transporting, and coordinated movement. | 90 min/session, 3×/week | 8weeks | FE + RTP Group: Forced cycling: 45min × 3/week × 4.5 = 607.5 Upper limb RTP: 45min × 3/week × 2.5 = 337.5 Total = 945 METs-min/week | 6MWT FMA-UE |
|  |  | High income | 30 | Upper limb | 76.7%I + 23.3%H | Chronic Phase | Moderate | 60.2±9.8 | M17/F13 | N | ULT |  | Completed the same structure of repetitive task training without aerobic exercise, with session time doubled to match total duration. | 90 min/session, 3×/week | 8weeks | RTP Only Group: RTP: 90min × 3/week × 2.5 = 675 METs-min/week |  |
| 391 | Gozde Iyigun Yatar，2015^422^ | Middle- and high-income | 15 | Trunk | I | Chronic Phase | Mild to moderate | 62.80±10.87 | M6/F9 | Y | VRG | Stroke confirmed; excluded epilepsy, MMSE ≤20, BDI ≥30, Rankin >3; MMSE for cognition, BDI for depression. | Each session included 30 minutes of Bobath-based neurodevelopmental therapy followed by 30 minutes of Wii Fit virtual reality training with three games (Soccer Heading, Ski Slalom, Balance Bubble), focusing on weight shift, coordination, and posture control. Each game was repeated three times with brief rest. | 60 min/session, 3×/week | 4weeks | Wii Fit Group: Wii Fit: 30min × 3/week × 3.5 = 315 NDT: 30min × 3/week × 3.0 = 270 Total = 585 METs-min/week | BBS |
|  |  | Middle- and high-income | 15 | Trunk | I | Chronic Phase | Moderate | 56.60±16.42 | M7/F8 | Y | BT |  | Received 30 minutes of the same neurodevelopmental training, then progressive balance training on the ground, including head-neck mobility, trunk rotation, reach, weight shifting, and single-leg stance. Tasks were adjusted based on functional ability. | 60 min/session, 3×/week | 4weeks | Wii Fit Group: Wii Fit: 30min × 3/week × 3.5 = 315 NDT: 30min × 3/week × 3.0 = 270 Total = 585 METs-min/week |  |
| 392 | Yiyeop Moon，2019^423^ | High income | 7 | Lower limb | 28.6%I + 71.4%H | Chronic Phase | Moderate | 59.1±10.0 | M6/F1 | Y | MBE | Clinically diagNsed stroke ≥12 months; able to walk >10 m independently; MMSE >24; N visual/hearing impairment, spatial neglect, or other neurological disorders. | Watched 10-minute video scenes of backward walking (straight, curved, unstable surfaces), then performed 20 minutes of ground-based backward walking mimicking the scenarios, under therapist guidance. Speed was self-paced. | 30 min/session (10 min observation + 20 min training), 3×/week | 4weeks | BWOT Group: Conventional rehab + action observation: 30min × 5/week + 30min × 3/week METs: 3.0 (rehab) + estimated 1.5–3.8 Total = 723 METs-min/week | 10MWT |
|  |  | High income | 7 | Lower limb | 57.1%I + 42.9%H | Chronic Phase | Moderate | 55.8±6.2 | M3/F4 | Y | RC |  | Watched 10-minute Nn-dynamic scenic videos without human movement, followed by the same walking tasks without action observation component. | 30 min/session (10 min observation + 20 min training), 3×/week | 4weeks | CG Group: Same as BWOT group Total = 723 METs-min/week |  |
| 393 | Caroline Lund，2017^424^ | High income | 17 | Lower limb | I | Chronic Phase | Moderate | 67.7±9.4 | M9/F4 | Y | AE | I stroke confirmed by physician; reduced lower limb strength on MMT; gait speed <1.4 m/s; N cognitive or neurological comorbidities. | Performed three 12-minute bouts of aerobic cycling on a Monark Ergomedic 828E ergometer. Target intensity was 75% heart rate reserve with RPE 14–16. Heart rate was continuously monitored. | ~45 min/session, 3×/week | 12weeks | AT Group (Aerobic Cycling): Rehab: 30min × 3 × 3.0 = 270 Cycling: 36min × 3 × 6.8 = 734 Total = 1004 METs-min/week | 6MWT |
|  |  | High income | 14 | Lower limb | I | Chronic Phase | Moderate | 67.3±7.4 | M11/F3 | Y | LLT |  | Engaged in five lower-limb resistance exercises (hip, knee, ankle, leg press), performed unilaterally. Exercises were done in three sets of eight reps at 80% of 1RM, with biweekly strength reassessment and load adjustment. | ~45 min/session, 3×/week | 12weeks | Resistance Training Group: Rehab: 270 Resistance: 45min × 3 × 6.0 = 810 Total = 1080 METs-min/week |  |
|  |  | High income | 17 | Upper limb | I | Chronic Phase | Moderate | 66.4±8.8 | M11/F5 | Y | ULT |  | Performed upper-limb resistance exercises (pull, push, shoulder movements) in three sets of 15 reps at 60% of 1RM. Exercises were guided by therapists and seated to limit trunk/lower limb involvement. | ~45 min/session, 3×/week | 12weeks | Upper Limb Control Group: Rehab: 270 UL training: 45min × 3 × 2.5 = 338 Total = 608 METs-min/week |  |
| 394 | Nancy Byl，2015^425^ | High income | 5 | Lower limb function | I | Chronic Phase | Mild | 66.2±5.0 | F3/M2 | Y | MBE | Physician-diagNsed stroke; FMA-LE ≥10; SLUMS >24; Beck Depression Inventory <12; CAFÉ FIM >50; able to walk 100 ft independently. | Completed 12 one-on-one gait training sessions, including dynamic balance, step coordination, trunk stability, strength, and dual-task walking. Participants wore smart shoes and joint sensors, receiving real-time gait feedback on an iPad. | 90 min/session, 2×/week | 12days | Upper Limb Control Group: Rehab: 270 UL training: 45min × 3 × 2.5 = 338 Total = 608 METs-min/week | 6MWT 10MWT BBS FMA-LE |
|  |  | High income | 7 | Lower limb function | I | Chronic Phase | Mild | 60.8±5.4 | F5/M2 | N | GT |  | Received identical gait training sessions without any feedback device. Training content was the same, focusing on treadmill and overground walking, obstacle navigation, strength, and flexibility. | 90 min/session, 2×/week | 12days | Control Group: Total: 60min × 2 × 3.5 = 420 METs-min/week |  |
| 396 | Zhishui Wu，2020^426^ | Middle- and high-income | 32 | Whole-body function | 66.7%I + 33.3%H | Acute Phase | Moderate | 56.73±11.85 | M19/F11 | Y | FT | Stroke diagNsed per 2014 Chinese Neurology Society criteria with CT/MRI; NIHSS score 5–15; motor impairment present. | Received standard inpatient rehabilitation, then joined a collaborative remote home program involving a multidisciplinary team. TCMeeting v6.0 was used for real-time online coaching by nurses and rehab engineers. Family caregivers assisted with task-oriented training using manuals and videos. | 5–7 min/session, 2×/week (remote) | 12weeks | Home-Based Rehab + Mixed Training: Remote training: 45min × 2 × 4.5 = 405 Rehab: 30min × 5 × 3.0 = 450 Total = 855 METs-min/week | 6MWT BBS FMA FMA-LE FMA-UE |
|  |  | Middle- and high-income | 32 | Whole-body function | 77.4%I + 22.6%H | Acute Phase | Moderate | 59.10±8.60 | M17/F14 | N | RC |  | Received the same inpatient rehabilitation but onlyweekly phone follow-ups post-discharge for general guidance. N video-based remote rehabilitation was provided. | 1×/week phone follow-up | 12weeks | Control Group: Rehab only: 450 METs-min/week |  |
| 397 | Yanan Zheng, 2020^427^ | Middle- and high-income | 30 | Trunk | 73%I + 27%H | Acute Phase | Severe | 63.50±10.36 | M24/F6 | Y | MBE | First stroke (method Nt specified); Brunnstrom upper limb stage ≥4; MMSE ≥24; sitting balance ≤ level 2; clinically stable. | Performed a modified version of “Liu Zi Jue” Qigong, combining six exhalation sounds (Xu, He, Hu, Si, Chui, Xi) with slow, coordinated movements. Breathing transitioned from thoracic to abdominal pattern. | 45 min/session (20 min main + 25 min conventional), 5×/week | 3weeks | Liuzijue Group: Liuzijue: 20min × 5 × 3.5 = 350 Rehab: 25min × 5 × 3.0 = 375 Total = 725 METs-min/week | BBS |
|  |  | Middle- and high-income | 30 | Trunk | 83%I + 17%H | Acute Phase | Severe | 67.23±9.15 | M19/F11 | Y | RC |  | Conducted traditional respiratory training in seated position, including prolonged exhalation, pursed-lip breathing, and manual rib compression during late exhalation by therapist. | 45 min/session (20 min main + 25 min conventional), 5×/week | 3weeks | Breathing Group: Breathing: 20min × 5 × 2.8 = 280 Rehab: 25min × 5 × 3.0 = 375 Total = 655 METs-min/week |  |
| 400 | Ji-Su Park, 2019^428^ | High income | 10 | Lower limb function | 50%I + 50%H | Subacute Phase | Mild to moderate | 70.13±7.56 | M6/F4 | Y | MBE | Unilateral stroke; MMSE >24; MIQ <3; ankle dorsiflexor strength >2; MAS <2; ambulatory but unstable; excluded pacemaker, epilepsy, skin disease. | Performed EMG-triggered electrical stimulation combined with motor imagery using Mentamove device. Each cycle included imagery, stimulation (triggered by imagined dorsiflexion), and relaxation. Standard physical and occupational therapy was provided alongside. | 30 min/session, 5×/week (total 4 weeks) | 4weeks | Breathing Group: Breathing: 20min × 5 × 2.8 = 280 Rehab: 25min × 5 × 3.0 = 375 Total = 655 METs-min/week | 10MWT FMA-LE |
|  |  | High income | 10 | Lower limb function | 40%I + 60%H | Subacute Phase | Mild to moderate | 65.86±13.10 | M5/F5 | Y | ESX |  | Performed identical motor imagery tasks without electrical stimulation feedback, with the same standard therapy. | 30 min/session, 5×/week (total 4 weeks) | 4weeks | MIT + EMG Group: Rehab: 450 MIT + EMG: 30min × 5 × 3.8 = 570 Total = 1020 METs-min/week |  |
| 401 | Ki Hun Cho, 2014^429^ | High income | 15 | Lower limb function | 67%I + 33%H | Chronic Phase | Moderate | 65.86±5.73 | M7/F8 | Y | MBE | Single stroke; MMSE-K >24; walking ability with functional impairment; excluded cardiac, hypertensive, orthopedic comorbidities or concurrent rehab trials. | Conducted treadmill training with real-environment video projection (e.g., outdoor scenes, obstacles, rain), combined with audio prompts to encourage forward gaze. Videos were changedweekly and sessions divided into three 10-minute modules. | 30 min/session, 3×/week (6 weeks) + 30 min PT + 30 min OT + 20 min FES daily | 6weeks | TBRVR Group (Treadmill + VR): VR treadmill: 30min × 3 × 4.5 = 405 Shared rehab (PT + OT + FES): 80min × 5 × 3.0 = 1200 Total = 1605 METs-min/week | BBS |
|  |  | High income | 15 | Lower limb function | 67%I + 33%H | Chronic Phase | Moderate | 63.53±5.54 | M8/F7 | Y | GT |  | Underwent conventional treadmill walking with identical parameters (speed, duration, harness), but without video or audio cues. | 30 min/session, 3×/week (6 weeks) + 30 min PT + 30 min OT + 20 min FES daily | 6weeks | Conventional Treadmill Group: Treadmill: 30min × 3 × 3.5 = 315 Shared rehab: 1200 Total = 1515 METs-min/week |  |
| 402 | Shuji Matsumoto, 2016^430^ | High income | 60 | Lower limb function | 19H + 41I | Subacute Phase | Subacute Phase | 62.4±10.7 | M42/F18 | Y | WA | Stroke (CT/MRI), neuro deficits; age 20–75; unilateral LL hemiparesis (Brunnstrom 3–6); ambulatory (aid); excl. <4 wk stroke, severe comorbidities, aphasia, cognitive/visual impair. | Performed 30-minute aquatic training in a therapy pool (chest-level, 30–31°C) including warm-up, endurance/strength exercises (walking, cross-step, resistance), and cool-down. Conventional rehabilitation was also provided. | 30 min/session, 2×/week (total 12 weeks) | 12weeks | Aquatic Exercise Group: Water training: 30min × 2 × 5.5 = 330 Rehab: 30min × 2/day × 6 days × 3.0 = 1080 Total = 1410 METs-min/week | 10MWT |
|  |  | High income | 60 | Lower limb function | 21H + 39I | Subacute Phase | Subacute Phase | 63.2±11.5 | M46/F14 | N | RC |  | Received the same duration and frequency of conventional rehabilitation, including ROM, strength, gait, ADL, and speech therapy. | 30 min/session, 2×/week (total 12 weeks) | 12weeks | Rehab: 30min × 2/day × 6 days × 3.0 = 1080METs-min/week |  |
| 405 | So Young Lee, 2018^431^ | High income | 18 | Lower limb function | 58%I + 42%H | Subacute Phase | Moderate | 57.58±13.98 | M9/F10 | N | WA | First unilateral I or H stroke; CT/MRI-confirmed; K-MMSE ≥10; deemed safe for exercise testing by rehab physician. | Received daily physical and occupational therapy plus 30-minute underwater treadmill training. Aquatic sessions included warm-up, main walking task (with speed and depth progression), and cool-down, monitored by therapists. Water temperature: 30–33°C. | 30 min aquatic + 60 min conventional, 5×/week | 4weeks | Underwater Treadmill Group: Aquatic treadmill: 30min × 5 × 5.0 = 750 Rehab: 60min × 5 × 3.0 = 900 Total = 1650 METs-min/week | BBS FMA FMA-LE |
|  |  | High income | 14 | Lower limb function | 50%I + 50%H | Subacute Phase | Moderate | 63.67±11.37 | M10/F8 | N | AE |  | Received daily physical and occupational therapy plus land-based upper and lower limb ergometer aerobic training. Initial intensity was 30–50% of max load, progressing to individual tolerance. N aquatic or simulation-based interventions. | 30 min aquatic + 60 min conventional, 5×/week | 4weeks | Land Ergometer Group: Cycling: 30min × 5 × 4.0 = 600 Rehab: 60min × 5 × 3.0 = 900 Total = 1500 METs-min/week |  |
| 406 | Emília Márcia Gomes de Souza e Silva，2017^432^ | Middle- and high-income | 19 | Lower limb function | 73.7%I + 26.3%H | Subacute Phase | Moderate | 52.0–63.0 | M13/F6 | Y | CIMT | Subacute stroke (~4.5 months); slow gait <0.8 m/s; independent 10 m walking without assistive device. | Conducted 30-minute treadmill walking with 5% body weight ankle load on the Nn-paretic side to promote paretic leg use. Also performed loaded weight-shifting tasks (3×10 reps) with verbal cueing at home. N other exercise training was provided. | 30 min training + home task, 5×/week | 2weeks | Treadmill + Light Load Group: Treadmill: 30min × 5 × 4.5 = 675 Home balance: 10min × 5 × 2.5 = 125 Total = 800 METs-min/week | BBS |
|  |  | Middle- and high-income | 19 | Lower limb function | 94.7%I + 5.3%H | Subacute Phase | Moderate | 47.0–66.0 | M10/F9 | Y | GT |  | Same treadmill walking without loading, intensity set to 50% HRmax with rest at 10 and 20 minutes. Same home training tasks were performed, without additional interventions. | 30 min training + home task, 5×/week | 2weeks | Treadmill Only Group: Treadmill: 30min × 5 × 4.0 = 600 Home: 125 Total = 725 METs-min/week |  |
| 407 | Patrícia P.B. Henrique，2019^433^ | Middle- and high-income | 16 | Upper limb and trunk | I | Chronic Phase | Mild to moderate | 76.19±10.09 | M7/F9 | N | VRG | CT-confirmed I stroke; age ≥55; MMSE meets education-adjusted threshold (≥19); N sensory deficits; excluded severe spasticity (MAS 3–4), aphasia, blindness. | Engaged in individualized Motion Rehab AVE 3D training using Kinect and projection, involving six contextual activities for upper and lower limb flexion/abduction, with real-time scoring and feedback. Vital signs were measured pre/post session. | 30 min/session, 2×/week | 12weeks | Experimental Group: 30min × 2 × 3.8 = 228 METs-min/week | BBS FMA-UE |
|  |  | Middle- and high-income | 15 | Upper limb and trunk | I | Chronic Phase | Mild to moderate | 76.20±10.41 | M7/F8 | N | RC |  | Performed therapist-guided conventional exercises matching the experimental group’s movements. Same rest intervals and total training time. N additional stroke rehabilitation provided. | 30 min/session, 2×/week | 12weeks | Control Group: 30min × 2 × 3.0 = 180 METs-min/week |  |
| 411 | Hyun-Joon Yoo, 2018^434^ | High income | 20 | Trunk | 11I + 9H | Acute Phase | Moderate | 34–82 | M14/F6 | Y | RT | First stroke (I or H); CT/MRI confirmed; NIHSS 5–36; basic command-following ability; excluded severe cardiopulmonary issues, aphasia, oral structural disorders, BP >180/100 mmHg. | Received standard rehabilitation and two daily 30-minute bedside respiratory muscle training sessions including breath stacking, inspiratory training (3-ball spirometer), and expiratory training (Acapella PEP device), supervised by a rehab physician. | 30 min/session ×2/day, 7×/week | 3weeks | Experimental Group (Respiratory Muscle + Conventional Rehab): Conventional rehab: 30min × 2/day × 5 days × 3.0 METs = 900 Respiratory training (Breath stacking + inspiratory + expiratory): 30min × 2/day × 7 days × 2.5 METs = 1050 Total = 1950 METs-min/week | BBS FMA |
|  |  | High income | 20 | Trunk | 12I + 8H | Acute Phase | Moderate | 34–86 | M12/F8 | N | RC |  | Received the same standard rehabilitation but without respiratory muscle training. | 30 min/session ×2/day, 7×/week | 3weeks | Control Group: Same rehab (without respiratory training): 900 METs-min/week |  |
| 412 | Wan-Yun Huang，2021^435^ | Middle- and high-income | 12 | Lower limb function | 8I + 4H | Chronic Phase | Moderate | 53.67±9.16 | M9/F3 | Y | MBE | CT/MRI-confirmed unilateral stroke; MMSE ≥24; ambulatory ≥15 m; N severe orthopedic or systemic diseases. | Received standard rehabilitation with additional 15-minute lateral stair training using a dynamic staircase. Tasks included stepping up/down varying heights (0–16.5 cm) with the paretic leg leading ascent and Nn-paretic leg for descent. | 30 min/session (15 min lateral + 15 min conventional), 1×/week | 12weeks | Experimental Group (Lateral Stair Walking): Traditional PT: 15min × 1/week × 3.0 METs = 45 Stair training: 15min × 1/week × 4.5 METs = 67.5 Total = 112.5 METs-min/week | FMA-LE |
|  |  | Middle- and high-income | 12 | Lower limb function | 8I + 4H | Chronic Phase | Moderate | 63.33±13.31 | M9/F3 | Y | ULT |  | Received the same standard rehabilitation without any stair training. | 30 min/session conventional, 1×/week | 12weeks | Control Group: Traditional PT only: 30min × 1/week × 3.0 = 90 METs-min/week |  |
| 413 | Letícia Cardoso Rodrigues，2016^436^ | Middle- and high-income | 8 | Upper limb function | I | Chronic Phase | Moderate | 58.4±8.3 | M4/F4 | N | MBE | Unilateral I stroke ≥6 months; CT/MRI-confirmed; FMA-UE 30–49; MAS ≤2; excluded neurological comorbidities, severe shoulder pain, neglect, aphasia, ongoing upper limb rehab. | Conducted home-based mirror therapy under therapist guidance. Tasks involved symmetrical bilateral upper limb coordination using common objects (e.g., bottles, balls) with visual feedback from a mirror box. Task difficulty was adjusted based on progress. | 60 min/session, 3×/week (total 4 weeks) | 4weeks | Mirror Therapy Group: Mirror therapy + bilateral symmetric tasks: 60min × 3/week × 3.0 = 540 METs-min/week | FMA-UE |
|  |  | Middle- and high-income | 8 | Upper limb function | I | Chronic Phase | Moderate | 56.6±5.3 | M6/F2 | N | ULT |  | Performed identical bilateral tasks with the mirror covered, directly observing the affected limb. Therapist feedback and progression matched the experimental group. | 60 min/session, 3×/week (total 4 weeks) | 4weeks | Control Group (N mirror): Bilateral tasks (same intensity): 540 METs-min/week |  |
| 414 | Donmo Choi，2018^437^ | High income | 14 | Trunk | 10I + 4H | Chronic Phase | Mild | 49.5 | M9/F5 | Y | VRG | First stroke (I or H); CT/MRI-confirmed; MMSE >24; MVPT-3 <45; 30-min standing tolerance; excluded severe visual impairments, vestibular disorders, medication affecting balance. | Received conventional rehabilitation plus Wii Fit-based virtual reality training with six progressive balance games (e.g., Tightrope Walk, Soccer Heading, Penguin Slide, Ski Slalom, Table Tilt), focusing on multi-directional weight shifts with visual and auditory feedback. | 90 min/session conventional daily + 30 min Wii, 3×/week | 6weeks | Wii VR Training Group (WVRT): Wii Fit games: 30min × 3/week × 3.8 = 342 Conventional rehab: 90min × 5/week × 3.0 = 1350 Total = 1692 METs-min/week | 10MWT BBS |
|  |  | High income | 14 | Trunk | 8I + 6H | Chronic Phase | Mild | 51 | M8/F6 | Y | MBE |  | Received conventional rehabilitation and performed platform-based balance training using mirrored feedback, including forward/backward and lateral shifts with increasing difficulty (e.g., balancing with a plate on the head). | 90 min/session conventional daily + 30 min balance, 3×/week | 6weeks | Group Balance Training (GBT): Balance board + mirror: 30min × 3/week × 3.3 = 297 Same rehab: 1350 Total = 1647 METs-min/week |  |
| 415 | Osman Karaca，2024^438^ | Middle- and high-income | 11 | Trunk | 82%I + 18%H | Chronic Phase | Moderate | 30–78 | M6/F5 | Y | ESX | Unilateral stroke (CT/MRI); MMSE ≥23; sit/walk (± aid); excl. severe cognitive, communication, neuro-ortho issues; age ≤85; TIS ≥20. | Conducted combined sensory training and trunk Bobath therapy. Sensory tasks included mirror lateral shifts, TENS to latissimus dorsi, trunk repositioning, and tactile stimulation. Bobath components were customized (e.g., scapular movement, core activation, trunk stability). Sessions also included standard rehabilitation. | 70 min/session (40 min main + 30 min conventional), 3×/week | 8weeks | Experimental Group: Bobath: 25min × 3 × 3.5 = 262.5 Perception training: 15min × 3 × 2.5 = 112.5 Conventional rehab: 30min × 5 × 3.0 = 450 Total = 825 METs-min/week | BBS |
|  |  | Middle- and high-income | 12 | Trunk | 92%I + 8%H | Chronic Phase | Moderate | 46–80 | M10/F2 | N | NPF |  | Performed the same Bobath-based trunk training without sensory stimulation. Training content and structure matched the experimental group. Standard rehabilitation was also provided. | 70 min/session (40 min main + 30 min conventional), 3×/week | 8weeks | Control Group: Bobath only: 40min × 3 × 3.5 = 420 Same rehab: 450 Total = 870 METs-min/week |  |
| 417 | Hsieh-Chun Hsieh，2018^439^ | Middle- and high-income | 28 | Lower limb function | I | Chronic Phase | Moderate | 63.5±12.5 | M17/F11 | Y | VRG | Clinically diagNsed I stroke; ankle dorsiflexion >10°; ambulatory with or without assistive device; excluded severe gait deficits or medical conditions. | Used adaptive foot switches to control video games via ankle dorsiflexion/plantarflexion. Training includedweekly clinic visits and daily home practice. | 30 min/session clinical 1×/week + daily home (duration unclear) | 10weeks | Video Game Foot Training Group (VG): Conventional rehab: 30min × 1/week × 3.0 = 90 VG foot training: 30min × 8/week × 4.5 = 1080 Total = 1170 METs-min/week | 10MWT |
|  |  | Middle- and high-income | 28 | Lower limb function | I | Chronic Phase | Moderate | 63.5±12.5 | M16/F12 | Y | GT |  | Conducted standard gait training without adaptive devices. | 1×/week conventional + daily walking | 10weeks | Control Group: Conventional rehab: 90 Walking: 30min × 7 × 3.0 = 630 Total = 720 METs-min/week |  |
| 418 | Maira Jaqueline da Cunha, 2023^440^ | Middle- and high-income | 16 | Lower limb function | 68.7%I + 31.3%H | Chronic Phase | Moderate to severe | 55.44±8.47 | M11/F5 | Y | ESX | CT/MRI-confirmed stroke ≥6 months; FMA-LE used for motor evaluation; MMSE used for cognitive screening. | Performed gait training while wearing foot-drop stimulators (FDs) combined with active bilateral tDCS stimulation. Sessions included 5 min seated tDCS, 20 min tDCS+FDs during walking, and 5 min post-training tDCS. | 30 min/session, 5×/week | 2weeks | Experimental Group (tDCS + FDS Gait): Rehab: 15min × 5 × 3.0 = 225 Gait with WalkAide + tDCS: 20min × 5 × 4.5 = 450 Total = 675 METs-min/week | FMA-LE |
|  |  | Middle- and high-income | 16 | Lower limb function | 81.2%I + 18.8%H | Chronic Phase | Moderate to severe | 58.25±9.75 | M11/F5 | Y | GT |  | Received identical procedures, but with sham tDCS (active only for initial 30 seconds). | 30 min/session, 5×/week | 2weeks | Control Group (Sham tDCS): Same structure and values Total = 675 METs-min/week |  |
| 419 | Sergen Öztürk, 2024^441^ | Middle- and high-income | 13 | Trunk and lower limb function | I | Chronic Phase | Moderate | 54.46±14.50 | M8/F5 | Y | FT | Clinically diagNsed by neurologist with imaging (type Nt specified); consistent with medical standards. | Completed conventional rehabilitation and trained on the TA-022 horseback riding simulator, mimicking forward, backward, and lateral saddle motion. Support was gradually removed as stability improved. | 60 min/session, 5×/week | 6weeks | Horse Simulator Group (HSG): Horse simulator: 15min × 5 × 3.5 = 262.5 Rehab: 45min × 5 × 3.0 = 675 Total = 937.5 METs-min/week | BBS |
|  |  | Middle- and high-income | 13 | Trunk and lower limb function | I | Chronic Phase | Moderate | 57.23±8.09 | M10/F3 | N | RC |  | Performed conventional rehab including bed mobility, sitting balance, weight-bearing, and obstacle/ladder gait training, under therapist supervision. | 60 min/session, 5×/week | 6weeks | Conventional Rehab Group (CEG): Rehab only: 60min × 5 × 3.0 = 900 METs-min/week |  |
| 420 | Egger, 2024^442^ | High income | 19 | Upper limb function | Mainly I | Chronic Phase | Moderate | 65.2±10.2 | M9/F6 | N | ESX | Physician-diagNsed unilateral stroke; SULCS 1–6 (moderate-to-severe upper limb paresis); excluded epilepsy, severe aphasia, cognitive deficits, inability to sit 45 min. | Wore the tipstim® glove for somatosensory stimulation (20 Hz, 2s on/5s off), followed immediately by 45-minute upper limb robot-assisted training using ArmeoPower, Spring, or Amadeo devices. | 90 min/session (RSS + robot), 4×/week | 3weeks | RSS Group: Repetitive sensory stimulation (RSS): 30min × 5 × 1.5 = 225 Robotic UL training: 45min × 5 × 2.5 = 562.5 Total = 787.5 METs-min/week | FMA-UE |
|  |  | High income | 21 | Upper limb function | Mainly I | Chronic Phase | Moderate | 63.6±9.4 | M9/F6 | N | RAT |  | Used identical-looking gloves with inactive circuits (sham stimulation), followed by the same robotic training protocol. | 90 min/session (RSS + robot), 4×/week | 3weeks | Control Group (Sham RSS): Sham RSS: 30min × 5 × 1.0 = 150 Same robotic training = 562.5 Total = 712.5 METs-min/week |  |

**Abbreviations:** Y, yes; N, no; I, ischemic stroke; H, hemorrhagic stroke; RC, routine care; NE, no exercise; NPF, neuro/proprioceptive facilitation; ULT, upper limb training; LLT, lower limb training; CST, core stability training; BT, balance training; WA, water-based activity; VT, vibration training; ESX, electrical stimulation plus exercise; RAT, robotic-assisted training; VRG, virtual reality/gaming; AE, aerobic exercise; HIIT, high-intensity interval training; GT, gait training; FT, functional training; RT, resistance training; MBE, mind–body exercise; TCM+EX, traditional Chinese medicine combined with exercise; TAE, technology-assisted exercise; TOT, task-oriented training; CIMT, constraint-induced movement therapy; METs, metabolic equivalent of task; BBS, Berg Balance Scale; 10MWT, 10-Meter Walk Test; 6MWT, 6-Minute Walk Test; FMA, Fugl–Meyer Assessment; FMA-UE, Fugl–Meyer Assessment for Upper Extremity; FMA-LE, Fugl–Meyer Assessment for Lower Extremi

**Supplementary 6 — Risk of Bias Assessment (RoB 2.0)**

**Table S6 Risk of bias assessment**

| study | Randomization process | Deviations from intended interventions | Mising outcome data | Measurement of the outcome | Selection of the reported result | Overall |
| --- | --- | --- | --- | --- | --- | --- |
| 1 | Low risk | Some concerns | High risk | Low risk | Low risk | High risk |
| 2 | Low risk | Some concerns | Low risk | Low risk | Some concerns | Some concerns |
| 3 | Low risk | Some concerns | Some concerns | Low risk | Low risk | Some concerns |
| 4 | Low risk | High risk | High risk | High risk | Low risk | High risk |
| 5 | Low risk | Some concerns | Low risk | Low risk | Low risk | Some concerns |
| 6 | Low risk | Some concerns | Low risk | Low risk | Low risk | Some concerns |
| 8 | Low risk | Some concerns | Some concerns | Low risk | Low risk | Some concerns |
| 9 | Low risk | Some concerns | Some concerns | Low risk | Low risk | Some concerns |
| 10 | Low risk | Some concerns | Low risk | Low risk | Low risk | Some concerns |
| 11 | Low risk | Some concerns | Low risk | Low risk | Low risk | Some concerns |
| 12 | Low risk | Some concerns | Low risk | Low risk | Low risk | Some concerns |
| 13 | Low risk | Some concerns | Low risk | Low risk | Low risk | Some concerns |
| 14 | Low risk | Some concerns | Low risk | Low risk | Low risk | Some concerns |
| 16 | Low risk | Some concerns | Low risk | Low risk | Low risk | Some concerns |
| 17 | Low risk | Some concerns | Low risk | Low risk | Low risk | Some concerns |
| 18 | Low risk | Low risk | Low risk | Low risk | Low risk | Low risk |
| 19 | Low risk | Some concerns | Low risk | Low risk | Low risk | Some concerns |
| 20 | Low risk | Some concerns | Low risk | Low risk | Low risk | Some concerns |
| 21 | Low risk | Some concerns | Low risk | Low risk | Low risk | Some concerns |
| 22 | Low risk | Some concerns | Low risk | Low risk | Low risk | Some concerns |
| 24 | Low risk | Some concerns | Low risk | Some concerns | Low risk | Some concerns |
| 25 | Low risk | Some concerns | Low risk | Low risk | Low risk | Some concerns |
| 26 | Low risk | Some concerns | Low risk | Low risk | Low risk | Some concerns |
| 30 | Low risk | Low risk | Low risk | Low risk | Low risk | Low risk |
| 31 | Low risk | Some concerns | Low risk | Low risk | Low risk | Some concerns |
| 32 | Low risk | Some concerns | Low risk | Low risk | Low risk | Some concerns |
| 35 | Low risk | Some concerns | Low risk | Low risk | Low risk | Some concerns |
| 37 | Low risk | Some concerns | Low risk | Low risk | Low risk | Some concerns |
| 38 | Low risk | Some concerns | Low risk | Low risk | Low risk | Some concerns |
| 40 | Low risk | Some concerns | Low risk | Low risk | Low risk | Some concerns |
| 42 | Low risk | Some concerns | Low risk | Low risk | Low risk | Some concerns |
| 43 | Low risk | Some concerns | Low risk | Low risk | Low risk | Some concerns |
| 44 | Low risk | Some concerns | Low risk | Low risk | Low risk | Some concerns |
| 45 | Low risk | Low risk | Low risk | Low risk | Low risk | Low risk |
| 46 | Low risk | Some concerns | Low risk | Low risk | Low risk | Some concerns |
| 49 | Low risk | Some concerns | Low risk | Some concerns | Low risk | Some concerns |
| 50 | Low risk | Some concerns | Low risk | Some concerns | Low risk | Some concerns |
| 53 | Low risk | Some concerns | High risk | Low risk | Low risk | High risk |
| 54 | Low risk | Some concerns | Low risk | Some concerns | Low risk | Some concerns |
| 57 | Low risk | Some concerns | Low risk | Some concerns | Low risk | Some concerns |
| 59 | Low risk | Some concerns | Some concerns | Some concerns | Low risk | Some concerns |
| 62 | Low risk | Low risk | Low risk | Low risk | Low risk | Low risk |
| 64 | Low risk | Some concerns | Some concerns | Some concerns | Low risk | Some concerns |
| 65 | Low risk | Some concerns | Some concerns | Some concerns | Low risk | Some concerns |
| 66 | Low risk | Low risk | Low risk | Low risk | Low risk | Low risk |
| 67 | Low risk | Some concerns | Low risk | Low risk | Low risk | Some concerns |
| 68 | Low risk | Some concerns | Low risk | Low risk | Low risk | Some concerns |
| 69 | Low risk | Some concerns | Low risk | Low risk | Low risk | Some concerns |
| 71 | Low risk | Some concerns | Low risk | Low risk | Low risk | Some concerns |
| 73 | Low risk | Some concerns | Low risk | Low risk | Low risk | Some concerns |
| 74 | Low risk | Some concerns | Low risk | Some concerns | Low risk | Some concerns |
| 75 | Low risk | Some concerns | Low risk | Some concerns | Low risk | Some concerns |
| 76 | Low risk | Some concerns | Low risk | Some concerns | Low risk | Some concerns |
| 77 | Low risk | Some concerns | Low risk | Some concerns | Low risk | Some concerns |
| 80 | Low risk | Some concerns | Low risk | Low risk | Low risk | Some concerns |
| 81 | Low risk | Some concerns | Low risk | Low risk | Low risk | Some concerns |
| 82 | Low risk | Some concerns | Low risk | Low risk | Low risk | Some concerns |
| 83 | Low risk | Some concerns | Low risk | Low risk | Low risk | Some concerns |
| 85 | Low risk | Some concerns | Low risk | Some concerns | Low risk | Some concerns |
| 86 | Low risk | Some concerns | Low risk | Low risk | Low risk | Some concerns |
| 87 | Low risk | Some concerns | Low risk | Low risk | Low risk | Some concerns |
| 88 | Low risk | High risk | Low risk | Low risk | Low risk | High risk |
| 90 | Low risk | Some concerns | Low risk | Low risk | Low risk | Some concerns |
| 91 | Low risk | Some concerns | Low risk | Low risk | Low risk | Some concerns |
| 93 | Low risk | Some concerns | Low risk | Low risk | Low risk | Some concerns |
| 94 | Low risk | Some concerns | Low risk | Low risk | Low risk | Some concerns |
| 95 | Low risk | Some concerns | Low risk | Low risk | Low risk | Some concerns |
| 96 | Low risk | Some concerns | Low risk | Low risk | Low risk | Some concerns |
| 97 | Low risk | Some concerns | Low risk | Low risk | Low risk | Some concerns |
| 101 | Low risk | Some concerns | Low risk | Low risk | Low risk | Some concerns |
| 103 | Low risk | Some concerns | Low risk | Low risk | Low risk | Some concerns |
| 104 | Low risk | Some concerns | Low risk | Low risk | Low risk | Some concerns |
| 106 | Low risk | Some concerns | Low risk | Low risk | Low risk | Some concerns |
| 107 | Some concerns | High risk | Low risk | Low risk | Low risk | High risk |
| 108 | Low risk | Some concerns | Low risk | Low risk | Low risk | Some concerns |
| 109 | Low risk | Some concerns | Low risk | Low risk | Low risk | Some concerns |
| 110 | Low risk | Some concerns | Low risk | Low risk | Low risk | Some concerns |
| 112 | Low risk | Low risk | Low risk | Low risk | Low risk | Low risk |
| 113 | Low risk | Some concerns | Low risk | Low risk | Low risk | Some concerns |
| 114 | Some concerns | Some concerns | Low risk | Low risk | Low risk | Some concerns |
| 115 | Some concerns | Some concerns | Low risk | Low risk | Low risk | Some concerns |
| 116 | Low risk | Some concerns | Low risk | Some concerns | Low risk | Some concerns |
| 117 | Low risk | Low risk | Low risk | Low risk | Low risk | Low risk |
| 118 | Low risk | Some concerns | Low risk | Low risk | Low risk | Some concerns |
| 119 | Low risk | Some concerns | Low risk | Low risk | Low risk | Some concerns |
| 120 | Low risk | Some concerns | Low risk | Low risk | Low risk | Some concerns |
| 121 | Some concerns | Some concerns | Low risk | Low risk | Low risk | Some concerns |
| 123 | Low risk | Some concerns | Low risk | Low risk | Low risk | Some concerns |
| 126 | Low risk | Low risk | Low risk | Low risk | Low risk | Low risk |
| 127 | Some concerns | Low risk | Some concerns | Low risk | Low risk | Some concerns |
| 128 | Low risk | Low risk | Low risk | Low risk | Low risk | Low risk |
| 129 | Low risk | Low risk | Some concerns | Low risk | Low risk | Some concerns |
| 130 | Low risk | Low risk | Low risk | Low risk | Low risk | Low risk |
| 131 | Low risk | Some concerns | Low risk | Low risk | Low risk | Some concerns |
| 133 | Low risk | Some concerns | Some concerns | Low risk | Low risk | Some concerns |
| 134 | Low risk | Some concerns | Some concerns | Low risk | Low risk | Some concerns |
| 135 | Low risk | Some concerns | Low risk | Low risk | Low risk | Some concerns |
| 136 | Low risk | Some concerns | Low risk | Low risk | Low risk | Some concerns |
| 138 | Low risk | Some concerns | Low risk | Low risk | Low risk | Some concerns |
| 139 | Low risk | Some concerns | Low risk | Low risk | Low risk | Some concerns |
| 140 | Low risk | Some concerns | Low risk | Low risk | Low risk | Some concerns |
| 141 | Low risk | Some concerns | Low risk | Low risk | Low risk | Some concerns |
| 142 | Low risk | Some concerns | Low risk | Low risk | Low risk | Some concerns |
| 143 | Low risk | Some concerns | Low risk | Low risk | Low risk | Some concerns |
| 144 | Low risk | Some concerns | Low risk | Low risk | Low risk | Some concerns |
| 145 | Low risk | Some concerns | Low risk | Low risk | Low risk | Some concerns |
| 146 | Low risk | Some concerns | Low risk | Low risk | Low risk | Some concerns |
| 147 | Low risk | Some concerns | Low risk | High risk | Low risk | High risk |
| 148 | Low risk | Some concerns | Low risk | Low risk | Low risk | Some concerns |
| 149 | Low risk | Some concerns | Low risk | Low risk | Low risk | Some concerns |
| 150 | Low risk | Some concerns | Low risk | Low risk | Low risk | Some concerns |
| 151 | Low risk | Some concerns | Low risk | High risk | Low risk | High risk |
| 152 | Low risk | Some concerns | Low risk | Low risk | Low risk | Some concerns |
| 154 | Low risk | Some concerns | Low risk | Low risk | Low risk | Some concerns |
| 155 | Low risk | Some concerns | Low risk | Low risk | Low risk | Some concerns |
| 156 | Low risk | Some concerns | Low risk | Low risk | Low risk | Some concerns |
| 157 | Low risk | Some concerns | Low risk | High risk | Low risk | High risk |
| 158 | Low risk | Some concerns | Low risk | Low risk | Low risk | Some concerns |
| 159 | Low risk | Some concerns | Low risk | Low risk | Low risk | Some concerns |
| 160 | Low risk | Some concerns | Low risk | Low risk | Low risk | Some concerns |
| 161 | Low risk | Some concerns | Low risk | Low risk | Low risk | Some concerns |
| 162 | Low risk | Some concerns | Low risk | Low risk | Low risk | Some concerns |
| 164 | Low risk | Some concerns | Low risk | Low risk | Low risk | Some concerns |
| 165 | Low risk | Some concerns | Low risk | Low risk | Low risk | Some concerns |
| 167 | Low risk | Some concerns | Low risk | Low risk | Low risk | Some concerns |
| 169 | Low risk | Some concerns | Low risk | High risk | Low risk | High risk |
| 170 | Low risk | Some concerns | Low risk | Low risk | Low risk | Some concerns |
| 171 | Low risk | Some concerns | Low risk | Low risk | Low risk | Some concerns |
| 172 | Low risk | Some concerns | Low risk | Low risk | Low risk | Some concerns |
| 173 | Low risk | Some concerns | Low risk | Low risk | Low risk | Some concerns |
| 175 | Low risk | Some concerns | Low risk | Low risk | Low risk | Some concerns |
| 176 | Low risk | Some concerns | Low risk | Low risk | Low risk | Some concerns |
| 177 | Low risk | Some concerns | Low risk | Low risk | Low risk | Some concerns |
| 178 | Low risk | Some concerns | Low risk | Low risk | Low risk | Some concerns |
| 179 | Low risk | Low risk | Low risk | Low risk | Low risk | Low risk |
| 180 | Low risk | Some concerns | Low risk | Low risk | Low risk | Some concerns |
| 181 | Low risk | Some concerns | Low risk | Low risk | Low risk | Some concerns |
| 182 | Low risk | Some concerns | Low risk | Low risk | Low risk | Some concerns |
| 183 | Low risk | Some concerns | Low risk | Low risk | Low risk | Some concerns |
| 185 | Low risk | Some concerns | Low risk | Low risk | Low risk | Some concerns |
| 186 | Low risk | Some concerns | Low risk | Low risk | Low risk | Some concerns |
| 187 | Low risk | Some concerns | Low risk | Low risk | Low risk | Some concerns |
| 188 | Low risk | Some concerns | Low risk | Low risk | Low risk | Some concerns |
| 189 | Low risk | Some concerns | Low risk | Low risk | Low risk | Some concerns |
| 190 | Low risk | Some concerns | Low risk | Low risk | Low risk | Some concerns |
| 191 | Low risk | Some concerns | Low risk | Low risk | Low risk | Some concerns |
| 192 | Low risk | Some concerns | Low risk | Low risk | Low risk | Some concerns |
| 193 | Low risk | Some concerns | Low risk | Low risk | Low risk | Some concerns |
| 196 | Low risk | Some concerns | Low risk | Low risk | Low risk | Some concerns |
| 197 | Low risk | Low risk | Low risk | Low risk | Low risk | Low risk |
| 198 | Low risk | Some concerns | Low risk | Low risk | Low risk | Some concerns |
| 199 | Low risk | Some concerns | Low risk | Low risk | Low risk | Some concerns |
| 202 | Low risk | Some concerns | Low risk | Low risk | Low risk | Some concerns |
| 203 | Low risk | Some concerns | Low risk | High risk | Low risk | High risk |
| 204 | Low risk | Low risk | Low risk | Low risk | Low risk | Low risk |
| 205 | Low risk | Low risk | Low risk | Low risk | Low risk | Low risk |
| 206 | Low risk | Some concerns | Low risk | Low risk | Low risk | Some concerns |
| 207 | Low risk | Low risk | Low risk | Low risk | Low risk | Low risk |
| 209 | Low risk | Some concerns | Low risk | High risk | Low risk | High risk |
| 210 | Low risk | Some concerns | Low risk | Low risk | Low risk | Some concerns |
| 211 | Low risk | Some concerns | Low risk | Low risk | Low risk | Some concerns |
| 212 | Low risk | Some concerns | Low risk | Low risk | Low risk | Some concerns |
| 215 | Low risk | Some concerns | Low risk | Low risk | Low risk | Some concerns |
| 216 | Low risk | Some concerns | Low risk | Low risk | Low risk | Some concerns |
| 217 | Low risk | Some concerns | Low risk | Low risk | Low risk | Some concerns |
| 219 | Low risk | Some concerns | Low risk | Low risk | Low risk | Some concerns |
| 220 | Low risk | Low risk | Low risk | Low risk | Low risk | Low risk |
| 221 | Low risk | Low risk | Low risk | Low risk | Low risk | Low risk |
| 225 | Low risk | Low risk | Low risk | Low risk | Low risk | Low risk |
| 226 | Low risk | Some concerns | Low risk | Low risk | Low risk | Some concerns |
| 227 | Low risk | Some concerns | Low risk | Low risk | Low risk | Some concerns |
| 228 | Low risk | Some concerns | Low risk | Low risk | Low risk | Some concerns |
| 229 | Low risk | High risk | Low risk | Low risk | Low risk | High risk |
| 230 | Low risk | Some concerns | Low risk | Low risk | Low risk | Some concerns |
| 231 | Low risk | Some concerns | Low risk | Low risk | Low risk | Some concerns |
| 232 | Low risk | Some concerns | Low risk | Low risk | Low risk | Some concerns |
| 233 | Low risk | Some concerns | Low risk | Low risk | Low risk | Some concerns |
| 234 | Low risk | Some concerns | Low risk | Low risk | Low risk | Some concerns |
| 235 | Low risk | Some concerns | Low risk | High risk | Low risk | High risk |
| 236 | Low risk | Some concerns | Low risk | High risk | Low risk | High risk |
| 237 | Low risk | Some concerns | Low risk | Low risk | Low risk | Some concerns |
| 238 | Low risk | Some concerns | Low risk | Low risk | Low risk | Some concerns |
| 239 | Low risk | Some concerns | Low risk | Low risk | Low risk | Some concerns |
| 241 | Low risk | Some concerns | Low risk | Low risk | Low risk | Some concerns |
| 243 | Low risk | Some concerns | Low risk | Low risk | Low risk | Some concerns |
| 246 | Low risk | Some concerns | Low risk | Low risk | Low risk | Some concerns |
| 247 | Low risk | Some concerns | Low risk | Low risk | Low risk | Some concerns |
| 248 | Low risk | Some concerns | Low risk | Low risk | Low risk | Some concerns |
| 249 | Low risk | Some concerns | Low risk | Low risk | Low risk | Some concerns |
| 250 | Low risk | Some concerns | Low risk | Low risk | Low risk | Some concerns |
| 251 | Low risk | Some concerns | Low risk | Low risk | Low risk | Some concerns |
| 252 | Low risk | Some concerns | Low risk | Low risk | Low risk | Some concerns |
| 253 | Low risk | Some concerns | Low risk | Low risk | Low risk | Some concerns |
| 254 | Low risk | Some concerns | Low risk | Low risk | Low risk | Some concerns |
| 255 | Low risk | Some concerns | Low risk | Low risk | Low risk | Some concerns |
| 256 | Low risk | Some concerns | Low risk | Low risk | Low risk | Some concerns |
| 257 | Low risk | Some concerns | Low risk | Low risk | Low risk | Some concerns |
| 258 | Low risk | Some concerns | Low risk | Low risk | Low risk | Some concerns |
| 259 | Low risk | Some concerns | Low risk | Low risk | Low risk | Some concerns |
| 261 | Low risk | Some concerns | Low risk | Low risk | Low risk | Some concerns |
| 263 | Low risk | Some concerns | Low risk | Low risk | Low risk | Some concerns |
| 265 | Low risk | High risk | Low risk | Low risk | Low risk | High risk |
| 266 | Low risk | Some concerns | Low risk | Low risk | Low risk | Some concerns |
| 267 | Low risk | Some concerns | Low risk | Low risk | Low risk | Some concerns |
| 268 | Low risk | Some concerns | Low risk | High risk | Low risk | High risk |
| 269 | Low risk | Some concerns | Low risk | Low risk | Low risk | Some concerns |
| 270 | Low risk | Some concerns | Low risk | Low risk | Low risk | Some concerns |
| 271 | Low risk | Some concerns | Low risk | Low risk | Low risk | Some concerns |
| 272 | Low risk | Some concerns | Low risk | Low risk | Low risk | Some concerns |
| 274 | Low risk | Some concerns | Low risk | Low risk | Low risk | Some concerns |
| 275 | Low risk | Some concerns | Low risk | Low risk | Low risk | Some concerns |
| 277 | Low risk | Some concerns | Low risk | Low risk | Low risk | Some concerns |
| 278 | Low risk | Some concerns | Low risk | Low risk | Low risk | Some concerns |
| 279 | Low risk | Some concerns | Low risk | Low risk | Low risk | Some concerns |
| 280 | Low risk | Some concerns | Low risk | Low risk | Low risk | Some concerns |
| 281 | Low risk | Some concerns | Low risk | Low risk | Low risk | Some concerns |
| 282 | Low risk | Some concerns | Low risk | Low risk | Low risk | Some concerns |
| 285 | Low risk | Some concerns | Low risk | Low risk | Low risk | Some concerns |
| 286 | Low risk | Some concerns | Low risk | Low risk | Low risk | Some concerns |
| 287 | Low risk | Some concerns | Low risk | Low risk | Low risk | Some concerns |
| 288 | Low risk | Some concerns | Low risk | Low risk | Low risk | Some concerns |
| 289 | Low risk | Low risk | Low risk | Low risk | Low risk | Low risk |
| 291 | Low risk | Some concerns | Low risk | Low risk | Low risk | Some concerns |
| 292 | Low risk | Some concerns | Low risk | Low risk | Low risk | Some concerns |
| 293 | Low risk | Some concerns | Low risk | Low risk | Low risk | Some concerns |
| 295 | Low risk | Some concerns | Low risk | Low risk | Low risk | Some concerns |
| 296 | Low risk | Some concerns | Low risk | Low risk | Low risk | Some concerns |
| 297 | Low risk | Some concerns | Low risk | Low risk | Low risk | Some concerns |
| 298 | Low risk | Some concerns | Low risk | Low risk | Low risk | Some concerns |
| 299 | Low risk | Some concerns | Low risk | Low risk | Low risk | Some concerns |
| 302 | Low risk | Some concerns | Low risk | Low risk | Low risk | Some concerns |
| 303 | Low risk | Some concerns | Low risk | Low risk | Low risk | Some concerns |
| 304 | Low risk | Some concerns | Low risk | Low risk | Low risk | Some concerns |
| 305 | Low risk | Some concerns | Low risk | Low risk | Low risk | Some concerns |
| 306 | Low risk | Some concerns | Low risk | Low risk | Low risk | Some concerns |
| 307 | Low risk | Some concerns | Low risk | Low risk | Low risk | Some concerns |
| 308 | Low risk | Some concerns | Low risk | Low risk | Low risk | Some concerns |
| 309 | Low risk | Some concerns | Low risk | Low risk | Low risk | Some concerns |
| 311 | Low risk | Some concerns | Low risk | Low risk | Low risk | Some concerns |
| 313 | Low risk | Some concerns | Low risk | Low risk | Low risk | Some concerns |
| 314 | Low risk | Some concerns | Low risk | Low risk | Low risk | Some concerns |
| 315 | Low risk | Some concerns | Low risk | Low risk | Low risk | Some concerns |
| 317 | Low risk | Some concerns | Low risk | Low risk | Low risk | Some concerns |
| 318 | Low risk | Some concerns | Low risk | Low risk | Low risk | Some concerns |
| 319 | Low risk | Some concerns | Low risk | Low risk | Low risk | Some concerns |
| 320 | Low risk | Some concerns | Low risk | Low risk | Low risk | Some concerns |
| 321 | Low risk | Some concerns | Low risk | Low risk | Low risk | Some concerns |
| 322 | Low risk | Some concerns | Low risk | Low risk | Low risk | Some concerns |
| 323 | Low risk | Some concerns | Low risk | Low risk | Low risk | Some concerns |
| 324 | Low risk | Some concerns | Low risk | Low risk | Low risk | Some concerns |
| 325 | Low risk | Some concerns | Low risk | Low risk | Low risk | Some concerns |
| 326 | Low risk | Some concerns | Low risk | Low risk | Low risk | Some concerns |
| 327 | Low risk | Some concerns | Low risk | Low risk | Low risk | Some concerns |
| 328 | Low risk | Some concerns | Low risk | Low risk | Low risk | Some concerns |
| 329 | Low risk | Some concerns | Low risk | Low risk | Low risk | Some concerns |
| 330 | Low risk | Some concerns | Low risk | Low risk | Low risk | Some concerns |
| 331 | Low risk | Some concerns | Low risk | Low risk | Low risk | Some concerns |
| 332 | Low risk | High risk | Low risk | High risk | Low risk | High risk |
| 333 | Low risk | Some concerns | Low risk | Low risk | Low risk | Some concerns |
| 334 | Low risk | Some concerns | Low risk | Low risk | Low risk | Some concerns |
| 336 | Low risk | Some concerns | Low risk | Low risk | Low risk | Some concerns |
| 337 | Low risk | Some concerns | Low risk | Low risk | Low risk | Some concerns |
| 338 | Low risk | Some concerns | Low risk | Low risk | Low risk | Some concerns |
| 339 | Low risk | Low risk | Low risk | Low risk | Low risk | Low risk |
| 340 | Low risk | Some concerns | Low risk | Low risk | Low risk | Some concerns |
| 341 | Low risk | Some concerns | Low risk | Low risk | Low risk | Some concerns |
| 342 | Low risk | Some concerns | Low risk | Low risk | Low risk | Some concerns |
| 343 | Low risk | Some concerns | Low risk | Low risk | Low risk | Some concerns |
| 345 | Low risk | High risk | Low risk | Low risk | Low risk | High risk |
| 348 | Low risk | Some concerns | Low risk | Low risk | Low risk | Some concerns |
| 349 | Low risk | Some concerns | Low risk | Low risk | Low risk | Some concerns |
| 350 | Low risk | Some concerns | Low risk | Low risk | Low risk | Some concerns |
| 351 | Low risk | Some concerns | Low risk | Low risk | Low risk | Some concerns |
| 352 | Low risk | Some concerns | Low risk | Low risk | Low risk | Some concerns |
| 353 | Low risk | Some concerns | Low risk | Low risk | Low risk | Some concerns |
| 354 | High risk | High risk | Low risk | High risk | Low risk | High risk |
| 355 | Low risk | Some concerns | Low risk | Low risk | Low risk | Some concerns |
| 356 | Low risk | Some concerns | Low risk | Low risk | Low risk | Some concerns |
| 357 | Low risk | Some concerns | Low risk | Low risk | Low risk | Some concerns |
| 358 | Low risk | Some concerns | Low risk | Low risk | Low risk | Some concerns |
| 359 | Low risk | Some concerns | Low risk | Low risk | Low risk | Some concerns |
| 360 | Low risk | Some concerns | Low risk | High risk | Low risk | High risk |
| 363 | Low risk | Some concerns | Low risk | Low risk | Low risk | Some concerns |
| 364 | Low risk | Some concerns | Low risk | Low risk | Low risk | Some concerns |
| 367 | Low risk | Some concerns | Low risk | Low risk | Low risk | Some concerns |
| 369 | Low risk | Some concerns | Low risk | Low risk | Low risk | Some concerns |
| 370 | Low risk | Some concerns | Low risk | High risk | Low risk | High risk |
| 371 | Low risk | Some concerns | Low risk | Low risk | Low risk | Some concerns |
| 372 | Low risk | Some concerns | Low risk | Low risk | Low risk | Some concerns |
| 374 | Low risk | Some concerns | Low risk | Low risk | Low risk | Some concerns |
| 375 | Low risk | Low risk | Low risk | Low risk | Low risk | Low risk |
| 376 | Low risk | Some concerns | Low risk | Low risk | Low risk | Some concerns |
| 379 | Low risk | Some concerns | Low risk | Low risk | Low risk | Some concerns |
| 383 | Low risk | Some concerns | Low risk | Low risk | Low risk | Some concerns |
| 384 | Low risk | High risk | Low risk | High risk | Low risk | High risk |
| 388 | Low risk | Some concerns | Low risk | Low risk | Low risk | Some concerns |
| 389 | Low risk | Some concerns | Low risk | Low risk | Low risk | Some concerns |
| 391 | Low risk | Low risk | Low risk | Low risk | Low risk | Low risk |
| 392 | Low risk | Some concerns | Low risk | Low risk | Low risk | Some concerns |
| 393 | Low risk | Some concerns | Low risk | Low risk | Low risk | Some concerns |
| 394 | Low risk | Some concerns | Low risk | Low risk | Low risk | Some concerns |
| 396 | Low risk | Some concerns | Low risk | Low risk | Low risk | Some concerns |
| 397 | Low risk | Some concerns | Low risk | Low risk | Low risk | Some concerns |
| 400 | Low risk | Some concerns | Low risk | Low risk | Low risk | Some concerns |
| 401 | Low risk | Some concerns | Low risk | Low risk | Low risk | Some concerns |
| 402 | Low risk | Some concerns | Low risk | High risk | Low risk | High risk |
| 405 | Low risk | Some concerns | Low risk | Low risk | Low risk | Some concerns |
| 406 | Low risk | Some concerns | Low risk | Low risk | Low risk | Some concerns |
| 407 | Low risk | Some concerns | Low risk | Low risk | Low risk | Some concerns |
| 411 | Low risk | Some concerns | Low risk | Low risk | Low risk | Some concerns |
| 412 | Low risk | Some concerns | Low risk | Low risk | Low risk | Some concerns |
| 413 | Low risk | Some concerns | Low risk | Low risk | Low risk | Some concerns |
| 414 | Low risk | Some concerns | Low risk | Low risk | Low risk | Some concerns |
| 415 | Low risk | Some concerns | Low risk | Low risk | Low risk | Some concerns |
| 417 | Low risk | Some concerns | Low risk | Low risk | Low risk | Some concerns |
| 418 | Low risk | Low risk | Low risk | Low risk | Low risk | Low risk |
| 419 | Low risk | Some concerns | Low risk | Low risk | Low risk | Some concerns |
| 420 | Low risk | Some concerns | Low risk | Low risk | Low risk | Some concerns |

**Supplementary 7 — GRADE Assessment for Network Evidence**

**Table S 7.1 GRADE Assessment for 6MWT**

| Comparison | Number of studies | Within-study bias | Reporting bias | Indirectness | Imprecision | Heterogeneity | Incoherence | Confidence rating |
| --- | --- | --- | --- | --- | --- | --- | --- | --- |
| AE:BT | 1 | Major concerns | Some concerns | No concerns | Some concerns | Some concerns | No concerns | Very Low |
| AE:CST | 1 | Some concerns | Some concerns | No concerns | Major concerns | No concerns | No concerns | Very Low |
| AE:GT | 3 | Some concerns | Some concerns | No concerns | No concerns | Major concerns | No concerns | Very Low |
| AE:HIIT | 3 | Some concerns | Some concerns | No concerns | Some concerns | No concerns | No concerns | Very Low |
| AE:MBE | 1 | Some concerns | Some concerns | No concerns | No concerns | Major concerns | No concerns | Very Low |
| AE:NE | 2 | Some concerns | Some concerns | No concerns | No concerns | No concerns | No concerns | Low |
| AE:RC | 10 | Some concerns | Some concerns | No concerns | No concerns | Some concerns | No concerns | Very Low |
| AE:TOT | 1 | Some concerns | Some concerns | No concerns | Some concerns | Some concerns | No concerns | Very Low |
| AE:ULT | 2 | Some concerns | Some concerns | No concerns | No concerns | Major concerns | Major concerns | Very Low |
| AE:VRG | 1 | Some concerns | Some concerns | No concerns | Some concerns | Some concerns | No concerns | Very Low |
| BT:FT | 1 | Major concerns | Some concerns | No concerns | Some concerns | Some concerns | No concerns | Very Low |
| BT:MBE | 1 | Some concerns | Some concerns | No concerns | Some concerns | Some concerns | No concerns | Very Low |
| CIMT:GT | 1 | Some concerns | Some concerns | No concerns | Some concerns | Some concerns | Major concerns | Very Low |
| CIMT:RC | 1 | No concerns | Some concerns | No concerns | No concerns | Some concerns | Some concerns | Very Low |
| CST:HIIT | 1 | Major concerns | Some concerns | No concerns | Some concerns | Some concerns | No concerns | Very Low |
| ESX:GT | 2 | No concerns | Some concerns | No concerns | Some concerns | No concerns | No concerns | Low |
| ESX:RC | 1 | Some concerns | Some concerns | No concerns | No concerns | No concerns | No concerns | Low |
| FT:NE | 2 | Some concerns | Some concerns | No concerns | No concerns | No concerns | No concerns | Low |
| FT:RC | 9 | Some concerns | Some concerns | No concerns | No concerns | Some concerns | No concerns | Very Low |
| FT:RT | 1 | Some concerns | Some concerns | No concerns | No concerns | Major concerns | No concerns | Very Low |
| FT:TOT | 1 | Some concerns | Some concerns | No concerns | Some concerns | Some concerns | No concerns | Very Low |
| FT:ULT | 2 | Some concerns | Some concerns | No concerns | Some concerns | Some concerns | Major concerns | Very Low |
| GT:MBE | 2 | Some concerns | Some concerns | No concerns | No concerns | Major concerns | Major concerns | Very Low |
| GT:NPF | 2 | Some concerns | Some concerns | No concerns | No concerns | Some concerns | No concerns | Very Low |
| GT:RAT | 4 | Some concerns | Some concerns | No concerns | No concerns | Major concerns | No concerns | Very Low |
| GT:RC | 9 | Some concerns | Some concerns | No concerns | No concerns | Some concerns | No concerns | Very Low |
| GT:TCMEX | 1 | Some concerns | Some concerns | No concerns | Some concerns | Some concerns | No concerns | Very Low |
| GT:TOT | 1 | Some concerns | Some concerns | No concerns | Some concerns | Some concerns | No concerns | Very Low |
| GT:VRG | 1 | Some concerns | Some concerns | No concerns | Some concerns | Some concerns | No concerns | Very Low |
| HIIT:RC | 1 | Some concerns | Some concerns | No concerns | No concerns | Some concerns | No concerns | Very Low |
| HIIT:TOT | 1 | Some concerns | Some concerns | No concerns | Some concerns | Some concerns | No concerns | Very Low |
| LLT:MBE | 2 | Some concerns | Some concerns | No concerns | Some concerns | Some concerns | Major concerns | Very Low |
| LLT:RC | 1 | Some concerns | Some concerns | No concerns | No concerns | Some concerns | No concerns | Very Low |
| LLT:ULT | 1 | Some concerns | Some concerns | No concerns | Some concerns | No concerns | Some concerns | Very Low |
| MBE:NE | 1 | Some concerns | Some concerns | No concerns | No concerns | No concerns | No concerns | Very Low |
| MBE:NPF | 1 | Some concerns | Some concerns | No concerns | No concerns | Some concerns | No concerns | Very Low |
| MBE:RC | 6 | Some concerns | Some concerns | No concerns | No concerns | Some concerns | No concerns | Very Low |
| MBE:VRG | 1 | Some concerns | Some concerns | No concerns | Some concerns | Some concerns | No concerns | Very Low |
| NE:RAT | 1 | Some concerns | Some concerns | No concerns | No concerns | Some concerns | No concerns | Very Low |
| NE:RC | 1 | Some concerns | Some concerns | No concerns | No concerns | Some concerns | No concerns | Very Low |
| NE:RT | 2 | Some concerns | Some concerns | No concerns | No concerns | No concerns | No concerns | Low |
| NE:VRG | 1 | Some concerns | Some concerns | No concerns | Some concerns | No concerns | No concerns | Very Low |
| NPF:RT | 1 | Some concerns | Some concerns | No concerns | No concerns | Some concerns | No concerns | Very Low |
| NPF:TOT | 1 | Some concerns | Some concerns | No concerns | No concerns | Some concerns | No concerns | Very Low |
| NPF:ULT | 1 | Some concerns | Some concerns | No concerns | No concerns | Some concerns | No concerns | Very Low |
| NPF:VT | 1 | Some concerns | Some concerns | No concerns | No concerns | Some concerns | Major concerns | Very Low |
| RAT:RC | 2 | Some concerns | Some concerns | No concerns | Some concerns | No concerns | No concerns | Very Low |
| RAT:ULT | 1 | Some concerns | Some concerns | No concerns | No concerns | Major concerns | Major concerns | Very Low |
| RC:RT | 6 | Some concerns | Some concerns | No concerns | No concerns | Some concerns | No concerns | Very Low |
| RC:TCMEX | 1 | Some concerns | Some concerns | No concerns | Major concerns | No concerns | No concerns | Very Low |
| RC:TOT | 3 | Some concerns | Some concerns | No concerns | Some concerns | No concerns | No concerns | Very Low |
| RC:VRG | 1 | Some concerns | Some concerns | No concerns | Some concerns | Some concerns | No concerns | Very Low |
| RC:WA | 2 | Some concerns | Some concerns | No concerns | Some concerns | Some concerns | Major concerns | Very Low |
| RT:ULT | 1 | Some concerns | Some concerns | No concerns | Some concerns | Some concerns | No concerns | Very Low |
| TOT:ULT | 1 | Some concerns | Some concerns | No concerns | Some concerns | Some concerns | Major concerns | Very Low |
| AE:CIMT | 0 | Some concerns | Some concerns | No concerns | Some concerns | Some concerns | Major concerns | Very Low |
| AE:ESX | 0 | Some concerns | Some concerns | No concerns | Some concerns | No concerns | Major concerns | Very Low |
| AE:FT | 0 | Some concerns | Some concerns | No concerns | No concerns | Major concerns | Major concerns | Very Low |
| AE:LLT | 0 | Some concerns | Some concerns | No concerns | Some concerns | Some concerns | Major concerns | Very Low |
| AE:NPF | 0 | Some concerns | Some concerns | No concerns | No concerns | Some concerns | Major concerns | Very Low |
| AE:RAT | 0 | Some concerns | Some concerns | No concerns | No concerns | Major concerns | Major concerns | Very Low |
| AE:RT | 0 | Some concerns | Some concerns | No concerns | No concerns | Major concerns | Major concerns | Very Low |
| AE:TCMEX | 0 | Some concerns | Some concerns | No concerns | Some concerns | Some concerns | Major concerns | Very Low |
| AE:VT | 0 | Some concerns | Some concerns | No concerns | Major concerns | No concerns | Major concerns | Very Low |
| AE:WA | 0 | Some concerns | Some concerns | No concerns | Major concerns | No concerns | Major concerns | Very Low |
| BT:CIMT | 0 | Some concerns | Some concerns | No concerns | Some concerns | Some concerns | Major concerns | Very Low |
| BT:CST | 0 | Some concerns | Some concerns | No concerns | Major concerns | No concerns | Major concerns | Very Low |
| BT:ESX | 0 | Some concerns | Some concerns | No concerns | Some concerns | No concerns | Major concerns | Very Low |
| BT:GT | 0 | Some concerns | Some concerns | No concerns | Some concerns | Some concerns | Major concerns | Very Low |
| BT:HIIT | 0 | Some concerns | Some concerns | No concerns | Some concerns | No concerns | Major concerns | Very Low |
| BT:LLT | 0 | Some concerns | Some concerns | No concerns | Some concerns | No concerns | Major concerns | Very Low |
| BT:NE | 0 | Some concerns | Some concerns | No concerns | Some concerns | No concerns | Major concerns | Very Low |
| BT:NPF | 0 | Some concerns | Some concerns | No concerns | Some concerns | Some concerns | Major concerns | Very Low |
| BT:RAT | 0 | Some concerns | Some concerns | No concerns | Some concerns | Some concerns | Major concerns | Very Low |
| BT:RC | 0 | Some concerns | Some concerns | No concerns | Some concerns | Some concerns | Major concerns | Very Low |
| BT:RT | 0 | Some concerns | Some concerns | No concerns | Some concerns | Some concerns | Major concerns | Very Low |
| BT:TCMEX | 0 | Some concerns | Some concerns | No concerns | Major concerns | No concerns | Major concerns | Very Low |
| BT:TOT | 0 | Some concerns | Some concerns | No concerns | Some concerns | Some concerns | Major concerns | Very Low |
[truncated: 398,004 more chars]
